# The great diversity: monomeric and oligomeric hirudins, hirudin-like factors and decorsins in the Asian medicinal leeches *Hirudo nipponia* and *Hirudo tianjinensis*

**DOI:** 10.1007/s00436-026-08634-0

**Published:** 2026-02-07

**Authors:** Pegah Kalatehjari, Robert Wolf, Gabriele Jedlitschky, Céline Tolksdorf, Bernhard H. Rauch, Christian Müller

**Affiliations:** 1 Animal Physiology, Zoological Institute and Museum, University of Greifswald, 17489 Greifswald, Germany; 2 Centre for Microbiology and Environmental Systems Science, Division of Microbial Ecology, University of Vienna, Vienna, 1030 Austria; 3 Section of Nephrology, Clinic and Policlinic of Internal Medicine A, University Medicine Greifswald, Ferdinand-Sauerbruch-Straße, 17475 Greifswald, Germany; 4 Department of General Pharmacology, Center of Drug Absorption and Transport (C_DAT), University Medicine Greifswald, 17489 Greifswald, Germany; 5 Pharmacology and Toxicology, University Medicine Oldenburg, Carl von Ossietzky University Oldenburg, 26129 Oldenburg, Germany

**Keywords:** Hirudin, Ornatin, Blood coagulation, Platelet aggregation, Hematophagous leeches, Phylogeny

## Abstract

Medicinal leeches express a broad variety of anticoagulants and other bioactive factors that are involved in the blood feeding process. For most of the anticoagulants, several genes exist that may encode isoforms of the respective proteins, including hirudins and decorsins. Decorsins negatively affect platelet aggregation, whereas hirudins are potent thrombin inhibitors. Both factors belong to the hirudin superfamily that also includes the group of hirudin-like factors, and all hematophagous leeches analyzed so far contain several gene copies that encode representatives of at least two of the three groups of these factors. Members of the hirudin superfamily may contain only one central globular domain, but others may contain two or more copies. Here we describe the molecular identification and partial functional characterization of a broad variety of putative mono-, bi and multimeric hirudins, decorsins and hirudin-like factors in two Asian medicinal leech species, namely *Hirudo nipponia* and *H. tianjinensis*. Some of the monomeric hirudins and decorsins have already been described before, but they represent only a small part of the overall diversity. For the first time, putative monomeric and one oligomeric decorsins of *H. tianjinensis* were expressed as recombinant proteins, functionally characterized and successfully verified as platelet aggregation inhibitors. In addition we have conducted phylogenetic analyses based on genomic and mitochondrial markers and found convincing evidence that *H. nipponia* and *H. tianjinensis* together with members of the genus *Whitmania* form a monophyletic clade that is clearly distinct from clades that are formed either by European members of the genus *Hirudo* or by members of the genus *Hirudinaria*.

**Supplementary Information:**

The online version contains supplementary material available at 10.1007/s00436-026-08634-0.

## Introduction

Hematophagous leeches, also referred to as medicinal leeches, do not form a monophyletic group, but rather comprise members that originate from very different lineages including the genera *Hirudo* Linnaeus, 1758, *Hirudinaria* Whitman, 1886, *Asiaticobdella* Richardson, 1969, *Limnobdella* Blanchard, 1893 and *Macrobdella* Verrill, 1872, respectively (Philiips and Siddall 2009). Medicinal leeches have been used for therapeutic purposes in numerous human societies and cultures for millennia (Abdulalkader et al. 2013; Dong et al. 2016; Elliott and Kutschera 2011; Lemke and Vilcinskas 2020; Munshi et al. 2008; Okka 2013; Wittke-Michalsen 2007). During feeding, leeches inject a broad variety of bioactive substances into the host to promote a largely undisturbed intake of their blood meal (Gross and Roth 2007; Hildebrandt and Lemke 2011; Shakouri and Wollina 2021). Leech saliva is produced and stored in salivary gland cells that are located near the anterior sucker of the animals (Lemke et al. 2013; Saglam et al. 2020) and may contain more than 100 different protein components (Baskova et al. 2004; Liu et al. 2019). However, only a few of them have been isolated and functionally characterized so far. Among them, hirudins are the most prominent examples and the only leech-derived bioactive factors that are in regular clinical use (Markwardt 1991; Greinacher and Warkentin 2008). Hirudins are highly effective thrombin inhibitors that hence specifically address the blood coagulation cascade (Markwardt 1970; Stone and Hofsteenge 1986; Grütter et al. 1990; Lindhout et al. 1990). Hirudin was first identified in the European medicinal leech *Hirudo medicinalis* Linnaeus 1758 (Haycraft 1884; Jacobj 1904; Markwardt 1955), but all hematophagous leeches analyzed to date express hirudins or functionally related factors like the haemadins (Strube et al. 1993). Both hirudins and haemadins belong to the hirudin superfamily (Müller et al. 2024) that additionally includes the leech-derived decorsins (Müller et al. 2019) and the hirudin-like factors (HLFs; Müller et al. 2017). Decorsins are inhibitors of platelet aggregation (Seymour et al. 1990). The biological function of HLFs, however, remains largely elusive (Müller et al. 2020). Hematophagous leeches like the American representatives *Limnobdella mexicana* Blanchard, 1893 and *Haementeria vizottoi* Castro, 1971 express hirudins, decorsins and HLFs (Amorim et al. 2015; Iwama et al. 2019; Pfordt et al. 2022), whereas the European members of the genus *Hirudo* apparently lack decorsin-encoding genes.

The archetypes of hirudin, decorsin and the HLFs are monomeric factors in a sense that they contain a single central globular domain that comprises six conserved cysteine residues, a hallmark of all members of the hirudin superfamily (Dodt et al. 1984; De Filippis et al. 1995). The central globular domain in turn is concordantly encoded by two exons, namely exons 2 and 3 of the respective genes (Scacheri et al. 1993; Müller et al. 2016; Ben Ahmed et al. 2024). The genetic structure of the exons, however, opens up the possibility of multiplication – a phenomenon that was first described for the tandem hirudin (TH) of *Hirudinaria manillensis* Lesson, 1842 (Lukas et al. 2022). The multiplication hypothesis was later confirmed by the identification and functional characterization of a multimeric decorsin in *Haemadipsa interrupta* Moore, 1935 (Müller et al. 2024) and by the identification of six individual TH-encoding genes in the genome of *Hirudinaria bpling* Phillips, 2012 (Khan et al. 2025).

The leech fauna of East Asia comprises several genera including *Hirudinaria*, *Haemadipsa* Tennent, 1859 and *Whitmania* Blanchard, 1887. Members of the genera *Hirudinaria* and *Haemadipsa* are hematophagous, whereas members of the genus *Whitmania* are macrophagous leeches that mainly feed on snails (Khan et al. 2019; Luo et al. 2022). Interestingly, both *W. pigra* Whitman, 1884 and *W. acranulata* Whitman, 1886 are ingredients of the Traditional Chinese Medicine (TCM) and are listed in the current *Chinese Pharmacopeia* for the treatment of cardiovascular diseases with a focus on the promotion of blood circulation and the prevention of blood stasis (Dong et al. 2016). *W. pigra* encodes both hirudins and hirudin-like factors (Müller et al. 2022), but interestingly the respective genes are absent in *W. acranulata* (Zhao et al. 2025). A third leech that is listed in the current *Chinese Pharmacopeia* is *Hirudo nipponia* Whitman, 1886. In contrast to members of the genus *Whitmania*, *H. nipponia* is hematophagous and is hence considered as an Asian medicinal leech (Hong et al. 1999). An in-depth analysis of transcriptome datasets of *H. nipponia* revealed clear evidence for the expression of putative hirudins, but indicated that the respective cDNA libraries (Lu et al. 2018; Cheng et al. 2019) were generated from leech individuals that belonged to either *H. nipponia* (named *H. nipponia* genotype 2 or *H. nipponia* sensu stricto) or a yet undescribed leech species (named *H. nipponia* genotype 1) (Müller et al. 2022). Recently a sister species to *H. nipponia* was described, namely *Hirudo tianjinensis* Wang et al. 2022 (Wang et al. 2022)d *tianjinensis* is identical to *H. nipponia* genotype 1 in Müller et al. (2022). In 2024, Zhao and colleagues presented a comparative genome analysis of *H. nipponia* and *H. tianjinensis*, and the authors identified several putative monomeric hirudin, decorsin and HLF genes in both species, but did not find evidence for the presence of multimeric variants of the respective factors (Zhao et al. 2024). In addition, the authors performed blood coagulations assays to functionally characterize the putative hirudins.

*H. nipponia* is currently classified in the same genus as the European medicinal leeches, but phylogenetic analyses based on molecular markers point to a closer relationship with the genus *Whitmania* (Phillips and Siddall 2009). A comprehensive analysis to clarify the exact relationships, however, remains to be done.

Aim of the present study was to screen the various genome and transcriptome data sets of both *H. nipponia* and *H. tianjinensis* for the presence of additional monomeric and multimeric hirudin, decorsin and HLF genes and to functionally characterize promising candidates. In addition we re-evaluated the phylogenetic relationships between members of the genera *Hirudo*, *Hirudinaria* and *Whitmania* using both genomic and mitochondrial markers to clarify whether or not *H. nipponia* and *H. tianjinensis* belong to the genus *Hirudo* or to the genus *Whitmania*.

## Materials and methods

### Transcriptome and genome data

Transcriptome data of *H. nipponia* and *H. tianjinensis* were obtained from GenBank sequence read archives (SRA) SRX3466461 (Lu et al. 2018), SRX4283435 (Cheng et al. 2019),SRX16766562 (Shen et al. 2022) and SRR26541739 (Zhao et al. 2024), genome data were obtained from GenBank bioprojects PRJNA762643 (Zheng et al. 2023) and PRJNA1033322 (Zhao et al. 2024).

### Bioinformatics and graphical tools

Basic Local Alignment Search Tool (BLAST) searches were conducted using the NCBI web portal (https://blast.ncbi.nlm.nih.gov/Blast.cgi) or BioEdit v7.2.5 (Hall 1999) and adjusted parameter settings for both word size and the expected threshold values. Multiple sequence alignment (MSA) files were generated using ClustalX 2.1 (Larkin et al. 2007) or the CLC Sequence Viewer software package v8.0 (QIAGEN, Aarhus, Denmark) using default settings. Particular attention was paid to the conservation of disulfide-forming cysteines. Alignments were exported as msf-files and further processed using Gene-Doc v2.7 (Nicholas and Nicholas 1997). The phylogenetic tree constructions were performed using the UPGMA algorithm (Michener and Sokal 1957) and the Jukes-Cantor model (Jukes and Cantor 1969) embedded into the CLC Sequence Viewer software package v8.0. Signal peptide sequences were predicted using the SignalP6.0 server (Teufel et al. 2022) and the Phobius web portal (Käll et al. 2007). Graphs were generated and analyzed using GraphPad Prism V5.01 (GraphPad Software, Boston, MA, USA).

### Gene synthesis

cDNA fragments of putative decorsins were generated using the GeneArt gene synthesis service of ThermoFisher Scientific (Darmstadt, Germany).

### Amplification and cloning of putative hirudin and decorsin cDNAs

For the amplification of putative hirudins and decorsin cDNAs, primers were derived from the respective transcriptome or genome sequences. A list of all primers that were used in the study is provided in Supplementary Information File Table S1. PCR reactions were performed using Q5 high-fidelity DNA polymerase (New England Biolabs, Frankfurt a. M., Germany), fragments of relevant sizes were purified and cloned into the expression vector pQE30Xa (QIAGEN, Hilden, Germany). Successfully cloned cDNAs were sequenced for control purposes by Biosearch Technologies (LGC, Berlin, Germany) or by Eurofins Genomics (Cologne, Germany).

### Expression, purification, processing, and quantification of putative hirudins and decorsins

The detailed procedure to express, purify, process and quantify the respective recombinant hirudins, decorsins and HLFs of *H. nipponia* and *H. tianjinensis* was described in numerous recent publications (e.g. Müller et al. 2016; Pfordt et al. 2022; Wang et al. 2025). Briefly, all factors were expressed in the laboratory strain *Escherichia coli* DH5α (Hanahan 1985). To obtain the recombinant proteins we applied an expression and purification system that was developed by QIAGEN (Hilden, Germany). The pQE30Xa vector encodes a factor Xa protease recognition site that is located between the His-tag coding region at the 5′ side and the multiple cloning site at the 3′ side. A subsequent factor Xa protease-treatment cleaves off the His-tag and results in a recombinant protein that is devoid of any vector-derived amino acid residues at the N-terminus. The successful expression, purification and factor Xa treatment of all factors was controlled by sodium dodecyl sulfate-polyacrylamide gel electrophoresis (SDS-PAGE) analyses. Molar concentrations of final protein solutions were calculated by dividing the absorbance at 280 nm by the molar absorption coefficient according to the equation ε = (nW × 5,500) + (nY × 1,490) + (nC × 125) (Gill and von Hippel 1989; Pace et al. 1995).

### Blood coagulation assays

To test the thrombin-inhibitory potencies of putative hirudins of *H. nipponia* and *H. tianjinensis*, we performed the thrombin time test (TTT; reference range 16.8–21.4 s) using a BFT II analyzer (Siemens Healthcare, Erlangen, Germany). All steps were carried out according to the manufacturer’s instructions. Protein samples were diluted with buffer to reach final concentrations in the reaction assays of 3.2 µmol/l or 0.32 µmol/l, respectively. The desired amount of substrate was directly transferred into the test cuvette immediately before the plasma was added. Dade^®^ Ci-Trol^®^ 1 (Siemens Healthcare, Erlangen, Germany) was used as standardized human plasma. The incubation of reaction mixtures was carried out at 37.4 °C. Measurements that reached 300 s before any coagulation was detected were stopped and considered as complete inhibition of coagulation. Blood coagulation tests were performed in three to four technical replicates.

### Platelet aggregation assays

All assays were performed with human blood samples that were obtained from healthy human volunteers after written informed consent and approval from the institutional ethics committee. Blood collection, sample preparation, and the subsequent experimental procedure were performed as described in Müller et al. (2024) and Schulz et al. (2025). Briefly, 10 ml of venous blood was taken from the antecubital vein using an S-Monovette^®^ (Sarstedt, Nürnbrecht, Germany) prefilled with citrate buffer. The first centrifugation step of the blood collection tube was performed at 200 g for 20 min at room temperature. After centrifugation, the supernatant (platelet-rich plasma, PRP) was transferred, and the remaining blood was centrifuged again for 10 min at 2000 g and room temperature. The supernatant was dedicated as platelet-poor plasma (PPP), transferred, and used as a reference value for maximal platelet aggregation. Measurements were performed using either a TA-8 V aggregometer (Diagnostica Stago S.A.S., Asnièressur-Seine, France) or an APACT-4004 aggregometer (LABiTec, Ahrensburg, Germany). The snake venom-derived platelet aggregation inhibitors tirofiban and eptifibatide (Sigma-Aldrich, Taufkirchen, Germany) were used as positive controls for complete inhibition of platelet aggregation. PRP was pre-incubated with the respective test and control compounds (final concentration 3.2 µmol/l) or buffer for 1 min at 37 °C. For the measurement, PRP was then transferred into test cuvettes and stimulated with ADP (200 µmol/l; Hart Biologicals, Hartlepool, UK; final concentration 5 µmol/l) after 1 min of runtime. The final volume in each test cuvette was 250 µL of diluted PRP. All experiments were performed at 37 °C over a time period of 400 s. Maximal aggregation in percentage and the area under the curve were calculated as quantitative output parameters (Zhou and Schmaier 2005). Platelet aggregation tests were performed in two to four technical replicates.

## Results

### Identification of monomeric and multimeric hirudin and HLF genes in*H. nipponia* and*H. tianjinensis*

Putative monomeric hirudin- and HLF-encoding cDNAs and genes of *H. nipponia* and *H. tianjinensis* were identified and descriebed in several reports over the last years, and some of the factors were already functionally characterized. Unfortunately, the correct species allocation is questionable for some of the reports, and only recently the reference genomes for both *H. nipponia* and *H. tianjinensis* were defined (Zhao et al. 2024). We have collected all available information (source, original denomination and functional characterization) on the various putative hirudins and HLFs, corrected the species allocation when necessary, assigned identical factors and summarized the data in Table 1.

**Table 1:** Identification and annotation of putative monomeric Hirudin and HLF genes in *H. nipponia* and *H. tianjinensis*. * indicates functional characterization; + indicates thrombin inhibitory potency; - indicates absence of thrombin inhibitory potency

species	Lu et al. 2018	Cheng et al. 2019	Fan et al. 2021	Müller et al. 2022	Zheng et al. 2023	Zhao et al. 2024/ Yin et al. 2025	this study
*H. nipponia*	-	-	-	Hnip_V5	-	hirudin_Hnip1*+	Hnip_HV1*-
	Hnip hirudin	-	-	Hnip_V3a	-	hirudin_Hnip2*+	Hnip_HV2*+
	-	-	-	-	-	hirudin_Hnip3*-	Hnip_HV3
	-	-	-	-	-	-	Hnip_HV4
	-	-	-	-	-	-	Hnip_HV5
	-	-	-	-	-	-	Hnip_HV6
	-	-	-	-	-	-	Hnip_HV7
*H. tianjinensis*	-	Hirudin-HN*+	-	Hnip_V1/Wpig_V5*+	HN-hirudin	hirudin_Htia1*+	Htia_HV1
	-	-	HLF-HN	Hnip_V2/Wpig_V4*+	-	hirudin_Htia2*+	Htia_HV2
	-	-	-	-	-	hirudin_Htia3*-	Htia_HV3
	-	-	-	Hnip_V4	-	-	Htia_HV4
	-	-	-	-	-	-	Htia_HV5*-
	-	-	-	-	-	-	Htia_HV6
	-	-	-	-	-	-	Htia_HV7

Zhao et al. (2024) identified three individual genes that encode putative monomeric hirudins or HLFs in both *H. nipponia* (Hnip) and *H. tianjinensis* (Htia), located on either chromosome 5 (Hnip) or chromosome 6 (Htia), respectively. We conducted in-depth analyses of both genome sequence data sets and confirmed the observation, but identified a fourth putative HLF gene on chromosome 6 of *H. tianjinensis*. The respective factor was already described in Müller et al. (2022) as Hnip_V4, but was renamed to Htia_HV4 in the present study (see Table 1).

Furthermore we identified a multitude of additional mono-, di-, oligo- and multimeric putative hirudin and HLF genes that are concordantly located on chromosome 2 in both leech species. The number of repeats of the central globular domain ranges from 1 to 16 in *H. nipponia* and from 1 to 18 in *H. tianjinensis*. The putative monomeric hirudins/HLFs were named Hnip_HV4-7 and Htia_HV4-7 (see Table 1), whereas the the di-, oligo- and multimeric factors were designated Hnip_mHV1-8 and Htia_mHV1-16, respectively. Three putative hirudin/HLF genes in *H. tianjinensis* contain stop codons within exons and are hence pseudogenes. To verify the genome sequence-based gene annotations, salivary gland transcriptome data sets of both leech species were analyzed for the expression of the respective monomeric and multimeric putative hirudin and HLF genes. Strikingly, all genes but Hnip_HV7 and Hnip_mHV2 are expressed in the salivary glands (see Supplementary Information File Table S2 for details). The exact positions, orientations, numbers of repeats of the central globular domain, isoelectric point (pI) and molecular mass (MW) values of the deduced proteins and the annotations of all putative hirudin and HLF genes in both leech species are summarized in Table 2.

**Table 2:** Localization, characterization and annotation of putative monomeric and multimeric hirudin/HLF genes in *H. nipponia* (A and B) and *H. tianjinensis* (C and D). Repeats provide the number of repetitive sequences encoding central globular domains within individual genes; pI indicates the isoelectric point values; MW indicates the molecular mass values; Fw indicates forward orientation, rev indicates reverse + complementary orientation; * indicates pseudogenes

A) Monomeric and multimeric hirudin genes on chromosome 2 of H. nipponia					
Position	Orientation	Repeats	pI	MW	Annotation
18,942,726–18,943,470	fw	1	8.20	8353.82	Hnip_HV4
18,971,422–18,972,160	rev	1	4.78	8111.58	Hnip_HV5
19,153,472–19,159,914	fw	12	7.53	54088.23	Hnip_mHV1
19,165,680–19,167,819	fw	1	4.54	5920.51	Hnip_HV6
19,165,680–19,167,819	fw	2	5.04	9352.51	Hnip_mHV2
19,188,245–19,194,790	rev	12	6.90	54262.54	Hnip_mHV3
19,324,607–19,336,935	rev	16	5.30	71774.46	Hnip_mHV4
19,343,623–19,344,105	rev	1	4.82	4797.26	Hnip_HV7
19,341,815–19,344,105	rev	4	5.38	18611.01	Hnip_mHV5
19,349,205–19,355,228	rev	5	8.69	23277.88	Hnip_mHV6
19,353,002–19,355,228	rev	2	8.97	9511.09	Hnip_mHV7
19,456,932–19,457,750	fw	2	8.82	12635.44	Hnip_mHV8
B) Monomeric hirudin genes on chromosome 5 of *H. nipponia*					
Position	Orientation	Repeats	pI	MW	Annotation
15,392,527 − 15,391,957	fw	1	6.48	5101.80	Hnip_HV3
15,402,226–15,402,777	fw	1	8.87	4808.68	Hnip_HV1
15,468,609–15,469,291	fw	1	6.72	6972.75	Hnip_HV2
C) Monomeric and multimeric hirudin genes on chromosome 2 of *H. tianjinensis*					
Position	Orientation	Repeats	pI	MW	Annotation
1,267,539–1,270,337	fw	5	5.40	21539.29	Htia_mHV1
1,277,208–1,280,662	fw	7	5.43	31986.23	Htia_mHV2
1,308,787–1,311,525	rev	5	5.94	22434.28	Htia_mHV3
1,327,361–1,331,102	rev	8	4.83	36189.50	Htia_mHV4
1,445,023–1,449,948	fw	5	8.79	23307.06	Htia_mHV5*
1,453,196–1,454,013	fw	1	9.34	5391.34	Htia_HV5
1,460,097–1,463,027	fw	5	6.93	20885.72	Htia_mHV6
1,467,292–1,473,689	fw	11	5.10	48180.22	Htia_mHV7
1,480,329–1,483,822	fw	7	4.76	30888.89	Htia_mHV8
1,492,096–1,500,774	fw	12	4.65	49082.58	Htia_mHV9
1,505,921–1,514,733	fw	13	5.10	55204.16	Htia_mHV10
1,519,970–1,540,854	fw	17	5.30	74754.37	Htia_mHV11
1,547,058–1,559,837	fw	18	5.60	79896.76	Htia_mHV12
1,706,734–1,717,866	rev	11	5.33	49886.35	Htia_mHV13
1,728,676–1,738,104	rev	12	5.83	53033.53	Htia_mHV14*
1,745,847–1,751,224	rev	11	6.05	52124.85	Htia_mHV15
1,922,211–1,926,178	fw	9	4.70	37738.13	Htia_mHV16
1,971,445–1,972,201	rev	1	5.19	8362.83	Htia_HV6
1,985,268–1,986,029	fw	1	5.19	8387.83	Htia_HV7*
D) Monomeric hirudin genes on chromosome 6 of *H. tianjinensis*					
Position	Orientation	Repeats	pI	MW	Annotation
15,622,291–15,622,887	rev	1	7.79	5394.08	Htia_HV3
15,633,162–15,634,521	fw	1	4.84	7668.44	Htia_HV1
15,636,955–15,637,387	fw	1	8.63	5293.08	Htia_HV4
15,703,335–15,703,985	fw	1	5.22	6349.00	Htia_HV2

**Fig. 1:**
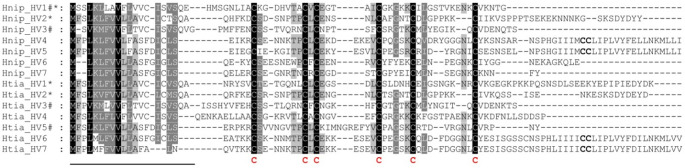
Multiple amino acid sequence alignments of putative monomeric hirudins and HLFs of *H. nipponia* (Hnip_HV1-HV7) and *H. tianjinensis* (Htia_HV1-HV7). A black background indicates fully conserved residues; a gray background indicates partially conserved residues. The six conserved cysteine residues giving rise to the three-dimensional structure of the central globular domain are marked in bold and red. The predicted signal peptide sequence is underlined. * indicates a thrombin inhibitory potency in a coagulation assay, # indicates the lack of a thrombin inhibitory potency. Abbreviations are used according to the IUPAC code

The complete nucleotide sequences including annotations of all genes and the deduced amino acid sequences of all proteins are provided as Supplementary Information Files S1 (hirudin/HLF genes on chromosome 2 of *H. nipponia*), S2 (hirudin/HLF genes on chromosome 2 of *H. tianjinensis*), S3 (hirudin/HLF genes on chromosome 5 of *H. nipponia*) and S4 (hirudin/HLF genes on chromosome 2 of *H. tianjinensis*), respectively. A multiple sequence alignment (MSA) of all putative monomeric hirudin and HLF proteins of both species is provided in Fig. 1.

### Identification of a multimeric decorsin gene in*H. nipponia* and *H. tianjinensis*

**Fig. 2:**
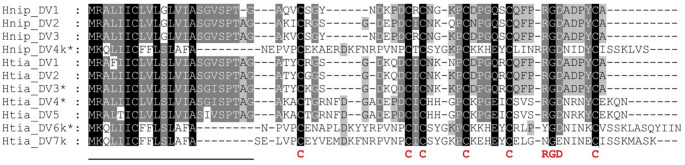
Multiple amino acid sequence alignments of putative monomeric decorsins of *H. nipponia* (Hnip_DV1-DV4k) and *H. tianjinensis* (Htia_DV1-HV7k). A black background indicates fully conserved residues; a gray background indicates partially conserved residues. The six conserved cysteine residues giving rise to the three-dimensional structure of the central globular domain and the conserved RGD motif are marked in bold and red. The predicted signal peptide sequence is underlined. k indicates a monomeric splice variant of the respective multimeric decorsin. * indicates the factors that were tested in a platelet aggregation assay. Abbreviations are used according to the IUPAC code

In addition to the putative hirudin/HLF genes, Zhao et al. (2024) also identified putative monomeric decorsin genes, namely decorsin_Hnip1-3 and decorsin_Htia1-5, respectively. Functional characterizations of all factors, however, are still pending. Our in-depth analyses of both genomes confirmed the presence of all putative monomeric genes (annotated as Hnip_DV1-3 and Htia_DV1-5), but we were able to identify genes that encode putative multimeric decorsins on chromosome 5 of *H. nipponia* (designated Hnip_DV4) and on chromosome 6 of *H. tianjinensis* (designated Htia_DV6 and DV7) as well. Both Hnip_DV4 and Htia_DV7 comprise four repeats of the central globular domain. Despite great efforts we failed to identify exon1 that encodes the signal peptide sequence of the Htia_DV6 gene. All putative monomeric decorsins and the multimeric Hnip_DV4 contain the conserved RGD motifs that are essential for the inhibition of platelet aggregation. In contrast, both Htia_DV6 and Htia_DV7 contain altered motifs (YGD in Htia_DV6, NGN and NGE in Htia_DV7) that may diminish the inhibitory effect of both factors on platelet aggregation (see Fig. 2 for details). Again, transcriptome data sets of both leech species were analyzed for the expression of the monomeric and multimeric putative decorsin genes, and all genes are expressed in the salivary glands (see Supplementary Information File Table S2). Luckily, the complete cDNA sequence of the Htia_DV6 gene including the signal peptide sequence could be assembled based on the transcriptome data of *H. tianjinensis*. The exact positions, orientations, numbers of repeats of the central globular domain, isoelectric point (pI) and molecular mass (MW) values of the deduced proteins and the annotations of all putative decorsin genes in both leech species are summarized in Table 3.

**Table 3:** Localization, characterization and annotation of putative monomeric and multimeric decorsin genes in *H. nipponia* (A) and *H. tianjinensis* (B). Repeats provide the number of repetitive sequences encoding central globular domains within individual genes; pI indicates the isoelectric point values; MW indicates the molecular mass values; Fw indicates forward orientation, rev indicates reverse + complementary orientation; * indicates an incomplete gene; k indicates a putative short splice variant and k1 and k2 indicate different short splice variants of the respective genes; incomplete genes are labeled in yellow

A) Monomeric and multimeric decorsin genes on chromosome 5 of H. nipponia					
Position	Orientation	Repeats	pI	MW	Annotation
15,256,672–15,257,744	fw	1	4.69	4067.46	Hnip_DV1
15,259,520–15,260,585	fw	1	4.78	4045.50	Hnip_DV2
15,262,002–15,263,038	fw	1	4.46	4068.44	Hnip_DV3
15,265,547–15,268,223	fw	4	8.78	23290.77	Hnip_DV4
15,265,547–15,267,732	fw	1	8.61	6220.11	Hnip_DV4k
B) Monomeric and multimeric decorsin genes on chromosome 6 of *H. tianjinensis*					
Position	Orientation	Repeats	pI	MW	Annotation
15,046,240–15,047,295	rev	1	6.91	4911.44	Htia_DV5
15,354,719–15,355,774	fw	1	6.05	4897.37	Htia_DV4
15,358,633–15,359,719	fw	1	7.79	4140.64	Htia_DV3
15,379,193–15,380,058	rev	4(2*)	8.92	23259.72	Htia_DV6
15,387,745–15,388,840	fw	1	6.14	4069.52	Htia_DV2
15,391,995–15,393,089	fw	1	6.14	4154.58	Htia_DV1
15,395,084–15,397,517	fw	4	8.66	23209.72	Htia_DV7

The complete nucleotide sequences including annotations of all genes and the deduced amino acid sequences of all putative monomeric and multimeric decorsins are provided as Supplementary Information Files S5 (decorsin genes on chromosome 5 of *H. nipponia*) and S6 (decorsin genes on chromosome 6 of *H. tianjinensis*), respectively. A multiple sequence alignment of all putative monomeric decorsins of both species is provided in Fig. 2.

Taken together, the number and diversity of putative hirudin/HLF and decorsin genes in both *H. nipponia* and *H. tianjinensis* is far more extensive than previously assumed, but we are aware that we still may have missed genes.

#### Functional characterization I: thrombin inhibition

**Fig. 3:**
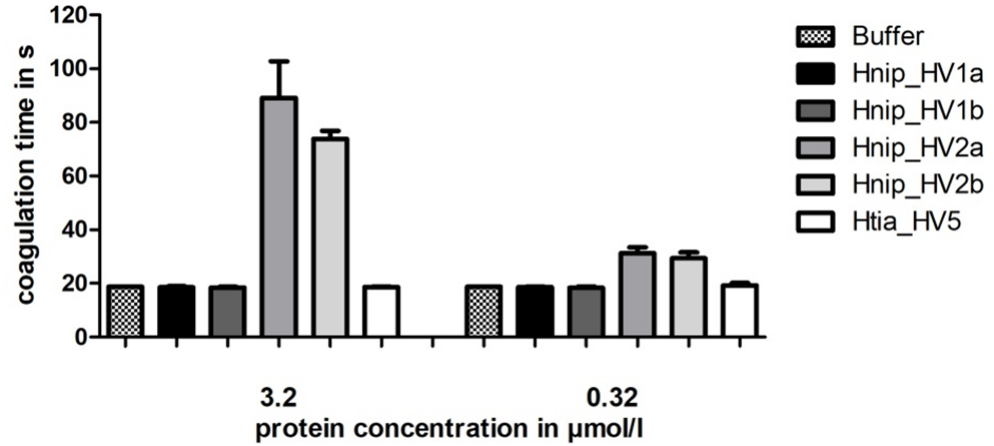
Standard blood coagulation assays of putative hirudins of *H. nipponia* (Hnip_HV1 and Hnip_HV2) and *H. tianjinensis* (Htia_HV5) using the thrombin time test (TT). All test compounds were used at final concentrations of 3.2 or 0.32 µmol/l, respectively. Results are the mean of three independent measurements, bars indicate standard deviation (SD)

Some of the putative hirudins/HLFs of both *H. nipponia* and *H. tianjinensis* have already been functionally characterized in previous investigations, namely Htia_HV1 and Htia_HV2 by Müller et al. (2022) and Hnip_HV1-3 and Htia_HV1-3 by Yin et al. (2025) (see Table 1 for details). We performed additional coagulation measurements using the thrombin time test (TTT) for a selection of putative hirudins/HLFs including Hnip_HV1, Hnip_HV2 and the newly identified Htia_HV5. The TTT specifically addresses the fibrinogen-converting activity of thrombin. To investigate the putative effects of alterations within the N-terminal regions we constructed variants of both Hnip_HV1 and Hnip_HV2. For Hnip_HV1 the signal peptide sequence predictions slightly varied between the different tools and we hence expressed variants that started either with E20 (Hnip_HV1a) or H21 (Hnip_HV1b) (see Fig. 1). For Hnip_HV2 the basic residue K24 (or K4 in the mature protein; Hnip_HV2a) was changed to a threonine residue (Hnip_HV2b) as it is present in the archetype hirudins of *H. medicinalis*. All factors were successfully expressed, purified and processed. Whereas both variants of Hnip_HV2 displayed clear thrombin-inhibitory potencies and can hence be denominated as hirudins, neither the variants Hnip_HV1a and Hnip_HV1b nor Htia_HV5 displayed any such potencies at the tested concentrations (see Fig. 3). However, the thrombin-inhibitory potencies of both variants of Hnip_HV2 were markedly lower compared to the archetype hirudin variant HV1 (hirudin-VV) of *H. medicinalis* that regularly reaches the maximal coagulation time of 300 s when tested at a concentration of 3,2 µmol/l using the TTT (see Müller et al. 2020 or Wang et al. 2025).

### Functional characterization II: platelet aggregation

**Fig. 4:**
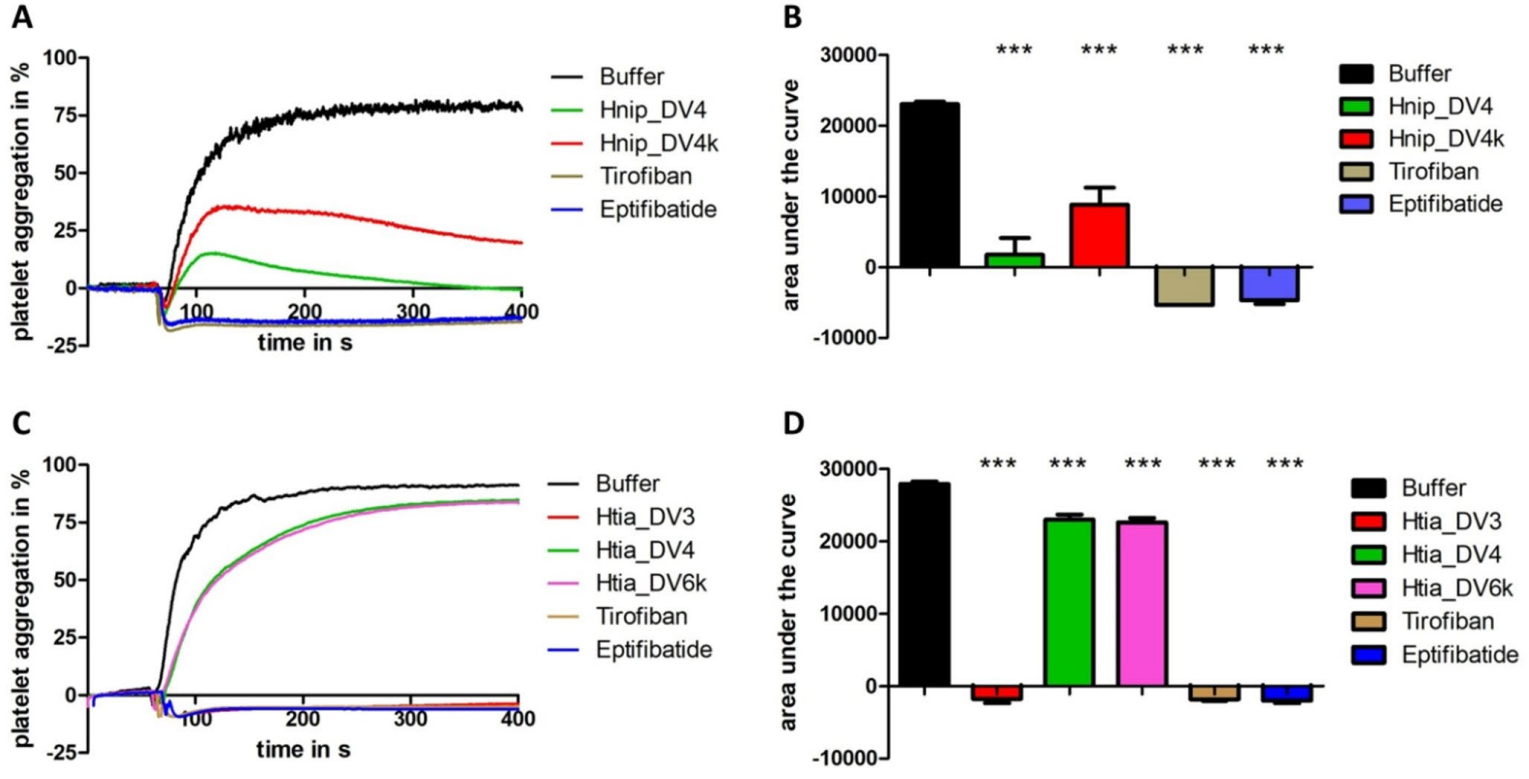
Standard platelet aggregation assays of putative monomeric and multimeric decorsin variants of *H. nipponia* (A + B) and *H. tianjinensis* (C + D). A and C display the aggregation curves, B and D the derived AUC values. Tirofiban and eptifibatide were used as positive control substances for the complete inhibition of aggregation; buffer was used as a negative control. All test compounds and the control substances were used at a final concentration of 3.2 µmol/l. Platelet aggregation was induced by the addition of ADP to a final concentration of 5 µmol/l. Results are the mean of two to four independent measurements, bars indicate SD. *** indicates a significant difference to the buffer control with p-values ≤ 0.001 applying unpaired two-tailed t-tests

To test them for potential platelet aggregation-inhibitory potencies we expressed the putative monomeric decorsins Htia_DV3 and Htia_DV4. Hnip_DV1-3 and Htia_DV1-3 share high degrees of sequence similarity (see Fig. 2) and we assumed that data on Htia_DV3 are representative for all respective factors. In contrast, Htia_DV4 and Htia_DV5 are apparently not present in *H. nipponia* and are clearly different from Htia_DV3. In addition we expressed both the putative multimeric decorsin Hnip_DV4 and a monomeric variant thereof (Hnip_DV4k) that only contains the first repeat of the globular domain with the RGD motif. However, we failed to express and purify the multimeric Htia_DV6, but we succeeded in purifying a monomeric variant thereof (Htia_DV6k) that contains the first repeat of the globular domain of Htia_DV6 with the YGD motif. The snake venom-derived platelet aggregation-inhibitors tirofiban and eptifibatide were used as positive controls to completely block platelet aggregation. As can be seen in Fig. 4A, both the putative multimeric decorsin Hnip_DV4 and its monomeric variant Hnip_DV4k strongly decreased platelet aggregation compared to the solvent control samples, yet to a lesser extent compared to the positive controls at the tested concentrations. Strikingly, the putative monomeric decorsin Htia_DV3 was as effective in inhibition of platelet aggregation as the positive controls tirofiban and eptifibatide at the tested concentrations, whereas the inhibitory effect of Htia_DV4 was considerably weaker and in about the same range as the inhibitory effect of Htia_DV6k (Fig. 4C). To conduct statistical analyses, the area under the curve (AUC) values for all samples were determined and pairwise compared to the values of the respective buffer controls applying unpaired two-tailed t-tests. We determined significant differences in the AUC values for all test samples, compared to the respective buffer controls with p-values ≤ 0.001 (Fig. 4B + D).

### Phylogenetic analyses

**Fig. 5:**
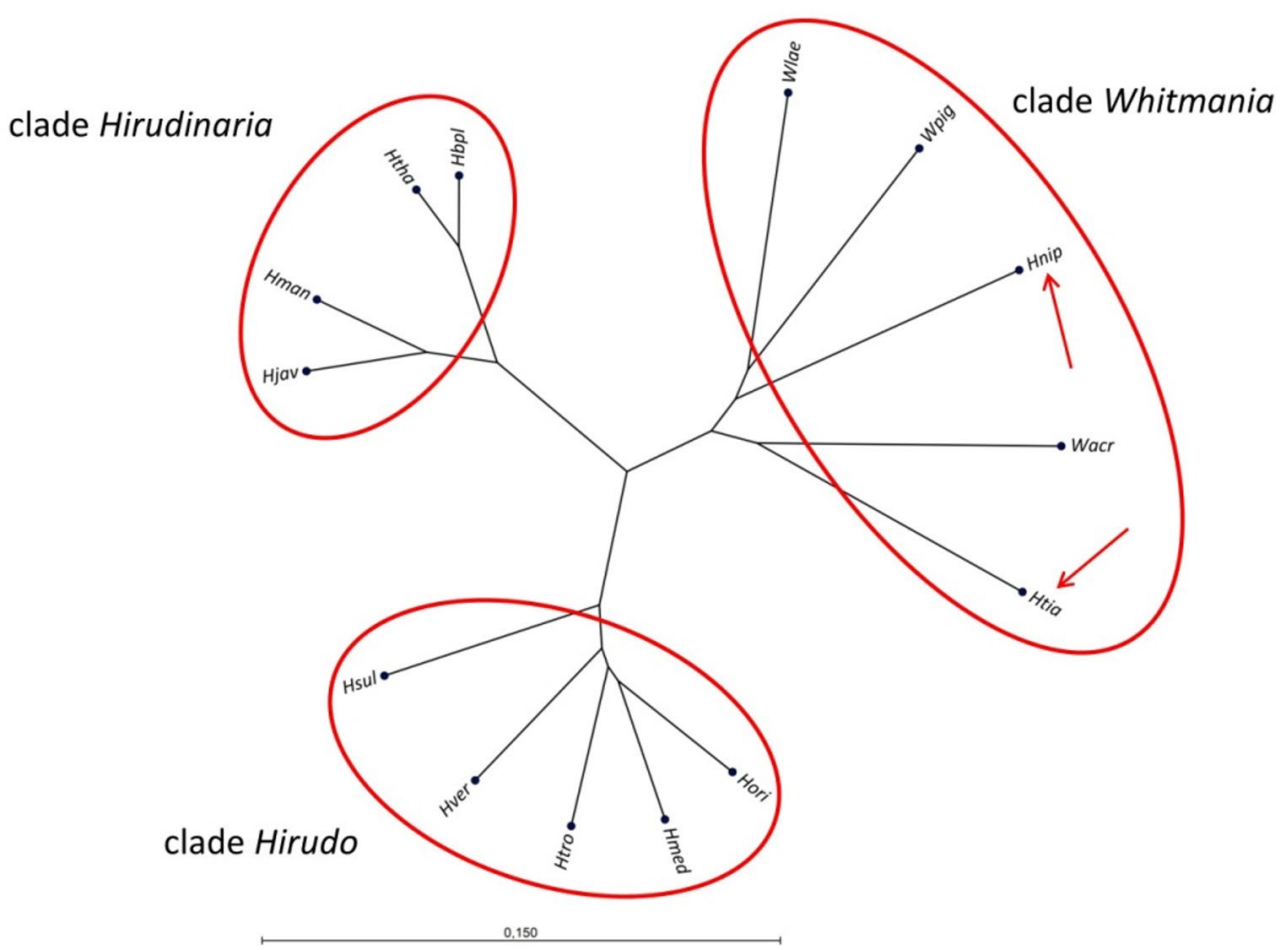
Unrooted phylogenetic tree based on partial *coi* sequences generated using the UPGMA algorithm. Red circles encompass the predicted clades “*Hirudo*”, “*Hirudinaria*” and “*Whitmania*”. The red arrows indicate the position of *H. nipponia* and *H. tianjinensis* within the predicted clade “Whitmania”. *Hmed* = *H. medicinalis*; *Hver* = *H. verbana*; *Hori* = *H. orientalis*; *Htro* = *H. troctina*; *Hsul* = *H. sulukii*; *Hman* = *H. manillensis*; *Hbpl* = *H. bpling*; *Hjav* = *H. javanica*; *Htha* = *H. thailandica*; *Wpig* = *W. pigra*: *Wacr* = *W. acranulata*; *Wlae* = *W. laevis*; *Hnip* = *H. nipponia*; *Htia* = *H. tianjinensis*. The scale bar indicates substitutions per site

Historically, *H. nipponia* is classified in the same genus *Hirudo* as the European medicinal leeches, but recent investigations based on widely used molecular markers seriously questioned the correctness of this classification. Instead, *H. nipponia* was more closely related to a member of the genus *Whitmania* (Phillips and Siddall 2009). We collected data and performed phylogenetic analyses using both a mitochondrial and a genomic marker to verify which of the two alternatives receives more support. A 624 bp fragment of the mitochondrial cytochrome oxidase subunit one gene (*coi*) and a fragment of the genomic internal transcribed spacers (ITS1 and ITS2) including the 5.8 S rRNA gene (Trontelj and Utevsky 2012) were used as molecular markers. The following species were included into our analyses based on the *coi* sequences: *H. medicinalis*, *Hirudo verbana* Carena, 1820, *Hirudo orientalis* Utevsky & Trontelj 2005, *Hirudo troctina* Johnson, 1816 and *Hirudo sulukii* Saglam, Saunders, Lang & Shain, 2016 as representatives of European members of the genus Hirudo; *H. manillensis*, *H. bpling*, *Hirudinaria thailandica* Jeratthitikul & Panha, 2020 and *Hirudinaria javanica* Wahlberg, 1856 as representatives of the Asian genus *Hirudinaria*; *W. pigra*, *W. acranulata* and *Whitmania laevis* Baird, 1869 as representatives of the genus Whitmania and eventually *H. nipponia* and *H. tianjinensis*. For the analysis based on the ITS1-5.8srRNA-ITS2 region respective data for *H. bpling* and *H. thailandica* were missing. The GenBank accession numbers of all sequence data are provided in Supplementary Information Files S7 (*coi* sequences) and S8 (ITS1-5.8srRNA-ITS2 region sequences), the MSAs of both analyses including a list of the pairwise degrees of sequence identity are provided in Supplementary Information Files S9 (*coi* sequences) and S10 (ITS1-5.8srRNA-ITS2 region sequences). Figure 5 displays the results of the analyses based on the *coi* sequences as an unrooted phylogenetic tree, the results for the analyses based on the ITS1-5.8srRNA-ITS2 region are provided in Supplementary Information File Figure S1. Concordantly in both analyses three distinct clades were predicted: the clade “Hirudo” that comprises the European members of the genus *Hirudo*, the clade “Hirudinaria” that comprises the members of the genus *Hirudinaria* and a third clade “Whitmania” that comprises the members of the genus *Whitmania* as well as *H. nipponia* and *H. tianjinensis*. It is evident that both *H. nipponia* and *H. tianjinensis* do not form a clade together with the European representatives of the genus *Hirudo*.

## Discussion

The hematophagous lifestyle of medicinal leeches critically depends on the adequate and timely availability of anticoagulants and other bioactive factors in their saliva (Lemke et al. 2016). To secure an undisturbed blood meal the saliva of medicinal leeches comprises factors that address both the primary (platelet aggregation) and the secondary (blood coagulation) hemostasis. Decorsins and hirudins are probably the most prominent representatives of respective proteins, and both factors belong to the hirudin superfamily. Many, but not all medicinal leech species analyzed so far accommodate at least one, but frequently several genes for both types of anticoagulants in their genomes (Min et al. 2010; Pfordt et al. 2022; Müller et al. 2024). The Asian medicinal leeches *H. nipponia* and *H. tianjinensis* each have three putative hirudin and three (Hnip) or five (Htia) putative decorsin genes, respectively (Zhao et al. 2024). However, functional characterizations to verify their actual biological activities were still pending. We expressed two putative decorsins of *H. tianjinensis*, namely Htia_DV3 and Htia_DV4, representing two distinct isoforms. The DV3-isoform is present in both *H. nipponia* and *H. tianjinensis*, whereas the DV4-type is present in *H. tianjinensis* only (see Fig. 1). Both factors revealed platelet aggregation-inhibitory potencies. The inhibitory effect of Htia_DV3, however, was more pronounced than that of Htia_DV4 (see Fig. 4 right). The platelet aggregation assays were performed with human platelets. Because mammalian platelets considerably differ from fish, amphibian or avian platelets (Menter et al. 2022; Russell and Heatley 2022; Ortiz and Esteban 2024), it might well be that the DV4-type decorsins reveal stronger aggregation-inhibitory effects on platelets of non-mammalian origin, but this remains to be proven. In addition to the monomeric decorsins Hnip_DV1-3 and Htia_DV1-5, both leech species also have genes that encode putative multimeric decorsins, namely Hnip_DV4 and Htia_DV6 and 7. A multimeric decorsin has only recently been described and functionally characterized for the first time (Müller et al. 2024). We have successfully expressed and purified the multimeric Hnip_DV4 and a monomeric derivative thereof, Hnip_DV4k, and both factors displayed potent platelet-inhibitory effects (Fig. 4 left). The molecule of Hnip_DV4 contains 24 cysteine residues that require the correct formation of 12 disulfide bonds. The recombinant expression of cysteine-rich proteins is a challenging task (Lukas et al. 2022; Wang et al. 2025) and we did not apply any highly sophisticated methods to determine and analyze the structures of all recombinantly expressed factors. However, the comparably high potency of Hnip_DV4 to inhibit platelet aggregation indicates that a substantial part of the recombinant proteins was correctly folded, and that the factors significantly contributes to the anticoagulatory repertoire of *H. nipponia*. In contrast, *H. tianjinensis* apparently lacks a respective active multimeric decorsin. Htia_DV7 does not contain the canonical RGD motif (see Supplementary Information File S6 for details). The gene for Htia_DV6 in the reference genome of *H. tianjinensis* is incomplete, but a complete cDNA of Htia_DV6 could be assembled from salivary gland transcriptome data. The monomeric variant Htia_DV6k displays a weak, yet detectable platelet aggregation-inhibitory effect (see Fig. 4 right), but the factor was less effective compared to its direct counterpart Hnip_DV4k. Interestingly, Htia_DV6k comprises an YGD motif instead of the RGD motif in Hnip_DV4k. The presence of an intact RGD motif is crucial for cell binding and internalization of adenoviruses, yet an alternative YGD motif restored the pathogenicity of a human adenovirus with a deletion of the canonical RGD loop (Robinson et al. 2013). An YGD motif may hence functionally replace the RGD motif, but whether or not this is the case also for decorsins remains to be proven.

The thrombin-inhibitory potencies of putative hirudins critically depend on the properties of the N-terminal five amino acid residues, length and charge of the C-terminal tail, the characteristics of the central globular domain and an overall acidic pI value (Müller et al. 2020, 2021). Of special importance in that context is the residue tyrosine (Tyr, Y) (or phenylalanine, Phe, F) at position 3 of the mature protein (Lazar et al. 1991; Huang et al. 2014). The putative hirudins Hnip_HV2, Htia_HV1 and Htia_HV2 concordantly contain either Tyr or Phe residues at the respective positions (see Fig. 1), and all three factors exhibit thrombin-inhibitory potencies. It is interesting to note that the pI value of Htia_HV2 is remarkably high (6.72) compared to both Htia_HV1 (4.84) and Htia_HV2 (5.22) (Table 2). The archetype hirudins of *H. medicinalis* possess pI values of about 4.1 (Müller et al. 2016). In contrast, the factors Hnip_HV3, Htia_HV3 and Htia_HV5 do not contain the crucial structural and biochemical features of hirudins and they accordingly do not inhibit thrombin activity (Müller et al. 2022; Yin et al. 2025; this study). Hnip_HV3, Htia_HV3 and Htia_HV5 can hence be considered as HLFs, and it is reasonable to assume that a similar classification also applies to Hnip_HV4-HV7 and Htia_HV4, HV6 and HV7. The classification of Hnip_HV1, however, remains obscure. Yin et al. (2025) measured an extremely high anti-thrombin activity of Hnip_HV1, but in our hands the factor did not inhibit the activity of thrombin in a TTT assay (Fig. 3). The structural (length and absence of Tyr or Phe residues within the N-terminus and a very short C-terminal tail, see Fig. 1) and biochemical (basic pI value of 8.87) features of Hnip_HV1 clearly point to a classification as a HLF, but further investigations are needed to clarify the discrepancies between the outcomes of the two different functional characterizations. As a first step, binding studies may indicate whether or not Hnip_HV1 and thrombin directly interact, and if this is the case, co-crystallization of both partners is mandatory.

Both *H. nipponia* and *H. tianjinensis* have several genes that encode additional monomeric and multimeric hirudins or HLFs. Strikingly, in both leeches the respective genes are located on different chromosomes (on chromosome 2) compared to the decorsins and hirudins (on chromosome 5 in *H. nipponia* and on chromosome 6 in *H. tianjinenesis*, see Table 2). Our analyses revealed that the genes of all monomeric factors and almost all multimeric hirudins/HLFs and decorsins are expressed in the salivary glands of *H. nipponia* and *H. tianjinensis*, respectively (see Table S2 for details). This observation indicates that the multimeric hirudins/HLFs and decorsins may play substantial roles in the blood feeding process. The biological function of the corresponding multimeric proteins, however, remains largely elusive and is methodically difficult to address. The strong platelet aggregation inhibitory effect of Hnip_DV4 indicates that multimeric factors may have equal or even higher inhibitory potencies compared to the archetype monomeric factors. Furthermore, multimeric factors may exhibit different binding properties, an increased proteolytic stability or enhanced specificity and target selectivity. In addition to altered biochemical properties of multimeric factors, the genetic organization of genes that encode multimeric hirudins/HLFs and decorsins offers the inherent capacity to express a multitude of additional factors based on the various mechanisms of mRNA processing. In a recent publication we have calculated that the Hman_DV3 gene of *H. manillensis* (a hexameric decorsin) comprises the potential to express almost 200 different factors, ranging from monomers to the hexameric DV3 protein, solely based on the mechanism of exon skipping (Tolksdorf et al. 2025). If intron retain is included as a second mechanism of mRNA processing, the number even doubles. The presence and expression of several multimeric hirudin/HLF genes in *H. nipponia* and *H. tianjinensis* may hence tremendously broadens the biochemical repertoire of both leech species. Intriguingly, so far there is no evidence that genes that encode multimeric hirudins/HLFs are present in the genomes of the European members of the genus *Hirudo*, without any obvious limitations in their blood-feeding competence. Future investigations will hopefully identify the traits that may explain the benefits of multimeric hirudins/HLFs. Interestingly, Htia_mHV14 of *H. tianjinensis* contains a RGD motif that is located within the last repeat of the central globular domain between the cysteine residues C5 and C6, but the spacing between the cysteine residues (34 amino acid residues, see Supplementary Information File S2) is much larger compared to the archetype decorsins (10 amino acid residues). Nevertheless Htia_mHV14 may serve as a “substrate” for evolutionary processes that may eventually result in the generation of a new decorsin variant.

To resolve the phylogeny of leeches is a challenging task, and several attempts have been made over the last decades to elucidate the correct relationships between the different taxa (Siddall and Burreson 1997; Apakupakul et al. 1999; Phillips and Siddall 2009; Siddall et al. 2011). Of particular interest in that context is the genus *Hirudo* that traditionally comprised the European medicinal leeches and the Asian medicinal leech *Hirudo nipponia* (Trontelj and Utevsky 2005). However, a previous study indicated that *H. nipponia* is more closely related to *W. laevis* compared to the European medicinal leeches (Phillips and Siddall 2009). The results of our investigations based on two molecular markers (a partial mitochondrial *coi* sequence and a partial sequence of the genomic ITS1-5.8srRNA-ITS2 region) are entirely in line with this observation. The two Asian members of the genus *Hirudo*, *H. nipponia* and *H. tianjinensis*, form a clade together with the members of the genus *Whitmania* that is different from the clades that are formed either by the European leeches (clade *Hirudo*) or by members of the genus *Hirudinaria* (clade *Hirudinaria*) (Fig. 5, Supplementary Information File Figure S1). Support for a relocation of *H. nipponia* and *H. tianjinensis* to the genus *Whitmania* comes from the observation that all members of the genus *Whitmania* analyzed so far and both *H. nipponia* and *H. tianjinensis* concordantly comprise 11 chromosomes (Liu et al. 2024; Zhao et al. 2024, 2025), whereas the European medicinal leeches comprise 12 (*H. orientalis*), 13 (*H. verbana*) or 14 (*H. medicinalis*) chromosomes, respectively (Utevsky et al. 2009). The need for further investigations to eventually clarify the correct phylogenetic relationships among medicinal leeches in particular and the broad variety of leeches in general, however, is evident.

## Conclusions

Our study revealed a remarkable diversity of putative bioactive factors that belong to the hirudin superfamily, namely hirudins, decorsins and HLFs, in the two species of Asian medicinal leeches *H. nipponia* and H. *tianjinensis*. Of particular interest is the presence of a yet unidentified multimeric decorsin that exhibited a strong inhibitory effect on platelet aggregation. Multimeric members of the hirudin superfamily may hence well be part of the broad repertoire of anticoagulants in hematophagous leeches, however, the exact biological functions of the majority of monomeric and multimeric HLFs remains to be determined. Additional efforts are also mandatory to further clarify the phylogenetic relationships within and among the different genera of medicinal leeches.

## Supplementary Information

Below is the link to the electronic supplementary material.

Supplementary Material 1 (ZIP 660 KB)

## Data Availability

Original sequence data are deposited in GenBank, specific entry numbers and information on gene locations are provided either within the manuscript or as Supplementary Information Files. Images that illustrate the expression, purification and processing of all recombinant factors are available on request from the corresponding author.
