# Supplementary material for: The great diversity: monomeric and oligomeric hirudins, hirudin-like factors and decorsins in the Asian medicinal leeches Hirudo nipponia and Hirudo tianjinensis
Source: Parasitol Res. 2026 Feb 7;125(1):18. doi: 10.1007/s00436-026-08634-0 (PMC12882960; doi:10.1007/s00436-026-08634-0)
Supplement: Supplementary file 1 — Supplementary Material 1 (ZIP 660 KB) [file 436_2026_8634_MOESM1_ESM.zip › S10_ MSA of ITS sequences.rtf]

Supplementary Information File S10: MSA of ITS1-5.8srRNA-ITS2 region sequences

                *        20         *        40         *        60         *        80         *      
Hmed : GGAAGTAAAAGTCGTAACAAGGTTTCCGTAGGTGAACCTGCGGAAGGATCATTATCGAAAGCCGTTG-ATCGTTCGTTCGTTCGTTCAAT :  89
Hver : GGAAGTAAAAGTCGTAACAAGGTTTCCGTAGGTGAACCTGCGGAAGGATCATTATCGAAAGCCGTTG-ATCGTTCGTTCGT----TCAAT :  85
Hori : GGAAGTAAAAGTCGTAACAAGGTTTCCGTAGGTGAACCTGCGGAAGGATCATTATCGAAAGCCGTTG-ATCGTTCGCTC--------AAT :  81
Htro : GGAAGTAAAAGTCGTAACAAGGTTTCCGTAGGTGAACCTGCGGAAGGATCATTATCGAAAGCCGTTG-ATCGTTCGCTC--------GAT :  81
Hnip : GGAAGTAAAAGTCGTAACAAGGTTTCCGTAGGTGAACCTGCGGAAGGATCATTATCGAAAGCCGTTG-GTCCAGTGTTCGCGCTGATCAT :  89
Htia : GGAAGTAAAAGTCGTAACAAGGTTTCCGTAGGTGAACCTGCGGAAGGATCATTATCGAAAGCCGTTG-GTCCAGTGTTCGCGCTGATCAT :  89
Wpig : GGAAGTAAAAGTCGTAACAAGGTTTCCGTAGGTGAACCTGCGGAAGGATCATTATCGAAAGCCGTTG-GTCCAGTGTTCGCGCTGATCAT :  89
Wlae : GGAAGTAAAAGTCGTAACAAGGTTTCCGTAGGTGAACCTGCGGAAGGATCATTATCGAAAGCCGCTG-GTCCAGTGTTCGCGCTGATCAT :  89
Wacr : GGAAGTAAAAGTCGTAACAAGGTTTCCGTAGGTGAACCTGCGGAAGGATCATTATCGAAAGCCGTTG-GTCCAGTGCTCGCGCTGATCAT :  89
Hman : GGAAGTAAAAGTCGTAACAAGGTTTCCGTAGGTGAACCTGCGGAAGGATCATTATCGAAAGCCGTCGTAGCGATCTCTCGCCTCGTCGAG :  90
Hjav : GGAAGTAAAAGTCGTAACAAGGTTTCCGTAGGTGAACCTGCGGAAGGATCATTATCGAAAGCCGTCGGAGCGATCTCTCGCCTCGTCGAG :  90

                                                                                                       
              100         *       120         *       140         *       160         *       180      
Hmed : CAATCGCGCTTGATTTGGTTTGTCACCGATTTCGTGCGTCCGCGGTCGCTCCTCGCTTCGTGCGAGTCGCCCGCGGTCGACTGCAAGTGC : 179
Hver : CAATCGCGCTTGATTTGGTTTGTCACCGATTTCGTGCGTCCGCGGTCGCTCCTCGCTTCGTGCGAGTCGCCCGCGGTCGACTGCAAGTGC : 175
Hori : CAATCGCGCTTCATTTGGTTTGTCACCGATTTCGTGCGTCCGCGGTCGCTCCTCGCTTCGTGCGAGTCGCCCGCGGTCGACTGCAAGTGC : 171
Htro : CAATCGCGCTTCATTTGGTTTGTCACCGATTTCGTGCGTCCGCGGTCGCTCCTCGCTTCGTGCGAGTCGCCCGCGGTCGACTGCAAGTGC : 171
Hnip : CGTGCAACGAACAA-TCGTTTGTCACCGATTTCGTGCGTCCTCGGTCGCTCCTCGCTTCGTGCGAGTCGCCCGTGGTCGACCGCAAGTGC : 178
Htia : CGTGCAACGAACAA-TCGTTTGTCACCGATTTCGTGCGTCCTCGGTCGCTCCTCGCTTCGTGCGAGTCGCCCGTGGTCGACCGCAAGTGC : 178
Wpig : CGTGCAACGAACAA-TCGTTTGTCACCGATTTCGTGCGTCCTCGGTCGCTCCTCGCTTCGTGCGAGTCGCCCGTGGTCGACCGCAAGTGC : 178
Wlae : CGTGCAACGAACAA-TCGTTTGTCACCGATTTCGTGCGTCCTCGGTCGCTCCTCGCTTCGTGCGAATCGCCCGTGGTCGACCGCAAGTGC : 178
Wacr : CGTGCAACGAACAAATCGTTTGTCACCGATTTCGTGCGTCCTCGGTCGCTCCTCGCTTCGTGCGAGTCGCCCGCGGTCGACTGCAAGTGC : 179
Hman : CGTTTCAATCGAAA---TGTCGTTACCGATTTCGAGCGTCCTCGGTCGCTCCTCGCCTAGTGCGAGTCGCCCGTGATCGACGGCAAGTGC : 177
Hjav : CGTTTCAATCGAAA---TGTCGTTACCGATTTCGTGCGTCCTCGGTCGCTCCTCGCCTAGTGCGAGTCGCCCGGGGTCGACGGCAAGTGC : 177

                                                                                                       
                *       200         *       220         *       240         *       260         *      
Hmed : CGACGTCGAAGTCTCCTGGCGCGCGTCCTATGGCG--GTCGTCGTTTTGTCT-CCTAGAGGTACTCCACGCCTATGGTACCTCGTAAGAC : 266
Hver : CGACGTCGAAGTCTCCTGGCGCGAGTCCTATGGCGCGGTCGTCGTTTTGTCT-CCTAGAGGTACTCCACGCCTATGGTACCTCGTAAGAC : 264
Hori : CGACGTCGAAGTCTCCTGGCGCGCGTCCTATGGCG--GTCGTCGTTTTGTCT-CCTAGAGGTACTCCACGCCTATGGTACCTCGTAAGAC : 258
Htro : CGACGTCGAAGTCTCCCGGCGCG--TCCGATCGCG--GTCGTCGTTTTGTCT-CCTAGAGGTACTCCACGCCTATGGTACCTCGTCAGAC : 256
Hnip : CGACTTCGAAGTCTCCCGGT-------CGATGATGATGATGATGACGATCGT--CTGGAGGTACTCCACGCCTATGGTACCTCTCTTGTC : 259
Htia : CGACTTCGAAGTCTCCCGGC-------CGATGATGATGATGATGACGATCGTGTCTGGAGGTACTCCACGCCTATGGTACCTCTCTTGTC : 261
Wpig : CGACTTCGAAGTCTCCCGGT-------CGATGACGACGACGACGAC---TGT--CCGGAGGTACTCCACGCCTATGGTACCTCTCTTGTC : 256
Wlae : CGACTTCGAAGTCTCCCGGT-------CGATGACGACGAAGACGAC---CGT--CCGGAGGTACTCCACGCCTATGGTACCTCTCTTGTC : 256
Wacr : CGACTTCGAAGTCTCCCGGT-------CGATGATGATGATGACGAT---AGT--CTGGAGGTGCTCCACGCCTATGGCACCTCTCTTGTC : 257
Hman : CGACGTCGACGTCTCACGTGGTCGAATCGAGCGCATCCCGGATGTC---T----CTAGAGGGCTTTCGCGGCTCTCGTATCC----GGGC : 256
Hjav : CGACGTCGACGTCTCACGTGGTCGAATCGAGCGCATCCCGGATGTC---TGTCTCTAGAGGGCTTTCGCGGCTCTCGTATCC----GGGC : 260

                                                                                                       
              280         *       300         *       320         *       340         *       360      
Hmed : GAC-----CTATCG---TCG----GACGATCGTCGCTCGT-CGTCGTGCATGGCTTAAAACATTTTCGATACCCCATTACAATAAAGTCG : 343
Hver : GAC-----CTATCG---TCG----GACGATCGTCGCTCGT-CGTCGTGCATGGCTTAAAACATTTTCGATACCCMATTACAATAAAGTCG : 341
Hori : GAC-----CTATCG---TCG----GACCATCGTCGCTCGT-CGTCGTGCATGGCTTAAAACATTTTCGATACCCAATTACAATAAAGTCG : 335
Htro : GAC-----CTATCG---TCG----GACGATCGTCGCTCGT-CGTCGTGCATGGCTTAAAACATTTTCGATACCCCATTACAATAAAGTCG : 333
Hnip : TCC-GTGACTGTCGTCCTCGTCGCTGTCGTCGCCGCTCGT-CGTCGTGCATCGCT--AAACATTTTCGATACCGTTGT-CTATAAAGTCG : 344
Htia : TCC-GTGACCGTCGTCTTCGTCGTCGTCGTCGCCGCTCGT-CGTCGTGCATCGCT--TAACATTTTCGATACCGTTGT-CTATAAAGTCG : 346
Wpig : TCTTGTGACTGTCG---TCGTTGTCGTCGTCGCCGCTCGTGTGTCGTGCATCGCT--AAACATTTTCGATACCGTTAT-CTATAAAGTCG : 340
Wlae : TCTTGTGACTGTCG---TCGTCGTCGTCGTCGCCGCTCGTGCGTCGTGCATCGCT--AAACATTTTCGATACCCTTAT-CTATAAAGTCG : 340
Wacr : TCT-GTGACTTTCG---TCGTCGTCGTCGTCTCCGCTCGT-CGTCGTGCATCGCT--AAACATTT-CGATACCGTAGT-CTATAATGTCG : 338
Hman : TTC------TCTCGTGCTCTT-----T-TAGGCCGGTCGT-CGTCGTGCATCGTCAAAACATTTTTCGATACCCCATGTCTATAAAGTCG : 333
Hjav : TTC------TCTCGTGCTCTT-----TACCGGCCGGTCGT-CGTCGTGCATCGTCAAAACATTTTTCGATACCCCATGTCTATAAAGTCG : 338

                                                                                                       
                *       380         *       400         *       420         *       440         *      
Hmed : CCGGAAGCCGTAATTGGCTTTCTGACGCGGTCGTCCGAGGAGGACGTCCAAAACGAGAGAACACTCTAAGCGGTGGATCACTCGGCTCGT : 433
Hver : CCGGAAGCCGTAATTGGCTTTCTGACGCGGTCGTCCGAGGAGGACGTCCAAAACGAGAGAACACTCTAAGCGGTGGATCACTCGGCTCGT : 431
Hori : CCGGAAGCCGTAATTGGCTTTCTGACGCGGTCGTCCGAGGAGGACGTCCAAAACGAGAGAACACTCTAAGCGGTGGATCACTCGGCTCGT : 425
Htro : CCGGAAGCCGTAATTGGCTTTCTGACGCGGTCGTCCGAGGAGGACGTCCAAAACGAGAGAACACTCTAAGCGGTGGATCACTCGGCTCGT : 423
Hnip : CCGGGAGCCGTAATTGGCTTTCTGACGCGGTCGTCTGAGGAGGACGTCCAAAACGAGAGAACACTCTAAGCGGTGGATCACTCGGCTCGT : 434
Htia : CCGGGAGCCGTAATTGGCTTTCTGACGCGGTCGTCTGAGGAGGACGTCCAAAACGAGAGAACACTCTAAGCGGTGGATCACTCGGCTCGT : 436
Wpig : CCGGGAGCCGTAATTGGCTTTCTGACGCGGTCGTCTGAGGAGGACGTCCAAAACGAGAGAACACTCTAAGCGGTGGATCACTCGGCTCGT : 430
Wlae : CCGGGAGCCGTAATTGGCTTTCTGACGCGGTCGTCTGAGGAGGACGTCCAAAACGAGAGAACACTCTAAGCGGTGGATCACTCGGCTCGT : 430
Wacr : CCGGGAGCCGTAATTGGCTTTCTGACGCGGTCGTCCGAGGAGGACGTCCAAAACTAGAGAACACTCTAAGCGGTGGATCACTAGGCTCGT : 428
Hman : CCGGGAGCCGTAACTGGCTTTCTGACGCGGTCGTCCGAGGAGGACGTCCAAAACGAGAGAACACTCTAAGCGGTGGATCACTCGGCTCGT : 423
Hjav : CCGGGAGCCGTAACTGGCTTTCTGACGCGGTCGTCCGAGGAGGACGTCCAAAACGAGAGAACACTCTAAGCGGTGGATCACTCGGCTCGT : 428

                                                                                                       
              460         *       480         *       500         *       520         *       540      
Hmed : GGGTCGATGAAGAGCGCAGCCAAATGCGTTAATTAATGTGAATTGCAGGACACATTGAACATCGACATCTTGAACGCATATTGCGGCCCC : 523
Hver : GGGTCGATGAAGAGCGCAGCCAAATGCGTTAATTAATGTGAATTGCAGGACACATTGAACATCGACATCTTGAACGCATATTGCGGCCCC : 521
Hori : GGGTCGATGAAGAGCGCAGCCAAATGCGTTAATTAATGTGAATTGCAGGACACATTGAACATCGACATCTTGAACGCATATTGCGGCCCC : 515
Htro : GGGTCGATGAAGAGCGCAGCCAAATGCGTTAATTAATGTGAATTGCAGGACACATTGAACATCGACATCTTGAACGCATATTGCGGCCCC : 513
Hnip : GGGTCGATGAAGAGCGCAGCTAAATGCGTTAATTAATGTGAATTGCAGGACACATTGAACATCGACATCTTGAACGCATATTGCGGCCCC : 524
Htia : GGGTCGATGAAGAGCGCAGCTAAATGCGTTAATTAATGTGAATTGCAGGACACATTGAACATCGACATCTTGAACGCATATTGCGGCCCC : 526
Wpig : GGGTCGATGAAGAGCGCAGCTAAATGCGTTAATTAATGTGAATTGCAGGACACATTGAACATCGACATCTTGAACGCATATTGCGGCCCC : 520
Wlae : GGGTCGATGAAGAGCGCAGCTAAATGCGTTAATTAATGTGAATTGCAGGACACATTGAACATCGACATCTTGAACGCATATTGCGGCCCC : 520
Wacr : GGGTCGATGAAGAGCGCAGCCAAATGCGTTAATTAATGTGAATTGCAGGACACATTGAACATCGACATCTTGAACGCATATAGCGGCCCC : 518
Hman : GGGTCGATGAAGAGCGCAGCCAAATGCGTTAATTAATGTGAATTGCAGGACACATTGAACATCGACATCTTGAACGCATATTGCGGCCCC : 513
Hjav : GGGTCGATGAAGAGCGCAGCCAAATGCGTTAATTAATGTGAATTGCAGGACACATTGAACATCGACATCTTGAACGCATATTGCGGCCCC : 518

                *       560         *       580         *       600         *       620         *      
Hmed : GGGTCCTCCCGGAGCCAGGCCTGTCTCAGGGTCGGTTTGAGTACAATCGCGGGTTTTCGCCTACGGCCCGCGCACTGGGTTTTCGCGGCA : 613
Hver : GGGTCCTCCCGGAGCCAGGCCTGTCTCAGGGTCGGTTTGAGTACAATCGCGGGTTTTCGCCTACGGCCCGCGCACTGGGTTTTCGCGGCA : 611
Hori : GGGTCCTCCCGGAGCCAGGCCTGTCTCAGGGTCGGTTTGAGTACAATCGCGGGTTTTCGCCTACGGCCCGCGCACTGGGTTTTCGCGGCA : 605
Htro : GGGTCCTCCCGGAGCCAGGCCTGTCTCAGGGTCGGTTTGAGTACAATCGCGGGTTTTCGCCTACGGCCCGCGCACTGGGTTTTCGCGGCA : 603
Hnip : GGGTCCTCCCGGAGCCAGGCCTGTCTCAGGGTCGGTTAGAGTACAATCGCGGGTTTTCGCCTATGGCTCGCGCAATGGGTTTTCGCGGCA : 614
Htia : GGGTCCTCCCGGAGCCAGGCCTGTCTCAGGGTCGGTTAGAGTACAATCGCGGGTTTTCGCCTATGGCTCGCGCAATGGGTTTTCGCGGCA : 616
Wpig : GGGTCCTCCCGGAGCCAGGCCTGTCTCAGGGTCGGTTAGAGTACAATCGCGGGTTTTCGCCTACGGCCCGCGCAATGGGTTTTCGCGGCA : 610
Wlae : GGGTCCTCCCGGAGCCAGGCCTGTCTCAGGGTCGGTTAGAGTACAATCGCGGGTTTTCGCCTACGGCCCGCGCAATGGGTTTTCGCGGCA : 610
Wacr : GGGTCCTCCCGGAGCCAGGCCTGTCTCAGGGTCGGTTAGAGTACAATCGCGGGTTTTCGCCTACGGCCCGCGCAATGGGTTTTCGCGGCA : 608
Hman : GGGTCCCCCCGGAGCCAGGCCTGTCTCAGGGTCGGTTTGAGTACAATCGCGGGTTTTCGCCTACGGCTCGCGCAATGGGTTTTCGCGGCA : 603
Hjav : GGGTCCCCCCGGAGCCAGGCCTGTCTCAGGGTCGGTTTGAGTACAATCGCGGGTTTTCGCCTACGGCTCGCGCAATGGGTTTTCGCGGCA : 608

                                                                                                       
              640         *       660         *       680         *       700         *       720      
Hmed : GCGGATGCGTGTTTAATCGGGAGG-CTCGTCTCAGGGCTCTTT-------------------------AGAGCCGGAGTCTTTCGTCTCG : 677
Hver : GCGGATGCGAGTTTAATCGGGAGG-CTCGTCTCAGGGCTCTTT-------------------------AGAGCCGGAGTCTTTCGTCTCG : 675
Hori : GCGGATGCGTGTTTAATCGGGAGG-CTCGTCTCTAGGCTCTTG-------------------------AGAGCCGGAGCCTTTCGTCTCG : 669
Htro : GCGGATGCGTGTTCAATCGGGAGG-CTCGTCTCAGTGCTCTAG-------------------------AGAGCCGGAGCCTTTCGTCTCG : 667
Hnip : GCGGATGCGCGTTTATTCGGAAG--CTCGTCTCAGGGCTCTCTGTCGT------------------GGAGAGCCGGAGCTTTTCGTCTCG : 684
Htia : GCGGATGCGCGTTTATTCGGAAG--CTCGTCTCAGGGCTCTCTCTCGT------------------GGAGAGCCGGAGCTTTTCGTCTCG : 686
Wpig : GCGGATTCGAGTTTATTCGAAAGG-CTCGTCTCACAGGTCTCCTTCTTTCTTTA-----ACAGAGAGAAGAGCCGGAGCTTTTCGTCTCG : 694
Wlae : GCGGATTCGAGTTTATTCGAAAGG-TTCGTCTCACAGGTCTCCTTTCTCTATCACACAGAGAGAGAGAGGAACCGGAGCTTTTCGTCTCG : 699
Wacr : GCGGACGAGTGTTTATTCGGAAGG-CTCGCCTCAGGGCTCTCCTCTAC-----------------AGGAGGGCCGGAGCTTTTCGTCTCT : 680
Hman : GCGGATGCGAGTCGAATCGAGCGCTCTCCTCTCTGATGTCGTCTCTCT-----------GCAAGGGGAGACGGCGGAGCGTCTCGTCTCG : 682
Hjav : GCGGATGCGAGTCGAATCGAGCGCTCTCCTCTCTGATGTCGTCTCTCT-----------GCAAGGGGAGACGGCGGAGCGTCTCGTCTCG : 687

                                                                                                       
                *       740         *       760         *       780         *       800         *      
Hmed : CATTCGACGGCCGGGTTGCCAGAATGTCAGACCGTCGGCCATCTTTT-----AGGAGTTTGGCCGTCGACTAGATTCGTCCGTTGGTCGT : 762
Hver : CGTTCGACGGCCGGGTTGCCAGAATGTCAGACCGTCGGCCATCTTTT-----AGGAGTTTGGCCGTCGACTAGATTCGTCCGTTGGTCGT : 760
Hori : CTTTCGACGGCCGGGTTGCCAGAATGTCAGACCGTCGGCCATCTTTT-----AGGAGTTTGGCCGTCGACTAGATTCGTCCGTTGGTCGT : 754
Htro : CTTTCGACGGCCGGGTTGCCAGAATGTCAGACCGTCGGCCATCTTTT-----AGGAGTTTGGCCGTCGACTAGATTCGTCCGTTGGTCGT : 752
Hnip : CGTCCGAAGGCCGGGTTGCCAGAATGTCAGACCGGCGGTTGCGCGTC-----ACT-GCGCTGCCGTCGATTAGATTCGTCCGTTGGTCGT : 768
Htia : CGTCCGAAGGCCGGGTTGCCAGAATGTCAGACCGGCGGTTGCGCTTC-----ACT-GCGCTGCCGTCGATTAGATTCGTCCGTTGGTCGC : 770
Wpig : CGTCCGAAGGCCGGGTTGCCAGAATGTCAGACCGGCGGTTGCGCGTC-----ACT-GCGCTGCCGTCGATTAGATTCGTCCGTTGGTCGC : 778
Wlae : CGTCCGAAGGCCGGGTTGCCAGAATGTCAGACCGGCGGTTGCGCGTC-----ACT-GCGCTGCCGTCGATTAGATTCGTCCGTTGGTCGC : 783
Wacr : CGTCCGAAGGCCGGGTTGCCAGAATGTCAGACCGATGGTTGCGCT------------CGCAGCCGTCGACTAGATTCGTCCGTTGGTCGC : 758
Hman : CCTCCGAAGGCCGGGTTGCCAGAATGTCAGACCGTCGTCGTTTCTTTCGAAAGGGAGAATCGCCGGCGACTAGATTCGTCCTTAGGTCGC : 772
Hjav : CCTCCGAAGGCCGGGTTGCCAGAATGTCAGACCGTCGTCGTTTCTTTCGAAAGGGAGAATCGCCGGCGACTAGATTCGTCCTTAGGTCGC : 777

                                                                                                       
              820         *       840         *       860         *       880         *       900      
Hmed : GCGTCGTCTGGT-CCGTCGTCGCGGTGTGCATTGTCTCTAGACGGGC-----TTCGTCCG--------TCGGCACCCGCATGTCCCCTGT : 838
Hver : GCGTCGTCTGGT-CCGTCGTCGCGGTGTGCATTGTCTCTAGACGGGC-----TTCGTCCG--------TCGGCACCCGCATGTCCCCTGT : 836
Hori : GCGTCGTCTGGT-CCGTCGTCGCGGTGTGCATTGTCTCTAGACGGGC-----TTCGTCCG--------TCGGCACCCGCATGTCCCCTGT : 830
Htro : GCGTCGTCTGGT-CCGTCGTCGCGGTGTGCATTGTCTCTAGACGGGC-----TTCGTCCG--------TCGGCACCCGCATGTCCCCTGT : 828
Hnip : GCGTCGTCTGATACAGTCGTCGCGGGG-GCA--GTCTCTTGACGG-------TTCGTCCG--------TTGGCTCCCGCATGTCCCATTG : 840
Htia : GCGTCGTCTGATACAGTCGTCGCGGGG-GCA--GTCTCTTGACGG-------TTCGTCCG--------TTGGCTCCCGCATGTCCCATTG : 842
Wpig : GCGTCGTCTGGT-CCGTCGTCGCGGGG-GCA--GTCTCTTGACGG-------TTCGTCCG--------TCGGCTCCCGCATGTCCCATTG : 849
Wlae : GCGTCGTCTGAT-CCGTCGTCGCGGGG-GCA--GTCTCTTGACGG-------TTCGTCCG--------TCGGCTCCCGCATGTCCCATTG : 854
Wacr : GCGTCGTCTGGT-CCGTCGTCGCGGGG-GCA--GTCTCCTGGCGG-------TTCTTCCG--------TCGGCTCCCGCATGTCCCATTG : 829
Hman : GCGTCGTCTGGT-CCGTCGTCGCGGGA-GTG--GTCTCTTGACCGGTGGTCGTTCGCTCGACTTCCGGTCGGCTCTCGCACGTCCCATTG : 858
Hjav : GCGTCGTCTGGT-CCGTCGTCGCGGGA-GTG--GTCTCTTGACCGGTGGTCGTTCGCTCGACTTCCGGTCGGCTCTCGCACGTCCCATTG : 863

                                                                                                       
                *       920         *       940         *       960         *       980         *      
Hmed : ACGGT--CAGAACGAACGGCGGCTCGATC-GTGTCTCAAGAGACTCGCTTTCGGTTA-GGCGAGCGACCGAACGGAACGA--AAC-ATCA : 921
Hver : ACGGT--CAGAACGAACGGCGGCTCGATC-GTGTCTCAAGAGACTCGCTTTCGGTTA-GGCGAGCGACCGAACGGAACGA--AAT-ATCA : 919
Hori : ACGGT--CAGAACGAACGGCGGCTCGATC-GTGTCTCAAGAGACTCGCTTTCGGTTA-GGCGAGCGACCGAACGGAACGA--AAT-ATCA : 913
Htro : ACGGT--CAGAACGAACGGCGGCTCGATC-GTGTCTCAAGAGACTCGCTTTCGGTTA-GGCGAGCGACCGAACGGAACGA--AAC-ATCA : 911
Hnip : ACGGTATCGGAACGAACGGCGGCTCGATT-TTGTCTCAAGAGATTCGCTTTCGATTCTGGCGAGCGACCTTACGGAACGACGATC-ATCA : 928
Htia : ACGGTATCGGAACGAACGGCGGCTCGATT-TTGTCTCAAGAGATTCGCTTTCGATTCTGGCGAGCGACCGTACGGAACGACGATC-CTCA : 930
Wpig : ACGGTATCGGAACGAACGGCGGCTCGATT-TTGTCTCAAGAGATTCGCTTTCGATTCTGGCGAGCGACCGTACGGAACGACAATC-CTCA : 937
Wlae : ACGGTATCGGAACGAACGGCGGCTCGATT-TTGTCTCAAGAGATTCGCTTTCGATTCTGGCGAGCGACCGTACGGAACGACGATC-CTCA : 942
Wacr : ACGGTATCGGAACGAACGGCGGCTCGATT-ATGTCTCAAGAGATTCGCTTTCGATTCTGGCGAGCGACCGTACGGAACGATTATC-CTTT : 917
Hman : ACGG---CCAGCCGAACGGCGGCTCGATTATTGTCGCAAGAGATTCGCTTTCGGTAAAGGCGAGCGATCTAAAGGAACGAACGTCTCTCT : 945
Hjav : ACGG---CCAGCCGAACGGCGGCTCGATTATTGTCGCAAGAGATTCGCTTTCGGTAAAGGCGAGCGATCTAAAGGAACGAACGTCTCTCT : 950

                                            
             1000         *      1020       
Hmed : AA-ATTCGACCTGAGATCAGACCGGATTACC : 951
Hver : AA-ATTCGACCTGAGATCAGACCGGATTACC : 949
Hori : AA-ATTCGACCTGAGATCAGACCGGATTACC : 943
Htro : AA-ATTCGACCTGAGATCAGACCGGATTACC : 941
Hnip : ACTATTCGACCTGAGATCAGACCGGATTACC : 959
Htia : ACTATTCGACCTGAGATCAGACCGGATTACC : 961
Wpig : ACTATTCGACCTGAGATCAGACCGGATTACC : 968
Wlae : ACTATTCGACCTGAGATCAGACCGGATTACC : 973
Wacr : ACTATTCGACCTGAGATCAGACCGGATTACC : 948
Hman : CCATTTCGACCTGAGATCAGACCGGATTACC : 976
Hjav : CCAATTCGACCTGAGATCAGACCGGATTACC : 981


degrees of pair wise sequences identities

         Hmed   Hver   Hori   Htro   Hnip   Htia   Wpig   Wlae   Wacr   Hman   Hjav 

  Hmed    951     98%    98%    97%    83%    83%    82%    81%    82%    77%    77%
            0     98%    98%    97%    83%    83%    82%    81%    82%    77%    77%
            0      0%     0%     1%     4%     4%     5%     6%     5%     6%     6%

  Hver    942    949     98%    97%    83%    83%    82%    81%    82%    76%    77%
          943      0     98%    97%    83%    83%    82%    81%    82%    77%    77%
            6      0      0%     0%     4%     4%     6%     6%     5%     6%     6%

  Hori    933    932    943     98%    83%    82%    82%    81%    82%    76%    77%
          933    933      0     98%    83%    82%    82%    81%    82%    76%    77%
            8      6      0      0%     5%     5%     6%     7%     6%     7%     7%

  Htro    928    925    928    941     83%    83%    82%    81%    82%    77%    77%
          928    926    928      0     83%    83%    82%    81%    82%    77%    77%
           10      8      2      0      5%     5%     6%     7%     5%     7%     7%

  Hnip    816    815    812    813    959     98%    94%    93%    92%    76%    76%
          816    815    812    813      0     98%    94%    93%    92%    76%    76%
           46     48     54     52      0      0%     2%     2%     1%     6%     6%

  Htia    815    814    811    812    948    961     94%    93%    92%    76%    76%
          815    814    811    812    948      0     94%    93%    92%    76%    76%
           46     48     54     52      2      0      2%     3%     1%     7%     6%

  Wpig    816    816    814    815    921    922    968     97%    92%    77%    77%
          816    816    814    815    921    922      0     97%    92%    77%    77%
           59     61     67     65     23     25      0      0%     2%     6%     6%

  Wlae    810    810    808    809    919    920    949    973     91%    76%    76%
          810    810    808    809    919    920    949      0     91%    76%    76%
           64     66     72     70     28     30      5      0      2%     7%     7%

  Wacr    806    804    805    806    892    895    895    891    948     75%    76%
          806    804    805    806    892    895    895    891      0     75%    76%
           51     53     59     57     17     19     22     27      0      7%     7%

  Hman    768    767    765    770    768    767    777    775    757    976     98%
          768    768    765    770    768    767    777    775    757      0     98%
           67     69     75     77     69     71     68     73     74      0      0%

  Hjav    772    771    769    774    772    772    781    779    762    969    981 
          772    772    769    774    772    772    781    779    762    969      0 
           64     66     72     74     68     66     67     72     73      5      0
