# Supplementary material for: The great diversity: monomeric and oligomeric hirudins, hirudin-like factors and decorsins in the Asian medicinal leeches Hirudo nipponia and Hirudo tianjinensis
Source: Parasitol Res. 2026 Feb 7;125(1):18. doi: 10.1007/s00436-026-08634-0 (PMC12882960; doi:10.1007/s00436-026-08634-0)
Supplement: Supplementary file 1 — Supplementary Material 1 (ZIP 660 KB) [file 436_2026_8634_MOESM1_ESM.zip › S1_putative hirudin genes on chromosome 2 of Hirudo nipponia.docx]

Supplementary Information File S1: Localization of putative monomeric and multimeric hirudin genes on chromosome 2 of *Hirudo nipponia*

**chromosome 2 position 18942726 – 18944000**

**atg**tttcctttgaagctatttgttgttctttttgcttcttttgacatttgcttgtctgaaa*gt*gagtttgatatgagtttatcaatgatagaatcatatgtaataaaaccagcttaaccggtaatgttggacggatggatggataaatgtttcattacatgccccttctgtttacattgaaaatgaatattggttgt*ag*ttaaaaga**tgc**tcagaaaataacaagactcct**tgc**cta**tgt**gag*gt*taagtattaaactacaacttttattcatcaagtaataacattaacaacgtttgacatcatcatcgaacattc*ag*aaagatgaaaaagaagta**tgt**cctcaggggagcaaa**tgt**caagtggatcgttatggtggtggcaactta**tgc**tacaaaa*gt*aattatgcttatgaactaatttatatatagtaaaataatgtatttttattaacactattttataaaaatataaattatttcatttttaatgttattaccaactgctctgaatctgttattaattaatcgatgcatattgtgtgtaaatctgaaatctaatgaattgctacttattatttaagaaaacaactttttgataaattattaatttttaaatttatttcccaaattgtaatctctgc*ag*gtaacagcgcacgcaactctccgcatggaatcatcataatg**tgctgt**ttgatacctcttgtatattttgaattattaaataaaatgttattgatt**taataa**

**atg**tttcctttgaagctatttgttgttctttttgcttcttttgacatttgcttgtctgaaa

ttaaaaga**tgc**tcagaaaataacaagactcct**tgc**cta**tgt**gag

aaagatgaaaaagaagta**tgt**cctcaggggagcaaa**tgt**caagtggatcgttatggtggtggcaactta**tgc**tacaaaa

gtaacagcgcacgcaactctccgcatggaatcatcataatg**tgctgt**ttgatacctcttgtatattttgaattattaaataaaatgttattgatt**taataa**

MFPLKLFVVLFASFDICLSEIKR**C**SENNKTP**C**L**C**EKDEKEV**C**PQGSK**C**QVDRYGGGNL**C**YKSNSARNSPHGIIIM**CC**LIPLVYFELLNKMLLI--

Theoretical pI/Mw: **8.20** / 8353.82

**Hnip_HV4 (this study)**

**chromosome 2 position 18971000 – 18972160 rev+comp**

**atg**tttcctctgaagctatttgttgttctttttgcttcttttgacatttgcttgtctgaaa*gt*gagtttgataagagtttatcaatgatagaatcatatgtgataaaaccagcttaaccggtaatgttggacggatggatggatagatgtttctttacatgccccttctgtttacattgaaaattaatattggttgt*ag*ttgaagga**tgc**atagaaaaaggcattactcct**tgc**cta**tgt**gag*gt*taactattaaactataacttttatttatcaagtattaacattaacaacgtttgacatcatcatcgaacattc*ag*aaatatgaaagcgtgtta**tgt**cctgaaggtagcaga**tgt**cgaatagattatcgtggcaacata**tgc**tccgaaa*gt*aattatgcttatgaactaatttatatatagtaaaataatgcatttttattaacactattttataaaaatataaattatttcatttttaatgttattaccaactgctctgaatctgttattaattaatcgatgcaaatcgtgtgtaaatctgaaatctaatgaattgctaattattatttaagaaaacaactttttgatgaattattaatttttaaatttatttcccaaattgtaatctctgc*ag*gtaac*ag*cgaactcaacccttcgcatggaatcatcataatg**tgctgt**ttgatacctcttgtatattttgaattattaaataaaatgttattgatt**taataa**

**atg**tttcctctgaagctatttgttgttctttttgcttcttttgacatttgcttgtctgaaa

ttgaagga**tgc**atagaaaaaggcattactcct**tgc**cta**tgt**gag

aaatatgaaagcgtgtta**tgt**cctgaaggtagcaga**tgt**cgaatagattatcgtggcaacata**tgc**tccgaaa

gtaac*ag*cgaactcaacccttcgcatggaatcatcataatg**tgctgt**ttgatacctcttgtatattttgaattattaaataaaatgttattgatt**taataa**

MFPLKLFVVLFASFDICLSEIEG**C**IEKGITP**C**L**C**EKYESVL**C**PEGSR**C**RIDYRGNI**C**SESNSELNPSHGIIIM**CC**LIPLVYFELLNKMLLI--

Theoretical pI/Mw: **4.78** / 8111.58

**Hnip_HV5 (this study)**

**chromosom 2 position 19153472 - 19159914**

**atg**ttgaagctgtttgttgtcctcttggcttcttttggtatcggtctgtctcaag*gt*gagtatgatctgagtttctgaacgaaagagtgatatgtaataaatacagcttcaccaatgatttgggatggatggattgatttgatagatggacgcatggatgtaaggatggatggatttaacatttcataccgtgataaattcacttctgtttattttaaaaatgaatttgtaatttgttggttgt*ag*aggtttataaacac**tgc**tctgatcgaaatgggaatgaacca**tgc**ctc**tgc**ggg*gt*aaattataagatttatttacaaaatataatcataaataacgtataacatgacattattaatatcttcattgaacattc*ag*gatggctctaaggta**tgt**ggtcgatatgaagat**tgt**gaagaaaacaaa**tgc**aaaa*gt*aatttttatgctaaaaatttactattaattttacaatttttaataattttatttttgttgttaatctattttttttaataatttcgttttttttaaattttatttttacatatataatattgtaacttattactactactattattattaatgaaagagtgacacttaacaaaaagcgccttaccaataacttggaatagatggatagatggatagatggatggatggatataacattacataccgtgatattttatttcctatttattttaaaaataaatgttggttgt*ag*cgatttttgatccc**tgc**tacattcgagatagtaggagtcca**tgc**ctc**tgc**agt*gt*aagttataagattacaactgttatttatcaattgttaaataacgtataatatcatcattgaacattc*ag*gataatcatgta**tgc**aaggacgatgaaatt**tgt**gacatgtggttaaacaaa**tgc**aaaa*gt*aattttcaaataataaaatttgtttataaagttttttattgattttatttttacaaacataatatggtaatttattattgatactattctcttaatagttatgataaaaattaatgttgtttgt*ag*aaaaatac**tgt**tcattaagaaatgtatggcca**tgc**tat**tgt**gag*gt*aaattataagataataacttttatttaccaaatgttatcataaaaagttcaatatcatcattgaacattc*ag*gaggatagtatg**tgt**aagaaagatgaaatt**tgt**gaaataggaaagaca**tgt**tatttaggaaaaaga**tgt**gatttgaagtctaaacccagcaaa**tgc**aaatttg*gt*aatttttttaacaatgaaatttaataataattttacttatgttactaaatttaatatttatattaattttaattttttcaacattaatattgtacgttattgtttgtactatgttcttatttttatattaaaaatatttgttggttgt*ag*aaaaa**tgc**acgaaaagaaatcgatatcct**tgc**tac**tgt**gtg*gt*aaattataagataataacttttagttatcaaatgttattataaaaaacgtataatatcatcattggacattc*ag*aatgactctcaagta**tgt**ggtgcaaatgaagat**tgt**caactaaatcctgagggaaacaaa**tgc**atag*gt*aatttttataagttattaattttatatttttattaattttatttgtacataaatattattgtaatttattattaatgctattatcctcaatgaaaaagtgatatgtaatacacagtgcctcactaccgtgataaattcacttcttctaaaaatattgttggttgt*ag*agattagtattcaa**tgc**tctgatcgaaaagggaatgaagca**tgc**ttc**tgt**ggg*gt*aaatgataagattacaatttttagttatcaaatgttatcataaataacgtgtaataccttcattgaacattc*ag*aatgaaactata**tgt**tatgaacaagaattt**tgt**gaaggaaacaaa**tgc**aaaa*gt*aatttgtatgcaataaaatttagtgttaaatttacaaatgttattaattacatttttgtgacttgagttccgaatttatattatgttcttaatatttatattaaaaataattgttagttgt*ag*aaaaa**tgc**aagaaaaataatgaatatcct**tgc**tac**tgt**gtg*gt*aaatttaaaaataataactttcagttaacaaatattatcataaataacgtataatatcatcattggacattc*ag*aatgactcgaaagta**tgt**ggtgaatatcaagag**tgt**aaactgaattctgaaggaaacaga**tgc**acag*gt*aatttttataagttattatttttatatttttattaattttaattttttatgttattaattttatttttattataaatttcattttgttttaaatttacacaaataatatattaatttattattaatattattacgctcgaaaaaaagaatgggtgggtggatgaatggttttgtaggtggatagatacatggatgtaaagatggatggatataacatgacataccgtgatatattcacttctgtttattttcaaaatgattgttgtttgtagagaattacagaccctgccctgattggaatgaaccatgcctctgtggggtaagttataagattacaacttttatttacaaaatgtaatcataaataacgtatgatatcataatttaaataaattaaaataaaataaataaatttaatttatcaactgttatcataattgtttttaagtgagtttattgaatcaaacaaacaaggcgttatatacacagaaaaaaataaacgggaaggcactacataaattcaaataaatccaaagggccagaattagggtccgctggctttatacaaaaatcataagctatacaggtccactcaataaatattgtttgcacgatattttgaatagtatataaagtaatttgaaagggcatgtgcttaactgacttagatacttgataaatgaaggtatatttaatataaataatctaaaatatgttattattaatatcttcattgaacattcagaatgactccaaggtatgtgatccatatcaacattgtaaaggaaacacctgcatag*gt*aatttttaattaataatgaatggatggatggatggatggatggatgggtggatgaatggatggaccaatggatagatggatataatattacataccgtgataaattcacttctgtttattttaaaaatgaatgttggttgt*ag*agatttacaacaac**tgc**tctaatcgagagggggattattat**tgc**cgc**tgt**ggg*gt*aagataagattacaactgttatttatcaaatgttatcataaataacatgtaatatcatcattgaacattc*ag*aatgaaactata**tgt**ttcggtcatcaaatt**tgt**gacacaaagacaaacaaa**tgc**aaaa*gt*aattttcatacaataaaatttgttaataatgtttgctattaattttatttttacaaaaatattgtaatttattaactatgattttaatatttttaataaaaatgaatgttggttgt*ag*aaaaatac**tgt**tcggaagaatcaaatgagtggcca**tgc**ttc**tgt**gaa*gt*aaattataagataataactttcagttatcaaatgttatcataaataacttataatatcatcattgaacattc*ag*gagaatcgcatc**tgt**tattcatatgaaaaa**tgt**gaactgaatcctgaaggaaacaaa**tgc**atatatg*gt*tattttgtataatgaaatttattaataattttacttattttgttaattttattttaattattaatttcaatttttttctatttaattttatttttacaaaaacaatattaaaatttattattatttaatttatattatgttcttattttttatattaaatatgaatgttggttgt*ag*aaaaa**tgc**acggaaagtaatcaatatcct**tgc**tac**tgt**gtg*gt*aaatcataaaataataactttcagttaacaaatgttatcataaataacgtataatatcatcattggacattc*ag*aatgactctcaaata**tgt**gctgaaaatgaacgt**tgt**gaactaaattctgaaggaaacaaa**tgc**atag*gt*aatttttattaagttattttatattcttattaatttcattttttattaattttatttttgcataaatattattgtcatttattattaatactattatcctcaacgaaatagttatacgtaatggaggggtggatggatggatggatggatggaaggatggatggatggatggatggatggatggatggatggatggatggatggatggatggatggatggatggatggatggatggatgggtggatggatggatataacattatataccgcgacaaattcaattctgtttattttaaaaatgaatgttgttgt*ag*aggtttataaacac**tgc**tctgaacgaaagtggaatgaaaaa**tgc**ctc**tgt**gcg*gt*aaattacaagattacaacttctatttatcaattgttttcataaataacattaatatcatcattgatcattt*ag*aatgaatccatg**tgt**tcacgaaatgaatat**tgt**gacctgaattatgaaggatacaaa**tgc**acac*gt*aatttttatgcaataaaatttaccattaattttattttgtgttattttttatgttaatattgattgatgaatggatgggtggatggatggatggatggatggatggatggatggatggatggatggatggatggatggatggatggatggatggatggatggatggatggatggatggatggatggatggatggatggatggatggatggatggatggatggatggatggatagatggatggatataacattacattctattttctttaaaaatgaattttggttgt*ag*agatttataaacac**tgc**tctgatcaaaatgggaatgaacca**tgc**ctc**tgt**ggg*gt*aaattataagattacaacttttatttatcaaatgttatcataaataacgtataatatcgacactaaacattc*ag*ggtaaaagcata**tgc**aatgaaaacgaaatt**tgt**gacctgaaaattaacaaa**tgc**aaaa*gt*aattttcaaattataaaatttttattaattttaactttaaaaaaaaataatattgttatttattagtgatctatatatttaatacaaggatattgtatgtatatttcttataaaatctttaattctgaaccgatttcattggaacttgaaacacttgagcacgttgactgtcagtttttttttgtttgttttttttttggaacacaaaaccagtacgagttggaaccgaaaagaaaaaaaaggaacgtcataaaggatggatggatggatggatgatggatggatggatggatggatggatggatggatggatggatgtatataacattacattctgttttctttaaaaatgaattttggttgt*ag*agaattataaacac**tgc**tctgatcaaaatgggaatgaacca**tgc**ctc**tgt**ggg*gt*aaatgataagattaaaactttatttatcaaatgttatcataaataatgtataatatcgtcactgaacattc*ag*ggtaaaagcata**tgc**aataaaaaggaaatt**tgt**gacctgaaaattaacaaa**tgc**aaaa*gt*aattttcaaattataaaatttttattaattttaactttttaaacaatataaaattgtaatttgttattaatctatatatttaaaacaaggatcttatatgtatatttcttatgcaaatctttaatcctgaaccgatttgattgaaacttgaaacacttgagtacgttaactatggggtcagtttagttttataacttaaatttgaatttacttggaaaagaaatcgataaattatttttcgtattattgccaaaaaacctctcctgggtttgttctcacgaaagtgttcgcgcagttttcaaagctggtgatcttaggcgcatgccgattaatattcatgtgcttaaacatttagtttatattataaattttccgttttatattttaaaattcacattttttattatttatctatgatttaatattatattaatattagcattatgttggtagccatcttgtcattcataatttctagccgtgtattcatcgttgcaaataaaagaatttaagtttattaatttgcatatctattaccgggca*ag*gaggtaaacgaagaggcgggaaaacagccagaataa*gt*aatttttaatattaaaatttgcaattaatttcagttcttttattaattttgttttattattaattttatttttgttagttatttttttatgttttaaaaaacaatgttcttattaatttcatttgtttgttatcaatttagttttataaaaataataatgtaattcattattagtattatgcttttaacctggtattaattaatagaggcaaacagtgtctaaagttgaaatttcttttttgactc*ag*aggtg**taa**

**atg**ttgaagctgtttgttgtcctcttggcttcttttggtatcggtctgtctcaag

aggtttataaacac**tgc**tctgatcgaaatgggaatgaacca**tgc**ctc**tgc**ggg

gatggctctaaggta**tgt**ggtcgatatgaagat**tgt**gaagaaaacaaa**tgc**aaaa

cgatttttgatccc**tgc**tacattcgagatagtaggagtcca**tgc**ctc**tgc**agt

gataatcatgta**tgc**aaggacgatgaaatt**tgt**gacatgtggttaaacaaa**tgc**aaaa

aaaaatac**tgt**tcattaagaaatgtatggcca**tgc**tat**tgt**gag

gaggatagtatg**tgt**aagaaagatgaaatt**tgt**gaaataggaaagaca**tgt**tatttaggaaaaaga**tgt**gatttgaagtctaaacccagcaaa**tgc**aaatttg

aaaaa**tgc**acgaaaagaaatcgatatcct**tgc**tac**tgt**gtg

aatgactctcaagta**tgt**ggtgcaaatgaagat**tgt**caactaaatcctgagggaaacaaa**tgc**atag

agattagtattcaa**tgc**tctgatcgaaaagggaatgaagca**tgc**ttc**tgt**ggg

aatgaaactata**tgt**tatgaacaagaattt**tgt**gaaggaaacaaa**tgc**aaaa

aaaaa**tgc**aagaaaaataatgaatatcct**tgc**tac**tgt**gtg

aatgactcgaaagta**tgt**ggtgaatatcaagag**tgt**aaactgaattctgaaggaaacaga**tgc**acag

agatttacaacaac**tgc**tctaatcgagagggggattattat**tgc**cgc**tgt**ggg

aatgaaactata**tgt**ttcggtcatcaaatt**tgt**gacacaaagacaaacaaa**tgc**aaaa

aaaaatac**tgt**tcggaagaatcaaatgagtggcca**tgc**ttc**tgt**gaa

gagaatcgcatc**tgt**tattcatatgaaaaa**tgt**gaactgaatcctgaaggaaacaaa**tgc**atatatg

aaaaa**tgc**acggaaagtaatcaatatcct**tgc**tac**tgt**gtg

aatgactctcaaata**tgt**gctgaaaatgaacgt**tgt**gaactaaattctgaaggaaacaaa**tgc**atag

aggtttataaacac**tgc**tctgaacgaaagtggaatgaaaaa**tgc**ctc**tgt**gcg

aatgaatccatg**tgt**tcacgaaatgaatat**tgt**gacctgaattatgaaggatacaaa**tgc**acac

agatttataaacac**tgc**tctgatcaaaatgggaatgaacca**tgc**ctc**tgt**ggg

ggtaaaagcata**tgc**aatgaaaacgaaatt**tgt**gacctgaaaattaacaaa**tgc**aaaa

agaattataaacac**tgc**tctgatcaaaatgggaatgaacca**tgc**ctc**tgt**ggg

ggtaaaagcata**tgc**aataaaaaggaaatt**tgt**gacctgaaaattaacaaa**tgc**aaaa

gaggtaaacgaagaggcgggaaaacagccagaataa

aggtg**taa**

MLKLFVVLLASFGIGLS

QEVYKH**C**SDRNGNEP**C**L**C**GDGSKV**C**GRYED**C**EENK**C**K

TIFDP**C**YIRDSRSP**C**L**C**SDNHV**C**KDDEI**C**DMWLNK**C**K

KKY**C**SLRNVWP**C**Y**C**EEDSM**C**KKDEI**C**EIGKT**C**YLGKR**C**DLKSKPSK**C**K

FEK**C**TKRNRYP**C**Y**C**VNDSQV**C**GANED**C**QLNPEGNK**C**I

EISIQ**C**SDRKGNEA**C**F**C**GNETI**C**YEQEF**C**EGNK**C**K

KK**C**KKNNEYP**C**Y**C**VNDSKV**C**GEYQE**C**KLNSEGNR**C**T

EIYNN**C**SNREGDYY**C**R**C**GNETI**C**FGHQI**C**DTKTNK**C**K

KKY**C**SEESNEWP**C**F**C**EENRI**C**YSYEK**C**ELNPEGNK**C**I

YEK**C**TESNQYP**C**Y**C**VNDSQI**C**AENER**C**ELNSEGNK**C**I

EVYKH**C**SERKWNEK**C**L**C**ANESM**C**SRNEY**C**DLNYEGYK**C**T

QIYKH**C**SDQNGNEP**C**L**C**GGKSI**C**NENEI**C**DLKINK**C**K

KNYKH**C**SDQNGNEP**C**L**C**GGKSI**C**NKKEI**C**DLKINK **C**KRGKRRGGKTARIKV--

Theoretical pI/Mw: **7.53** / 54088.23

**Hnip_mHV1 (this study)**

**chromosome 2 position 19165680 – 19167819**

**atg**ttgaagctgtttgttgtcctcttggcttcatttggtatcggtctgtctcaag*gt*gagtatgatctgagtttctgaacgaaagagtgatatgtaataaatacagcttcaccaatggtatggggtggatgtacctcaccgataaaaatatgcgagtaaaaacatgaattgtctaacgagcggttattctaagagctctgactaaataaatcattttattgaatgctcgtcgtgcgaataattctgaaattaaacgtaaaaaatgtcattaactatatgaaatccaatacattttcaccgataaaaagttacaaaaattttgtaaaagtattatattaatactagtgttgaagttgttttaagaatttgatcatttattttttcttaaaaattcattacgtcgagcgacaaaatccgaatataatcgtaaattttattgttatttttgagttataaatatgtcattaatgataaaaaatcccacaaatttccatcgataaaagtaaacaaaaggaacaattattcaaaaaatataaagatgtccttaaaccacctctcccatgttgccctgctttttaacgaatgcgagtgtttaagctgtaggcaaatgagtttgaattatttcttctaacatttttaaatttttgagaccctttttgcgtggtttaaaacttaagaacaattgtaaaatatattttatttcttattaaagttattttgctttttaaaattatctttattgcagaaatttaattaattgttaaattgtagagtaatgggttaaattagtaccgctattttactacataatttatttcatatctatggtgatgatgatgatgatgatgatgatgatgatgatgatgatgatgatgatgatgatgatgatgatgatgatgatgatgatgatgatgatgatgatgatgatgatgatgatgatgatgatgatgatgatgatgatgatgatgatgatgatgatgatgatgatgatgatgatgatgatgatgatgatgatgatgatgatgatgatgatgatgatgatgatgatgatgatgatgatgatgatgacaatttattaactatgattttaatatttttaataaaaataaatgttggttgt*ag*aaaaatac**tgt**tcggaagaatcaaatgagtggcca**tgc**ttc**tgt**gaa*gt*aaattataagataataactttcagttatcaaatgttatcataaataacttataatatcatcattgaacattc*ag*gagaatcgtgtc**tgt**tatttttatgaaaaa**tgt**gaactgaatcctgaaggaaacaaa**tgc**atatatg*gt*tattttgtataattactaattttacttattttgttaattttatttttattattaattttaattttttctatttaattttatttttaaaaacaatattataatatattattatttaatttacattatgttcttattttttatattaaatatgaatgttggttat*ag*aaaaa**tgc**acgaaaagaaatcct**tgc**tac**tgt**gta*gt*aaatcataaaataataactttaagatatcaaatgttatcataaataacgtataatatcatcatgttatcgtaaataacgtataatatcatcattgaacattc*ag*aatgactctaaaata**tgt**gctaaaaatgaagct**tgt**atagaaaacaga**tgc**atag*gt*aatttgtataagttattttatatttttattaatttcattttttattaattttatttttgcataaatgttattgtcatttattattaatactattttaattttcaaattaaaaagtcgataaattatttttcgtaatattgccaaaaaacctctcctgggtttgttctcacgagagcgttcgcgcagttttcaaaactggtgagcttaggattaatattcatgtgcttaaacatttagtttatattgcaaattttccgttttattttttaaaatgtacattttttattatttatctatgatttattattacattaatattagcattatgttggtagccatcttgtcattcataatttctagccgtgcattcgtcgttgcaaataaaagaatttaagtttattaatttgcatatctattaccgggcaa*ag*gaggaaacgagaaggcaggaaaacagctagaa**taa**

**atg**ttgaagctgtttgttgtcctcttggcttcatttggtatcggtctgtctcaag

aaaaatac**tgt**tcggaagaatcaaatgagtggcca**tgc**ttc**tgt**gaa

gagaatcgtgtc**tgt**tatttttatgaaaaa**tgt**gaactgaatcctgaaggaaacaaa**tgc**atatatg

gaggaaacgagaaggcaggaaaacagctagaa**taa**

MLKLFVVLLASFGIGLSQEKY**C**SEESNEWP**C**F**C**EENRV**C**YFYEK**C**ELNPEGNK**C**IYGGNEKAGKQLE-

QEKY**C**SEESNEWP**C**F**C**EENRV**C**YFYEK**C**ELNPEGNK**C**IYGGNEKAGKQLE

Theoretical pI/Mw: **4.54** / 5920.51

**Hnip_HV6 (Müller et al. 2025)**

**atg**ttgaagctgtttgttgtcctcttggcttcatttggtatcggtctgtctcaag

aaaaatac**tgt**tcggaagaatcaaatgagtggcca**tgc**ttc**tgt**gaa

gagaatcgtgtc**tgt**tatttttatgaaaaa**tgt**gaactgaatcctgaaggaaacaaa**tgc**atatatg

aaaaa**tgc**acgaaaagaaatcct**tgc**tac**tgt**gta

aatgactctaaaata**tgt**gctaaaaatgaagct**tgt**atagaaaacaga**tgc**atag

gaggaaacgagaaggcaggaaaacagctagaa**taa**

MLKLFVVLLASFGIGLS

QEKY**C**SEESNEWP**C**F**C**EENRV**C**YFYEK**C**ELNPEGNK**C**I

YEK**C**TKRNP**C**Y**C**VNDSKI**C**AKNEA**C**IENR **C**IGGNEKAGKQLE-

Theoretical pI/Mw: **5.04** / 9352.51

**Hnip_mHV2 (this study)**

**chromosome 2 position 19188245 – 19194790 rev+comp**

**atg**ttgaagctgtttgttgtcctcttggcttcttttggtatcggtctgtctcaag*gt*gagtatgatctgagtttctgaacgaaagagtgatatgtaataaatacagcttcaccaatgatttgggatggatggattgatttgatagatggacgcatggatgtaaggatggatggatttaacatttcataccgtgataaattcacttctgtttattttaaaaatgaatgttggttgt*ag*agatttataaacac**tgc**tctgatcgaaatgggaatgaacca**tgc**ctc**tgt**ggg*gt*aaattataagatttctttacaaaatataatcataaataacgtataacatgacattattaatttcttcattgaacattc*ag*gatggctctaaggta**tgt**ggtcgatatgaagat**tgt**gaagaaaacaaa**tgc**aaaa*gt*aatttttatgcttaaaatttactattaattttacaatttttaataatttcgtttttttttaattttatttttacatatataatattgtaacttattactactactattattattaatgaaagagtgacacttaacaaaaagcgcctcaccaataacttggaatagatggatagatggatggatggatataacattgcataccgtgatattttatttcctatttattttaaaaatgaatgttggttgt*ag*cgatttttgatccc**tgc**tacattcgagatagtaggagtcca**tgc**ctc**tgt**ggg*gt*aagttataagattacaactgttatttatcaattgttaaataacgtataatatcatcattgaacattc*ag*gataatcttgta**tgc**aaggacgatgaaatt**tgt**gacatatggaataacaga**tgc**ataa*gt*aattttcaaataataaaatttgttaaaaaatgtttttattgattttatttttacaaacataatatggtaatttattattgatactatgctcttaatatttatgataaaaattaatgttgtttgt*ag*aaaaatac**tgt**tcattaagaaatgtatggcca**tgc**tat**tgt**gag*gt*aaattataagataataacttttatttaccaaatgttatcataaagacgttcaatatcatcattgaccattc*ag*gaggatagtatg**tgt**aagaaagatgaaatt**tgt**gaaataggaaagaca**tgt**tatttaggaaaaaga**tgt**gatttgaagtctaaacccagcaaa**tgc**aaatttg*gt*aatttttttacaatgaaatttaataataattttacttatgttactaaatttaatttttatattaattttaattttttcaacattaatattgtaatttattgtttgtactatgttcttatttttatattaaaaatatttgttggttgt*ag*aaaaa**tgc**acgaaaagaaatcgatatcct**tgc**tac**tgt**gtg*gt*aaattataagataataacttttagttatcaaatgttatcataaaaaacgtataatatcatcattggacattc*ag*aatgactctcaagta**tgt**ggtgcaaatgaagat**tgt**caactaaatcctgagggaaacaaa**tgc**atag*gt*aatttttataagttattaattttatatttttattaattttatatttacataaatattattgtaatttattattaatgctattatcctcaatgaaaaagtgatatgtaatacaaagtgcctcactaccgtgataaattcacttcttctaaaaatattgttggttgt*ag*agattagtattcaa**tgc**tctgatcgaaaagggaatgaagca**tgc**ttc**tgt**ggg*gt*aaatgataagattacaacttttagttatcaaatgttatcataaataacgtgtaataccttcattgaacattc*ag*aatgaaactata**tgt**tatgaacaagaattt**tgt**gaaggaaacaaa**tgc**aaaa*gt*attttttatgcaataaaatttagtgttaaatttacaaatgttattaattacatttttgtgacttgagttccgaatttatattatgttcttaatatttatattaaaaatgattgtt*ag*ttgtagaaaaa**tgc**aagaaaaataatgaatatcct**tgc**tac**tgt**gtg*gt*aaatttaaaaataataactttcagttaacaaatattatcataaataacgtataatatcatcattggacattc*ag*aatgactcgaaagta**tgt**ggtgaatatgaagag**tgt**aaactgaattctgaaggaaacaga**tgc**acag*gt*aatttttataagttattatttttatatttttattaattttaattttttatgttattaattttatttttattataaatttcattttgttttgaatttacacaaataatatttcaatgtattattaatattattacgctcaaaagaaagaatgggtgggtggatgaatggttttgtaggtggatagatacatggatgtaaagatggatggatataacatgacataccgtgatatattcacttctgtttattttcaaaatgaatgttgtttgtagagaattacagaccctgccctgattggtatgaaccatgcctttgtggggtaagttataagattacaacttttatttgcaaaatgtaatcataaataacgtatgatatcataatttaaaataaatttaatttatcaactgttatcataattgtttttaagtggtttattgaatcaaacaaacaaggcgttatatacacagaaaaaaaacgggaaagcactacataaattcaaataaatccaaagggccagaattagggcgttatacaaaaatcataagctatacaggtccactcaataaatattgtttgcacgatattttgaatagcatataaagtaatttgaaagggcatgcgcttaactgacttagatacttgatagatgaaagtatgattaatatatataacctaaaatatgttattattaatatcttcattgaacattcagaatgactccaaggtatgtgataaatatcaacaatgtgacggaaacacctgtataggtaatttttaattaataatgaatggatggatggatggatggatgggtggatgaatggatggctggatggacgaatggatagatggatataatattacataccgtgataaattcacttctgtttattttaaaaatgaatgttggttgt*ag*agagttacaaaaac**tgc**tctgatccagaaaggaattattat**tgc**cgc**tgt**ggg*gt*aagataagattacaactgttatttatcaaatgttatcataaataatatgtaatatcatcattgaacattc*ag*aatgaaactgta**tgt**ttgcttcatcaaatt**tgt**gacacaaagacaaacaaa**tgc**aaaa*gt*aattttcatacaataacatttgttaataatgtttgctattaattttatttttaagaaaatagtattgaaatttattattattaatactatgatcttaatatttatattaaaaatgaatgttggttgt*ag*aaaaatac**tgt**tcggaatcaaataagcatcca**tgc**ttc**tgt**gag*gt*aaattataagataataactttcagttatcaaatgttatcataaataacttataatatcatcattgaacattc*ag*gagaatcgtatc**tgt**tatttatatgaaaaa**tgt**gaactgaatcctgaaggaaacaaa**tgc**atatatg*gt*tattttgtataatgaaatttattaataattttacttattttgttaattttatttttattattaatttcaatttttttctatttaattttatttttacaaaaacaatattaaaatttattattatttaatttatattatgttcttattttttatattaaatatgaatgttggttgt*ag*aaaaa**tgc**acggaaagtaatcaatatcct**tgc**tac**tgt**gtg*gt*aaatcataaaataataactttcagttaacaaacgttatcataaataacgtataatatcataattggacattc*ag*aatgactctcaaata**tgt**gctgaaaatgaacgt**tgt**gaactgaattctgaaggaaacaga**tgc**atag*gt*aatttttattaagttattttatattcttattaatttcattttttattaattttatttttgcataaatattattgtcatttattattaatactattatcctcaacgaaatagttatatgtaatggaggggtggatgggtggatggatggatggatggatggaaggatggatggatggatggatggatggatggatggatggatggatggatggatggatgggtggatggatggatggatggatggatggattgatggatggatggatggatggatggatggatggatggatggatggatggatgggtggatggatggatggatggatggatggatggatggatggatggatggatggatggatggatggatggatggatggatggatggatgggtggatggatggatataacattatataccgcgacaaattcaattctgtttattttaaaaatgaatgttgttgt*ag*aggtttataaacac**tgc**tctgaacgaaagtggaatgaaaaa**tgc**ctc**tgt**gcg*gt*aaattacaagattacaacttctatttatcaattgttttcataaataacattaatatcatcattgatcattt*ag*aatgaatccatg**tgt**tatcgagatgaatat**tgt**gacctgaattatgaaggatacaaa**tgc**acag*gt*aatttttatgcaataaaatttaccattaattttattttgtgttattttttatgttaattttaattgatgaatggatgggtggatggatggatggatggatggatggatggatggatggatggatggatggatggatggatggatggatggatggatggatggatggatggatggatggatggatggatggatggatggatgggtgggtggatggatggatggatggatataacattacattctattttctttaaaaatgaattttggttgt*ag*agatttataaacac**tgc**tctgatcaaaatgggaatgaacca**tgc**ctc**tgt**ggg*gt*aaattataagattacaacttttatttatcaagtgttatcataaataatgtataatatcgacactaaacattc*ag*ggtaaaagcata**tgc**aatgaaaaggaaatt**tgt**gacctggaaattaacaaa**tgc**aaaa*gt*aattttcaaattataaaatttatattaattttaactttaaaaaaaaacaatattgttatttattagtgatctatatatttaatacaaggatattgtatgtatatttcgtataaaatctttaattctgaaccgatttcattggaacttgaaacacttgagcacgttaacagtcagtttttttttgtttttttggaacacaaaaccagtacgagttggaaccgaaaagaaaaaaaaggaacgtcataaaggatggatggatggatggatggatggatggatggatggatgatggatggatggatggatggatggatggatggatggatggatggatggatggatggatggatggatggatggatggatataacattacattctgttttctttaaaaatgaattttggttgt*ag*agaattataaacac**tgc**tctgatcaaaatgggaatgaacca**tgc**ctc**tgt**ggg*gt*aaatgataagattacaacttttatgttatcataaataatgtataatatcgtcactgaacattc*ag*ggtaaaagcata**tgc**aataaaaaggaaatt**tgt**gacctgaaaattaacaaa**tgc**aaaa*gt*aattttcaaattataaaatttttattaattttaactttttaaacaaaataaaattgtaatttattattaatctatatatttaaaacaaggatcttatatgtatatttcttatgcaaatctttaatcatgaaccgatttgattgaaacttgaaacacttgagtacgttaactatggggtcagtttagttttataacttaaatttgaatttacttggaaaagaaatcgataaattatttttcgtattattgccaaaaaacctctcctgggtttgttctcacgaaagtgttcgcgcagttttcaaagctggtgatcttaggcgcatgccgattaatattcatgtgcttaaacatttagtttatattataaattttccgttttatattttaaaattcacattttttattatttatctatgatttaatattatattaatattagcattatgttggtagccatcttgtcattcataatttctagccgtgtattcatcgttgcaaataaaagaattgaagtttattaatttgcatatctattaccgggca*ag*gaggaaaacgaagaggcgggaaaacagccagaataa*gt*aatttttaatattaaaatttgcaattaatttcagttcttttattaattttgttttattattaattttatttttgttagttatttttttatgttttaaaaaacaatgttcttattaatttcatttttttgttatcaatttaattttacaaaaataatagtggaattcattattagtattatgcttttaatctggtattaattaatagaggcaaacagtgtctaaagttgaaatttcttttttgactc*ag*aggtg**taaTAG**

**atg**ttgaagctgtttgttgtcctcttggcttcttttggtatcggtctgtctcaag

agatttataaacac**tgc**tctgatcgaaatgggaatgaacca**tgc**ctc**tgt**ggg

gatggctctaaggta**tgt**ggtcgatatgaagat**tgt**gaagaaaacaaa**tgc**aaaa

cgatttttgatccc**tgc**tacattcgagatagtaggagtcca**tgc**ctc**tgt**ggg

gataatcttgta**tgc**aaggacgatgaaatt**tgt**gacatatggaataacaga**tgc**ataa

aaaaatac**tgt**tcattaagaaatgtatggcca**tgc**tat**tgt**gag

gaggatagtatg**tgt**aagaaagatgaaatt**tgt**gaaataggaaagaca**tgt**tatttaggaaaaaga**tgt**gatttgaagtctaaacccagcaaa**tgc**aaatttg

aaaaa**tgc**acgaaaagaaatcgatatcct**tgc**tac**tgt**gtg

aatgactctcaagta**tgt**ggtgcaaatgaagat**tgt**caactaaatcctgagggaaacaaa**tgc**atag

agattagtattcaa**tgc**tctgatcgaaaagggaatgaagca**tgc**ttc**tgt**ggg

aatgaaactata**tgt**tatgaacaagaattt**tgt**gaaggaaacaaa**tgc**aaaa

ttgtagaaaaa**tgc**aagaaaaataatgaatatcct**tgc**tac**tgt**gtg

aatgactcgaaagta**tgt**ggtgaatatgaagag**tgt**aaactgaattctgaaggaaacaga**tgc**acag

agagttacaaaaac**tgc**tctgatccagaaaggaattattat**tgc**cgc**tgt**ggg

aatgaaactgta**tgt**ttgcttcatcaaatt**tgt**gacacaaagacaaacaaa**tgc**aaaa

aaaaatac**tgt**tcggaatcaaataagcatcca**tgc**ttc**tgt**gag

gagaatcgtatc**tgt**tatttatatgaaaaa**tgt**gaactgaatcctgaaggaaacaaa**tgc**atatatg

aaaaa**tgc**acggaaagtaatcaatatcct**tgc**tac**tgt**gtg

aatgactctcaaata**tgt**gctgaaaatgaacgt**tgt**gaactgaattctgaaggaaacaga**tgc**atag

aggtttataaacac**tgc**tctgaacgaaagtggaatgaaaaa**tgc**ctc**tgt**gcg

aatgaatccatg**tgt**tatcgagatgaatat**tgt**gacctgaattatgaaggatacaaa**tgc**acag

agatttataaacac**tgc**tctgatcaaaatgggaatgaacca**tgc**ctc**tgt**ggg

ggtaaaagcata**tgc**aatgaaaaggaaatt**tgt**gacctggaaattaacaaa**tgc**aaaa

agaattataaacac**tgc**tctgatcaaaatgggaatgaacca**tgc**ctc**tgt**ggg

ggtaaaagcata**tgc**aataaaaaggaaatt**tgt**gacctgaaaattaacaaa**tgc**aaaa

gaggaaaacgaagaggcgggaaaacagccagaataa

aggtg**taa**

MLKLFVVLLASFGIGLS

QEIYKH**C**SDRNGNEP**C**L**C**GDGSKV**C**GRYED**C**EENK**C**K

TIFDP**C**YIRDSRSP**C**L**C**GDNLV**C**KDDEI**C**DIWNNR**C**I

KKY**C**SLRNVWP**C**Y**C**EEDSM**C**KKDEI**C**EIGKT**C**YLGKR**C**DLKSKPSK**C**K

FEK**C**TKRNRYP**C**Y**C**VNDSQV**C**GANED**C**QLNPEGNK**C**I

EISIQ**C**SDRKGNEA**C**F**C**GNETI**C**YEQEF**C**EGNK**C**K

IVEK**C**KKNNEYP**C**Y**C**VNDSKV**C**GEYEE**C**KLNSEGNR**C**T

ESYKN**C**SDPERNYY**C**R**C**GNETV**C**LLHQI**C**DTKTNK**C**K

KKY**C**SESNKHP**C**F**C**EENRI**C**YLYEK**C**ELNPEGNK**C**I

YEK**C**TESNQYP**C**Y**C**VNDSQI**C**AENER**C**ELNSEGNR**C**I

EVYKH**C**SERKWNEK**C**L**C**ANESM**C**YRDEY**C**DLNYEGYK**C**T

EIYKH**C**SDQNGNEP**C**L**C**GGKSI**C**NEKEI**C**DLEINK**C**K

KNYKH**C**SDQNGNEP**C**L**C**GGKSI**C**NKKEI**C**DLKINK **C**KRGKRRGGKTARIKV-

Theoretical pI/Mw: **6.90** / 54262.54

**Hnip_mHV3 (this study)**

**chromosome 2 position 19324607 – 19336935 rev+comp**

**atg**ttgaagttgtttgttgtcctcttggcttcttttggtatcggtttgtctcaag*gt*gagtatgatctgagtttctgaacgaaagggtgatatgcaataaatagagcttcaccaatgacctgggatggatgtatggattgatttgatagatggacgcatggatgtaaggatggatagatataacattacatgccatgataaattcacttctgtttattctaaaaatgttgttggttgt*ag*agattaatattcaa**tgc**tctgatcgaaattggaatgaacca**tgc**ctc**tgt**ggg*gt*aaattataagattacaactattatttacaaaatgttatcataaataacatataatttgatattattaatatcttcattgaacattc*ag*aatggctctaaggta**tgt**agtcgatatgaagat**tgt**caaggaaacaaa**tgc**aaag*gt*aatattcatttaatcaaatttacttttaattttgcttatgttattaattttatttttgtcatcaatttcacttttgtttaaaatttatgtttatataaataatatttatttttaatgctgttatgtccaaggaaagaatgataattaataaaaagtgcctcactaatgatttggaatagatgaaaggatgtatgaagggaggcatataacattacatatcgtgataaattcacttctgtttattataaaaatgttgttggttgt*ag*agattaatattcat**tgc**tctgatcgaaattggaatgaacca**tgc**ctc**tgt**ggg*gt*aaatgataagattacaacttttagttatcaattgttatcataaataacgtataatattttcactaaacattc*ag*aatgacactata**tgt**taccgatatgaagat**tgt**gagcagaattctgaaggatacaaa**tgc**aaaa*gt*atttttaatgcaatgaaatttagttttaaatttacaaatgttattaattacatttgtgtgaattgaattccaaggaaagaaatgcatgggtaatttttataagttattaattttacatttttattaatttaaactttgtcatcaattttttaatattaataatttttgtttttattatcaatttcattattttattacatttacacaaatgttaatgtaatttataattaaattgattatgctcaacgaaagagtgatatgtaataaaatgtgggattgatagaatagatgggtggttggattatggaaggatggatggttttgtgcatagatagatagatggatggattgatgtatggatggatggatataacattacataccgtgatatattcacttctgtttacttgataaatgatttttgtttat*ag*agaattttagaccc**tgc**actaatcgatatctgatggatcca**tgc**ctc**tgt**ggg*gt*aaattataaaattttaacgttaatttatcaactgttattatatttatttgtggtttatgagtttatcgaataaaacaaacaagcctttatatacaaaaaaaaaaaacaaatcgcgaaagcactaaataaattgaaataaacccaaagggccatctttagagaagcacacgcttcggctttcggactgaccctgaaagtggctatatacaaattcataagctatacaattccattcaataaatattgtttgcacgatattttgaatagtaaataaagcaattttaatgggcatgtgtttaactgagttagatccttgatagatgaaagtatgaagtctgtttaatataaataagtataatatgatatgatattattaatatcatcattgaacattc*ag*aatggctctaaggta**tgt**agtcgatatgaagat**tgt**gaaggaaacaaa**tgc**aaag*gt*aatatttacttaataaaatttacttttaattttgattatgttattaatgttatttttgtcatcaatttcacttttgtttaaaatttatgtttatataaataatatttattattaatgctgttatgcccaagaaaagaatgataatcaataaaaagtgcctcactaatgatttggaatggatgaaaggcagagatgggttttaactacttaaaaagtagttaactacagtagttaactatttgtagaaaaaagtagttagtaactattttttaactactttttaaataagtagttagtaactacttcttaactacatgcatagtagttaactactttttaactacttttcagttgacatttgataaaattaattttcaggacaactgaacaagacgtaattattagcatatttattcacactagtgtaacgttatcaaaaaattttattgtccaaattaatctaaaaacagtagtgtactgtagatgttttagccgatctcttaaaaaatcatcatgtcgatatgattatccgatagtttacaccgtctgttacatatagggttgccagatgtccagcaaaaggaggacatgtcctcctttttaacctgttgtcctccgtctggcaggctaagttaaaaccctttaaaaagtccggcttttcttatattttaaaggaggcatttatttcaactgaatagaattctgatcaccaccttgtaacatttggcttttataaatgttgaacatataacgtcttgtacaacatcagcattttttacacttttttgccattgatctatcataaagtcgtttgtattacagattatcccttgctttcattgcaagattttttttgttcattttattttaaaatgttattaaaagtagttaactatttgtagttaataacaactttaaaagtaattaattactactttttttaaaagtagttattaactacttgtagttagtaactgctcatctcagatgaaatgatgtatgaagggataacattacaaattgaatgtgatattttcacttctgtttattataaacatgaatgttggttgtagagatttataaactttttatttatggatggatggatggatggatggatggatggatggatggatggatgagtggatggatgggtcgatgggtggataggtgatttggtttgtggttgaatgcatttgtggattgatggatggatggatggatggatataacattacataccctgataaatttacttcagtttattctaaaaatgttgttggttgt*ag*agattcac**tgc**tctgatcgaaatgcgaatgatccattt**tgc**ctt**tgt**ggg*gt*aaatgataagattacaacttttagttatcaattgttatcataaataacgtataatattttcattgaacattc*ag*aatgacactata**tgt**tatcgatatgaagat**tgt**gagcagaattctgaaggatacaaa**tgc**aaaa*gt*atttttaatgcaatgaaatttagttttaagtttataaatgttattaattacatttttgtgaattgaattccaaggaaataaatgcatgggtaatttttataagtttttaattttatatttttattaatttaaattttgtcatcaattttttaattttaataatttttgtttttattatcaatttcattattttattacatttacacaaatgttaatgtaatttataattaacttgattatgctcaacgaaagagtgatatgtaataaaaatgtgggattgatgaatggatggatggttggattatggaaggatggatggttttgtgcatagatagatagatggatggattgatgtatggatggatggatataacattacataccgtgatatattcacttctgtttacttgataaatgatttttgtttat*ag*agaattttagaccc**tgc**actaatcgatatctgatggatcca**tgc**ctc**tgt**ggg*gt*aaattataacattttaacgttaatttatcaactgttattatatttatttgtggtttatgagtttattgaataaaacaaacaagccgttatataaaaaaaaaaaacaaatcgcaaaagcactaaatactttcaaataaacccaaagggccagccttacggaagcacacgcttcatctgtcggactgaccctgaaagtggctatatacaaattcataagctatataattccattcaataaatattgtttgcacgatattttgaatagtaaataaagcaattttaatgggcatgtgtttaactgagttagatccttgatagatgaaggtatgaagtctgtttaatataaataagtataatatgatatgatattattaatatcatcattgaacattc*ag*gatggctcaaaggta**tgt**agtcagtatgaagtt**tgt**gaagaaaacgaa**tgc**atag*gt*aatatttatgtaataaaatttacttttaattttgtttatgtttttaattttatttttatcattaattccatttttttaatttttatttttacataaataatattgtaatttattgttgtttattacataaaaatttactttcatataaataatattgttatattatattaatgctgttatgctcaacgaaagtgtgatacttaggaaaaagtgcttcactaaagatttggattttggaatggatggatggatggatggatggatggatggacggatgtttataacattacataccgtgatatattcacttctgtttattttcatattgaatgttggttgt*ag*agattgatgtaaac**tgc**actgatagaaatgcgagagaacca**tgc**ttc**tgt**ggg*gt*aaattacaaaattacaactttacaatttatcaactgttatcatatgtttttgtggttgatgggtttattgaataaaacaagcatgccgttatatacccagaaaaagcaaaacgtgtaagcacttaataaattcaaataaacccaaagccatctttagagaagcacacgcttcggctttcggactgaccctgaaagtggctatatacaaaatcataagctatataattccattcaataaatattgtttgcacgatattttaaatagtaaatagagcaatttaaaatggcatgtgtttaagtcataagatccttgattgatgaaggtatggaggagtaaagtctgtataacataaacaacgtatagtatgatatgatattattaatatcatcattgaacattc*ag*gatggctctaaggta**tgt**agtcgatatgaatat**tgt**gatgaaaacaca**tgc**aaag*gt*aatttttatgtattaaaatttactattaattttgcttaggttattaattttatttttgttatcaatttcttttttttaaattttatttctattttatattattgtattttattattaatactgttatgctcaacgatagattgatacttaacaaaaagtgccttatcagaaatttggaatagatggatggatggatggatgcagtgttatttccatttgccaaataatttccattacaattcaaattccattattatttgacaaattcttttttgtgttctaattccattcaaagttctaaaaaattccattcaattaccattgctgaaataatttataatttatagttgactgatataaattagcttagctggattagaaaatttaatcataatattatttgttacctaagtttgagataaaccgaattggcttcagaaataatttctatttctcattctaattccattttacaaaataaattctacttctcattctatttccatttttcgggcaatttcaattcctattcctaattccattaccaaattttaatttccgttttcgagaaaaaataatttctatttcaatttctatttcaattaccattaatggaaatatcactggatggatggatacataccgtgatattttctcttatgtttattataaaaatgaatgctggttgt*ag*tgtatagaagaaga**tgc**tctgaa**tgt**aataagtggcca**tgc**ctc**tgc**aag*gt*aagttataagattacaacttttaattatcagatgttatcataaataacatataatatcatcattgaacattc*ag*ggtaatcatgta**tgt**ggttattatgaaatt**tgt**gaactaggagagattaacaaa**tgc**aaaa*gt*aattttcgtgtaataacatttgttaataatgtttttttattcatgttattttttataaaaaaaattaatattgtaatttattgttaatactatggtcttaatatttacatcaaaaatgaaacttgattgt*ag*aaaaatac**tgt**tcggaaataaatgaggaatat**tgc**ctc**tgt**gag*gt*aaattataagataataacttatatttaccaaatgttatcataaataatatataatatcatcattgaacattc*ag*aatgaaactgta**tgc**gattctgatgaaatt**tgt**gatataaagacaaacaaa**tgc**aaaa*gt*aattttcataaaaataaaatttgctattactgttttttattaattttatttttacgaaaataagattgtaatttattattgatactatgatcttaatatttataataaaaatgaatgttggttgt*ag*aaaaatac**tgt**tcggaagaatcaaatgagtggcca**tgc**cac**tgt**ggg*gt*aaattataagataatgacttttacttatcagatgttatcataaataacgtatactatcatcattgaacattc*ag*aatgatagtatc**tgt**tattcaaataaaaaa**tgt**gaactgaatcctgaaggaaacaaa**tgc**atatttg*gt*aattttttataatgaaatttaataataattttacttatattattaattctatttttattattaatttcaaattttatttttgtaaaaataatattacaatttattattatttaatttatattacgctcttaatatttatattaaaaatgaatgttggttgt*ag*aaaaa**tgc**acggaacgtaatcgatatcca**tgc**ttc**tgt**gtg*gt*aaattataaaataataactttcagttatcaaatgttatcataaataacgtataatatcatcatgttatcatatatagcgtataatatcatcatgttatcataaataacgtataatatcatcatgttatcataaataacgtataatatcatcatgttatcatatatagcgtataatatcatcatgttatcataattaacgtataatatcatcatgttatcataaataacatataatatcatcatgttatcataaataacgtataatatcatcatgttatcataaataacgtataatatcatcatgttatcataaataacgtataatatcaccatcgaactttc*ag*aatgactctcaaata**tgt**ggtgaaaatcaagag**tgt**gaactgaattctgaaggaaacaga**tgc**atag*gt*aatttttaaaaagttgttaattttatatttttattaaatttaattatgttattaattattttatgttattatttttatttctattatgaataatattgtaatttattgttaatactattaacctcaacaaaaaaaaagagcctcaccaataatttaggagggaggtagtgatgtaaggatgtaaggatggatggatggatgtaacattacataccgtgatgataaattcacttctgtttattgcaaaaatgatattggttgc*ag*aaatctttgaagtc**tgt**tctgatcgaaatgattggcca**tgc**cgc**tgt**ggg*gt*aagttataaaataacaacttttagttatcaatcgttatcataaataacgtataatatcatcgttgaatattc*ag*aatgaaactcta**tgt**tataaacatcaatat**tgt**gacctgaattctgaaggagagaaa**tgc**aaaa*gt*aatttttgtgcaatgacatttacaaatgatacaaatttaattttttttattaatttcattttattaaatttacacaaataatattataatttattattaatactattatgctcagtgaaagagtgacatgcaaaactgatgtggattgatggatggatggatggatggatggatggatggatggatggatggatggatggatggatggatggatggatggatataacatttcataccctgatattcacttctgtttattttaaatatgaatgttggttgt*ag*agattaattttagtctc**tgc**tctgaacgtaatgtgtatcca**tgc**cgc**tgt**gat*gt*aagttataagattacaacttttatttatcaaatgttgtcataaataacgtataatattttcactgaacattc*ag*ggtgataatata**tgc**aatgaaaatgaatta**tgt**gacctgggaattaacaaa**tgc**aaaa*gt*aattttcatttaataaaatttttcattattttttaaaaattgaaattaataaaaaatagcattgtaatttaatattaataccatgctcttaattatttataataaaaatgaatgttcattgt*ag*aaaaatac**tgt**tcggaaataaatgagcagcca**tgc**ttc**tgt**gag*gt*aaattataagataataacttttacttatcagatgtcatcataaataatgtataatatcgtcattgaacattc*ag*ggtgaacgtacc**tgt**gacttgcttgaaact**tgt**gaattcaatcgtaaaagaaacaga**tgc**aaac*gt*aatttttatgtattaaatttcactattgatttcgaatatgctaataattctatttattatacttttagcattaattttttttttactaattgt*ag*ttagaaaa**tgc**tcggaagaaaatggatatcct**tgc**cgt**tgt**gtg*gt*aaatttgaagataataacttttatttatcaaatgttatcgtaaataacatataatatcttcattgaacattc*ag*gagaatcgtgtc**tgt**cagtattatgaaaaa**tgt**gaactgaattctgaaggaaacaaa**tgc**ataacac*gt*aatttttataagttgtttggtatttatgaaacttaataataattttactcgcattatcaattttatttttattattaatttcaatttttgttattaattcttttttacaaaaaaatatcataatttattaataattaatttatattatgttcttattttttatattaaatacgaatgttggttgt*ag*aaaaa**tgc**gaggatagagatggatatcca**tgc**cgt**tgt**gtg*gt*aaatctaaaaatacaaacttttagttatgaaatgttatcataaatatcgtataatatcatcatgttatcataaatatcgtataatatcatcatgttatcataaatatcgtataatatcatcatgttatcataaatatcgtataatatcatcatgttatcataaatatcgtataatatcatcatgttatcataaatatcgtataatatcatcatgttatcataaatatcgtataatatcatcatgttatcataaatatcgtataatatcatcatgttatcataaatatcgtataatatcatcatgttatcataaatatcgtataatatcatcatgtcatcataaatatcgtataatatcatcatgtcatcataaatatcgtataatatcatcatgttatcataaatatcgtataatatcatcatgttatcataaatatcgtataatatcatcatgttatcataaatatcgtataatatcatcatgtcatcataaatatcgtataatatcatcatgttatcataaatatcgtataatatcatcatgttatcataaatatcgtataatatcatcatgttatcataaatatcgtataatatcatcatgttatcataaatatcgtataatatcatcatgttatcataaataacgtataatatcatcatgttatcataaatatcgtataatatcatcatgttatcataaatatcgtataatatcatcatgttatcataaatatcgtataatatcatcatgttatcataaatatcgtataatatcatcatgttatcataaatatcgtaaaatatcatcatgttatcataaatatcgtataatatcatcatgtcatcataaatatcgtataatatcatcatgttatcataaatatcgtataatatcatcatgtcatcataaatatcgtataatatcatcatgttatcataaataacgtataatatcaccatcgaactttc*ag*aatgactctcaaata**tgt**gcttataatgaacgt**tgt**gaactgaattctgaaggaaacaaa**tgc**atag*gt*aatttttataagttattaattttatatttttattaatatcatttttaattattttatttttttaattaaatttattttttacataaatattattgtaatttattattaatttattattaatactaaataaagtgatatgtaagaagaaaaaaagtggatttgggatggatggatggatggatggatggatggatggatggatggatggatggatggatggatggatggatggatggatggatggatggatggatggatggatggatggatggatggatggatggatggatggatggatggatggatggatggatggatggatggatggatggatggatggatggatggatggatggatggatggatggatggatggatggatggatggatggatggatggatggatggatggatggatggatggatggatggatggatggatggatggatggatggatggatggatggatggatggatggatggatggatggatggatggatggatggatggatggatggatggatggatggatggatggatggatggatggatggatggatggatggatggatggatggatggatggatggatggatggatggatggatggatggatggatggatggatggatataaattacttctgtttattttaaaaatgaatgttggttgt*ag*aggtttataaaacc**tgc**tctaaacgaaagtggaatgaaaaa**tgc**ttc**tgt**ggg*gt*aaattacaagattacaacttctatttatcaattgttttcataaataacattaatatcgtcattgatcattt*ag*aaaaaagccata**tgt**tctccaaaggaaatt**tgt**gaactgaattgtgatggacacgaa**tgc**aaaa*gt*aatttttatgcaataacatttgctattcattttacaatggttattaattgtatttttattatctttttttttttattaaatttacacaaacattattttaattttgtatcaaatctattatgctcaacgaaagagtgatatgtaatgaaacgtgctgaaaactgatgagggatggatggatggatgcatttttttttttttttgaaacacaaaaccagtactagttggagccgaaagtaaaaaaaggaacgtcataaaggaaggatggatggatggatggaaggaaggatggatggatggatacattacataccataataagttcacttctgtttattttaaacatgaatgttggttgt*ag*agatttataaacac**tgc**tctgatcaaaatgggaatgaacca**tgc**ctc**tgt**ggg*gt*aaatgataagattaaaacttttatgttatcataaataacgtataatatcgtcactgaacattc*ag*ggtgaaagcata**tgc**aatgaaaaggaaatt**tgt**gacctgaaaattaacaaa**tgc**aaaa*gt*aattttcaaattataaaatttttattaattttaactttttaaacaaaataaaattgtaatttattattaatctatacatttaaaacaaggatcttatatgtatatttcttatgcaaatctttaatcctgaaccgatttgattgaagcttgaaacacttgagtacgttaactatggggtcagtttagttttataacttaaatttgaatttacttggaaaagaaatcgataaattattgttcgcattattgccaaaaaacctctcctgggttctcacgagagcgttcgcgcagttttcaaagctggtgatcttaggcgcatgccgattaatattcatgtgcttaaacatttagtttatattataaattttccgttttatattttaaaattcacattttttattatttatctatgatttattattatattaatatt*ag*cacaaagttg*gt*agccatcttgtcattcataatttctagccgtgtattcatcgttgcaaataaaagaatttaagattattaatttgcatatctattaccgggca*ag*gaagaaaacgaagaggcgggaaaacagccagaataa*gt*aatttttaatattaaaatttgcaattaatttcagttcttttattaattttgttttattattaattttatttttgttagttatttttttatgttttaaaaaacaatgtttttaataatttcatttttttgttatcaatttagttttacaaaaataataatgtaattcattattagtattatgcttttaatctggtattaattaatagaggcaaacagtgtctaaagttgaaatttcttttttgactc*ag*aggtgtaatagctggagctcaggagattcacaaaaaggaatcaaca**taa**

**atg**ttgaagttgtttgttgtcctcttggcttcttttggtatcggtttgtctcaag

agattaatattcaa**tgc**tctgatcgaaattggaatgaacca**tgc**ctc**tgt**ggg

aatggctctaaggta**tgt**agtcgatatgaagat**tgt**caaggaaacaaa**tgc**aaag

agattaatattcat**tgc**tctgatcgaaattggaatgaacca**tgc**ctc**tgt**ggg

aatgacactata**tgt**taccgatatgaagat**tgt**gagcagaattctgaaggatacaaa**tgc**aaaa

agaattttagaccc**tgc**actaatcgatatctgatggatcca**tgc**ctc**tgt**ggg

aatggctctaaggta**tgt**agtcgatatgaagat**tgt**gaaggaaacaaa**tgc**aaag

agattcac**tgc**tctgatcgaaatgcgaatgatccattt**tgc**ctt**tgt**ggg

aatgacactata**tgt**tatcgatatgaagat**tgt**gagcagaattctgaaggatacaaa**tgc**aaaa

agaattttagaccc**tgc**actaatcgatatctgatggatcca**tgc**ctc**tgt**ggg

gatggctcaaaggta**tgt**agtcagtatgaagtt**tgt**gaagaaaacgaa**tgc**atag

agattgatgtaaac**tgc**actgatagaaatgcgagagaacca**tgc**ttc**tgt**ggg

gatggctctaaggta**tgt**agtcgatatgaatat**tgt**gatgaaaacaca**tgc**aaag

tgtatagaagaaga**tgc**tctgaa**tgt**aataagtggcca**tgc**ctc**tgc**aag

ggtaatcatgta**tgt**ggttattatgaaatt**tgt**gaactaggagagattaacaaa**tgc**aaaa

aaaaatac**tgt**tcggaaataaatgaggaatat**tgc**ctc**tgt**gag

aatgaaactgta**tgc**gattctgatgaaatt**tgt**gatataaagacaaacaaa**tgc**aaaa

aaaaatac**tgt**tcggaagaatcaaatgagtggcca**tgc**cac**tgt**ggg

aatgatagtatc**tgt**tattcaaataaaaaa**tgt**gaactgaatcctgaaggaaacaaa**tgc**atatttg

aaaaa**tgc**acggaacgtaatcgatatcca**tgc**ttc**tgt**gtg

aatgactctcaaata**tgt**ggtgaaaatcaagag**tgt**gaactgaattctgaaggaaacaga**tgc**atag

aaatctttgaagtc**tgt**tctgatcgaaatgattggcca**tgc**cgc**tgt**ggg

aatgaaactcta**tgt**tataaacatcaatat**tgt**gacctgaattctgaaggagagaaa**tgc**aaaa

agattaattttagtctc**tgc**tctgaacgtaatgtgtatcca**tgc**cgc**tgt**gat

ggtgataatata**tgc**aatgaaaatgaatta**tgt**gacctgggaattaacaaa**tgc**aaaa

aaaaatac**tgt**tcggaaataaatgagcagcca**tgc**ttc**tgt**gag

ggtgaacgtacc**tgt**gacttgcttgaaact**tgt**gaattcaatcgtaaaagaaacaga**tgc**aaac

ttagaaaa**tgc**tcggaagaaaatggatatcct**tgc**cgt**tgt**gtg

gagaatcgtgtc**tgt**cagtattatgaaaaa**tgt**gaactgaattctgaaggaaacaaa**tgc**ataacac

aaaaa**tgc**gaggatagagatggatatcca**tgc**cgt**tgt**gtg

aatgactctcaaata**tgt**gcttataatgaacgt**tgt**gaactgaattctgaaggaaacaaa**tgc**atag

agatttataaacac**tgc**tctgatcaaaatgggaatgaacca**tgc**ctc**tgt**ggg

ggtgaaagcata**tgc**aatgaaaaggaaatt**tgt**gacctgaaaattaacaaa**tgc**aaaa

cacaaagttg

gaagaaaacgaagaggcgggaaaacagccagaataa

AGgtgtaatagctggagctcaggagattcacaaaaaggaatcaaca**taa**

MLKLFVVLLASFGIGLS

QEINIQ**C**SDRNWNEP**C**L**C**GNGSKV**C**SRYED**C**QGNK**C**K

EINIH**C**SDRNWNEP**C**L**C**GNDTI**C**YRYED**C**EQNSEGYK**C**K

KNFRP**C**TNRYLMDP**C**L**C**GNGSKV**C**SRYED**C**EGNK**C**K

EIH**C**SDRNANDPF**C**L**C**GNDTI**C**YRYED**C**EQNSEGYK**C**K

KNFRP**C**TNRYLMDP**C**L**C**GDGSKV**C**SQYEV**C**EENE**C**I

EIDVN**C**TDRNAREP**C**F**C**GDGSKV**C**SRYEY**C**DENT**C**K

VYRRR**C**SE**C**NKWP**C**L**C**KGNHV**C**GYYEI**C**ELGEINK**C**K

KKY**C**SEINEEY**C**L**C**ENETV**C**DSDEI**C**DIKTNK**C**K

KKY**C**SEESNEWP**C**H**C**GNDSI**C**YSNKK**C**ELNPEGNK**C**I

FEK**C**TERNRYP**C**F**C**VNDSQI**C**GENQE**C**ELNSEGNR**C**I

EIFEV**C**SDRNDWP**C**R**C**GNETL**C**YKHQY**C**DLNSEGEK**C**K

KINFSL**C**SERNVYP**C**R**C**DGDNI**C**NENEL**C**DLGINK**C**K

KKY**C**SEINEQP**C**F**C**EGERT**C**DLLET**C**EFNRKRNR**C**K

LRK**C**SEENGYP**C**R**C**VENRV**C**QYYEK**C**ELNSEGNK**C**I

TQK**C**EDRDGYP**C**R**C**VNDSQI**C**AYNER**C**ELNSEGNK**C**I

EIYKH**C**SDQNGNEP**C**L**C**GGESI**C**NEKEI**C**DLKINK**C**K

TQSWKKTKRRENSQNKGVIAGAQEIHKKEST-

Theoretical pI/Mw: **5.30** / 71774.46

**Hnip_mHV4 (this study)**

**chromosome 2 position 19341815 – 19344105 rev+comp**

**atg**ttgaagctgtttgttgtcctcttggcttcttttggtatctgtctgtctcaagaattag*gt*gagtgtgatatgagtttctcaacgataaagttatatgtaataaaaagtgatatgggattgatggatggatagatggaaggaaggatggatggatggttggaaggaatgaaggatggatggatggatagaacatctcgtaccagggtacattttttcatcaatcaattttattttgtactttcacaaaaagttcacttctatttatattaaaaatgaatgttgattgt*ag*agcgc**tgc**tcagaaggtaatagaactaat**tgc**cga**tgt**gag*gt*taattataagaatataacttttatttatcaaatgttatcataactaacgtataataatatcgtcaatc*ag*ggtgactctacc**tgt**ggtccatatgaaata**tgt**atgctgaattcggaaggaaacaaa**tgc**aaaaataatttttat**taa**ttttattaattttaactttgttattaattttaattttactaaatataatttgtaatttattattaagaaaattatgtgatagaaggtgcttcaccagtgatgtgggattgatcgatagatggatggctatgcatatggcgctatataaatgaagttacaataaattcacttcaattttaattcaattttaaaaatgaatgctggttgt*ag*agatttataaatac**tgc**tctgatcgaaatgggaatgaacca**tgc**atc**tgt**ggg*gt*aaattataatattacaactttaatttatcaactgttatcataaacaatgaatgatacgatattattaatatcttcattgaacattc*ag*gatgactctaattta**tgt**aataaaaatgaaaat**tgt**gaaggaagcata**tgc**aaaa*gt*aatttttatttaataaaagttattattaatttttctaatgttattaattttttttttttttaaatttcattttacataaataatattgtaatttattataaatgctgttatgctcaacgaaatagtgatatgtaataaaacgtgcttcacagtgacttgggatggatggattgatggatggatggatggatggatggatcgattgatggatgaatgggtggatggatataacattacataccgtgataaattcacttctgtttattttaaaaatgaatgttggttgt*ag*gtattcataaacgc**tgc**tatggtcataatgtgtggcca**tgc**ctc**tgt**ggg*gt*aaatgataagttacaacttatatttaccaaatgttatcataaataacgtataatatcataattaaacattc*ag*gatcatgta**tgt**cgtttagatgaacat**tgt**gaaacgggt**ttactagga**gatattaacaaa**tgc**aaaa*gt*aattttcttatattaaaatctgttatcaatttttttattttttaacatttttttgttaataattttatttttacaaatatattattgttgt*ag*aaaaatca**tgt**tcggaaaaaaatagttatcca**tgt**cgt**tgt**atg*gt*aaattataagttaataacttttagttaccaaatgttatcataaataacttataatatcatcatttgactttc*ag*ggtgactcctac**tgt**caaacatatgaaatt**tgt**gaagtgaattctgaaggaaacaaa**tgc**ataatgc*gt*aattttttataataaattaattaattaattattttacaattaattttacatattttattaatttaatttttgtttttatttttttctggttattaattttatttttctataaaatataataccgtaatttattattaatacttgatattattattaatattaaaaattaattttggttgt*ag*aaaaattc**tgc**tcgttaaataatgaatatcct**tgc**atc**tgt**gtg*gt*aaatttgaagatactaacttttatttatcagatgttatcataaataacttataatatcatcattgaacgttc*ag*aataatcaggtc**tgt**ggtcaacttgaaact**tgt**gaagtgaatcctcaaggaaacaaa**tgc**ataa*gt*aatttataaaaaataatttactattaattttacttattttattaattttatttttattattagtatttttgttagtaatattttttatgttattaattatactaattttatttttacaaaaataatattataattcattactaatattatgctctaaatctgatatttattaatagaggcaaacagtgtctaaagttgaaatttatttttttactc*ag*gtgcaatagctggagctcaggagattcacaaaatggaatcatca**taa**

**splice variant 1:**

**atg**ttgaagctgtttgttgtcctcttggcttcttttggtatctgtctgtctcaagaattag

agcgc**tgc**tcagaaggtaatagaactaat**tgc**cga**tgt**gag

ggtgactctacc**tgt**ggtccatatgaaata**tgt**atgctgaattcggaaggaaacaaa**tgc**aaaaataatttttat**taa**

MLKLFVVLLASFGICLSQELER**C**SEGNRTN**C**R**C**EGDST**C**GPYEI**C**MLNSEGNK**C**KNNFY-

QELER**C**SEGNRTN**C**R**C**EGDST**C**GPYEI**C**MLNSEGNK**C**KNNFY

Theoretical pI/Mw: **4.82** / 4797.26

**Hnip_HV7** **(this study)**

**splice variant 2:**

**atg**ttgaagctgtttgttgtcctcttggcttcttttggtatctgtctgtctcaagaattag

agatttataaatac**tgc**tctgatcgaaatgggaatgaacca**tgc**atc**tgt**ggg

gatgactctaattta**tgt**aataaaaatgaaaat**tgt**gaaggaagcata**tgc**aaaa

gtattcataaacgc**tgc**tatggtcataatgtgtggcca**tgc**ctc**tgt**ggg

gatcatgta**tgt**cgtttagatgaacat**tgt**gaaacgggt**ttactagga**gatattaacaaa**tgc**aaaa

aaaaatca**tgt**tcggaaaaaaatagttatcca**tgt**cgt**tgt**atg

ggtgactcctac**tgt**caaacatatgaaatt**tgt**gaagtgaattctgaaggaaacaaa**tgc**ataatgc

aaaaattc**tgc**tcgttaaataatgaatatcct**tgc**atc**tgt**gtg

aataatcaggtc**tgt**ggtcaacttgaaact**tgt**gaagtgaatcctcaaggaaacaaa**tgc**ataa

gtgcaatagctggagctcaggagattcacaaaatggaatcatca**taa**

MLKLFVVLLASFGICLS

QELEIYKY**C**SDRNGNEP**C**I**C**GDDSNL**C**NKNEN**C**EGSI**C**K

SIHKR**C**YGHNVWP**C**L**C**GDHV**C**RLDEH**C**ETGLLGDINK**C**K

KKS**C**SEKNSYP**C**R**C**MGDSY**C**QTYEI**C**EVNSEGNK**C**I

MQKF**C**SLNNEYP**C**I**C**VNNQV**C**GQLET**C**EVNPQGNK **C**ISAIAGAQEIHKMESS-

Theoretical pI/Mw: **5.38** / 18611.01

**Hnip_mHV5 (this study)**

**chromosome 2 position 19349205 - 19355228 rev+comp**

**atg**ctgaagctgtttgtcctcctcttggctgttttcatctgcgtgtctcgatcat*gt*gagttttaatttgagcttctcaatgaaagtgatatctaataagaagagctactatatcagtggaagatggatggatggatggatcgattggtgttatatcacataccatcaagggcggatttaggggcggggagccatggccctccccgtcggttggaagtgacaatataagaacaaactaagtgaacctatttgtaataattaatcaaaaatttattttaatttattatttgttaaattagtttgataacttaccaagtttgacgaaaattttagtagctgtacccaacccctcccctaaatccgcccactggttcctgccctctcccgccaaagtgttatagatccgcccttgctaccagtgtacactcacttctgttagcattagaaatgaatattggttgt*ag*taaggtatctacca**tgc**tcacagaataacaagactcca**tgc**ctt**tgt**aag*gt*aaattcttagattacaacttttattttctaaatgttatcataaatagcgtataacatcgtcatcgaatattc*ag*aatgaaaaagagctg**tgt**cctgctgatttcact**tgt**caactgaattctaagggaaacaga**tgc**atgagtaaag*gt*aaatttttttttattaattttcacttttttttacatcgtggtatattgcatattaattttattttatttcgaacaatttggtcaaagggcaaaggtcacacatgggtttaccccatacccctcgagcttaacgtactttatggttaaacaacgcctttaacaaaggctagcgacgctatttctgaattcttgaatactccttagaaaagcttctctcatatttaaaatagcaaatgtcagttgaagagattttttggcgatgtttcagcagacttggaaaatgggttattgtttctaatttaaaatcttgagcaaaaatggataattgaccgagtagtaccgagtacaaagtagttctttcgagaattgcttgagtcttttactgcaagtcaggccggcctattgggtagaatttctacatgcgacgataataataacaatcgttatgtgtcgcgttggcaacccagcctgcactgccagagatcgcccttgggtaggtatgccgtgtaaatggcgggtcgattaagaatattccatcacctttacatcatgctcggaaacataaggcattcaaaaaaataagatatgtacaaaaaatactaacaagcttatgtaagtatcgaagtttaagctgtgacttgctcgctcttgctcagctcgagttattacgtaactacgcgctctgcatgctacatcagttctgccgacacctgacttgccagtcaaaaccgatttgtgaacgactggccgagccgcactcagatcgctaaggagccgcgatttgccgaccactgaactagaaggtagcttgcataaatttaaaataagatcgtagtaccaaaagaaatacccagagggtgcagaatgccatcccaaggaacaaatgaggattattatgtattttgttgttgagtatcagattctagaagacttttactgtatttacttctgtttatgttaaaaatgaatgctggttgt*ag*ttaaaccc**tgt**tcgaaaggaaatacatggcca**tgc**ctc**tgt**gag*gt*aaattataagacagtaacttttatttaccaaatgttatcgcaaataacgtataatatcatcattgaacattc*ag*aacgaaaacaagatc**tgt**cgtcctggtcatatt**tgt**caaatggaacctagtgggaacaga**tgc**attaagagaa*gt*aatttttatataccataatttcacgtagataacacagatataagttaatatagcggacgaatttagctcgcaatttttccgatttgtgtaaaaaatcattaagatattaaagacattctctacaataaggctacgtgacatctgcatattaaagtgttaaacagcacccaggaattccccctgagggcagcttgccctccctgaaataagtagaataaactgaaataagttggaaaaattcggattcaaaattcacatgctgttgtgataatttctcgaaggtttaggatttcaaattacaaaaattattttaattaaaatattgctcccagacttctatctctctcggtattaaattaaattctagatggatgcacaagatcttgaaaagagaatctgtgaacacctccttagaagccattgcggaagtactttatgaaatttcgtatcaaaagtgttatgacttaacaactaaatctaatgctgtggctttatagaaacgtatcatgagctttgactttattttgtctgttatgtctatgtacatagtaaaagtgttaactgaatcgttggaaaaattggcctgagtgttattgattttggtagaaaacataaaagcgctcactaagcgaccttgaaattcctcaacattgaaaccgagatgacacaaattaatccagactggaaaccaattttcattcaaattacacataaatgcggaagactattttaagcgtcgtcacagacatagagtggcgatttgatgacaaagctgaatctcattctttgggcgggagtttaaggaagttctggatccactaataaactatgtgaaatcgaacgtgttgcagtgtgtaaatcagtggtgcacaatcttttttggccgagggctgatatcatcaacatcaagacaacacaaacaataagacacatgtacagacgaataaagacaaagttgtggactagatgggctgatgttcatatcttgacagttttaagttaatgatataacgtggcagctgaggctgcatgtctgcaacaagctttgaaatatcaggctgttcgtagcttgcagaacgatggaaaataatcgaacagcaaaaatggcggaggctgtggaggtccagtgagtcgaggtgtatcacgacgccctctaagatgattggcgaggcatagctgtcacgccatggaactagtcagtaactcggctgtttgttagtccagtgagaaagaatttttttaaatttagagatgtacgtctctgtgcttactcctccacaaccttttgaaaattgactgaagaacctatttgtccactttttcttgtcagccttcgcggtccacttgacggctggtcgagtgcctgatgtggccgccggtcggaggttgtcgtataaaagataaattttatgtttattaaacatgtagaaagacattgaaagattttaaagatttttgcgctattatattttaccaatgtttttgagctggtcataatagttcctattaacgagcaataaatatcgacaaaataataagtaaacagtttttttgagtttgaaaaaactgtcctttatgcccccctccccccactattttgccttcggtacgtccttgacagcaccaaccaaggtatggtgtcttctcttacagcagtgttgtgaaaacactattacatctcaaactgtaaacactaacaccaattataattattcttgtcataattattattaaatgtctgctcttaaggatcaggagctagaggacttttactgttttcacttctgtttatattaatagtgaatattggttgc*ag*ttaaactc**tgc**tcggaaggtaatgtcactcca**tgt**ctc**tgt**gag*gt*aaattataacttttacctatcataaataacgcataatatcatcattgaacattc*ag*gatgataaaaaaata**tgt**cgttctggttactat**tgt**caactggattctaatggaaacaga**tgc**ataaacaaaa*gt*aattcttttatagtaaaatattgtattttattagtgatactgtttttatatgagacaatattgtggtttattattagtactattttacatgatataatattgtatatttattttattttattttatttcaaaaaaaatgttcagaggacaccttttttggtcatcgtttaaattgtttctctcaagttacaataataactatttaatcatcgtcatcagcatcgtccctttgtcaaatactacaatagaaatagaagatttcaaattttatctattttctcattttacattctatgcatgcttacaccgtaaataagattttttagacatttgttgactttaacatttaaacggcaatcttggccattaaatggccaattacggtgacctttatgcgggccgcgtgtgtgcttgcaaagggccgcatgagtgtttgacatgcctgctctagatgacttactttattcagttctgtttatattaaatatgtgtattggttgc*ag*ttaaaccc**tgc**tcagaaagtaatgtgctacca**tgc**ctc**tgt**gag*gt*aaattataagtttataactgttatttatcaaatgctttcataaataaagtataatatcaccattgaacatac*ag*aataaagacaaacaa**tgt**cgtactggttactat**tgt**cttcagaattctaccggatacaat**tgc**ataaacttaa*gt*aatttttacataataaaatattgtatatttattttattttatttcaaaaaaatgggtcaaagatgattttaaaagcgcctctaatatgaccaaaaagaaagacaatcactgaagaataacgaaagctgcgccgagcgaaaaagggaagggcagaaatgaaacagcagagtaaatcatcgaatttcttgaagctcaatcatcaacaatcaaaaagcgggagaggtcagcatgcgaaaccaaagttttatgacgtgaaaaagtaccagctgacaatgtcaaagggagcgagttccagatttctataagtgccattttttacattgttatgtctacgatagcctttggtgtctgggtttcacagaaagtacacttatacatccgtaatcagtgaactgattaacactacgtttgttgttggcaaaaatttgaggaagttttaaaacagcaacaagtaagcaacaggtaagatgacatcaccgacatatggagcgagttccagatttttgtaacccgatgtatccatttaatagacgtatgcatgtaatgcatgtatccatttgatagatgtatccatgtaatacgtgtatccaagtatcctaaccgtacttcagcgtacgttttaacacccatccataactccttgacacaaaataaagggacagaaatgaataatattgttaaatacagtcaggaccttgacaattactaactatctagccatcgtcatcagcatcgtccctctgtcaatagaaacagtcgacttcaagtttaatttactatctattcatgtttattgtaatatttaatattaaataataataatataataacataatgttatataatatttaatacgtttaacaatttcatctcaatttacaatttattattaatattttaaaaaaaaagtaagttatatcaatatatttaaaattattattaaaccaatatttccaaattgaaacgaatttcaatataaaatagttaaactcaaataatatattatttatgaattgattaaatttcaacggtaaaacatgaatgtcctttgt*ag*ttaaaccc**tgt**tcggaaaacaatgagtggcct**tgc**ctc**tgt**gtg*gt*aaattggaagataatagcttttatttatcaaatgctatcataagtaacctgtaatatcaccattga*ag*atgaagaatgatggacaagaattacgttca**tgt**tggtttgatgaaact**tgt**aaactgaattctaatggaaacaaa**tgc**ataa*gt*aatt*gt*tctaaatgttaaaaatattaattttatttttacttaacaaataattaattttattatcctattaatctgttattatttgatagaaacagaccgtgactaaagctgacatttaatgaaattctatttattacttagagataaacaagataaaaaactcttttgagctacataatgaaatattaattattaaatataatattcaaatgtcatttcgac*ag*gtaaaggcaggaga**tga**

**atg**ctgaagctgtttgtcctcctcttggctgttttcatctgcgtgtctcgatcat

taaggtatctacca**tgc**tcacagaataacaagactcca**tgc**ctt**tgt**aag

aatgaaaaagagctg**tgt**cctgctgatttcact**tgt**caactgaattctaagggaaacaga**tgc**atgagtaaag

ttaaaccc**tgt**tcgaaaggaaatacatggcca**tgc**ctc**tgt**gag

aacgaaaacaagatc**tgt**cgtcctggtcatatt**tgt**caaatggaacctagtgggaacaga**tgc**attaagagaa

ttaaactc**tgc**tcggaaggtaatgtcactcca**tgt**ctc**tgt**gag

gatgataaaaaaata**tgt**cgttctggttactat**tgt**caactggattctaatggaaacaga**tgc**ataaacaaaa

ttaaaccc**tgc**tcagaaagtaatgtgctacca**tgc**ctc**tgt**gag

aataaagacaaacaa**tgt**cgtactggttactat**tgt**cttcagaattctaccggatacaat**tgc**ataaacttaa

ttaaaccc**tgt**tcggaaaacaatgagtggcct**tgc**ctc**tgt**gtg

atgaagaatgatggacaagaattacgttca**tgt**tggtttgatgaaact**tgt**aaactgaattctaatggaaacaaa**tgc**ataa

gtaaaggcaggaga**tga**

MLKLFVLLLAVFICVSRSLRYLP**C**SQNNKTP**C**L**C**KNEKEL**C**PADFT**C**QLNSKGNR**C**MSKVKP**C**SKGNTWP**C**L**C**ENENKI**C**RPGHI**C**QMEPSGNR**C**IKRIKL**C**SEGNVTP**C**L**C**EDDKKI**C**RSGYY**C**QLDSNGNR**C**INKIKP**C**SESNVLP**C**L**C**ENKDKQ**C**RTGYY**C**LQNSTGYN**C**INLIKP**C**SENNEWP**C**L**C**VMKNDGQELRS**C**WFDET**C**KLNSNGNK**C**ISKGRR-

LRYLP**C**SQNNKTP**C**L**C**KNEKEL**C**PADFT**C**QLNSKGNR**C**MSKVKP**C**SKGNTWP**C**L**C**ENENKI**C**RPGHI**C**QMEPSGNR**C**IKRIKL**C**SEGNVTP**C**L**C**EDDKKI**C**RSGYY**C**QLDSNGNR**C**INKIKP**C**SESNVLP**C**L**C**ENKDKQ**C**RTGYY**C**LQNSTGYN**C**INLIKP**C**SENNEWP**C**L**C**VMKNDGQELRS**C**WFDET**C**KLNSNGNK**C**ISKGRR-

Theoretical pI/Mw: **8.69** / 23277.88

**Hnip_mHV6 (this study)**

**chromosome 2 position 19353002 - 19355228 rev+comp**

**atg**ctgaagctgtttgtcctcctcttggctgttttcatctgcgtgtctcgatcat*gt*gagttttaatttgagcttctcaatgaaagtgatatctaataagaagagctactatatcagtggaagatggatggatggatggatcgattggtgttatatcacataccatcaagggcggatttaggggcggggagccatggccctccccgtcggttggaagtgacaatataagaacaaactaagtgaacctatttgtaataattaatcaaaaatttattttaatttattatttgttaaattagtttgataacttaccaagtttgacgaaaattttagtagctgtacccaacccctcccctaaatccgcccactggttcctgccctctcccgccaaagtgttatagatccgcccttgctaccagtgtacactcacttctgttagcattagaaatgaatattggttgt*ag*taaggtatctacca**tgc**tcacagaataacaagactcca**tgc**ctt**tgt**aag*gt*aaattcttagattacaacttttattttctaaatgttatcataaatagcgtataacatcgtcatcgaatattc*ag*aatgaaaaagagctg**tgt**cctgctgatttcact**tgt**caactgaattctaagggaaacaga**tgc**atga*gt*aaaggtaaatttttttttattaattttcacttttttttacatcgtggtatattgcatattaattttattttatttcgaacaatttggtcaaagggcaaaggtcacacatgggtttaccccatacccctcgagcttaacgtactttatggttaaacaacgcctttaacaaaggctagcgacgctatttctgaattcttgaatactccttagaaaagcttctctcatatttaaaatagcaaatgtcagttgaagagattttttggcgatgtttcagcagacttggaaaatgggttattgtttctaatttaaaatcttgagcaaaaatggataattgaccgagtagtaccgagtacaaagtagttctttcgagaattgcttgagtcttttactgcaagtcaggccggcctattgggtagaatttctacatgcgacgataataataacaatcgttatgtgtcgcgttggcaacccagcctgcactgccagagatcgcccttgggtaggtatgccgtgtaaatggcgggtcgattaagaatattccatcacctttacatcatgctcggaaacataaggcattcaaaaaaataagatatgtacaaaaaatactaacaagcttatgtaagtatcgaagtttaagctgtgacttgctcgctcttgctcagctcgagttattacgtaactacgcgctctgcatgctacatcagttctgccgacacctgacttgccagtcaaaaccgatttgtgaacgactggccgagccgcactcagatcgctaaggagccgcgatttgccgaccactgaactagaaggtagcttgcataaatttaaaataagatcgtagtaccaaaagaaatacccagagggtgcagaatgccatcccaaggaacaaatgaggattattatgtattttgttgttgagtatcagattctagaagacttttactgtatttacttctgtttatgttaaaaatgaatgctggttgt*ag*ttaaaccc**tgt**tcgaaaggaaatacatggcca**tgc**ctc**tgt**gag*gt*aaattataagacagtaacttttatttaccaaatgttatcgcaaataacgtataatatcatcattgaacattc*ag*aacgaaaacaagatc**tgt**cgtcctggtcatatt**tgt**caaatggaacctagtgggaacaga**tgc**attaagagaa*gt*aatttttatataccataatttcacgtagataacacagatataagttaatatagcggacgaatttagctcgcaatttttccgatttgtgtaaaaaatcattaagatattaaagacattctctacaataaggctacgtgacatctgcatattaaagtgttaaacagcacccaggaattccccctgagggcagcttgccctccctgaaataagtagaataaactgaaataagttggaaaaattcggattcaaaattcacatgctgttgtgataatttctcgaaggtttaggatttcaaattacaaaaattattttaattaaaatattgctcccagacttctatctctctcggtattaaattaaattct*ag*atgga**tgc**acaagatct**tga**

**atg**ctgaagctgtttgtcctcctcttggctgttttcatctgcgtgtctcgatcat

taaggtatctacca**tgc**tcacagaataacaagactcca**tgc**ctt**tgt**aag

aatgaaaaagagctg**tgt**cctgctgatttcact**tgt**caactgaattctaagggaaacaga**tgc**atga

ttaaaccc**tgt**tcgaaaggaaatacatggcca**tgc**ctc**tgt**gag

aacgaaaacaagatc**tgt**cgtcctggtcatatt**tgt**caaatggaacctagtgggaacaga**tgc**attaagagaa

atgga**tgc**acaagatct**tga**

MLKLFVLLLAVFICVSRSLRYLP**C**SQNNKTP**C**L**C**KNEKEL**C**PADFT**C**QLNSKGNR**C**MIKP**C**SKGNTWP**C**L**C**ENENKI**C**RPGHI**C**QMEPSGNR**C**IKRNG**C**TRS-

LRYLP**C**SQNNKTP**C**L**C**KNEKEL**C**PADFT**C**QLNSKGNR**C**MIKP**C**SKGNTWP**C**L**C**ENENKI**C**RPGHI**C**QMEPSGNR**C**IKRNG**C**TRS

Theoretical pI/Mw: **8.97** / 9511.09

**Hnip_mHV7 (this study)**

**chromosome 2 position 19456932 – 19457750**

**atg**ttgaagctgtttgttgtcctcttggcttcttttggcatctgtctgtctgatt*gt*gagtttgaactgagtttttcaatgataaagttataaaaacgtgcttaaccaatgggatggatggataccacaacacgtgccgtgacataatcacttttttttattttaataatgaattttggttgt*ag*atcttaaacac**tgc**tctgttcaaaatacgtggcca**tgc**tac**tgc**gaa*gt*aaattataagattacaagttttagttatcaaatgttatcataaataactcataataccatcattgaaaattc*ag*aaagacaatata**tgc**cgtaaatatcaaact**tgt**gaaaagaattctgaaggatacaag**tgt**aaag*gt*aatttttattcaataaaaagtactattaattttatttatgttattaactttattttaaaaatgaatactggttat*ag*attttgtaaac**tgc**tggtataaaaataagcagcca**tgt**atc**tgt**gag*gt*aagttataagatttcaacttttatttatcaaatgttataaataatgtttaatatcatcattgaatattc*ag*tacaacagagttgta**tgc**catcaaggtcaaatt**tgt**gatgtgggtttttatgcatcttatagcaaa**tgc**aaag*gt*aattttgatattgttataaacgcgaaattaaatataaacttaattatttattgtgtttaattaattttgtcttatggtc*ag*atcgtttttctatgatgcctcagaagctgtcagctcagaagcccaaacatccatttgcatccttaagtacctcaaattccataaacgct**taa**

**atg**ttgaagctgtttgttgtcctcttggcttcttttggcatctgtctgtctgatt

atcttaaacac**tgc**tctgttcaaaatacgtggcca**tgc**tac**tgc**gaa

aaagacaatata**tgc**cgtaaatatcaaact**tgt**gaaaagaattctgaaggatacaag**tgt**aaag

attttgtaaac**tgc**tggtataaaaataagcagcca**tgt**atc**tgt**gag

tacaacagagttgta**tgc**catcaaggtcaaatt**tgt**gatgtgggtttttatgcatcttatagcaaa**tgc**aaag

atcgtttttctatgatgcctcagaagctgtcagctcagaagcccaaacatccatttgcatccttaagtacctcaaattccataaacgct**taa**

MLKLFVVLLASFGICLS

DYLKH**C**SVQNTWP**C**Y**C**EKDNI**C**RKYQT**C**EKNSEGYK**C**K

DFVN**C**WYKNKQP**C**I**C**EYNRVV**C**HQGQI**C**DVGFYASYSK**C**KDRFSMMPQKLSAQKPKHPFASLSTSNSINA-

Theoretical pI/Mw: **8.82** / 12635.44

**Hnip_mHV8 (Müller et al. 2025)**
