# Supplementary material for: The great diversity: monomeric and oligomeric hirudins, hirudin-like factors and decorsins in the Asian medicinal leeches Hirudo nipponia and Hirudo tianjinensis
Source: Parasitol Res. 2026 Feb 7;125(1):18. doi: 10.1007/s00436-026-08634-0 (PMC12882960; doi:10.1007/s00436-026-08634-0)
Supplement: Supplementary file 1 — Supplementary Material 1 (ZIP 660 KB) [file 436_2026_8634_MOESM1_ESM.zip › S2_putative hirudin genes on chromosome 2 of Hirudo tianjinensis.docx]

Supplementary Information File S2: Localization of putative monomeric and multimeric hirudin genes on chromosome 2 of *Hirudo tianjinensis*

**chromosom 2 position 1267539 - 1270337**

**atg**ttaaagctgtttgttgtcctcttggctttttttggcatcggtctgtctcaaa*gt*gagtatggtctgagtttctgaacgaatgactgatatataataaataaagcttcaccaatgatttgggatggatggatttgaaagatggacgcatggatgtaaggatggatagatataacattacatatcttgataaattcacttcagtttattttaaaaatgaatgttggttgt*ag*agatt**tgt**aaacac**tgc**tctgatcgaaatgggaatgaacca**tgc**ctc**tgt**gga*gt*aagtcataagatttatttacaaaatgttattataaatattattaatatcgtcattgaacgttc*ag*aatggctctaaggta**tgt**ggtcgatatgaagat**tgt**gaaggaaacata**tgc**aaag*gt*aatttttatataaaatttacaattaatttcacatttgttattaattttatttttgttattaatttcattttttaaaatttacttttatgtcaataatattgtatttttattttattaattccgatatgctcaacgaaatagtgatacaaaacaaaacgtgccataccaataatttggaatagatggatggatggatggatggatggatggatagatggatagatagatattatatcacataccgtgatattttagtttctgtttattttaaaaatggttat*ag*agatttacgatcgc**tgc**tatgttcggaataagtggcca**tgc**ctt**tgt**ggg*gt*aagttataagattacaactgaagatgaaatt**tgt**gaaacaaagataaacaaa**tgc**aaaa*gt*aacacaaagttattattaatactattaacctcaacgaaaatgtgataattaataaaaagtgcctcaccaatgattttggagcgatggatgcaaggatggatggatgtaacattacataatattacatatcgtgatatattcacttctgttaattctaaaaatgatgttggttgt*ag*agatttataaacac**tgc**tctgatcgaaatgggaatgaacca**tgc**ctc**tgt**ggg*gt*aagttataagatttatttacaaaatgttattataaatattattaatatcgtcattgaacattc*ag*aatggctctaaggta**tgt**ggtcgaaatgaagat**tgt**gaaggaaacata**tgc**aaaa*gt*aattttgctataaaatgagtttctcaacgataaagatatatgtaataaaaagtgctttcccagtgataagggatggatgggtggatggatggatggatggttggatggatggatggatggatggatggatggatggatggatggatggatggatggatggatggatggatggatggatggatggatggatggatggatggatggatggatggatggatggatggatggatggatggatggatggatggatggatggatggatggatggatggatataacattacataccatgataaattcacttctgtttattttaaaaatgaatattggttgt*ag*tgatttttgaactc**tgc**tctgatcaaaatgaatggcca**tgc**ttc**tgt**gtg*gt*aaataataagatttcaacttttatttacaaaatgttatcataaataacatgtaatatcttcattgaacattc*ag*ggtgaaagtttg**tgt**agcccaactgaaact**tgt**gaactgaatcctgaaggaaacaaa**tgc**aaag*gt*aatttttatgcaataaaattttctattaatttttacaattgttattaattatatttttgttactaaatttttatgttattaattttacttatactagcaatttcatatttttattaaatatataatattgtaattttttcttaattattacgctcaacgaaagagtgatatgcattaagacgtgctacaaaactgatgagggactgattgatggatgggtgaatgaatggatggatggatgggttggtttatggatggatgattttgtcgatggatggatataacattacataccgtgatatattcacttctgtttattttgaatatgaatgttggttgt*ag*agaaagttagtctc**tgc**tttgatcgaaatgagcatcca**tgc**cgc**tgt**gag*gt*gagttataaggttacaacttttatttatcaaatgttatcataaataacgtataatattttcattgagcattc*ag*ggtgataatata**tgc**aatacatatgaacaa**tgt**gacctaggaattaacaaa**tgc**aaaa*gt*gattttcatataatttatcattattttttttaatttcaattttaaaaaataatattgtaatttaatatttatattatattaattattcaaaattatatattaatactatgctcttaatacttataataaaaatgaaagtggattgt*ag*aaaaatac**tgt**tcggaaagaaatacgtatcct**tgc**ttc**tgt**gagaaaaatgtaaag*gt*aataacttttatttatcagatgttatcataaatagagtataatatcatcattgaacattc*ag*aatggtgatcgtacc**tgt**agccgaggtcaaatt**tgt**gaactgaatcctgaaggaaacaaa**tgc**atatttg*gt*atttttttaaatgaaatttaatattctaataacaatttaataatattttaataacttaattttatcaattttttttatgttattaattatattttattattaatttcatttttttgttattactttttattttacaaaaattattttgtaatttattactaatactaagatcttaatcttggattaattaatagaggcaaacagtatctaaagttgaaatttcttttttgactc*ag*agatg**taa**

**atg**ttaaagctgtttgttgtcctcttggctttttttggcatcggtctgtctcaaa

agatt**tgt**aaacac**tgc**tctgatcgaaatgggaatgaacca**tgc**ctc**tgt**gga

aatggctctaaggta**tgt**ggtcgatatgaagat**tgt**gaaggaaacata**tgc**aaag

agatttataaacac**tgc**tctgatcgaaatgggaatgaacca**tgc**ctc**tgt**ggg

aatggctctaaggta**tgt**ggtcgaaatgaagat**tgt**gaaggaaacata**tgc**aaaa

tgatttttgaactc**tgc**tctgatcaaaatgaatggcca**tgc**ttc**tgt**gtg

ggtgaaagtttg**tgt**agcccaactgaaact**tgt**gaactgaatcctgaaggaaacaaa**tgc**aaag

agaaagttagtctc**tgc**tttgatcgaaatgagcatcca**tgc**cgc**tgt**gag

ggtgataatata**tgc**aatacatatgaacaa**tgt**gacctaggaattaacaaa**tgc**aaaa

aaaaatac**tgt**tcggaaagaaatacgtatcct**tgc**ttc**tgt**gagaaaaatGTAAAG

aatggtgatcgtacc**tgt**agccgaggtcaaatt**tgt**gaactgaatcctgaaggaaacaaa**tgc**atatttg

agatg**taa**

MLKLFVVLLAFFGIGLS

QKI**C**KH**C**SDRNGNEP**C**L**C**GNGSKV**C**GRYED**C**EGNI**C**K

EIYKH**C**SDRNGNEP**C**L**C**GNGSKV**C**GRNED**C**EGNI**C**K

MIFEL**C**SDQNEWP**C**F**C**VGESL**C**SPTET**C**ELNPEGNK**C**K

EKVSL**C**FDRNEHP**C**R**C**EGDNI**C**NTYEQ**C**DLGINK**C**K

KKY**C**SERNTYP**C**F**C**EKNVKNGDRT**C**SRGQI**C**ELNPEGNK**C**IFEM-

Theoretical pI/Mw: **5.40** / 21539.29

**Htia_mHV1 (this study)**

**chromosom 2 position 1277208 - 1280662**

**atg**ttttctttgaagctgtttgttgtcctcttggcttgttttggcatctgtctgtccgaaacag*gt*taatttgatatgagttactgaacgaaagagtgacaataaaaagtactttaccaatgatttgggatggaaggacggacggacggacggatggatataacattaaataccattttaaaaatgatgttggttgt*ag*agattaatattcgc**tgc**tcatatctaaaggggaatgaacca**tgc**ctc**tgt**ggg*gt*aaattataagattacaacttttatttacaaaatattatcattaataacgtataattttatattattaatatcttcattgaacattc*ag*catggctctattaaggtg**tgt**ggtcgaaatgaatat**tgt**gagggaatcaaa**tgc**aaag*gt*aatttttatgtaataaaatttactattaattttatttttgttattaattttagtgttattattattattattattattattatttgatttcattttataaaatttaattttacgtaaatagtattgtaatttattattattaatattattatcaacgaaagagtgatacgtaacaaaaagcgccccagcaataatttaaaatggatgaatggatggatggatggatggatggatggatggatggatggatggatggatggatggatggatggatggatggatggatggatggatggatggatggatataacattacataccattttaaaaatgattttggttat*ag*agattaagaaacac**tgc**tctgatcgaaatgggaatgaacca**tgc**ctc**tgt**ggt*gt*aaattataagattataacttttatttattaaatgttagcataaataacatgtaatatcttcattgaacattc*ag*gttaatagtctg**tgt**agcccagttgaaaca**tgt**gaacttaattctgaaggatacaaa**tgc**aaaa*gt*aatttttatttcataaattttactatttgtttataattatctttaggttattaattttattgttttgtttgtgtataatatagtaatttattactaacaccatgtttttaatatttatattaaaaatgaatatttcttat*ag*aaaaatac**tgt**tcggaaagaaatgaatatcct**tgc**tac**tgt**aggatatcttattgg*gt*aattacttttattgatcagatgttatcataaatagcgtataataacatcattgtacattc*ag*ggtgatgatattatc**tgt**aacccagctgaaatt**tgt**gaagtgaatcctaaaggatacaaa**tgc**gaaa*gt*aatttttatttcataaactttattattgattttaattatgttgtatattctatttttgtttataattatttttgggttattaatttatttttttttgataatagttttattttttataaatataataatgtaatttactattaatactatgcttttaatatttataataaaaatgaaagtggattgt*ag*aaagattt**tgt**tcggaaaattcaactaactgggtactg**tgc**tac**tgc**gag*gt*aaaatataagataataacttttatttatcaaatgttatcataaacaacatataatgtcatcattgaacattc*ag*gaggatcgtatt**tgt**gatcatctatatgaaaaa**tgt**gaatttaattctgaaggaaacaaa**tgc**aaatatg*gt*aattatttataattaaatttaataataattttagttatgttgttaattttatttttattattaatttcaaatttttgtttttaattttatttttacaaaaataatattataatttattattatttaaaatatatgttatgttcttattttttatattaaatataattgttggttgt*ag*aaaaa**tgc**tcgaaaagaaatgaatatcct**tgc**tac**tgt**gag*gt*aaatttaaaaataataacttttaattatcaaatgttatcataaataacgtataatatcatcattgaacattc*ag*aatgactctgaagta**tgt**gggaaatatcaagat**tgt**gaagtgaatcctgaaggaaacaaa**tgc**atag*gt*aaatttttaagttattaattttatatttttattaaagaaggatggatgtaaggaaggaaggaaggaaggatggatggatgtaaggaaagaaggaaggatgtatggatggatggatggatggatgtaaggaaggaaggaaggatggatggatagatgtaaggaaggaaggaaggaaggaaggaaggaaggatggatggatataacattacataccgtaatatattcactcctgtttgttttctaaaaatgatgttggttgt*ag*agattaaaattcac**tgc**tctgatcgaaaaagaaaggaacca**tgc**gtc**tgt**ggg*gt*aagttaaaatattacttttttttattaacttttatttattaaatgctatcataaataacgtataatattatcattatacattc*ag*aaggacgtc**tgt**cgttcacatgaaaaa**tgt**gttatgaattctgaaggaaacaaa**tgc**aaaattg*gt*acttttgacaatgaaatttaatattttaataataattcaataatattttaataatttaatttaacaaattttttttatggtattaatgatattttattttacacaaattattttgtaatatattactaatattaagatcttaatcttggattaattaatagaggcaaacagtgtctaaagttgaaatttattttttgactcagagttgtaatagctggagctaaaaaatttactattaattatacaaatgttattaattgtatttttgcaattaattttactttattatgaaattcattttttgtactaaatttacacaaatattattgtaatttattattaaaactattatactcagcgaaataatgatatgtaataaaacgtgcttcaaatctgatatgaaattgatggatggatggatatatggatggacgaacggatggatggaaggatggatggatgtaacattatatatcgtgatatattcactgtattttaaaaataaatgttggttgat*ag*atagatac**tgc**tctgaaataaatgaacaacct**tgc**ttt**tgt**gag*gt*aagttataagattacaacctttatttattaagtgttatcataaataacgtataatatcatcattgaacattc*ag*gagaatcgtatc**tgt**tattcatatgaaaaa**tgt**gaactgaattctgaaggaaacaaa**tgc**atacttg*gt*atttttacaatgaaatttaatattgtaattataatgtaatagtattttaataataatttaatagtattttaataatttaattttactaatttttttatgttatgaattatattttttatgaattttaatttttataaattacaaaaaatattttttaatttattactaatactaagctcttaaacttgcattatttaatagaggcaaacagtgtttaaagttgaaatttctttta*ag*acccagagatgtta**tag**

**atg**ttttctttgaagctgtttgttgtcctcttggcttgttttggcatctgtctgtccgaaacag

agattaatattcgc**tgc**tcatatctaaaggggaatgaacca**tgc**ctc**tgt**ggg

catggctctattaaggtg**tgt**ggtcgaaatgaatat**tgt**gagggaatcaaa**tgc**aaag

agattaagaaacac**tgc**tctgatcgaaatgggaatgaacca**tgc**ctc**tgt**ggt

gttaatagtctg**tgt**agcccagttgaaaca**tgt**gaacttaattctgaaggatacaaa**tgc**aaaa

aaaaatac**tgt**tcggaaagaaatgaatatcct**tgc**tac**tgt**aggatatcttattgg

ggtgatgatattatc**tgt**aacccagctgaaatt**tgt**gaagtgaatcctaaaggatacaaa**tgc**gaaa

aaagattt**tgt**tcggaaaattcaactaactgggtactg**tgc**tac**tgc**gag

gaggatcgtatt**tgt**gatcatctatatgaaaaa**tgt**gaatttaattctgaaggaaacaaa**tgc**aaatatg

aaaaa**tgc**tcgaaaagaaatgaatatcct**tgc**tac**tgt**gag

aatgactctgaagta**tgt**gggaaatatcaagat**tgt**gaagtgaatcctgaaggaaacaaa**tgc**atag

agattaaaattcac**tgc**tctgatcgaaaaagaaaggaacca**tgc**gtc**tgt**ggg

aaggacgtc**tgt**cgttcacatgaaaaa**tgt**gttatgaattctgaaggaaacaaa**tgc**aaaattg

atagatac**tgc**tctgaaataaatgaacaacct**tgc**ttt**tgt**gag

GAGaatcgtatc**tgt**tattcatatgaaaaa**tgt**gaactgaattctgaaggaaacaaa**tgc**atacttg

acccagagatgtta**tag**

MFSLKLFVVLLACFGICLS

ETEINIR**C**SYLKGNEP**C**L**C**GHGSIKV**C**GRNEY**C**EGIK**C**K

EIKKH**C**SDRNGNEP**C**L**C**GVNSL**C**SPVET**C**ELNSEGYK**C**K

KKY**C**SERNEYP**C**Y**C**RISYWGDDII**C**NPAEI**C**EVNPKGYK**C**E

KRF**C**SENSTNWVL**C**Y**C**EEDRI**C**DHLYEK**C**EFNSEGNK**C**K

YEK**C**SKRNEYP**C**Y**C**ENDSEV**C**GKYQD**C**EVNPEGNK**C**I

EIKIH**C**SDRKRKEP**C**V**C**GKDV**C**RSHEK**C**VMNSEGNK**C**K

EIDRY**C**SEINEQP**C**F**C**ENRI**C**YSYEK**C**ELNSEGNK**C**ILDPEML-

Theoretical pI/Mw: **5.43** / 31986.23

**Htia_mHV2 (this study)**

**chromosom 2 position 1308787 - 1311525 rev + comp**

**atg**ttgaagctgtttgttgtcctcttggcttgttttggcatctgcctgtctgacatag*gt*gaatttgatatgagtttctgaacgaaagagttacatgtaataaaaagttctttaccaatgattttggatggatggacgcatggaagtaaggatggatggatggatggatggatggatggatggatggatggatggatggatggatggatggatggatggatggatggatggatggatggatggatggatggatggatggatggatggatggatggatggatggatggatggatggatggatggatggatggatggatggatggatggatggatataacattacataccattttaaaaatgatgttggttaaagggatcataaattttactattgatttcaattatgctattaattctatttttgtttataattatctttaggttattaattttattgttttgtttatatgtaatattgtaatttattattaacaccatgcttttaatatttatattaaaaatgaatgtttattac*ag*taaaaaac**tgt**tcggaaataaatgaatggcca**tgt**tac**tgt**gag*gt*aaattacatgataataacttttatttatcagatgttatcattaataacgtataatatcatcattgaacattc*ag*ggtgttaatata**tgc**agactaggtcacatt**tgt**gaagtgaatcctgaaggaaacaaa**tgc**aaaa*gt*aatttttatttaatgaattttactattgattttaattatgatgttaattctatgtttgttcttaattatttttaggttattaatttaatttaataatagttttattttttatataaataatattgtaatttattattaatactatgcttttaatatttataataaaaatgaaagatgattgt*ag*aaaaatac**tgt**tcggaaaattcaaataacgggcta**tgt**tac**tgt**gag*gt*aaattataaaataataccttttacttatcagaacttacttttagttacgaaatcttatcataaataacttataatatcatcattaaacattc*ag*gaaaatcgtatc**tgt**cttcattatgaaaaa**tgt**gaatttaattctgaaggaaacaaa**tgc**aaatatg*gt*aattatttataatgaaatttaataataattttagttatgttgttaattttatttttattattaatttcaattttttttgtttttaattttatttttacaaaaataatattataatttattattatttaatttatattatgttcttactttttatattaaatatgaatgttggttgt*ag*aaaaa**tgt**tcgaaaaaagatcgatatcct**tgt**tac**tgt**gag*gt*aaatttagaaataataacttttatttatcaaatattatcataaataaagtataatatcatcattgaacattc*ag*aatgactctgaagta**tgt**gggaaatatcaaaaa**tgt**gaactgaattctgaaggaaataaa**tgc**atag*gt*aaattttataagttattaattttatatttttaatactttaatttttttatgttattaattttatttttattatgaataatattgtaatttactattaatgctattatcctcaacaaaaaagtgatatgtaataaaaaaagtccctcaccaatgattttggagggatgggtggatggatgtaaggaaggaaggatggatggatataacattacataccgtaatacattcattactatttattctaaaaatgatgttggttgt*ag*agattaaacac**tgc**cttgatcgaaatgggaaagaaccaatacatgaacta**tgc**ctt**tgt**ggg*gt*aaattataagattacaacttttattttaaaaaatattatcataaataacttattattataagtgaacattaaattattatcattgaacattc*ag*aacaacacgcta**tgt**cgtacatatgaagat**tgt**gattctgagagaatgaaa**tgc**aaaa*gt*aaaatttactattaattatacaaatgttattaaattttatttttgctattaattttattttattatcaattccatttttaaaaaattaaatttacacaaatatgtaatatatagtattgtattttattattaatactattatgctcagcgaaagaatgatatgtaataaaacgtgcttcaaaaaatggatggatggatggatggatgaatgaatggaatggatggataaatggacagtcggacggacggacggacggatggatataacattacgtacgtgatatattcacttctgtgtattttaaaaatgaatcttggttggt*ag*aaagatac**tgc**tctgaaataaatgaacgttct**tgc**ttc**tgt**ggg*gt*aagttataagattacaacttttatttatcaaatgttatcatatataacgtataaaatcaacattgaacattc*ag*gagaatcgtatc**tgt**cactcacatgaaaaa**tgt**gaagtgaattctaatggaaacaaa**tgc**gtatttg*gt*attttttacaatgaaatttaatattttaataataacttgatagtattttaataatttaatattactaattgttttatgttatgaattatatatttttattaatttcattttttttattaattttaattttgaattaaatttaaataataataaaaataatttttgtaatttattactaatactaagctcttaaacttgcattatttaatagaggcaaactgtgtttaaagttgaaatttttttta*ag*actcagaggtg**taatag**

**atg**ttgaagctgtttgttgtcctcttggcttgttttggcatctgcctgtctgacatag

taaaaaac**tgt**tcggaaataaatgaatggcca**tgt**tac**tgt**gag

ggtgttaatata**tgc**agactaggtcacatt**tgt**gaagtgaatcctgaaggaaacaaa**tgc**aaaa

aaaaatac**tgt**tcggaaaattcaaataacgggcta**tgt**tac**tgt**gag

gaaaatcgtatc**tgt**cttcattatgaaaaa**tgt**gaatttaattctgaaggaaacaaa**tgc**aaatatg

aaaaa**tgt**tcgaaaaaagatcgatatcct**tgt**tac**tgt**gag

aatgactctgaagta**tgt**gggaaatatcaaaaa**tgt**gaactgaattctgaaggaaataaa**tgc**atag

agattaaacac**tgc**cttgatcgaaatgggaaagaaccaatacatgaacta**tgc**ctt**tgt**ggg

aacaacacgcta**tgt**cgtacatatgaagat**tgt**gattctgagagaatgaaa**tgc**aaaa

aaagatac**tgc**tctgaaataaatgaacgttct**tgc**ttc**tgt**ggg

gagaatcgtatc**tgt**cactcacatgaaaaa**tgt**gaagtgaattctaatggaaacaaa**tgc**gtatttg

actcagaggtg**taa**

MLKLFVVLLACFGICLS

DIVKN**C**SEINEWP**C**Y**C**EGVNI**C**RLGHI**C**EVNPEGNK**C**K

KKY**C**SENSNNGL**C**Y**C**EENRI**C**LHYEK**C**EFNSEGNK**C**K

YEK**C**SKKDRYP**C**Y**C**ENDSEV**C**GKYQK**C**ELNSEGNK**C**I

EIKH**C**LDRNGKEPIHEL**C**L**C**GNNTL**C**RTYED**C**DSERMK**C**K

KRY**C**SEINERS**C**F**C**GENRI**C**HSHEK**C**EVNSNGNK **C**VFDSEV-

Theoretical pI/Mw: **5.94** / 22434.28

**Htia_mHV3 (this study)**

**chromosom 2 position 1327361 - 1331102 rev + comp**

**atg**ttaaagctgtttgttgtcctcttggcttcttttggcatcggtctgaatcaag*gt*aagtgtggcctgagtttctgaacgaaagacttacatgtaataaaaagagctttacaaatgatttgtgttggatggatggattgatttgatagatggacgcatggatgtaaggatggatggatataacattacataccgtgataaattcacttctgtttattttaaaaatgattgttgtttgt*ag*tgatttataaacac**tgc**tctgaacgaaatgggaatgaatca**tgc**ctc**tgt**ggg*gt*aagttataagatttacttacaaaatataatcataaataacgtaaataacatgatattattaatttcttcattgaacattc*ag*gatggttctaaggta**tgt**ggtgcatatgaaatt**tgt**gaagaaaacgaa**tgc**gaaa*gt*aattttgctataaaatttacaataaattttatatttattattaattttttacaataaattttatatttattattaattttatatttattattaattttatttttgtttttaattttttatgttattaatttatttttattaataatttcgttttttttaatttttttttatataaataatattttaatttattattactaatattattattaacgaaagagtattacgcagcaaaaagcgcctcagcaataatttagaatggatgaatggatggatggatggatggatataacattacataccataataaattcacttatgtttattttaaaaatgaatgtttgttgt*ag*agaagtttaaaccc**tgc**tatcgtggaaatgaatatcca**tgc**ttc**tgt**gtg*gt*aaataataagatttcaacttttatttacaaaatgttatcataaataacatgtaatatcttcatcgaacattc*ag*ggtgaaagtttg**tgt**agcttcactgagact**tgt**gaactgaatcctgaaggaaacgaa**tgc**aaaa*gt*aatttttatgcaataaaattttctattaatttttacaattgttattaattatatttttgttaattatattttacttaggttagcaatatcatatttttattaaatttacacaaataatattgtacttttttcttaattattacgctcaacgaaagagtgatatgcattaaaacgtgctacaaaactgatgagggattgattgatggatgggtgaatgaatggatggatggatggatggatggttttgtcgatggatggatataacattacataccctgatatattcatttttgtttattttaaatatgaatgttgattgt*ag*gtatttataaactc**tgc**tctgaacgtaatgagtatcca**tgc**atc**tgt**gac*gt*aagttataagattacagcatttatttatcaaatattatcataaatgacgtataatattgtcgttgaacattc*ag*gatgatata**tgc**attgatttggcatta**tgt**gacctggaatttaacaaa**tgc**aaaa*gt*aattttcatataaaaaaatttatcaataattttgtattaatttcaatttaaaaaataatattgtaatttattattatatattttaatatatatttttactataaatattatatatattaattaaaatattattaaattttatatatattaatactatgctctcaatgtgtgtaataaaattgaaagtggattgt*ag*aaaaatac**tgt**tcggaaaaaaatacgtttcct**tgc**tac**tgt**gagacaacagaagag*gt*aataacttttatttataaaatgttgtcataaataacatcataaatatgtcataaatataatatcgtcactgtattcattgaacattc*ag*aatggtgatcgtagc**tgt**agacaagatcacatt**tgt**gaactgaatcctgaagggaacaaa**tgc**aaaa*gt*aatttttatttaataaattttactattgattttaattatgttgttaattctaattttgtttataattatatttaggttagtaattttattgttttgcttgtgtataatattgtaatttattaataatactatgctcttaatatttatatcataaatgaatcttgattga*ag*aaaaatac**tgt**tcggaaaatgtaactgactggcca**tgc**ttc**tgt**gag*gt*aaattattaagtaataacttttatttatcagatgttatcattaataatgtataatatcatcattgaacattc*ag*aatgaaaatcatacc**tgt**gaccgaggtcaaatt**tgt**gaactgaatcctgaaggaaacaaa**tgc**aaaa*gt*aatttttatttaataaattattctattgattttaattatgttgttaattctatttttgtttttaattatttttaggttagtaatttaattttttttttaatagttttattttttttataaaaataatattgactatggtcttaatatttataataaaaatgaattttgcttgt*ag*aaaaatac**tgt**tcggaaaactcaactgactacagtgat**tgc**tac**tgt**gag*gt*aaattattagataagaacttttatttatcaaatgttatcataaataacgtgcaatgtcatcattgaacattc*ag*gaggatcgtatt**tgt**aatcatctatatgaaaaa**tgt**gaatttaactctcaaggaaacaaa**tgc**aaatatg*gt*aattatttataatgaaatttaataataattttagttatgttgttaattttatttttacaaaaataatattataatttattattatttaaaatatatgttatgttcttattttttatattaaatataattgttggttgt*ag*aaaaa**tgc**tcgaaaagaaatgaatatcct**tgc**tac**tgt**gag*gt*aaatttaaaaataataactttcagttatcaaatgttatcataaataacgtataatatcatcattgaacattc*ag*aatgactctgaagta**tgt**gggaaatatcaagat**tgt**gaagtgaattttgaaggaaacaaa**tgc**atag*gt*aatttgtatgagttattaattttatgtttttaataattttaattctgttattattttctttatgctattaattttatttctattatgaataacattataagttattattaatactattttcctcaacaaaaaagtgatatgcaataaaaaaaagtgccgcaccaatgatttgggagagagggagtgatggatggatggatggatggacggatggatggatagatggatggatagttggatgaatggatgtaacattacgtaatacattaatttctgtttatttttaaaatgaatgttggctgt*ag*atgcatataaacgt**tgc**cctgatcgaacatggaaaaggatgcttgaacca**tgc**ctc**tgt**ggg*gt*aataacttttatttattaaatgttatcataactaatgtataatatcaacattgaacgtcc*ag*gaggatcttgtc**tgt**tttagtgatgaaaaa**tgt**gtactgaacaaaactgaaggaaacaaa**tgc**atatttg*gt*atttttttaaaatgaaatttaatattctaataataatttaataatatttgaataatttaattttactaatttttctatgttattaattatatcatattattaatttcatttgtttgttattaaattttattttttttattaattttaattttgaattaaatttaaataataataaaaataatttttgtaatttattactaatactaagctcttaaacttgcattatttaatagaggcaaacagtgcttaaagttgaaatttctttta*ag*actcagaggtg**taaTAG**

**atg**ttaaagctgtttgttgtcctcttggcttcttttggcatcggtctgaatcaag

tgatttataaacac**tgc**tctgaacgaaatgggaatgaatca**tgc**ctc**tgt**ggG

gatggttctaaggta**tgt**ggtgcatatgaaatt**tgt**gaagaaaacgaa**tgc**gaaa

agaagtttaaaccc**tgc**tatcgtggaaatgaatatcca**tgc**ttc**tgt**gtg

ggtgaaagtttg**tgt**agcttcactgagact**tgt**gaactgaatcctgaaggaaacgaa**tgc**aaaa

gtatttataaactc**tgc**tctgaacgtaatgagtatcca**tgc**atc**tgt**gac

gatgatata**tgc**attgatttggcatta**tgt**gacctggaatttaacaaa**tgc**aaaa

aaaaatac**tgt**tcggaaaaaaatacgtttcct**tgc**tac**tgt**gagacaacagaagag

aatggtgatcgtagc**tgt**agacaagatcacatt**tgt**gaactgaatcctgaagggaacaaa**tgc**aaaa

aaaaatac**tgt**tcggaaaatgtaactgactggcca**tgc**ttc**tgt**gag

aatgaaaatcatacc**tgt**gaccgaggtcaaatt**tgt**gaactgaatcctgaaggaaacaaa**tgc**aaaa

aaaaatac**tgt**tcggaaaactcaactgactacagtgat**tgc**tac**tgt**gag

gaggatcgtatt**tgt**aatcatctatatgaaaaa**tgt**gaatttaactctcaaggaaacaaa**tgc**aaatatg

aaaaa**tgc**tcgaaaagaaatgaatatcct**tgc**tac**tgt**gag

aatgactctgaagta**tgt**gggaaatatcaagat**tgt**gaagtgaattttgaaggaaacaaa**tgc**atag

atgcatataaacgt**tgc**cctgatcgaacatggaaaaggatgcttgaacca**tgc**ctc**tgt**ggg

GAGgatcttgtc**tgt**tttagtgatgaaaaa**tgt**gtactgaacaaaactgaaggaaacaaa**tgc**atatttg

actcagaggtg**taaTAG**

MLKLFVVLLASFGIGLN

QVIYKH**C**SERNGNES**C**L**C**GDGSKV**C**GAYEI**C**EENE**C**E

KKFKP**C**YRGNEYP**C**F**C**VGESL**C**SFTET**C**ELNPEGNE**C**K

SIYKL**C**SERNEYP**C**I**C**DDDI**C**IDLAL**C**DLEFNK**C**K

KKY**C**SEKNTFP**C**Y**C**ETTEENGDRS**C**RQDHI**C**ELNPEGNK**C**K

KKY**C**SENVTDWP**C**F**C**ENENHT**C**DRGQI**C**ELNPEGNK**C**K

KKY**C**SENSTDYSD**C**Y**C**EEDRI**C**NHLYEK**C**EFNSQGNK**C**K

YEK**C**SKRNEYP**C**Y**C**ENDSEV**C**GKYQD**C**EVNFEGNK**C**I

DAYKR**C**PDRTWKRMLEP**C**L**C**GDLV**C**FSDEK**C**VLNKTEGNK **C**IFDSEV--

Theoretical pI/Mw: **4.83** / 36189.50

**Htia_mHV4 (this study)**

**chromosom 2 position 1445023 - 1449948**

**atg**ttttctttgaagctgtttgtcgtcctcttgactgttttcatctgcgtgtctcga**tag**t*gt*gagttttaatttgagcttctcaatgaaagtgatatctagtaagaagtgctactatatctggaggatggatggatggatggatcgattggtgtaatatcacataccaccaagggcggatttaggggcggggagccatggtcctccccgtagtttggaagtgataatataagaaaaaacgaagtaaatctatttgtaataattaatgaaaaaaataataaaaaattcattttaatttaatatttgttagattactttgataacttaccacatttgaagaacattttagtagctgtacccccctacatccgcccacccgttcctaccctctcccgccaaagtgtcatagatccaccctaactaacagggtatactcacttctgtttacattagaaatgaatattggttgt*ag*caaggtatctacca**tgc**tcagagagtaacaagactcca**tgc**ttc**tgt**aag*gt*aaattctaagattacaacttttattttgcaagtgttatcacaaataacgtataacatcgtcatcgaatattc*ag*gacggaaaagagctg**tgt**cctgctgatttcact**tgt**cgactgaattcttctggaaacaga**tgc**atgagtagag*gt*cactttttttattataaattatcactttcttacatagtgacatattgcatattaattttattttatttcgaacaatttggtcaaaggacaaaggtcacacatgtgtttacccccatacccctcgagcgtaacgtactttatggtcggtcccctttaacaaaggctagcgacgctatttctgaaatcctgaatactctttagaaaagcttctctcatatttaaaattgttgtttgaaatagcaaatgtcagttacagagatattttttgacgatgtttcagcagacttggaaaatgggttattgtttctaatttaaaatcttgagcaaaaatggacaatttaccgagtagtaccgagtagaaagtagttctttcgagaattgcttgagtcttttactgcaggtcaggttggcctattgggtagaatttctacttgcgacgataataacaacagtcgttatgtgtcgcgttggcaacccagcctgcattgccagagatcgccctcgggtaggtactccatgtaaatggtgggtggattaagaatattccatcacctttagatcctgctcggaaacataaagacactcaaaaaaataagataagtaaaaaaatactaacaagcatatgtaagtaccgaagtttaagctgtgactggctcgctctcgctcagctcgtgttattatataactatgcgctctacatgctacatcagttcttgccagtcaaaaccgatttgtgaccgactggccgagccgcactcagatcgctaaagagccgcgatttgccgaccactgaactagaaggtagcctgcataatttttaaaataagatcctagtatcaaaagaaatagccagagggtgtagaatgccataccaaggaacgaatgaggattaatatgtattttgtcgctgaggaaaagattctagaagacttttactgtatttacttttgtttatattaaaaatgaaaactggttctattaat*ag*ttaaaccc**tgt**tcgaaaagaaatgca**tgg**cca**tgc**ttctgtgag*gt*aaattataagacaataacttttatttaccaagtgttatcggaaataacgtataatatcatcattgaacattcaga*ag*gaaaacaagata**tgt**cttcctggtcatttt**tgt**caaatgaaatctaatgggaacaga**tgc**attaagagaa*gt*aatttttatataccataatttcacgtagataacacagatataagttaatatagctgacgaatataactcgcaatttttccgatttgtgtaaaaaatcattaagatgttaaagacattatctacaataagtctacgtgacatctgcatattaaagtgctaaacatcacccagggattccccctgagggggcaagggaagggcagcttgccattccacttggaataaaccgaaataagttggaaaaattcggattcaaaagtcacatgcatgttgtgataatttctctaaggtttaggatttcaaattataacatttattttaattaaaataatgctcccagacttctatctctctcggtattaaattaaattctagatggactcacaagatctgaatctttgaaagaacatctgtgaacacctccttagaagccattgcggaagtactttatgaaatttcgtttcaaaagtgacatgacttaataactaaatctaatgctgtggctttatacaaacttatcatgagctttgacattatgttgtctgttacgtctatgtacatagtaaaagtgttaactgaatctttggaaaaattggcctgagtgttgttgatgcagtagaaaacataaaagcgcccaccaggcgaccttgaaattcctcaacattgaaaccgagatgatacaaattaatccagactggaaaccaagtttcattcaaattacacataaatgcggaagactattttaagcgtcgtcacagacatagagtggcgatttgatgacaaagttgaatctcattctttgggagggagtttaaggaagttctggatccactaataaactattagaagtcgaacgtgttacagtgtgtaaatcagtggtgcacaaccttttttgaccgagggctgatatcatcagcatcaagacaacgcaaacaataagacacatgtacagaaaaataaagacaaagttgtgaactagatgggctgatgttcatagtaaatcttgacagctttaagttaatgtcacaacgtgacagctgaggctgcatgtctgcaacaagctttggcatatcaggctgttcgtagtttgtataaggatggaaaataatcgaacagcaaaaatggcggagcgccctctaagataattggtgaggcatagctgtcacgctcaaaatactaagtaaacagtttttttgagttgagaaaaactgttctttatggcccccccccctcccgaattttgcattcggtacgtccctgacagcaccgagcaaggtatggtgtctgctcttactgcagtgttgtgaaaacactgttacatctcaaactgtaaactctaacaccaattataattattcttgtcataattattattaaatgtctgctcttaaggatcaggagctagaggacttttactgttttcacttctgtctatattaaaagtgaatattggttgc*ag*taaaactc**tgc**tcggaaagtaatgtcactcca**tgt**ctc**tgt**aag*gt*aaattataacttttacctatcattaataacgtataatatcataattgaacattc*ag*gatgataaaataata**tgt**cgttatggttactat**tgt**cacctggattccaaaggaaacaga**tgc**ataaacaaaa*gt*aattctcttataataaaatattgtattatattagtgatattgttatattattatataagaaaatattttaatttattattaatactattttacataatataatattgtatatttattttattttatttaaaaaaaattgttcaaaggataccttttttggttgtcgtttaaatcgtttttctcaagttacaataataactatttaatcatcatcatcagcatcgtccctttgccaaatgctacaatagaaatagaagatttcaaattttatctattttatcattttacattctatccatgtttacaccgtaaagagttcttgcaaagggccgcattagtgtttgacatgcctgctctatatgccttttactttattcacttctgtttatattaaatatgagtattggttgc*ag*ttaaaccc**tgc**tcagaaagtaacgtgctacca**tgc**ctc**tgt**gag*gt*aaattatgaatttataactgttatttatcaaatgctttcataaataacgtataatatcaccattgaacatac*ag*aataaagacaaacaa**tgt**cgtattggctactat**tgt**cttcagaattctactggatacaat**tgc**ataaacttaa*gt*aatttttattactatttgaacacctccttagaagccttaattgttattagtaaaatatatatttattttattttattttatttcaaaaaaattggtcaaagatcattttaaaagcgcctctaatataaccagaaagaaagacaatcactaaagaaaaacgaaaaaagtacgtttgcaacacccatccataactccgcgacacaaaataaagggacagaaatgaataatatagttaaatacagtcaggaccttgacaattactaactatctagtcatcatcatcagcatcgtccctctgccaaacacgccaatagaaacagacgaaatcaaatttactttactataatacctattcacgtttattgtaattaattaaataataataacaaaataataacataatgttatgtaatatttaatatgtttaataatttatctcaatttacaatttattattaacatttaagaattaattagtttaaatgtattaaacggtaaaaaatgaatgtcgattgt*ag*ttaaaccc**tgc**tcggaagataatgagtggcca**tgc**ctc**tgt**gtg*gt*aaattggaagataatagcttttatttatcaaatgctatcataagtaacatgtaatatcatcattga*ag*atgaaggataatggacaggaattacgtgca**tgt**tggcacgacgaaact**tgt**gaactgaatcctaaaggaaacaaa**tgc**ataac*gt*aattgttctaaatgttaaaaatattaattttatttttacataacaaatatcgaattttagcatcctattaatctgttattatttaagagagacagactaaaactgaaatttaatgaaattctatttattacttagagataaacaaaaaaaaactcttttgagctgcataatgaaatattaattattaaatataatattctaatgtcatttcgac*ag*gaaaaggcaggaga**tga**

**atg**ttttctttgaagctgtttgtcgtcctcttgactgttttcatctgcgtgtctcga**tag**t

caaggtatctacca**tgc**tcagagagtaacaagactcca**tgc**ttc**tgt**aag

gacggaaaagagctg**tgt**cctgctgatttcact**tgt**cgactgaattcttctggaaacaga**tgc**atgagtagag

ttaaaccc**tgt**tcgaaaagaaatgca**tgg**cca**tgc**ttctgtgag

gaaaacaagata**tgt**cttcctggtcatttt**tgt**caaatgaaatctaatgggaacaga**tgc**attaagagaa

taaaactc**tgc**tcggaaagtaatgtcactcca**tgt**ctc**tgt**aag

gatgataaaataata**tgt**cgttatggttactat**tgt**cacctggattccaaaggaaacaga**tgc**ataaacaaaa

ttaaaccc**tgc**tcagaaagtaacgtgctacca**tgc**ctc**tgt**gag

aataaagacaaacaa**tgt**cgtattggctactat**tgt**cttcagaattctactggatacaat**tgc**ataaacttaa

ttaaaccc**tgc**tcggaagataatgagtggcca**tgc**ctc**tgt**gtg

atgaaggataatggacaggaattacgtgca**tgt**tggcacgacgaaact**tgt**gaactgaatcctaaaggaaacaaa**tgc**ataa

gaaaaggcaggaga**tga**

MFSLKLFVVLLTVFICVSR**S**SS

RYLP**C**SESNKTP**C**F**C**KDGKEL**C**PADFT**C**RLNSSGNR**C**MSR

VKP**C**SKRNAWP**C**F**C**EENKI**C**LPGHF**C**QMKSNGNR**C**IKR

IKL**C**SESNVTP**C**L**C**KDDKII**C**RYGYY**C**HLDSKGNR**C**INK

IKP**C**SESNVLP**C**L**C**ENKDKQ**C**RIGYY**C**LQNSTGYN**C**INL

IKP**C**SEDNEWP **C**L**C**VMKDNGQELRA**C**WHDET**C**ELNPKGNK**C**IRKGRR-

Theoretical pI/Mw: **8.79** / 23307.06

**Htia_mHV5 (this study)**

**chromosom 2 position 1453196 - 1454013**

**atg**ttctctttgaagctgtttgttgtcctattggcttcttttgacatatgtctgtctgaaa*gt*gagtgatctgtgtttctcaatgataacgttttattacataatacataatgtgctacgtccgtgaagggtggatggatggatgtaacatcacataccacggtatatacgtttatgtttatattagggctgcagattaatgtgtgttaacgggcgattaatgcgttaagaaaataattaacgctgtgatcaatcagttaaattaaaacaatttttttgagtttctcaatgtttctcacgtttttcatcattttttttataaatttaacgcattaattaattattttattcgcagaaattttcttaaagcataaatcgcccgttgacgcatataaatctgcagccctattaataagctttaaatcaccttaaataattttttttaataaaaaaatgaagagttttttggaaattaattggataatagaatggtgcgatttaaaatgagttaactaagattaaaataatcaatctaagtgcgactaatcgcgattgaaaatattaatcgatctgcagccctagtttatattaaaaatgaatgttgattgt*ag*gaccaaagcgc**tgc**tcagtggttaatggagtgacgcct**tgt**ctg**tgt**aag*gt*aaattagaagattataacttttattcttcataattctaataaataacgtaaattatcatc*ag*attatgaatgggcgggaattctatgta**tgt**cctgctgggagcaaa**tgt**cgaacagatgttcgcggtaacttg**tgc**gttagaccgaactat**taa**

**atg**ttctctttgaagctgtttgttgtcctattggcttcttttgacatatgtctgtctgaaa

gaccaaagcgc**tgc**tcagtggttaatggagtgacgcct**tgt**ctg**tgt**aag

attatgaatgggcgggaattctatgta**tgt**cctgctgggagcaaa**tgt**cgaacagatgttcgcggtaacttg**tgc**gttagaccgaactat**taa**

MFSLKLFVVLLASFDICLSERPKR**C**SVVNGVTP**C**L**C**KIMNGREFYV**C**PAGSK**C**RTDVRGNL**C**VRPNY-

Theoretical pI/Mw: **9.34** / 5391.34

**Htia_HV5 (this study)**

**chromosom 2 position 1460097 - 1463027**

**atg**ttgaagctgtttgtagtcctcttggcttcttttggcatctgtctgtctaaattag*gt*gagtgtgatatgagtttctcaacgataaagctatatgtaaaaaaatgcttccccagtgatatgggatggatggatggatggatggatggatggatggatggatggatggatggatggatggatggatggatggatggatggatggatggatggatggatggatggatggatggatggatggatggatggatggatggatggatggatggatggatggatggatggatggatggatggatggatggatggatggatggatggatggatggatggatggatggatggatggatggatggatggatggatggatggatggatggatggatggatggatggatggatggatggatggatggatggatggatggatggatggatggatggatggatggatggatggatggatggatggatggatggatggatggatggatggatggatggatggatggatggatggatggatggatggatggatggatggatggatggatggatggatggatggatggatggatggatggatggatggatggatggatggatggatggatggatggatggatggatggatggatggatggatggatggatggatggatggatggatggatggatggatggatggatggatggatggatggatggatggatggatggatggatggatggatggatggatggatggatggatggatggatggatggatggatggatggatggatggatggatggatggatggatggatggatggatggatggatggatggatggatggatggatggatggatggatggatggatggatggatggatggatggatggatggatagaacatctcgtaccagggtacatttttttatcaatcaattttattttgtactttcacgaaaagttcacttctgtttatattaaatatgaatgttgattgt*ag*agcgc**tgc**tcagaacgtaaaagaactaat**tgc**ata**tgt**gag*gt*aaattatgagattgtaacttttatttatcaaatgttatcataaataacgtataataatatcgtcaatc*ag*ggtggctctgcc**tgt**ggtctatatgaaatt**tgt**gtactgaatgctgaaggaaacaaa**tgc**aaaa*gt*aatttttattaattattttattatttttattttatataattgtaattcattattaatactattatgtaataaaaggtgcttcaccagtgatgtgggattgttggatggatggatggatgcacggatggatggatggatggatggatggatggatggatggatgatcgatgaatggattgatggatggatggatggctatgcgtatggcgctatataaataaagtgacaataaattcacttctgtttatattaaaaatgaatgttggttgc*ag*agctttataaacac**tgc**tctgatcgaaatgagagggaacca**tgc**atc**tgt**ggg*gt*aaataataattattacaattattattatattattattataatattacaaatttaattaatcaactgttatcataaataacgtataatatgatactattaatatcatcgttgaaactttc*ag*gatgactctaattta**tgt**ggtcgatatgaaaat**tgt**gaaggaaacata**tgc**aaaa*gt*aatttttatataataaaattaattattaatttttctaatgtttttaattttattttaattattaatttcatttttttttaaattttattttacataaataacattgtaatttattaataatgctgttatgctcaacgaaagagtgatatgtaataaaaagtgcttcacaatgacttgggatggatggatggatcgatcgattgctggatgaatggatgggtataacacttctgtttattttaaaaatgaatgttgtttgt*ag*aaatttataaaagc**tgt**tctagtcataatgcgtggcca**tgc**ctc**tgt**ggg*gt*aaattagattacaacttttatttaccaaatgttatcataaataacgtataatatcataattaaacattc*ag*gataagtta**tgt**ggtatagatgaaaat**tgt**caaacgggtttactaggagatattaacaaa**tgc**aaaa*gt*aattttcatatattaaaatctgttatcaattttgtatttattaacattttttcttaataattgtatttttacaaatatattattgttgt*ag*aaaattca**tgc**tcggaagaaaatagttatcct**tgt**ctt**tgt***gt*aactttcagttatcaaatgttaccataaataacgtataatatcatcatcgaactttc*ag*ggtggctcttac**tgt**cgtagatatgaaatt**tgt**gaactgaaaggaaataaa**tgc**ataacac*gt*aatttttgtaataaattttacaatcaattatttattattttatattaattttatttttgtttttattttttctggttattaattttattttttataaaaataatatcgtaattttgttctacatatttatattgaaaatgaattttggttgt*ag*aaaaattc**tgc**tcggaaaaaaataatgaatatcct**tgc**att**tgt**gtg*gt*aaatttgaagataataacttttattaatcagatgttatcataaataatgtataacatcgtcattgaacgttt*ag*ggtaatcatgtc**tgt**ggtcaacatgaaact**tgt**gaactgaatcctgaaggaaacaaa**tgc**ataa*gt*aatattttttataattttatttgtttaattaatcttttttgttagcaatatttttgtgttattaataatactaattttatttttataaaaataataatataatccattactaatattatgctcttaatctggtattaattaatagaggcaaacagtgtctaaagttgaaatttcttttta*ag*actcagaggtg**taa**

**atg**ttgaagctgtttgtagtcctcttggcttcttttggcatctgtctgtctaaattag

agcgc**tgc**tcagaacgtaaaagaactaat**tgc**ata**tgt**gag

ggtggctctgcc**tgt**ggtctatatgaaatt**tgt**gtactgaatgctgaaggaaacaaa**tgc**aaaa

agctttataaacac**tgc**tctgatcgaaatgagagggaacca**tgc**atc**tgt**ggg

gatgactctaattta**tgt**ggtcgatatgaaaat**tgt**gaaggaaacata**tgc**aaaa

aaatttataaaagc**tgt**tctagtcataatgcgtggcca**tgc**ctc**tgt**ggg

gataagtta**tgt**ggtatagatgaaaat**tgt**caaacgggtttactaggagatattaacaaa**tgc**aaaa

aaaattca**tgc**tcggaagaaaatagttatcct**tgt**ctt**tgt**

ggtggctcttac**tgt**cgtagatatgaaatt**tgt**gaactgaaaggaaataaa**tgc**ataacac

aaaaattc**tgc**tcggaaaaaaataatgaatatcct**tgc**att**tgt**gtg

ggtaatcatgtc**tgt**ggtcaacatgaaact**tgt**gaactgaatcctgaaggaaacaaa**tgc**ataa

actcagaggtg**taa**

MLKLFVVLLASFGICLS

KLER**C**SERKRTN**C**I**C**EGGSA**C**GLYEI**C**VLNAEGNK**C**K

KLYKH**C**SDRNEREP**C**I**C**GDDSNL**C**GRYEN**C**EGNI**C**K

KIYKS**C**SSHNAWP**C**L**C**GDKL**C**GIDEN**C**QTGLLGDINK**C**K

KNS**C**SEENSYP**C**L**C**GGSY**C**RRYEI**C**ELKGNK**C**IT

QKF**C**SEKNNEYP**C**I**C**VGNHV**C**GQHET**C**ELNPEGNK**C**INSEV-

Theoretical pI/Mw: **6.93** / 20885.72

**Htia_mHV6 (this study)**

**chromosom 2 position 1467292 - 1473689**

**atg**ttgaagctgtttgttgtcctcttggctttttttggcatctttctgtctgattgtgagtttgatctgtctcaag*gt*gagtgtggcctgagtttctgaacgaatgactgatatgtaatagatagagcttcaccattgatttgggttggatggatggattgatttggtagatggacgcgtggatgtaaggatggatggatataacattacataccgtgataaattcacttcagattattttaaaaatgaatgttggttgt*ag*agatttataaacac**tgt**tctgatcgaagtgggaatgaatca**tgc**ctc**tgt**gga*gt*aattcataagatttatttacaaaatgttatcataaatattattaatatcatcattgaacattc*ag*aatggctctaaggta**tgt**ggtcgatatgaagat**tgt**gaaggaaacata**tgc**aaag*gt*aatttttatataataaaatttactattaaatttacacttgttattaattttatttttgttattaacatcatttttttaaatttattttatttaaatatattttaattttattttattaattctgatatgctcaacgaaatagtgatacttaacaaaacgtgccacaataatttagaatagatggatggatggatggatatgatgtgatatcacatgccatgatattttgttttctgtttattttaaaaatggttgt*ag*aaatttacgatcgc**tgc**tatgttcgcaataagtggcca**tgc**ctt**tgt**ggg*gt*aagtcataagattacaactgttatttatcacatgttaaataatgtataatatcatcattgaacattc*ag*gatgatcaagta**tgt**gataaagatgaaatt**tgt**gaaacaaagataaacaaa**tgc**aaaa*gt*aatacaaagttattattaatactattaacatcaacgaaaaagtgataattaataaaaagtgcctcatcaatgatttgggagggatggatggatataacattacatatcgtgatatattcacttctgttaattctaaaaatgatgttggttgt*ag*agattaatattcac**tgc**tctgatcgaaaaggaaatgaaccg**tgc**ctc**tgt**ggg*gt*aaattataagattatacaacttgtatttacaaaatgttaacataaataacataaaatatcttcattgaacattc*ag*aatgagactatg**tgt**tatcgatatgaagtt**tgt**gacctgaat**tgt**gaaggaaataaa**tgc**atag*gt*agtttttattagttattaattttatatttttattaattataattttgtcatttaattttgatattggattgatagatggatgggtgattggttggatataacattacatacatatttttttggtttattttcaaaattaatgttgtttgt*ag*agaagtttgcatcc**tgc**tctgatcgaaatgggaaggtacca**tgc**ttc**tgt**ggg*gt*aaattataaagttacaactttattttatcaactattatcataattgtttttgtggttatttgaaagaaacaaacaagccgttatatacacagaaaaaacaaaacgcgaaagcactaaataaattcaaataaacccaaagggccagccttaaggaagcacacatttcggctgtcggactgaccctgaaagtagctatataaaaaatcataagctatacccctcccctcaataaatattgtttgcctgacattttgaattgtaaataaattaatttgaacggtcatgttcagttagatcagttagatccttcatagatgaaggtatggaggagtaaagtctgtttatcataaataacgtttaatatgatattattgatatcatcattgaacattc*ag*aatgactccaaggta**tgt**agtcgatatgaatat**tgc**aaagaaaacaca**tgc**atag*gt*aatttttatgtaataaaatttactattaatttattattaattttgcttatgttataaaatttttatgttaataatttatttttattatcaatttcatttttttaaattttatttttacataaataatgttccaattaattgttaatactgttatgcccaacggaagagtgattcttaacaaaaagtgcctcaccaaagatttggaatggatgaatggatggatataacattgcataacgtgatattttcacttatgtttcttataaaaatgaatgcttggttgt*ag*tgtattatagaagt**tgc**tctgatcttaataagtggcct**tgc**atc**tgt**gag*gt*aaattataagattataactgttatttatcaaattttaacttacataacgtataatattgaacattc*ag*gaagaaaga**tgt**cgtttagatgaaatt**tgt**gaactaggagagattaacaaa**tgc**aaaa*gt*aattatcatataataaaatttgttattaatgtttttttaatgactttatttttacaaaaatattgctgtaatttgttagtaatattatgctattaatatgtatattaagaatgaaacttgattgt*ag*gaaaatac**tgt**tcggaaataaatgtgcagcca**tgc**ttc**tgt**gaa*gt*aaattataacataataacttataattaccaaatgttatcataaataacgtataaaatcatcattgaaaattc*ag*gatgatcatgta**tgt**gatgacgatgaaatt**tgt**gaagtaggagagattaacaaa**tgc**aaaa*gt*aattttcatataataaaattttttaataatgttttttattaattatatttttacaataatagtattttaataataataataataataataataataataataataataataataataataataataataataataataataataataataataataataataataataataataataataataataataataataataataataataataataataataataataataataataataataataataataataataataataataataataataataataataataataagtgcatttgtaaagcgccataagcgtcagcgacgctccgtccttggttgtgttggataaggtgagtctggaggctggccttgaattggttgaatgatgcagaggccctgaggttagccggtggagcgttccagaggagcggtgtacttgaaggactttgcttcaatcttgcttctcggaataggaatgctgagaaagttggatgtctccgaccgcaagcatcttcaatgggattacggggagggtactggcgaggttgcattccagtgctgaggtttttgtgcgcaaagagagaaaccttgaacttaattctatcagcaactggaagcctatgcagtgctcccaatgcttcggttggttcagcctcctatcataagtcgagctgatttattttgaatcctctgcagtttccgtagagaagcagaggatagttgatgtaaaatggagttacagtagctgagccttgacaagttgaggcaacggcaaagagccgcggacttcaactgcaagaagatgactgatatggcataaagcccatatttgatagttgcatgctctaacaactgtggaaacgtgcctgtagggtcagagggtgctgtcaagggtaaccccgatgagttttatggtgctggatagctcgatgctggtgtttggcaagactaacagaagcgatgctgagtctctgtatggaagggtgggtgcctagcatcatgacttcgaatttggacggattcagttgcagctggtttacgagatgccagtcttgaatcttctggatacattcagcagccttttgtaggtcggccatgatggttgttgatgagacgagtacatgaatgctggtgtcatcagcatacagctacacccaaagttcgatatcaaatcgcagattgggaaagtgtaagttatgaagtggatagccccgaggacagacccttggagaacaccaacgtcgagagagaagagttcggttgagctctcaccaagcttgacgaattgtttcctgtcgctgaggtacgacatcaaccacagaagagctgagtccttgatggcaaattcgtcgtcgaggcgacggagcaggatgctgtgataaacggtgtcgaaagctgctgataattcaagagtgaccagcattgtatatccgttcttgctgactgaattgacaccgtcaaaggtatggagaagtgcagtctccgtagagtggccgcagcggtaagcggattggtagctagaatagttactgttggataaaaggaagtatctgtgctatcgtatgggtggtgggaaatatgcagtcgctgaaagagaggttggcgagttttgctatgagaagggaacagcgagagagaagcgtttgatcactgacgtcgctattacatcgactgtgaaggacttatttgagagggacgagatcaaactgcttagaatccactgagatttactgaaatttattgtaatttattattactaagctcttaatatttatattacaaaagaatgttgtttgt*ag*aaaaatac**tgc**tcggaaataaatgagaagcca**tgc**tac**tgt**ggg*gt*aaattataagataataaattttatttatcagatgttatcattaataacgtataataccatcattgaacatc*ag*aatgaaactgaa**tgc**gatctgtatcatatt**tgt**gatacacggacaaacaaa**tgc**ataa*gt*aattttcttataataacatttgttattaatgtttgttattaattttatttttacaaaaataatgttgtaatttattattaatactatgctcttaatatttataataaaaataaaagttgattgt*ag*aaaattac**tgt**tcggaaaattcaactgactggtgg**tgc**tac**tgt**acg*gt*aaattattagataataacctttagttacgaaatgttatcataaataatatatcatcatgtatcatgtttgaataatatcatcattgaacattc*ag*gagaatcgtagc**tgt**cgtatatatgaaaaa**tgt**gaatttaattctgaaggaaacaaa**tgc**atatttg*gt*aattatttataatgaattttaataataattttacttatgttgttaattttatttttattattaatttcaatttattgtttttaatttcatgttcttattttttatattaaaattatatgaatgttggttgt*ag*aaaaa**tgt**aaggatagaaatggatatcct**tgt**tac**tgt**gtg*gt*aaatttagaaataataactgtcagttaccaaatgttatcataaataacgtataatatcatcattgaacattc*ag*aatgactctgaagta**tgt**gggaaatatcaaaaa**tgt**gaagtgaattctgaaggaaacaaa**tgc**atag*gt*aatttttataagttattaattttatatttttattaattttaattttgattattattttttttatattattaattttatttttattatgaataatattgtaatttattattacttattatcctcaacaaaaaagtgataagcaataaaaaaaagtaaggatgaatagatataacattacataccgtaatacattcattcctgtttatttttaaaatgttgttggttgt*ag*agaaatataaacac**tgc**tctgatcgaaatgggagggaacca**tgc**ctt**tgt**ggg*gt*aaattataagattacaacttttagttatcaattgttatcataaataacttataatatcattgaacattc*ag*aacgaaacgcta**tgt**cgtacatatgaagat**tgt**gacctgaattctgagggaatgaaa**tgc**aaaa*gt*aattttcatgcagtaaaatttactattaattttacaaatgttataaattttataataaaacgtgcttaaaaaatgatatgaaattgatggatggatagacggatggatgggtggattgatagagggacggatggatggatggatggaatggatagatggttgggtaaatggacggtcagacggacggatggatggatggacggacggatggatataacattacgtaccgtgatatattcacttctgtgtattttaaaaatcaatgttggttggt*ag*aaagatac**tgc**tctgaaataaatgatcgccat**tgc**gtc**tgt**ggg*gt*aagttataagattaaaacttttatttatcaaatgttatcataaataacgtataatatcatcattgaacattcagg*ag*gatttcatc**tgt**cgttcatatgaaaaa**tgt**gaactgaaatctgaaggaaacaaa**tgc**atatttg*gt*atttttttacaatgaaatttaatatttaaattaataaattaaatttaatttaataatttaataatattttaataatttaattttactaattgtattatgttatgaattatattttttttttattaattttaattttacaaaacttattgtgtaatttattactaatactaagctcttaaacttgattaatt*ag*tagaggaaaacagtgtc**taa**

**atg**ttgaagctgtttgttgtcctcttggctttttttggcatctttctgtctgattgtgagtttgatctgtctcaag

agatttataaacac**tgt**tctgatcgaagtgggaatgaatca**tgc**ctc**tgt**gga

aatggctctaaggta**tgt**ggtcgatatgaagat**tgt**gaaggaaacata**tgc**aaag

aaatttacgatcgc**tgc**tatgttcgcaataagtggcca**tgc**ctt**tgt**ggg

gatgatcaagta**tgt**gataaagatgaaatt**tgt**gaaacaaagataaacaaa**tgc**aaaa

agattaatattcac**tgc**tctgatcgaaaaggaaatgaaccg**tgc**ctc**tgt**ggg

aatgagactatg**tgt**tatcgatatgaagtt**tgt**gacctgaat**tgt**gaaggaaataaa**tgc**atag

agaagtttgcatcc**tgc**tctgatcgaaatgggaaggtacca**tgc**ttc**tgt**ggg

aatgactccaaggta**tgt**agtcgatatgaatat**tgc**aaagaaaacaca**tgc**atag

tgtattatagaagt**tgc**tctgatcttaataagtggcct**tgc**atc**tgt**gag

gaagaaaga**tgt**cgtttagatgaaatt**tgt**gaactaggagagattaacaaa**tgc**aaaa

gaaaatac**tgt**tcggaaataaatgtgcagcca**tgc**ttc**tgt**gaa

gatgatcatgta**tgt**gatgacgatgaaatt**tgt**gaagtaggagagattaacaaa**tgc**aaaa

aaaaatac**tgc**tcggaaataaatgagaagcca**tgc**tac**tgt**ggg

aatgaaactgaa**tgc**gatctgtatcatatt**tgt**gatacacggacaaacaaa**tgc**ataa

aaaattac**tgt**tcggaaaattcaactgactggtgg**tgc**tac**tgt**acg

gagaatcgtagc**tgt**cgtatatatgaaaaa**tgt**gaatttaattctgaaggaaacaaa**tgc**atatttg

aaaaa**tgt**aaggatagaaatggatatcct**tgt**tac**tgt**gtg

aatgactctgaagta**tgt**gggaaatatcaaaaa**tgt**gaagtgaattctgaaggaaacaaa**tgc**atag

agaaatataaacac**tgc**tctgatcgaaatgggagggaacca**tgc**ctt**tgt**ggg

aacgaaacgcta**tgt**cgtacatatgaagat**tgt**gacctgaattctgagggaatgaaa**tgc**aaaa

aaagatac**tgc**tctgaaataaatgatcgccat**tgc**gtc**tgt**ggg

gaggatttcatc**tgt**cgttcatatgaaaaa**tgt**gaactgaaatctgaaggaaacaaa**tgc**atatttg

tagaggaaaacagtgtc**taa**

MLKLFVVLLAFFGIFLSDC

EFDLSQEIYKH**C**SDRSGNES**C**L**C**GNGSKV**C**GRYED**C**EGNI**C**K

EIYDR**C**YVRNKWP**C**L**C**GDDQV**C**DKDEI**C**ETKINK**C**K

KINIH**C**SDRKGNEP**C**L**C**GNETM**C**YRYEV**C**DLN**C**EGNK**C**I

EKFAS**C**SDRNGKVP**C**F**C**GNDSKV**C**SRYEY**C**KENT**C**I

VYYRS**C**SDLNKWP**C**I**C**EEER**C**RLDEI**C**ELGEINK**C**K

RKY**C**SEINVQP**C**F**C**EDDHV**C**DDDEI**C**EVGEINK**C**K

KKY**C**SEINEKP**C**Y**C**GNETE**C**DLYHI**C**DTRTNK**C**I

KNY**C**SENSTDWW**C**Y**C**TENRS**C**RIYEK**C**EFNSEGNK**C**I

FEK**C**KDRNGYP**C**Y**C**VNDSEV**C**GKYQK**C**EVNSEGNK**C**I

EKYKH**C**SDRNGREP**C**L**C**GNETL**C**RTYED**C**DLNSEGMK**C**K

KRY**C**SEINDRH**C**V**C**GEDFI**C**RSYEK**C**ELKSEGNK **C**IFVEENSV-

Theoretical pI/Mw: **5.10** / 48180.22

**Htia_mHV7 (this study)**

**chromosom 2 position 1480329 - 1483822**

**atg**ttttctttgaagttgtttgttgtcctcttggcttcttttggcatccgtctgtctcaag*gt*gagtgtgatctgagtttctgaacgaaagagtgatatgtaataaaaagtgcttcaccagttattttgggatggatggatataacattacatatcgttatatattaactgctatttattttaaaaatgaattttggttta*ag*ggaattataaaccc**tgc**tctggtcgaaataagtggcca**tgc**ctt**tgt**gag*gt*aaattataagattacaacttttaaataaataatgtcgtcattgaacattc*ag*gatggctctgtc**tgt**gataatgatgaaact**tgt**aaattgatttctgaaggaaacaaa**tgc**aaaa*gt*aacttttatataatacaattatctaataattttacttaatgtaagaaatgtatatcaatatctcttgtgagtcatcagttatgacccacatgcacacaaacacaagttaaaattattgttattattattattattattattattattattattattattattattattattattattattattattattattattattattattattattattattattattattattattattattattattattattattattattattattattattattattattattattattattattattattattattattattattattattattattattattgaaataacccgggaatcctcaaattagtaagtttatcggagcaagtattcaactgaggtaaattgtttgaggatttgaggtacttaagaacacaaatggatatttgggcttctcagtggtctggactactttgtgtgacataatgctgatacatctaagcgcaaaaagatactgaatataatgctcaacgtttacaatattattgttcttgttgttgttgttgtcgtcgtcgtcgttgtggtcgttgtcgtcgtcgtcgtcgttgttgtttgttgttgttgttgttgttgttgttgttgttgtcgttctcacctcagaatgttataagcatgaaatttgtgaactaggggagattaacaaatgcaaaagtaattttcatataataacatttgttattaatgttttttaattattttattttatttgcaactaatatttatattaaaaatgaatgttgtttgt*ag*aaaaaaac**tgt**acagaactaaatcagtctcca**tgc**tac**tgc**aag*gt*aaaatataatataactttcagttattaaatgttatcttaaataacgtataatatcatcattgaacattc*ag*aatgattatgaa**tgt**ggtgaatttcaattt**tgt**gatacaaagacaaaccta**tgc**gaaa*gt*aattctcatattattacgcttgttattaatgctttttattaattttattttaacaaaaattgtattgaaatttattatttctaatatttatatgctcctaatatttatattaaaaatggatgttgtttgt*ag*aaaaatac**tgt**tcgaaaataaatgaacgacaa**tgc**tac**tgt**gag*gt*aaattataacataataaattttagttatcaaatgttatcataaataacgtataatatcatcattgaacattc*ag*ggtgatcatgta**tgt**gatgatggtgaaatt**tgt**gaactaggagagactaacaaa**tgc**aaac*gt*aattttcatataataacatttgttattaatttttgttattaattttatttcaacaaaaattgtattgaaatttattattaatactatgctcttaatatttatattaaaaatgaatgttgtttgt*ag*aaatacac**tgt**tcggaaataaatgaatggcca**tgc**ata**tgt**gag*gt*aaattataagataaaaacttttagttaccaaatgttatcataaataacgtataaaatcatcattgaacattc*ag*tatgattatata**tgt**gatgacgatgaaatt**tgt**gaagtaggagagactaacaaa**tgc**aaaa*gt*aactttcatataataacgcttgttattaatgtttgttaataattttaatttgacaaaaattgtattgacatttattattaatactatgctcttaatatttatattaaaaatgaatgttgtctgt*ag*aaaaatac**tgt**tcggaaataaatacgtatcca**tgc**tac**tgt**gag*gt*aaattataaaataataacttttagttatcaaatgttatcataaataacgtataatatcatcattgaacattc*ag*gatgattatata**tgt**gatgacaatgaaatt**tgt**gaagtaggagagattaacaaa**tgc**aaaa*gt*aattttcatataataacatttgttattaatgtttctttatttattttattttatttattttaaattgtattgacatttattattaaaattatgctcttaatatttatattaaaaatgaaagtggattgt*ag*taaaatac**tgt**tcgaaaataaatgagtttcca**tgc**tac**tgt**gag*gt*aaattttaagataataaatgttatctatcagatgttatcatatataacgtataatatcgtcattgaacattc*ag*gaggatcaaaaa**tgt**aaggaggatgaaatt**tgt**gaactaggagagattatcaaa**tgc**aaaa*gt*aattttcatataacatatgtttttaatgttttttattaattttattgttacaaaaattgtattatattattattaaatttattattaatactatgctcttaataatataataataataatgtatgtcgtcattgttgtgacgccatctgagagtataaaaaggaacaggcctcgggagcgggggagtttttacgggagttttgtacatgagttgaacgatagggattgcgagttgtccgatgttacatgcgaattcggcagccattagcgatatttccgttcttgattctgtttctgaatattctgaataagccaataaagttttctgttcaaattgttcttcttacaaaaactcaacagtcattatgaaaatgaactttcttttttgactattatgctcaacgaaagagtgatatgcaataaaacgggcttcaaaaaagatatggatgagtggatggatggttttgtgcatggatggacggacagatggttggatataacattacatatcgcgatatatccaattatgtttattttaaatatgaatgttggttgt*ag*aaaattataaactc**tgc**tctgatcgtaatgggaatgaacca**tgc**ctc**tgt**ggg*gt*aattgataagattacaacttttatttatcaaacattttcataaataacgtataatatcgtcattgaacattc*ag*ggtgaaagaata**tgc**aatgaaaaggaattt**tgt**gacctggaaattaaccaa**tgc**aaaagaaattttcaaaaaa*gt*aatatttttaaatttacatttttaattatttatctatgatttaatgttagtgttatgttggtagccatctcataccgtttgttattcccaatttct*ag*cagggtattcgtcgttgcaaataaaagaattgaagtt**taa**

agagtgatatgcaataaaacgggcttcaaaaaagatatggatgagtggatggatggttttgtgcatggatggacggacagatggttggatataacattacatatcgcgatatatccaattatgtttattttaaatatgaatgttggttgtagaaaattataaactctgctctgatcgtaatgggaatgaaccatgcctctgtggggtaattgataagattacaacttttatttatcaaacattttcataaataacgtataatatcgtcattgaacattcagggtgaaagaatatgcaatgaaaaggaattttgtgacctggaaattaaccaatgcaaaagaaattttcaaaaaagtaatatttttaaatttacatttttaattatttatctatgatttaatgttagtgttatgttggtagccatctcataccgtttgttattcccaatttctagcagggtattcgtcgttgcaaataaaagaattgaagtt**taa**

acagggtattcgtcgttgcaaataaaagaattgaagtt**taa**

**atg**ttttctttgaagttgtttgttgtcctcttggcttcttttggcatccgtctgtctcaag

ggaattataaaccc**tgc**tctggtcgaaataagtggcca**tgc**ctt**tgt**gag

gatggctctgtc**tgt**gataatgatgaaact**tgt**aaattgatttctgaaggaaacaaa**tgc**aaaa

aaaaaaac**tgt**acagaactaaatcagtctcca**tgc**tac**tgc**aag

aatgattatgaa**tgt**ggtgaatttcaattt**tgt**gatacaaagacaaaccta**tgc**gaaa

aaaaatac**tgt**tcgaaaataaatgaacgacaa**tgc**tac**tgt**gag

ggtgatcatgta**tgt**gatgatggtgaaatt**tgt**gaactaggagagactaacaaa**tgc**aaac

aaatacac**tgt**tcggaaataaatgaatggcca**tgc**ata**tgt**gag

tatgattatata**tgt**gatgacgatgaaatt**tgt**gaagtaggagagactaacaaa**tgc**aaaa

aaaaatac**tgt**tcggaaataaatacgtatcca**tgc**tac**tgt**gag

gatgattatata**tgt**gatgacaatgaaatt**tgt**gaagtaggagagattaacaaa**tgc**aaaa

taaaatac**tgt**tcgaaaataaatgagtttcca**tgc**tac**tgt**gag

gaggatcaaaaa**tgt**aaggaggatgaaatt**tgt**gaactaggagagattatcaaa**tgc**aaaa

aaaattataaactc**tgc**tctgatcgtaatgggaatgaacca**tgc**ctc**tgt**ggg

ggtgaaagaata**tgc**aatgaaaaggaattt**tgt**gacctggaaattaaccaa**tgc**aaaagaaattttcaaaaaaA

cagggtattcgtcgttgcaaataaaagaattgaagtt**taa**

MFSLKLFVVLLASFGIRLSQGN

YKP**C**SGRNKWP**C**L**C**EDGSV**C**DNDET**C**KLISEGNK**C**K

KKN**C**TELNQSP**C**Y**C**KNDYE**C**GEFQF**C**DTKTNL**C**E

KKY**C**SKINERQ**C**Y**C**EGDHV**C**DDGEI**C**ELGETNK**C**K

QIH**C**SEINEWP**C**I**C**EYDYI**C**DDDEI**C**EVGETNK**C**K

KKY**C**SEINTYP**C**Y**C**EDDYI**C**DDNEI**C**EVGEINK**C**K

IKY**C**SKINEFP**C**Y**C**EEDQK**C**KEDEI**C**ELGEIIK**C**K

KNYKL**C**SDRNGNEP**C**L**C**GGERI**C**NEKEF**C**DLEINQ **C**KRNFQKNRVFVVANKRIEV-

Theoretical pI/Mw: **4.76** / 30888.89

**Htia_mHV8 (this study)**

**chromosom 2 position 1492096 - 1500774**

**atg**ttttctttgaagttgttagttgtcctcttggcttcttttggcatccgtctgtctcaag*gt*gagtgtgatctgagtttctgaacgaaagagtgatatgtaataaaaagtgcttcaccagttattttgggatggatggatataacattacatatcgtgatatattaactgctatttattttaaaaatgaattttggttta*ag*ggaattataaaccc**tgc**tctggtcaaaataagtggcca**tgc**ctt**tgt**gag*gt*aaattataagattacaacttttaaataaataatgtcgtcattgaacattc*ag*gatggctctgtc**tgt**gataataatgaaact**tgt**aaattgatttctgaaggaaacaaa**tgc**agaa*gt*aatttgtatttaataaaattaactattaattttacttaatgtaagaaatgtatataaaatatctcttgtttatcataagttatgacccacatgcacacaaacacaagttaaaattattattattatcattattatttttttttttttttaagacttttggctaagtttattttcccgaacaaaacaatcttaacatccgataaataatcagtgtgaaaaaaacccccaaaaaaatttttaaagtccctcaccatggctcgggatctccacgtgacgataagttgaatttaacttaaataaataactgaaactgtgcatgcacgccagatcaaaaaacgaaagcaacacaaaggaaaattatacaaagtaaataattagttctgttgatttcattcaacaactcttcttctgtaagcaacagtgtggaaggcaccaggttctcctctgatcctcacaacccacttcttgcttcctccctttaagttgatctcttttaatttgtcttcaaacaattttttttttaatctccctgtcttcctttgacaaatcatggctaagaattactttgttgaagacttcactgtctttcaatttgttgctttattattctgtcttctttgcctctcattgtttatttctatattatactttgtgtctgtcacagagccttgaacctttgtgatcaagtgatcaaccttgttttcaaataaacttgaccataatttggtttcttgcttgttttcctctaactttgttacagacactttatttagttccttcaagttttgttgaagattttctatttgctttccttgctccattatagcatttaccaatttatctgttgtcctgataactgttttgagctctttaacatctttagacaagattaaattcttcctttgtaacttttttattttcatctgcgaatttcttcaggcaatcctcgttcaccttcagacaattttcacataagtggaaacctaggatttcggccgtaatcttcgattcgcagctggcctctttcgcacttctcgttctcggcataattttgtcttttgaaaagcaccgagaatatattttgaattagtaacatcaatattttttctcaccgaaattaatttgtcgatttttattcgcagaattttaacgtagaattcgcacaaggtgtttacacgacgataattattattattattattattattattattattattattattattattattattattattattattattattattattattattattattattattattattattattattattattattgttgttgttgttgttattgaaataatccggggtccgcaaattagtaagtttaactaataagtttattgaagcaagtattcagttaactggactgaataaccaaagttaaatttttaaggatttgaggtacataaggacgcaaatggatatttgggcttctaagttgtctggactactttgacataatgctgatacgactatgtgcaaaatgatactgaatataattctacaacgtttataacattattgttattgttgttgttattgttgttgtcgttgtcgttgtcacctcatctgggcgatccggaacatccgtccgttactttccattgatgtagcttctaccaccgcacgctgccttgtctgtctcgcctatactaatataaatctcttctctatagcacatccgctcttttaattaaatccttgcaacgcgtccaaaacaagcttgccaagcttgttctcttcaaccctaccctcagtaacacggactgcctgaagagattgcactggttgccgattcatagacgtatcaccttcaaaatcgccctcatgacatattacatactagcaacatccattccacctcatctccatcacctaatagatcgccgccaagcacaagtcctggttcatcgtcaaccactccacttcaccagccggtacttatataatccctcccaaacagaggcttctcgcaagcctctccagctgtcttgaacagacttcctcccaccgtcagaacccagccatccctggagctcttcaagcgccacctgaagacttcttcagtacttcatgggacagcgaagacacatatgaaaactgatgctatgcatatggcgctttataaattaaatattgttattattttttaaattttattttttcaaaaattgtattgaaatttattattagtgctatgctagtaatatttatattaaaaatgaatgttgtttgt*ag*aaaaatac**tgt**tcggaaataaatacgtggcca**tgc**tac**tgt**gag*gt*aaattataatataataacttttagttatcaaatgttatcataaataacgtatactaacgtcattgaacattc*ag*ggtgatcataaa**tgt**gaaatatatcaaatt**tgt**ttcctaggagagattaacaaa**tgc**aaag*gt*aattatcatataataacatttgttattaatgttttttaattattttattttattggcaactaatatttatattaaaaatgaacgttgtttgt*ag*aaccaaac**tgt**acagaactaaatcagtctcca**tgc**tac**tgc**aag*gt*aaaatataatataactttcagttattaaatgttatcataaataacgtataatatcatcattgaacattc*ag*aatgattatgaa**tgt**ggtgaatttcaattt**tgt**gatacaaagacaaaccta**tgc**gaaa*gt*aattctcatattattacgcttgttattaatgttttttattaattttattttaacaaaaattttattgaaatttattattaaaactatgctcctaatatttatattaaaatgaatgtttgt*ag*acaaatac**tgt**tcagaaataattaagatgcca**tgc**tac**tgt**ggg*gt*aaattataacataataacttttagttaccaaatgttttcataaataacgtttaatatcatcattgaaaattc*ag*gctgatactcta**tgt**gatgctggtgaaaag**tgt**gtactagagactaatgat**tgc**caaa*gt*aattttcatataataacatttgttatttatgttttttattaattctattttttcaaaaattgtattgaaatttattattaataatatgctcttaatatttatattaaaaatgaatgttgtttgt*ag*aaatacac**tgt**tcggaattaaatgaatggcca**tgc**ata**tgt**gag*gt*aaattataacataataacttttagttgtcaaatgttatcttaaataacgtatactatcgtcattgaacattc*ag*ggtgaagaaata**tgt**gagtacgatgaaatt**tgt**aaactaggagagaataacaca**tgc**acaa*gt*aattttcttatcataacatttgttattaatgttttttatttattttaatttatttattttaaattgtattgaaatttattattaaaactcttaatatttatattaaaaatgaatgttgttcgt*ag*agaaatac**tgt**tcggaattaaatacgtttcca**tgc**tac**tgt**cag*gt*aaattataagataataaattttatctatcagatgttatcataaataacttataatatcatcattaaacattc*ag*gaggatcataaa**tgt**caggaatatgaaatt**tgt**gatctaagagagattatcaaa**tgc**aaaa*gt*aattttcatatcataatatttgttattaatgtttcttattaattttattcttacaaaaattgtattgaaatttattattaatactatgctcttaataatatattaataatgtatgtcgtcattatgaaaaatgaactttcttttttacctattatgctcaacgaaagagtgatatgcaataaaaaatgttattttgggatggatggatataacattacatatcgtgatatattaacttatattttttttaaaatgaattttggttta*ag*ggaattataaaccc**tgc**tctggtcaaaataagtggcca**tgc**ctt**tgt**gag*gt*aaattataagattacaacttttaaataaataatgtcgtcattgaacattc*ag*gatggctctgtc**tgt**gataataatgaaact**tgt**aaattgatttctgaaggaaacaaa**tgc**agaa*gt*aatttgtatttaataaaattaactattaattttacttaatgtaagaaatgtatataaaatatctcttgtttatcataagttatgacccacatgcacacaaacacaagttaaaattattattattatcattattatttttttttttttttaagacttttggctaagtttattttcccgaacaaaacaatcttaacatccgataaataatcagtgtgaaaaaaacccccaaaaaaatttttaaagtccctcaccatggctcgggatctccacgtgacgataagttgaatttaacttaaataaataactgaaactgtgcatgcacgccagatcaaaaaacgaaagcaacacaaaggaaaattatacaaagtaaataattagttctgttgatttcattcaacaactcttcttctgtaagcaacagtgtggaaggcaccaggttctcctctgatcctcacaacccacttcttgcttcctccctttaagttgatctcttttaatttgtcttcaaacaattttttttaatctccctgtcttcctttgacaaatcatggctaagaattactttgttgaagacttcactgtctttcaatttgttgctttattattctgtcttctttgcctctcattgtttatttctatattatactttgtgtctgtcacagagccttgaacctttgtgatcaagtgatcaaccttgttttcaaataaacttgaccataatttggtttcttgcttgttttcctctaactttgttacagacactttatttagttccttcaagttttgttgaagattttctatttgctttccttgctccattatagcatttaccaatttatctgttgtcctgataactgttttgagctctttaacatctttagacaagattaaattcttcctttgtaacttttttattttcatctgcgaatttcttcaggcaatcctcgttcaccttcagacaattttcacataagtggaaacctaggatttcggccgtaatcttcgattcgcagctggcctctttcgcacttctcgttctcggcataattttgtcttttgaaaagcaccgagaatatattttgaattagtaacatcaatattttttctcaccgaaattaatttgtcgatttttattcgcagaattttaacgtagaattcgcacaaggtgtttacacgacgataattattattattattattattattattattattattattattattattattattattattattattattattattattattattattattattattattattattattattattattattattattattgttgttgttgttgttattgaaataatccgggggtccgcaaattagtaagtttaactaataagtttattgaagcaagtattcagttaactggactgaataaccaaagttaaatttttaaggatttgaggtacataaggacgcaaatggatatttgggcttctaagttgtctggactactttgacataatgctgatacgactatgtgcaaaatgatactgaatataattctacaacgtttataacattattgttattgttgttgttattgttgttgtcgttgtcgttgtcacctcatctgggcgatccggaacatccgtccgttactttccattgatgtagcttctaccaccgcacgctgccttgttctgtctcgcctatactaatataaatctcttctctatagcacatccgctcttttaattaaatccttgcaacgcgtccaaaacaagcttgccaagcttgttctcttcaaccctaccctcagtaacacggactgcctgaagagattgcactggttgccgattcatagacgtatcaccttcaaaatcgccctcatgacatattacatactagcaacatccattccacctcatctccatcacctaatagatcgccgccaagcacaagtcctggttcatcgtcaaccactccacttcaccagccggtacttatataatccctcccaaacagaggcttctcgcaagcctctccagctatcttgaacagacttcctcccaccgtcagaacccagccatccctggagctcttcaagcgccacctgaagacttcttcagtacttcatgggacagcgaagacacatatgaaaactgatgctatgcatatggcgctttataaattaaatattgttattattttttaaattttattttttcaaaaattgtattgaaatttattattagtgctatgctagtaatatttatattaaaaatgaatgttgtttgt*ag*aaaaatac**tgt**tcggaaataaatacgtggcca**tgc**tac**tgt**gag*gt*aaattataatataataacttttagttatcaaatgttatcataaataacgtatactaacgtcattgaacattc*ag*ggtgatcataaa**tgt**gaaatatatcaaatt**tgt**ttcctaggagagattaacaaa**tgc**aaag*gt*aattatcatataataacatttgttattaatgttttttaattattttattttattggcaactaatatttatattaaaaatgaacgttgtttgt*ag*aaccaaac**tgt**acagaactaaatcagtctcca**tgc**tac**tgc**aag*gt*aaattataatataactttcagttatcaaatgttatcataaataacgtataatatcatcattgaacattc*ag*aatgattatgaa**tgt**ggtgaatttcaattt**tgt**gatacaaagacaaaccta**tgc**gaaa*gt*aattctcatattattacgcttgttattaatgttttttattaattttattttaacaaaaattttattgaaatttattattaaaactatgctcctaatatttatattaaaatgaatgtttgt*ag*acaaatac**tgt**tcagaaataattaagatgcca**tgc**tac**tgt**ggg*gt*aaattataacataataacttttagttatcaaatgttttcataaataacgtttaatatcatcattgaaaattc*ag*gctgatactcta**tgt**gatgctggtgaaaag**tgt**gtactagagactaatgat**tgc**caaa*gt*aattttcatataataacatttgttatttatgttttttattaattctattttttcaaaaattgtattgaaatttattattaataatatgctcttaatatttatattaaaaatgaatgttgtttgt*ag*aaatacac**tgt**tcggaattaaatgaatggcca**tgc**ata**tgt**gag*gt*aaattataacataataacttttagttgtcaaatgttatcataaataacgtatactatcgtcattgaacattc*ag*gatgatgatgaa**tgt**gaagaggatgaaatt**tgt**gaactaggagagattaacaaa**tgc**aaaa*gt*aattttcatatcataacatttgttattaatgtttttttatttattttaatttatttattttaaattgtattgaaatttattattaaaactcttaatatttatattaaaaatgaatgttgttcgt*ag*agaaatac**tgt**tcggaattaaatacgtttcca**tgc**tac**tgt**cag*gt*aaattataagataataaattttatctatcagatgttatcataaataacttataatatcatcattaaacattcagg*ag*gatcataaa**tgt**caggaatatgaaatt**tgt**gatctaagagagattatcaaa**tgc**aaaa*gt*aattttcatatcataacatttgttattaatttttcttattaattttattcttacaaaaattgtattgaaatttattattaatactatgctcttaataatataataatttttggactatttttggacttttttgactattatgctcaatgaaagagtgatatgcaataaaacgggcttcaaaaaggatatggatgagtggatggatgaatggacggatggatagatggctataacattacataccgtgttatatccacttctgtttatgttaaatatgaatgttggttgtagagatttataaactctgctctgatctttataagtaatttttttatattaaaatttgcaattaatttcagttcatttattaattttgtttttagtattgattttatttttgttagtttttttgttttaaaatacaatgttattattaatttaattcttttgttatcaagttatttttacaaatctaataatgtaattcattattagtattatgctcttaatctggtattaattattagaggcaaacagtgtctaaagtcgaaatttattttttgactc*ag*aggtg**taa**

**atg**ttttctttgaagttgttagttgtcctcttggcttcttttggcatccgtctgtctcaag

ggaattataaaccc**tgc**tctggtcaaaataagtggcca**tgc**ctt**tgt**gag

gatggctctgtc**tgt**gataataatgaaact**tgt**aaattgatttctgaaggaaacaaa**tgc**agaa

aaaaatac**tgt**tcggaaataaatacgtggcca**tgc**tac**tgt**gag

ggtgatcataaa**tgt**gaaatatatcaaatt**tgt**ttcctaggagagattaacaaa**tgc**aaag

aaccaaac**tgt**acagaactaaatcagtctcca**tgc**tac**tgc**aag

aatgattatgaa**tgt**ggtgaatttcaattt**tgt**gatacaaagacaaaccta**tgc**gaaa

acaaatac**tgt**tcagaaataattaagatgcca**tgc**tac**tgt**ggg

gctgatactcta**tgt**gatgctggtgaaaag**tgt**gtactagagactaatgat**tgc**caaa

aaatacac**tgt**tcggaattaaatgaatggcca**tgc**ata**tgt**gag

ggtgaagaaata**tgt**gagtacgatgaaatt**tgt**aaactaggagagaataacaca**tgc**acaa

agaaatac**tgt**tcggaattaaatacgtttcca**tgc**tac**tgt**cag

gaggatcataaa**tgt**caggaatatgaaatt**tgt**gatctaagagagattatcaaa**tgc**aaaa

ggaattataaaccc**tgc**tctggtcaaaataagtggcca**tgc**ctt**tgt**gag

gatggctctgtc**tgt**gataataatgaaact**tgt**aaattgatttctgaaggaaacaaa**tgc**agaa

aaaaatac**tgt**tcggaaataaatacgtggcca**tgc**tac**tgt**gag

ggtgatcataaa**tgt**gaaatatatcaaatt**tgt**ttcctaggagagattaacaaa**tgc**aaag

aaccaaac**tgt**acagaactaaatcagtctcca**tgc**tac**tgc**aag

aatgattatgaa**tgt**ggtgaatttcaattt**tgt**gatacaaagacaaaccta**tgc**gaaa

acaaatac**tgt**tcagaaataattaagatgcca**tgc**tac**tgt**ggg

gctgatactcta**tgt**gatgctggtgaaaag**tgt**gtactagagactaatgat**tgc**caaa

aaatacac**tgt**tcggaattaaatgaatggcca**tgc**ata**tgt**gag

gatgatgatgaa**tgt**gaagaggatgaaatt**tgt**gaactaggagagattaacaaa**tgc**aaaa

agaaatac**tgt**tcggaattaaatacgtttcca**tgc**tac**tgt**cag

gaggatcataaa**tgt**caggaatatgaaatt**tgt**gatctaagagagattatcaaa**tgc**aaaa

aggtg**taa**

MFSLKLLVVLLASFGIRLSQG

NYKP**C**SGQNKWP**C**L**C**EDGSV**C**DNNET**C**KLISEGNK**C**R

KKY**C**SEINTWP**C**Y**C**EGDHK**C**EIYQI**C**FLGEINK**C**K

EPN**C**TELNQSP**C**Y**C**KNDYE**C**GEFQF**C**DTKTNL**C**E

NKY**C**SEIIKMP**C**Y**C**GADTL**C**DAGEK**C**VLETND**C**Q

KIH**C**SELNEWP**C**I**C**EGEEI**C**EYDEI**C**KLGENNT**C**T

KKY**C**SELNTFP**C**Y**C**QEDHK**C**QEYEI**C**DLREIIK**C**KR

NYKP**C**SGQNKWP**C**L**C**EDGSV**C**DNNET**C**KLISEGNK**C**R

KKY**C**SEINTWP**C**Y**C**EGDHK**C**EIYQI**C**FLGEINK**C**K

EPN**C**TELNQSP**C**Y**C**KNDYE**C**GEFQF**C**DTKTNL**C**E

NKY**C**SEIIKMP**C**Y**C**GADTL**C**DAGEK**C**VLETND**C**Q

KIH**C**SELNEWP**C**I**C**EDDDE**C**EEDEI**C**ELGEINK**C**K

KKY**C**SELNTFP**C**Y**C**QEDHK**C**QEYEI**C**DLREIIK**C**KKV-

Theoretical pI/Mw: **4.65** / 49082.58

**Htia_mHV9 (this study)**

**chromosom 2 position 1505921 - 1514733**

**atg**ttgaagctgtttgtcctcttggcttcttttggcatcggtctgtctgattgcgagtttgatctgtctcaag*gt*tagtgtgatttgagtttctgaacgaaagagtgatatgtaatatttgtactacatattatttcaccaatgattttggaagaatggatggatggatggatggatgaaaggatggatggatataacattacataccgtgacaaattcacttcattttattttaaaaatgaatgttggttgt*ag*aaatttataaacac**tgt**tctgatcgaaatgggactgaacca**tgc**ctc**tgt**ggg*gt*aagttataagatttatttgcaaaatgttatcagaaatataatatgatattataatattaaaattatcataactgaacattc*ag*aatagctctaaggta**tgt**ggtcgatatgaagat**tgt**caaggaaacaaa**tgc**aaag*gt*aatttttaattaattaaatatactattaattttgcttatgtttatggtagatggattgaaggattgatggatggatggatgagtggatttataggcgatttggtttgtggatgaatgcatctgtggtttgataggtggatgggtgatataacattacataccctgacaaattcacttctgtttattctaaaattgttgttggttgt*ag*agattaatattcac**tgc**tctggtcgaaatgggaatgaacca**tgc**ctc**tgt**ggg*gt*aaattataagattacaactattatttacaaaatgttatcataaataacatataattttatattattaatatcttcattgatcattc*ag*aatggctctaaggta**tgt**agtcgatatgaaaat**tgt**gaaggtaacata**tgc**aaag*gt*aatttttaattaatcaaatatactattaattttgcttatgttattaattttatttttgtcatcaatttcacttttgttgaaaatttatttttacataaataatattcattatcaatactgttatgcccaaggaaagaatgataattaataaaaattgtctctccaatgatttggagtggatgagaggatgtatgaagggatggatataacattacatgtcgtgatattttcacttctgtttattataaacataaatgttggatgtagagatttataaactttttatttgtggatggatggatggctctatgaatggatggatggatggaagggtagatggatggatggatgagtgcatggatgggtgatttgatttgtggatgaatgcatttgtgctttgatggatggatggatgatataacattacataccctgataaattcacttctgtttattctaaaaatgttgttggttgt*ag*agattaatattcac**tgc**tctgatcgaattgggaatgaacca**tgc**ctc**tgt**ggg*gt*aaattataagattacaactattatttacaaaatattatcattaataacgtataattttatattattaatatcttcattgatcattc*ag*aatggctctaaggta**tgt**ggtcgatatgaaaat**tgt**gaagggaacata**tgc**aaaa*gt*aatttttattaattttatttttgttattaatttatttttattaacaatttcattttttaaaattttatcatctctctcaacgtgagtgtaatttgagtttctcaatgataaagttatatttaacaacaactgcttcaccagttatttgggatggatgtatggatggatggatataaaattatttaccgtgatatattcaattctgtttattgttaaaactgaatgttgtttgt*ag*agaactttacagtc**tgt**tctattgaaaatgacgatcca**tgc**ctc**tgt**gga*gt*aagttattagattataattatttgttatcaaatgttgaattacgtgtaatatcatcaatgaacattc*ag*gataaaaga**tgt**ctttttgatgaaatt**tgt**gatttagagacaaacaaa**tgc**caaa*gt*aagctaatgaaatttgttattaatgtttttctattaattttattttaacaatattattattgtaatttattattattattactatggtctattattattattattattattattattattattattattattattattattattattattattattattactaaaaatgaaagttgattgc*ag*aaaaatac**tgt**tcggtaataaatgagcagcca**tgc**tac**tgt**gaa*gt*aaattataaataataacttttatttaccaaatgttatcataaataacgtataatatcatcattgaacattc*ag*ggtgaagaaata**tgt**gagtacgatgaaatt**tgt**gaagtaggagaggtgaacaaa**tgc**aaaa*gt*aattttcaaataataacatttgttattaatgttttttaccatgtttacttttgcaataatagtattgaaatttgttattaatactatgctcttaatatttatgttaaaaattaatttttttgt*ag*aaaaatac**tgc**tcaatatcaagtcggtggcca**tgc**tac**tgt**acg*gt*aaattataagataatgacttttatttatcaattgttatcataaataacgtataatatcatcatcgaacattc*ag*aatgatactgta**tgc**catccaagtcaaaca**tgt**gaacagaattctgaaggaaacaaa**tgc**gtatttg*gt*atttagtaacaatgaaatttaataataattttacttatattattacttttatatttattttcatttcaattttttgttaataatttttttttaaactttaatttattatttatattaaaaatgattgttggttgt*ag*aaaaa**tgc**tcggaaatatttgcatatcct**tgc**ctc**tgt**gtg*gt*aaattataagataataacttttaattatcaaatgttatcataaataacgtataatatcatcattgaacattc*ag*aattactctgaaata**tgt**ggtgaaaatcaagag**tgt**gaagtgaattctgaaggaaacaaa**tgc**atag*gt*aatttatttatttaataatatttataagttaataattttatatttttattcatttcattttttttattcatttttttttacataaatgatattgaaatttatttgaacaattcaaaaagctagtattgtcgactgcgccactgtttaatttacgtattgaatgctggagaaataactcgtaactgtaataaaacaactttattacaaaactcgaatcgaaagcgaaaaacaaattttaggtcaagtagccctcaatattcatgccgacacagcaattaatactattatcctcaacgaaaggtgatatgtaataaaacgtgcgtcaccatttattttgggatggatggatggatggatggatggatggatggatggatggatggatggatggatggatggatggatggatggatggatggatggatggatggatggatggatggatggatggatggatggatggatggagggatggatggatggatggatggatggatggatggatggatggatgggtggatggatggatggatggatggatggatggatggatggatggatggatggatggatggatggatggatggatggatggatggatggatggatggatggatggatggatggatggatggatggatggatggatggatggattgatggattgatggattgatggattgatggatataacataacataacataaggtgataaatccacttctgtttatttaaaaattgaatgtggttgtagaggtttatgaacactgctaaataaataaataccgtaatacattcattcctgtttatatttaaaatgttgttggttgt*ag*agaaatataaacac**tgc**tctgatccaattgggagggaacca**tgc**cta**tgt**ggg*gt*aaaatatacgattgcaatttttatttatcatatattatcataaataacatataatatcaccattgaacattc*ag*ggtacaaatttg**tgt**tatgaatttcaatat**tgt**gacctgaattctgaaggaaacgaa**tgc**aaat*gt*aagttttatgcaataaaatttactattgattttacaaatgttattaattttatttttattattattttttaaattacattttcacaaatattattgtaatttatttttaaaactattatgctcagcgaaagagtgatatgtaataaaacgtgctcaaaactgatgtgggattgatggacggatataacattaaataccacgatacatattcgcttcggtttatttgaaaaatgaattttggtttt*ag*ggaagtataaagac**tgc**tctgatcgaagtgggtggcca**tgc**cga**tgt**ggg*gt*aaattataagattacaacttttagttattaaatgttatcataaataacatttaatatcatcattgaacattc*ag*aaggaaactgta**tgt**tatcaacatgaatat**tgt**gacctgaattctgaaggaaacaaa**tgc**aaaa*gt*aatttttatgcaataaaatttactattaattttacaaatgttataaattttatgttttttattaattttttatgttattaattttatttttattatcatatttttatttattaaatttatacaaataatgtcattgcttgaaccctaaaatggatcaacctacaagaataacaccaaagagcgctaccattatcgataacattttcacgaacgtattcgacagaaacagtttttcaaaaattctagtaaacgacatttcggatcatcttcccatctacttctcgttcagtgctggtgttcttaattactgcagatcaatttcttcacccattagaatgcatacagaggaaggcattgctagatttattgtagagattgagggaatggattggtctgatattctgggttattgcaagactctggaggtttctaaagcataccaatcctttgtaaatgtatactctgaagtctataacagatgtttcccaatttctcctcatcgtacaaatcattatcgcttcaaaaaaccctggatgaccccgggacacttaaattcgcgtaataaaataaacaatctctataaaaagttcttgaaatatttgaaaataatgtcgtcaagaactcctgtaccgaggtggaatcacttgttacgtccaaattgaaatatcccaacaataggcaatctccaattttcctgatcttatcaagcgtgaccttgaattcctcattaaatagtttcttttaggacaccgagtgttatcacagtgttgcgtaactttacatttacaaacacgttttcaaaggtcgcatcagaaaaatgatgttttattgaacaagatacaatatcccgatgaataaaaattccaactcctcctctcttttctgatcgacttgttgcaataaatgattagccaaggatacaaatggaaacacaatgactaccatttaaccaagtttcagtctgcaatttctgcaattatattggcctttatcctattattcaacacgttgatggcgtgtattttgtttggtaagcttctacaatttatctggagaaatgagatattatttggaatttttgcagcccatcaaagccttcaggtgttatatatgaatgtgaaaattctttatttataaaattataatctggattaatatcttctaattgaaaaacggaatctcctttatcaaaataaaaatccggaacagaagccgtcttcgtggggtaaacataaatttcttgacttcgatcagcttctccagccccttcagatcctgtatccgaacgggcttcaaagattcggttctgctgagatacactaagcctttccaagtccagactgctgccatttttttcctttcttcgttgacgaggacttgtgagttgccgtgtgtttcacgtagtgaaaacctggcgtgcaggtaagccgaatgggttatgtgcttgcacatggaggtaatggcaacagtcgttcgaaacccagtctgcactgccgccagcgcccttgggaaggagcgtatataaatggttaaaagaagcgcctctgacgctatgcatatggcgctatataaattaactaactacaattacgtagtgcgttttatgctgcgtacaccttatctcggactcctttaactgagaatgtcacaacggtaggacggggacccttactggtagatattcgataacaattgacgatgtcattgatggtgacgccaagtttcatccccgacctacaacagtctattacagcacggattgtgtcctgtctaacaacccctccagcttcctcagaagtcgatgctgcctcagcatatgatgcttcaggtaacccatatatcacgaggttatttgatctggattgcttttccagattgtaaaccctgatttttagaattcggttttattcaactgttttgttgaatccttctgtcatagacctcagttcacttctgacagccttgggaaccacatttgcaacttcctgtttaatgatctctctcatcattctttcgatactgggtattaagtttgctttcactttctcagcaaactcatcgctgtctatcgtagattccagttttcgaaacattttgtgagattaggagtggttcgtagtcagtattgggtccaaattcataatcgaagtattcagcaaaagaaaacgaatccaaacgtatgctttgttttgtcgaatttagtagtagtaaactaagttattttaatttaaggcgcgagaatattactagcacgttcatctcgagttcattgttaccatggatggatggatggatggatggatggatggatggatggatggatggatggatggatggatggatggatggatggatggatggatggatggatggatggatggatggatggatggatggatggatggatggatggatggatggatggatggatggatggatggacggacggacggacggacggacggacggacggacggacggacggacggacggacggacggacggatggatggatgactggatggttggatgactggatggttggatggatggatgggtggattgatgggtggatggatggatggatataatttcacttctgtttattttaaaaacgaatgctggttgt*ag*agattaattttagtctc**tgc**tctgaaagtaataagtatcca**tgc**cgc**tgt**gag*gt*aagttataagattgcaacttttagttatcaaatgttatcataaataacgtataatatcgtcattgaacattc*ag*ggtgataatata**tgc**agtgaatatgaatta**tgt**gacctgggaattaacaaa**tgc**aaaa*gt*aattttcatacaattaaatttatcattaatttttattaatttcaatatatattgttgtaaatgcttaaacgcgacagtaactatttaaaattatattgccaaaaaataattacatctaaatttaaatgttttttgtggtagtattcatcatcgtattctgcatagtcgtatcagcattatgacatacaaaacagagttccgaagcagttcagatgcagttcctgttccgaaccattaatcagagaatccaacaaagcctaacattctctatacttaaaccctcatcttgattattcagttctaataactgtaataccttaataaacttactctaagactttctagttcacaaaccccctggttatttaaatatatatatataataaaaatgaatgtttattgt*ag*aaaaatac**tgt**tcggaaagaaatgaatatcct**tgc**ttc**tgt**gataaagatcatagg*gt*aataacttttatttatcagatgttatcataaataacgtataatatcgtcattatacattc*ag*ggtgatatttcc**tgt**agccgaactggaatt**tgt**gaactgaatcctgaaggaaataaa**tgc**aaaa*gt*aagtttttatttaataaattttatttatttgtaatatattgtatattctattctatttaatttgattttaattatgttatttattctatttttgtttataattatttttaggttattaattttattttttgttaaaagttttattttttctaaatataatattgtaatttatttattaatttgctcttaatatttatattaaaaatgaaagtggattgt*ag*aaaaatac**tgt**tcgaaaataaatgagcttcca**tgc**ttc**tgt**gag*gt*aaattttaagataataaatttatctatctatcatataaattttaattttatctatcagatgttatctcaaacctatacatatatatatatatatattgaacattc*ag*ggtgatcgtata**tgt**caggacggtgaaatt**tgt**gaactaggagagaaaaacaaa**tgc**aaaa*gt*atttctcatataataaaatttgttattaatgttttttatttttttatttttacaataatagtattgtaatatattattaatactatgctcttaatatttatattaaaaatgaattttggttgt*ag*aaaaatac**tgt**tcggaaataaatgagtggcca**tgc**ttc**tgt**tta*gt*aagttataataacttttatttatcagatgttatcataaataacatataaaatcatcattc*ag*agtgatagtatt**tgt**cgtccatatgaaaaa**tgt**gaactgaattctgaagggaacaaa**tgc**atatttg*gt*aaatttttacaatgaaattaaatattttaataataatttaataatattttaataatttaattttacttatttttttatgttagtaatttcatttttattattaacttcaatttttttattaattttaattttacaaatatatttttttaatttattactaatgctaggatcttaatctggtattaattaatagaggcaaacagtatctaaagttgaaatttgtttttttgactc*ag*aggtg**taa**

**atg**ttgaagctgtttgtcctcttggcttcttttggcatcggtctgtctgattgcgagtttgatctgtctcaag

aaatttataaacac**tgt**tctgatcgaaatgggactgaacca**tgc**ctc**tgt**ggg

aatagctctaaggta**tgt**ggtcgatatgaagat**tgt**caaggaaacaaa**tgc**aaag

agattaatattcac**tgc**tctggtcgaaatgggaatgaacca**tgc**ctc**tgt**ggg

aatggctctaaggta**tgt**agtcgatatgaaaat**tgt**gaaggtaacata**tgc**aaag

agattaatattcac**tgc**tctgatcgaattgggaatgaacca**tgc**ctc**tgt**ggg

aatggctctaaggta**tgt**ggtcgatatgaaaat**tgt**gaagggaacata**tgc**aaaa

agaactttacagtc**tgt**tctattgaaaatgacgatcca**tgc**ctc**tgt**gga

gataaaaga**tgt**ctttttgatgaaatt**tgt**gatttagagacaaacaaa**tgc**caaa

aaaaatac**tgt**tcggtaataaatgagcagcca**tgc**tac**tgt**gaa

ggtgaagaaata**tgt**gagtacgatgaaatt**tgt**gaagtaggagaggtgaacaaa**tgc**aaaa

aaaaatac**tgc**tcaatatcaagtcggtggcca**tgc**tac**tgt**acg

aatgatactgta**tgc**catccaagtcaaaca**tgt**gaacagaattctgaaggaaacaaa**tgc**gtatttg

aaaaa**tgc**tcggaaatatttgcatatcct**tgc**ctc**tgt**gtg

aattactctgaaata**tgt**ggtgaaaatcaagag**tgt**gaagtgaattctgaaggaaacaaa**tgc**atag

agaaatataaacac**tgc**tctgatccaattgggagggaacca**tgc**cta**tgt**ggg

ggtacaaatttg**tgt**tatgaatttcaatat**tgt**gacctgaattctgaaggaaacgaa**tgc**aaat

ggaagtataaagac**tgc**tctgatcgaagtgggtggcca**tgc**cga**tgt**ggg

aaggaaactgta**tgt**tatcaacatgaatat**tgt**gacctgaattctgaaggaaacaaa**tgc**aaaa

agattaattttagtctc**tgc**tctgaaagtaataagtatcca**tgc**cgc**tgt**gag

ggtgataatata**tgc**agtgaatatgaatta**tgt**gacctgggaattaacaaa**tgc**aaaa

aaaaatac**tgt**tcggaaagaaatgaatatcct**tgc**ttc**tgt**gataaagatcatagg

ggtgatatttcc**tgt**agccgaactggaatt**tgt**gaactgaatcctgaaggaaataaa**tgc**aaaa

aaaaatac**tgt**tcgaaaataaatgagcttcca**tgc**ttc**tgt**gag

ggtgatcgtata**tgt**caggacggtgaaatt**tgt**gaactaggagagaaaaacaaa**tgc**aaaa

aaaaatac**tgt**tcggaaataaatgagtggcca**tgc**ttc**tgt**tta

agtgatagtatt**tgt**cgtccatatgaaaaa**tgt**gaactgaattctgaagggaacaaa**tgc**atatttg

aggtg**taa**

MLKLFVLLASFGIGLSDC

EFDLSQEIYKH**C**SDRNGTEP**C**L**C**GNSSKV**C**GRYED**C**QGNK**C**K

EINIH**C**SGRNGNEP**C**L**C**GNGSKV**C**SRYEN**C**EGNI**C**K

EINIH**C**SDRIGNEP**C**L**C**GNGSKV**C**GRYEN**C**EGNI**C**K

KNFTV**C**SIENDDP**C**L**C**GDKR**C**LFDEI**C**DLETNK**C**Q

KKY**C**SVINEQP**C**Y**C**EGEEI**C**EYDEI**C**EVGEVNK**C**K

KKY**C**SISSRWP**C**Y**C**TNDTV**C**HPSQT**C**EQNSEGNK**C**V

FEK**C**SEIFAYP**C**L**C**VNYSEI**C**GENQE**C**EVNSEGNK**C**I

EKYKH**C**SDPIGREP**C**L**C**GGTNL**C**YEFQY**C**DLNSEGNE**C**K

WKYKD**C**SDRSGWP**C**R**C**GKETV**C**YQHEY**C**DLNSEGNK**C**K

KINFSL**C**SESNKYP**C**R**C**EGDNI**C**SEYEL**C**DLGINK**C**K

KKY**C**SERNEYP**C**F**C**DKDHRGDIS**C**SRTGI**C**ELNPEGNK**C**K

KKY**C**SKINELP**C**F**C**EGDRI**C**QDGEI**C**ELGEKNK**C**KKKY**C**S

EINEWP**C**F**C**LSDSI**C**RPYEK**C**ELNSEGNK**C**IFEV-

Theoretical pI/Mw: **5.10** / 55204.16

**Htia_mHV10 (this study)**

**chromosom 2 position 1519970 - 1540854**

**atg**tcgaagctgtttgttgtcctcttggcttcttttggcatcggtctgtctgattgcgagtttgatctgtctcaag*gt*tagtgtgatttgagtttctgaacgaaagagtgatatgtaatatttgtactacatattatttcaccaatgattttggaagaatggatggatggatggatggatgaaaggatggatggatataacattacataccgtgacaaattcacttcattttattttaaaaatgaatgttggttgt*ag*agatttataaacac**tgt**tctgatcgaaatgggactgaacca**tgc**ctc**tgt**ggg*gt*aagttataagatttatttacaaaatgttatcagaaatataatatgatattaaaaatattaaaaatatcataactgaacattc*ag*aatagctctaaggta**tgt**ggtcgatatgaagat**tgt**gaagggaacata**tgc**aaag*gt*aatttttaattaatcaaatatactattaattttgcttatgttattaattttatttttgtcaacaatttcacttttgtttaaaaattatttttacataaataatattcattatcaatactgttatgcccaaggaaagaatgataattaataaaaattgtctcaccaatgatttggagtggatgaaaggatgtatgaagggatggatataacattacatgtcgtgatattttcacttctgtttattatagacatgattgttgagatttataaacattgtatttgtagattgatggatggatggatggatggatagatctaaaattacataccgtgataaattcacttctgtttattctaaaaatgttgttggttgt*ag*agattaatattcac**tgc**tctgatcgaaatgggaatgaacca**tgc**ctc**tgt**ggg*gt*aaattataagattacaactattatttacaaaatgttaacataaataacatataattttatattattatgatctttattgatcattc*ag*aatgactataaggta**tgt**agtcgatatgaagat**tgt**caaagaaacaaa**tgc**aaag*gt*aatatttatgtaataaaatttactactaattttatttttgtcatcaatttcacttttgtttaaaatttatttttgcatgaataatatttgttattaatactgtcatggccaaggaaagaatgataatgaataaaaggtgtgtcaccaatgatttggaatggatgaaatgatatgtgaaattatggctataacattacatatcatgatattttcacttatatatattgtaaacatgaatgttggttgtagagatttataaactttttatttgtggatggatgattggtgggatgagtggatggatggattgatggattatggatggatagatggatgattggatggatggttcggtgggcggatgggtgatttggtttttggatgaatgtatttgtggattgatggatggaaggatggatataacattacataccgtgataaattcacttctgtttattctaaaaatgttgttggttgt*ag*agattaatattcac**tgc**tctgatcgaattgggaatgaacca**tgc**ctc**tgt**ggg*gt*aaattataagattacaactattatttacaaaatattaacataaataacatataagtttatattattaatatcttcattgaacattc*ag*aatggctctaaggta**tgt**agtcgatatgaagat**tgt**gaaagaaacaaa**tgc**aaag*gt*aatattaatatttatgtaataatatgtataaacatgaatgttggttgtacagatttataaactttctatgtgcggatgcatgggtgatttggtttgtggatgaatgcatttgtggattgatggatagatataacattacataccgtgataaaatcacttctgtttattctaaaaatgttgttggttgt*ag*agattaatattcac**tgc**tcagatcgaaattggaatgaacca**tgc**ctc**tgt**ggg*gt*aaattataagattacaacttttagttatcaattgttatcataaataacgtataatattttcattgaacattc*ag*aatgagactata**tgt**tatcgatatgaagtt**tgt**gagcagaattctgaaggaaacaat**tgc**aaag*gt*aattttaatacaataaaatttagttttaatttcacaaaagttattagttacatttttgcagagatgggtagttgctaactataagtagctaactactagttaataattactttttaactactttaaagtagttaataactacttattaaaaatatagttaaaaactagtagttaactaattttaaagtagttagtaactacaaatagttaactactatttaactgcttttaataacattttaaaataaaattttaatttttcaatttggtacatcactatataagctttgtttctttgatttcatagtttgtttatatactctgacttctgaattatcactcttagtagccttatttggaaaacgttttatataaatgatgacatggacatggatgtgccactatgtgatatttaaggaaataatgaaagtcttgttggtgaaagtatagccgatttgattgaaaatgaagaaaatattgataatacggataagcactgcggtaatcattccttaaatcttgtagctagcgtagatgcaatgaaagcaagggataatctgcaatacaaatgattttatgatagatcaatggcaaaggtttcatcattatcaaactcagttcatcgcagtgtaaaaaatgctgaggttgtacaggacattacaggttcaacatttataaagccaaatgttgcaaggtggctctcagaattctattcaattgaaagagttgttgaaataggtttcaatgaagtttgcgtctgccagaaggagttagggcaacagaaactgaatttgctttgttgaaatcgttcatccagattatgaaactgttactgcaatgggtatctttcaaagtgaattcatacattggtcacattattccaaccatcattggcatcaacaaaaaattatcatcttgttcagataaaactgtcaagccctatgattatgtggaatgtatatcaaacccatgtgataatgtgttagctgaacagcttaatgattatttatcaaactcagcaaggaatgtacaaagtctactcaattatccaactgtttgtgctgcattcatcaagcttaattcgacattacctatgttcggcagcagttgaaagactttttagtatagcagggcaggttttatgtaacagacgatgtaaattatcggataatcatatcgtcatgatgatttttttaagagatctcccaacacatccactgtatacttctgttttagaccaatttggacaataaaatttcgttgataatgttataatagtgtaaataaatatgctgataattaagtcttgttcagttgtcctgaaaattaatgttatcaaatgtcaactgaaaagtagtttaaaagtagttactaactacttatttaaaaattagtttaaaatagttactaactacattttcctataagtagttaactaccttttaattagttaaagcccatcgctgcatttttaatatttgaattccaaggaaagaaatgcatgggtaatttttataagttactagatatttaccctgctctgcccggtaatagatttgcaattgaataaactttttttttttttttttttattttatttacaaactgaagggcccaataggaccactaacacaatttggtcaattcgagcaagggacaaatcgagagcatgagtcatggtgacgataacgacgactatgagagtcagttatatataaggtatttgctaagatcaaaatcacgtaggagtgcgttgaatctggaaacagttgttgcattaatgaaatcaggtggcagtgagttccaaggttgaacaatcctattgcaaaaagaatatttaacgatgtcggaagaaaatttgcagtgttttagtttgagattgtgaccccttgtgccgcgataactttgaaaaaccaggaaatgttttagggggagtctacaaaaccctttagcagtttgaagcaaaaggtcaggtcggccaggaggcggcgcttttccaaggtggggagattgaggagtttaagtctatccatataggaatatgatgcgacagaaggtattgatttagtgaatcttttttgaactttttcaattttgtcaatgagaaatatatggtggggattccaggctggtgaactatattcgaggattggtctgacgtaagtgcaaaaggcgagctttaggatttcctggtctttggcagaaaaagacttcctgatcatccaggagatattggcagctttatgagtgatggcttcaatatgggtaacaaactttaaagaaggacttattatgaccccgagatctctaatcatgtctacgctttgaagggcctgaccgttgatggagtatttccggagttcacctgtttttttctgcggctggctatagtgacgatctgacatttatcgggtgcaagttgcaactgccatgtataactccattgtgcgaccttgtcaagagctgattgtaactgtgtttgtgaaggcgttgggtggcaatagagcttaacatcatccgcaaatagtttgcagacaatattagtatctttaaacagatttgggaggtcattgatgtatagtgagaaaagagtaggtccaagtacactgccctgggggacgccgctggtgacagaggccgtatccgatagaattccaccgatgttaacacgttggctcctcaatgataagaagtcggtgatccatcccataatgttgccactgatgccataatgaggtagtttgcttaagagtttgtagtgcgatactgaatcaaaagcctttttgaagtcgagatatgcgacatccaccgactgtctagattggagtgcttccgaccactgttgcatacattcaagaagatttgttacagtagatctgcctctgacaaacccgtgctgatccctgcttattaatttgtggtgcagtaaatggtgcataatcactttgttgatgatcttctccattacacgacaacaagttgatgtgatcgaaataggtctgtagttttctgggagaacagtaggtcctttcttatgtataggagtaaccaatgcaattttccattccgcgggtaatctttgatctttaagggaaatatcgaatatgtgggcaagtggtaaagccagaaatgttgcacatctctttaaaaagatattgggtattccatctggacctgaagatagtgaggagtcaaggctcaacaacatcagtcgcactatatctggtgtgaatctaatttctcctatttctgtttgcttttgaagagaaaattcaggattcttgccatcgtcagttgtaaagactgaggaaaagtagtagttgaggatgtcacctatttctgatgggtcactagtcagggcaccggaaggatgggcgatctcagataagacggcacgggtgtgaagcttactattgacatatctgtggaaccttccaaaatttttgctcctaagtagattaagttccaatgatgcatagtgttgcttgatggatttactacaagttttagcaaaactgtagtatctggtcctatcatcaatacttcgtccgctcctccagatcttccatagcttagattttttattgagcagtttgttgataaaatgaggatgtccgtttttgtgttttcgatcacagagaggaaatttagggataaacatgcttatgacttgatcaatttgatttttgaactctttccaacagtagtccacatttggacagaattgaaatcgtgtgttccagtctatatatgagagatattcattaatcagcatgtagtttcctttcctgaagttgggagacggagacgatggttggtgatataaaggtttgcagagaatgtttgtcttgaaggaaatgacattgtgatcacttgttgagatggggcacagtgcttctatctccgatatgaactgagagtcgtttgacaagacaagatcaaggatgttgtcatggcgtgtggcagatgtaacaaactgatggaacccatagtcattgaagaagtctgaaaacattttgtagatattgttgtttggtactgcatgataagtccaattgaaggcagggagattgaaatctccgagtaagcagatggtgtgttcgattgaacaaagatgataaaaacacttgatactttcattggcgtagtcctcagcctccacgtcaaaacctccataacgatagtagagtattattctgcacgtagaatgacttgttgacaggtcaacacaaacaagttcaatatcggaaaactcattcggaatcttaactactcgatgtgaaaaacaatttctgataattagcatgactccacccccttttgtgtctctgtcccacctaatcacatggtagccattgggacaaaacaatgagttgggggtgtcagaattaagccacgtttctgtgcagcaaaaatatcaggatcctctgagctcaaaaacagaaacacttcattaaacttattgttgcaacttctgagatttgttagaatacacttgcatgtcagatgtctctcatcgaaagggctttgtaggagccacaggttgatctcgagaagctgagcgtctcccaaccctccgaagatatgcttgttctgattgcaagcgtgtcaagtcgcgattgaagaacaccttcctcagctcagggtcgggtgaagctctaagtacccgagatgaagataggattgaatcaacacaacttttgctttcaagagtgacacagagcttagaagtaggacccctgtctctacccaatctcctggtcctaatcactcgaggcttgacggtcagatttagttcacagaattcctcgaatattgtggagtcatttgtgtcaggtctttgtgtaagtcctgagacaatgacatttgttgacctccgctcaatgtcactcttctccttctcgaagtctagtaaggctttggtcgttaattttgcaacagcgtcagcatctgcagttgcatttgttatggtgatgaaccaatgagagttgagcgcgctaagaacacaatggaatatgttctataaatagccttgggctatcgctcagcctctctctctcgtgtagtgaccgtgtattatcgttgtttacttgttaataaaccgtttcacgtttaacttcgtcttcttcggtatcccagagatataacattttggcgacgaggatgggatccgaagataaatttctctctatgatgacgatgttcatggaagaaagtcgtcgtcgcgatgaagaaagtcgtcgtcgcgatgaagaaaatcgacgacgagacgaagaaaacaagaaacgagacgaagaaaacaagaaacgcggcgatgttttgttgagactgctggaggaattgagtctacgttcgtctacacatcgtcaagacgaagaactgagcactcccgtgatcagcaggactcagacggtcaaccacgacctagaatgtggaggcacattcgtcaagaggatcgaactggttgaagacgcagtgtcaacacacggtccacgactgccagagtcggaggaggagagagcaagggtccagagaaacagcagaaggaagcctcgaccagagttccgtagcaacgaatctgaccgaccttcaactgcaaggagatgtttcatctgtgacgagacaactcatctggcaaagagttgtccaaaaaagatgcgagcaaagaagattgacgataacaacaagaaggggaaaagaaaatttgccgagatcgattttggaagcatcaagatcgaaatgcaactggactcgggtgctgatgtaaccatcatcaacacagacacatgggagaagattggagctccagatctggacaagtcttcagtgtcactgtcagctgcaaacggcaccccgatcgaggtacttggttgttttgaggtgcaattccgctgccaagggttcactggccatggtcgctgcttcgtggctaaggatgtggaccagcttctgggcattgagtggctggaccagttgccaccatttgcgaaggccttcaacgccatctgctgtcaagtggaacagagatcagttgaagtaggaggtctgtcccaagagttgtcagacagataccccgaggtcttcaaggaggaactgggccgatgctccatgaagaaggcagaattcagagttaaagaaggaagttgcccgatcttctgtaggccgaggaagattcccttcacgttagagaaagctgtggatgtcgagttagaccgtcttgtgcaagcaggagtcttgaagaaagtagactacagtcagtgggctgcaccgatagtcatcgtgcacaagaagaacgggactgtcaggatctgtgccgacttcaagactggcctgaatgaagcactggagactcaccgacatccactgccgactcctgaagagatcttctctcacctgaaccaaggcgcatggttcacacagattgacttggcggatgcatatctgcagatggaagtcaatgaagactcaaaggaactggtgacgatcaacacgcacagggggctgttccagtaccagcgactaccctttggagtcaagtgcgcacccggcattttccaagaagctatggatggcatgctcgccggattacaaggttgtgcagcgtacctggacgacatcatcgtgagtggtacaacattggaggagcacaaccggaacgtccatgcccttttcaagaggatcgcagagtgtggatttcgtgtccgaatggagaagtgttcgttcgcgaagaaggagatcaagtttctgggaaatctgattagcaaggatggacgtcgaccagacccagagaaaatccacgcgatcgccgaaatgccgccgcccaaggacaagaagcagttgaaatccttcttgggcatgataagtttctattcgtctttcgttccagagatgcgatccatgagaggacttctggatgacctggagaaggaagacaactttttgtggactgctgagcatctgggggccttcaacaaattgaagacggtgctacagtcggatctgctagtgacacatttcagacccgaaatggatatcgtggttgttgctgacgcatgtgagtatggattgggtgcggtgatctcacaccgatttccgaacggcacggaaaaggccatagcacatgcaggaagaagcctgacaaaagcagagcagaactacgggcagatcgagaaagaagctctttcactggttttcgctgttagaaaatttcaccgatacctgtatggacgacgcttcacgctgttgacggaccataaaccattgctgtctattttcggatcaaagacaggagtgtccgctcattcagccaatcgacttcaaagatgggagctttcattacttagttatgatttcagtattgagtacaggaagacggaccactttggacaagccgatgcactatcccgactcattgcttcaaagttgccagaacctgaggatgtcatcatcgcaaagattgagaaggacattcaagcacttcgaagtgatgtgattcgccagttgccagtaacgcacacggacatccggaagatgaccgaaagtgatgagctacagatcattgtgcaagccgtgctatcaggacgttggccagattttcggactggatcgttgatgcatgccttccacagtcgtgctaaagacctgtcagtgcatgatggagttctttttcttggaatgcgagcagttgttcctttggctctgcgacatcgtgttctgaaaatgctccatgaaggacacccaggatgcacacggatgaaactgatagctcgaaggtatgtttattggcagggcattgatagggatattgaagagcaggtgaagaattgcagcgcttgtcaggatgctgcaaaaatgcctgcaaggaatgaaccgtcgccatggccaacaccgaattgtgcatgggagaggatccacgtcgattttgccggacccctggaaggattgatgttcctcatagtagtggatgcctactccaagtggcctgaagtagttcagatgtcatcaacaacagccactggtacaattaaagagttaactaggatttttgctcagcatggatttcctaaagtactcgtgtcggacaatgggacgcagttcacatctcaggagttccagagttactgtcagcagtatggtattcagcacattcgttcacctccatatcacccgcagagtaatggccaggccgaacgtttcgtggacaccttcaagaggacttttcagaagttgagaggggagggagccacatcagatgtgattcagaagtttctgtggacttacagatcgactccatgtttgtcttccccaggccagaagacacctgcagaaaactgcattgggcggcagttgaggactccggtaagtgatctacagttgcagagtcatctacaacaggaagcgatgaaaggcaaagcgcagcagacgcacaaggttcatcgcagggagttcgccgaaggggacacagtgtacttgaggatgtatgaagatccaagaggtagatgggcaccaggagtggtcatcagaagacttggaacagtgctctatgatgtccgatggaacaacaagattgtcaagcgacatgccaatcagctcaggcctcggatagtggatgatgccctaaacgtcatgatggagaccttcgacctgcctctattgcccctcagccaaccggaaggtaacaacgtcattgttacagagcctagcacgacaggagaagaagttcttgccgaagcagaccctgccccacgaccaaagaggagacgccgaccaccgaggagattggagatggacgcgtccaggaagaagtaccaactttgttaggaggggaggtgttatggtgatgaaccaatgagagttgagcgcgctaagaacacaatggaatatgttctataaatagccttgggctatcgctcagcctctctctctcgtgtagtgaccgtgtattatcgttgtttacttgttaataaaccgtttcacgtttaacttcgtcttcttcggtatcccagagatataacagcattcgaattgttgtccttaatactctcgagtttcgataacaatggtgtgagagtggttgttaggtagtctgtgaacgatttagtaacttgcatcaacatattctgaaacattgcagtcatattttccgtcatcttggtcatacattcgctgagtttctcattgaaactagtcattaatttgtcattcatctgcagcattttttcgaacatactttctgaaaaatcgcccaagacttctctcgggaagtcttccgatgtagaagtttttgctgacctcggcatagcgaaaaagatgctcgataaattagaaacagaaaaacactgaacttatgatccagaaacgaaatttgaggtaactaaatcgctcgaaagttcgggagtgaaatcaaaacatccgccatgtttggcctcaaaacctaaaaacttcatgtatagtataacatataatatgaattaacacaatactttgatataaacagcattcttagtctaacccctggagcaagaataaataaattgtttgggggaacccacccttgagcatgcaacataatgttgtccatgggaaaaacatggacgtaatttcagcttgttccaagggcgggcaaatcctcgtaaaatatgaggggatgacctcttttttgcgtctagtctaaaatcagtctaaaggccaatttctcctttagccctgatttcagccagaattaaattggttaaaaatgtttttcctgtgccttcaaaagcatcaaggaaaatgatacagtcttgttgttttgctagaattgcaaccttaccggccggtttgtaggcccgcgaatttatatatgtaataaaatgtgggattgatgaatggatgggtggttggatggatggatgaatgtatggatggatatatataacatataacattatataccgtgatatattcacttctgcttatttgaacaatgattgttgtttat*ag*agaattttagaaac**tgc**actgatcgatatttgatggaacca**tgc**ctc**tgt**ggg*gt*aaattataaaattacaactttaatttatcaactgttattatatatgttcgtggtttatgagtttattgaataaaacaaacaagccgttatatgcaaagaaaaaaacaaaacgcgaaagcactaaataaattcaaagaaacccaaagggccagccttaaggaagcacacgcttcggctgtcggactgatcctgaaagtggttaaatatacaaaatcataacctatacccctcccctcaataaacattgtttgcacgactttttgaatagtaaataaagtaatttgaataggcatgtgtttaactgagttagatccttgatagataaaggtatggaggagtaaagtctgtttaatataaataagtataatacaatatgatattaataatatcatattattaatattatcattgaacattc*ag*aatggctccaaggta**tgt**agccgatatgaagat**tgt**gacggaaacaaa**tgc**aaag*gt*aatatttatgtaataaaatttactattatatgtgcttatgttattaattttatttttgtcattaatttcacttttgtttaaaatttaattttgcatgaataatatttattattaatattgttatgcccaaggaaagaattataaataataaaatgcctcatcaatgatttgaaatggattaaaggatgtatgaagttatggctataacattacatatcgtgatattttcacatctgttcattataaacatgaatgttgattgtagagatttataaactttttatttgtggatggaaggatgggtttatgaatggatggatggatggatgtatggatggatggatgggttgatgggtgcatgagtgatttggtttgtggatgaatgcatttgtggattgatggatggatggttggttatagcattacataccgtgaaaaattcacttatgattattcttgaaatgttgttggttgt*ag*agattcac**tgc**tctgatcgaaatgcgaatgaaccattt**tgc**ctt**tgt**ggg*gt*aaatgataagattacaagttttagttatcaattgttatcataaataaagaataattttttattgaacattc*ag*aatgagactgta**tgt**aatcgatacgaagtt**tgt**gagcagaattctgaaggaaacaaa**tgc**aaaa*gt*aattttaatttcacaaatgttattaattacatttttgtgatttgaattccaaggaaagaaatgcatgggtaattttgataagttattaattttacatttttaataatttaaattttgtcattaattttttaatgttaataatttttgtttttattatcaatttcatttttttttattaaattttaaaaaatgttaatgcaatttattgttaactttattatgctcaacgaaagagtgatatgtaataaaatgtgggattgatgaatggatgggtggctggatggatggatgaatgtatggatggatgtatataacatataacattatataccgtgatatattcatttctgtttatttgaacaattattgttgtttat*ag*agaattttagaaac**tgc**actaatcgatattacatggaacca**tgc**ctc**tgt**ggg*gt*aaattataaaattacaactttaatttatcaactgttattatatatgttcgtggtttatgagtttattgaataaaacaaacaagccgttatatgcaacgaaaaaaacaaaacgcgaaagcactaaataaattcaaagaaacccaaagggccagcctgaaagtggttaaatatacaaaatcataagctatacacttcccctcaataaacaatgtttgcacgacttcttgaatagtaagtaaagtaatttgaatgggcatgtgtttaactgagttagatccttgatagataaaggtatggaggagtaaagtctgtttaatataaataagtataatatgatatgatattattaatatcattattcaacattc*ag*aagggcgctaaggta**tgt**agtcagtatgaagat**tgt**gaagaaaacgaa**tgc**atag*gt*aatttttatttcataaaattttctattaattttccttatgttatttattttatttttgttattaattacattttttttaaattttatttttacataaatatcattgtaatttattgttttttttaaaatttactttcatttaaataatattgttatatttgttaatgctcaacgaaagactgatagttagcaaatagtatcaaggatttggaatggatggatggatatttataacattacatactgtgatatattcacttctcttcattttgaaattgaatatttgttgt*ag*agattgatgtacac**tgc**tcttatcgaaatgctagggaatca**tgc**ctc**tgt**ggg*gt*aaattaagattgcaactttaatttatcaactgttattatatttttttgtggcttatgagtttattgaataaaacaaacaagccgctatatacgcggaaaaaacaacatgcgaacacactaaatagattcaaataaacccaaaggactgtccgtgaaagtggctatatacaaaatctatgtaaaaaataagctatagacttccactcaataaatattgtttgcctgacattttgaattgtaaataaattaatttaaacgatcatgttcagatatatcagttagatcattgatagatgaaggtatggaggagtaaagtctgtttatcataaataacgtataatatgatattactgatattatcattgaacattc*ag*aatgactctaaggca**tgt**agtcgatatgaaaat**tgt**caaggaaacaaa**tgc**aaag*gt*aatatttatgtaataaaatttactattaattttgcttatgttattaaatttatttttgctaataatttatttttgctatcaatttctttttatatttttattaatttcattttttattaatcttatttttataatattttaatttattattaatactgttaagctcaacgaaagattgatacttaacaaaaagtgcctcatcaaagatttggaatggatggatggatgcagtgttatttccatttgccaaataatttccatttcaattttaattccattattatttgacaaataatttctatttgtgttctaattccattcaaagttctaaatttccattcaattctaattaccattgctgaaataatttctaatttctatttctgtttttattctaattccatttttcaatattttgttttaatgtatggcctttcgaaagtggaattgattataaaaccatccttaaagtaatgctagaactacaactaatatgcagtagctaacattaaaatcatggaacatttattgccacagttaacaaaagcatgcagtcacatagcaaaaaaaatctatcaaaatgagtttagtttgagaaaagctctcatatccaaagactgtagcattcgattgcgccgttctgtagtcaggatgccgcagagcaaaaatatccgctccatgtaggcctgtgacaataagtcctgggctatgtcatacaatttactgtagcgagtagtcttctctttccagaaatttattgcatcattatgttcttcttctattatatcagctagatatcttttgagctgattatgaacagtgtcccttttgctgatgtaacactcacagcaattgtactggactgaatcttatttgtgaaaaatttaaacttcttaagttaagtcggagtcagcaatgatagtgacgatgcagatacaccaccagaaccagacacatgaactgcctgaactgaatcttccttgctttcataaaaaaaagtacagctaagttgcaaacagcagcagtgcacattccggtgagataattgctcgtgacaatgtcttggaaaaatataccgatagatgactgcaataaattagcatagaaaaaagattggaaaattcatccataatattatttgttaactaagcttgggttacaccgaattggcttcagaaataatgtctattgctcattataattccattttacaaaataaattctacttctcagtctatttccatttttttgggcaatttcaattcctattcaaaatttcattcccaaatttcaattttcattttcgagaaaaaataatttttatttcaattaccattaatggatatatcactgggtggatggatataacattacataccgtgatattttaactaatgtttattttaaaaatgaatactggttgt*ag*cgcgttatagaagc**tgc**tctgatcataataagtggcca**tgc**ctc**tgt**aag*gt*aagttataagattacaacttttaattatcagatgttatcattaataacgtataatatcatcattgaacattc*ag*ggtaatcataaa**tgt**gattattatgaaact**tgt**gaactaggagagattaacaaa**tgc**aaaa*gt*aattttcatataataacatttgttaataaatttttgtagtcattttattttttataaataataaaatattaattcattattaatacaatgctcttaatatttgtattaaaatgaaactttgatgattga*ag*aaaaatac**tgt**tcggaaaaaaatgaaaagcca**tgc**tac**tgt**ggg*gt*aaattataagattataacttatatttaccaaatgttatcttaaataacgtataatatcatcattgaacaatc*ag*aatgaaactcaa**tgc**aaatttgatcaaatt**tgt**gatgcaaagacaaacgaa**tgc**aaaa*gt*aatttccatataataaaatttgctattaatgtttttttattaattttagttttactaaagtaatattgcaacttaatatttatactatgctcttaaaatttataataaaaacgaatgttgattgt*ag*aaaattac**tgt**tcggaaaattcgaatgaatggccg**tgc**tac**tgt**gtc*gt*aagttataagataataacttttagttatcagatgttatcataaataacgtataatatcatcattgaacattc*ag*aatgaaactcgc**tgt**aaactacatgaaaaa**tgt**gaactgaattctgaaggaaacaaa**tgc**ataattg*gt*aatttttatactgaaatttaataataattgtacttatattattaattttatttttattattaattttatttttattattaattttatttttattattaatttcaaattttagttatgttgttaattttatttttacaaaaataatattataatttactgttatttaatttacattacgtccttaatatttatattaaaaatgaatgttggttgt*ag*aaaaa**tgc**acggttagaaatggatattct**tgc**tac**tgt**gag*gt*taattaaaaataataactttcagttatcaaatgttatcataaataacgtataatatcatcattgaacattc*ag*aatgactctgaagta**tgt**ggtgagtatcaagag**tgc**gaagtgaattctgaaggaaacaaa**tgc**atag*gt*aaattttttaagttattaattttatatttttttaaattgttattttgttattaatttttatatggtactaattttatttttattatgaataacatttttatttattattaatactattaacctcaacgaaaaaaagcctcaccaatgatttgggaggtagggagtcatggatggatggatggatggatggatggatggatggatggatgaatgaatgatggatggatgtaacattacacaccgtgatgataaattcacttctgtttatttcaaaaatgttattggttgt*ag*agttgtatgaactc**tgt**tctgatggaagtgactggcca**tgc**cgc**tgt**ggg*gt*aaattataagattaaatattattattaaagattataataacttttagtcatcaaatgtttacataaataacgtataatatcatcattgaacattc*ag*aatgaaactctt**tgt**tataaacatcaatat**tgt**gacctgaattctgaaggagaaaaa**tgc**aaaa*gt*agttattgggcaataaattttataaatgataataatttgatttttgttattaattttattttattaattttacacaaataatactgtaatttaatattaatactaatatgatcagtgatatgcaataaaacgtgcttcaaaactgatatgggatggatggatggatataacattacataccgtgatatattcaattctgtttattttaaatataaatattggttgt*ag*agattaattttagtctc**tgc**tctgaacgtaatgagtatcca**tgc**cgc**tgt**gag*gt*aagttataagattgcaacttttatttatcaaatgttatcataaacaacgtataatgttgtcattgaacattc*ag*ggtgataatata**tgc**agtgaatatgaatta**tgt**gacctggaaattaacaaa**tgc**aaaa*gt*aattttacataataaaatttatcattaatttttatttatttcaattttaaaaaaattacattgtaatttattattaataccatgctcttaatatttataataaaaatgaatgtttattgt*ag*aaaaatac**tgt**tcggaaagaaatgaatatcct**tgc**ttc**tgt**gtg*gt*aaacattttacattcattagataataacttttattatcataaataacgtcatcataaataatatataataacttttaagattataataagtattagtcatcaaatgttatcataaataacgtataatgccatcattaaacattc*ag*ggtgaaaaaacc**tgt**gacgtttttgacact**tgt**gaattgaatcgcacaagaaacaaa**tgc**agac*gt*aatcttatataataaatatgtatatgtatattaaatttcactattgatttcaaatacgttcttatttctattttttaaatttatttttagcattaattcgtgttattactaattgtttgattgt*ag*ttagatat**tgt**tcggaaaaaaatgaacatcct**tgt**cgt**tgt**ttg*gt*aaatttgaaaataataactttatttatcaaatgttatcataaataacgtataatatcatcatc*ag*atggaggagaatcgtgtc**tgt**tattatcatgaaaat**tgt**gaattgaattctgaaggaaacaaa**tgc**gtaacaa*gt*aatttttataagttatttggtatttatgaaatttaataataattttatttgtgttattaattttttttacaaaaataatgtcataatttattattatttaatttatattatgttcttatattttatattaaatatgaatgttggttgt*ag*aaaaa**tgc**acggaaagaaattattatccc**tgc**ctc**tgt**gtg*gt*aaattataagataataactttcagttatcaaatgttatcataaataacgtataatatcatcattgaacattc*ag*aatgacactgaacaa**tgt**ggtaaatatgaagag**tgt**gaaattcatgatgaaggaaacaaa**tgc**atag*gt*aatttttataagttattaattttatatatttattaatttcatttttacataaatattattgtgatttataattaatactatcatcctcaacgaaaaagtgatatgtaatagaaggtgcctcaccaatgatttgggatggatggatggaaggatggatggatggatggatggattattgccaaaaaacctctcctgggcttattctcccgagagcgttcgcgcagttttcaaagctggtgagcttaggcgcatgctgattaatattcatgtgcttaaacatttagtttatattgtaaattttcctttttatttttttaaatttacattttctattatttatctatgatttaatattagcgtttcataggtagacatctcatattgtaacgttctttctcctaaatactcgagatgtgcccaaacgagccgagtcgaatcgaaataacgccacaagattcagatattatgaaggcaatatattaaaaatcacaagtgtcaatcaaaatgtacaagaaattgatttcaaataacaattaaatacaatattcgataaaaactgtcaaccataaaactcttcaaaactcgtctgccactctcgcctctctatctatctatctctctctctctctctctctctttctctctctctctctcactaacatgctctccgacctttcccaatgctgaaaagctaatctgtcggttggttggttggtattttattaactaatctgtatttatattatttatttatttgattatagcccgtcgcataactagatgattaggacggactaatccgtcgtctgcatggcgccttttatagccatgcgacccagatatgttaattgtctcactgcgcagccaatcaacgcatcgcgcggttccaggcagacgacagacacgccgcatcacattcgttccacctacgcgatataaaataaaaagccaaaataaaatacaataaataacataaataaatacagttaggacactgacacattcgtaatttctggcgttgtattagtcgttgtaaataaaagaattgaagtttattaatttgaatatctattacc*ag*gcaaaggaggaaacgaggaggcggtggaaacagccagaataa*gt*aattttttatattaaaatttgcaattaatttcagttcttttaataattttgtttttagtattgattttatttttgttatttttttttgttttaaaatacaatgttattattaatttcatagttttcatcaatttatttttacaaaaataataatgtaattcattattagtattatgctcttaatctggtattaattaataggggcaaacagtgtctaaagttgaaatttcttttttgattc*ag*aggga**taa**

**atg**tcgaagctgtttgttgtcctcttggcttcttttggcatcggtctgtctgattgcgagtttgatctgtctcaag

agatttataaacac**tgt**tctgatcgaaatgggactgaacca**tgc**ctc**tgt**ggg

aatagctctaaggta**tgt**ggtcgatatgaagat**tgt**gaagggaacata**tgc**aaag

agattaatattcac**tgc**tctgatcgaaatgggaatgaacca**tgc**ctc**tgt**ggg

aatgactataaggta**tgt**agtcgatatgaagat**tgt**caaagaaacaaa**tgc**aaag

agattaatattcac**tgc**tctgatcgaattgggaatgaacca**tgc**ctc**tgt**ggg

aatggctctaaggta**tgt**agtcgatatgaagat**tgt**gaaagaaacaaa**tgc**aaag

agattaatattcac**tgc**tcagatcgaaattggaatgaacca**tgc**ctc**tgt**ggg

aatgagactata**tgt**tatcgatatgaagtt**tgt**gagcagaattctgaaggaaacaat**tgc**aaag

agaattttagaaac**tgc**actgatcgatatttgatggaacca**tgc**ctc**tgt**ggg

aatggctccaaggta**tgt**agccgatatgaagat**tgt**gacggaaacaaa**tgc**aaag

agattcac**tgc**tctgatcgaaatgcgaatgaaccattt**tgc**ctt**tgt**ggg

aatgagactgta**tgt**aatcgatacgaagtt**tgt**gagcagaattctgaaggaaacaaa**tgc**aaaa

agaattttagaaac**tgc**actaatcgatattacatggaacca**tgc**ctc**tgt**ggg

aagggcgctaaggta**tgt**agtcagtatgaagat**tgt**gaagaaaacgaa**tgc**atag

agattgatgtacac**tgc**tcttatcgaaatgctagggaatca**tgc**ctc**tgt**ggg

aatgactctaaggca**tgt**agtcgatatgaaaat**tgt**caaggaaacaaa**tgc**aaag

cgcgttatagaagc**tgc**tctgatcataataagtggcca**tgc**ctc**tgt**aag

ggtaatcataaa**tgt**gattattatgaaact**tgt**gaactaggagagattaacaaa**tgc**aaaa

aaaaatac**tgt**tcggaaaaaaatgaaaagcca**tgc**tac**tgt**ggg

aatgaaactcaa**tgc**aaatttgatcaaatt**tgt**gatgcaaagacaaacgaa**tgc**aaaa

aaaattac**tgt**tcggaaaattcgaatgaatggccg**tgc**tac**tgt**gtc

aatgaaactcgc**tgt**aaactacatgaaaaa**tgt**gaactgaattctgaaggaaacaaa**tgc**ataattg

aaaaa**tgc**acggttagaaatggatattct**tgc**tac**tgt**gag

aatgactctgaagta**tgt**ggtgagtatcaagag**tgc**gaagtgaattctgaaggaaacaaa**tgc**atag

agttgtatgaactc**tgt**tctgatggaagtgactggcca**tgc**cgc**tgt**ggg

aatgaaactctt**tgt**tataaacatcaatat**tgt**gacctgaattctgaaggagaaaaa**tgc**aaaa

agattaattttagtctc**tgc**tctgaacgtaatgagtatcca**tgc**cgc**tgt**gag

ggtgataatata**tgc**agtgaatatgaatta**tgt**gacctggaaattaacaaa**tgc**aaaa

aaaaatac**tgt**tcggaaagaaatgaatatcct**tgc**ttc**tgt**gtg

ggtgaaaaaacc**tgt**gacgtttttgacact**tgt**gaattgaatcgcacaagaaacaaa**tgc**agac

ttagatat**tgt**tcggaaaaaaatgaacatcct**tgt**cgt**tgt**ttg

atggaggagaatcgtgtc**tgt**tattatcatgaaaat**tgt**gaattgaattctgaaggaaacaaa**tgc**gtaacaa

aaaaa**tgc**acggaaagaaattattatccc**tgc**ctc**tgt**gtg

aatgacactgaacaa**tgt**ggtaaatatgaagag**tgt**gaaattcatgatgaaggaaacaaa**tgc**atag

gcaaaggaggaaacgaggaggcggtggaaacagccagaataa

aggga**taa**

MSKLFVVLLASFGIGLSDC

EFDLSQEIYKH**C**SDRNGTEP**C**L**C**GNSSKV**C**GRYED**C**EGNI**C**K

EINIH**C**SDRNGNEP**C**L**C**GNDYKV**C**SRYED**C**QRNK**C**K

EINIH**C**SDRIGNEP**C**L**C**GNGSKV**C**SRYED**C**ERNK**C**K

EINIH**C**SDRNWNEP**C**L**C**GNETI**C**YRYEV**C**EQNSEGNN**C**K

ENFRN**C**TDRYLMEP**C**L**C**GNGSKV**C**SRYED**C**DGNK**C**K

EIH**C**SDRNANEPF**C**L**C**GNETV**C**NRYEV**C**EQNSEGNK**C**K

KNFRN**C**TNRYYMEP**C**L**C**GKGAKV**C**SQYED**C**EENE**C**I

EIDVH**C**SYRNARES**C**L**C**GNDSKA**C**SRYEN**C**QGNK**C**K

ARYRS**C**SDHNKWP**C**L**C**KGNHK**C**DYYET**C**ELGEINK**C**K

KKY**C**SEKNEKP**C**Y**C**GNETQ**C**KFDQI**C**DAKTNE**C**K

KNY**C**SENSNEWP**C**Y**C**VNETR**C**KLHEK**C**ELNSEGNK**C**I

IEK**C**TVRNGYS**C**Y**C**ENDSEV**C**GEYQE**C**EVNSEGNK**C**I

ELYEL**C**SDGSDWP**C**R**C**GNETL**C**YKHQY**C**DLNSEGEK**C**K

KINFSL**C**SERNEYP**C**R**C**EGDNI**C**SEYEL**C**DLEINK**C**K

KKY**C**SERNEYP**C**F**C**VGEKT**C**DVFDT**C**ELNRTRNK**C**R

LRY**C**SEKNEHP**C**R**C**LMEENRV**C**YYHEN**C**ELNSEGNK**C**V

TKK**C**TERNYYP**C**L**C**VNDTEQ**C**GKYEE**C**EIHDEGNK **C**IGKGGNEEAVETARIKG-

Theoretical pI/Mw: **5.30** / 74754.37

**Htia_mHV11 (this study)**

**chromosom 2 position 1547058 - 1559837**

**atg**ttgaagctgtttgttgtcctcttggcttcttttggcatcggtctgtctgattgcgagtttgatctgtctcaag*gt*tagtgtgatttgagtttctgaacgaaagagtgatatgtaatatttgtactacatattatttcaccaatgattttggaagaatggatggatggatggatggatgaaaggatggatggatataacattacataccgtgacaaattcacttcattttattttaaaaatgaatgttggttgt*ag*agatttataaacac**tgt**tctgatcgaaatgggactgaacca**tgc**ctc**tgt**ggg*gt*aagttataagatttatttacaaaatgttatcagaaatataataagatattaaaaatatcataactgaacattc*ag*aatagctctaaggta**tgt**ggtcgatatgaagat**tgt**gaagggaacata**tgc**aaag*gt*aatttttaattaatcaaatatactattaattttgcttatgttattaattttatttttgtcaacaatttcacttttgtttaaaaattatttttacataaataatattcattatcaatactgttatgcccaaggaaagaatgataattaataaaaattgtctcaccaatgatttggagtggatgaaaggatgtatgaagggatggatataacattacatgtcgtgatattttcacttctgtttattatagacatgattgttgagatttataaacattgtatttgtagattgatggatggatggatggatggatagatctaaaattacataccgtgataaattcacttctgtttattctaaaaatgttgttggttgt*ag*agattaatattcac**tgc**tctgatcgaaatgggaatgaacca**tgc**ctc**tgt**ggg*gt*aaattataagattacaactattatttacaaaatgttaacataaataacatataattttatattattaatatcttcattgatcattc*ag*aatgactctaaggta**tgt**agtcgatatgaagat**tgt**caaagaaacaaa**tgc**aaag*gt*aatatttatgtaataaaatttactaataattttatttttgtcatcaatttcacttttgtttaaaatttatttttgcatgaataatatttgttattaatactgtcatggccaaggaaagaatgataatgaataaaaggtgtgtcaccaatgatttggaatggatgaaatgatatgtgaagttatggctataacattacatatcatgatattttcacttatatatattgtaaacatgaatgttggttgtagagatttataaactttttatttgtggatggatgattggtgggatgggtggatggatggattgatggattatggatggatagattgatgattggatggatggttcggtgggcggatgggtgatttggtttttggatgaatgtatttgtggattgatggatggaaggatggatataacattacataccgtgataaattcacttctgtttattctaaaaatgttgttggttgtagagattaatattcactgctctgatcgaattgggaatgaaccatgcctctgtggggtaaattataagattacaactattatttacaaaatattatcataaataacatataagtttatattattaatatcttcattgaacattcagaatggctctaaggtatgtagtcgatatgaagattgtgaaagaaacaaatgcaaaggtaatattaatatttatgtaataatatgtataaacatgaatgttggttgtacagatttataaactttctatgagcggatgcatgggtgatttggtttgtggatgaatgcatttgtggattgatggatagatataacattacataccgtgataaaatcacttctgtttattctaaaaatgttgttggttgt*ag*agattaatattcac**tgc**tcagatcgaaattggaatgaacca**tgc**ctc**tgt**ggg*gt*aaattataagattacaacttttagttatcaattgttatcataaataacgtataatattttcattgaacattc*ag*aatgagactata**tgt**tatcgatatgaagtt**tgt**gagcagaattctgaaggaaacaaa**tgc**aaag*gt*aattttaatacaataaagtttagttttaatttcacaaaagtttttagttacatttttgcagagatgggtagttgctaactataagtagctaactattagttaataattactttttaactactttaaagtagttaataactacttattaaaaatataattaaaaactagtagttaactaattttaaagtagttagtaactacaaatagttgactactatttaactacttttaataacattttaaaataaaattttaatatttcaatttggtacatcactatataagctttgtttctttgatttcatagtttgtttatatactctgacttctgaattatcactcttagtagccttatttggaaaacgttatatttaaatgatgacatggacgagccactatgtgatatttaagaaaataatgaaggtccaactatcattggcatcaacaaaaaattatcatcttgttcagataaaactgtcaagccctatcattacatggaatgtataccaaactcttgtgataatgtgttagctgaacagcttaatgattatttatcaaactcagcaaggaatgtacaaagtctactcaattatccaactgtttgtgctgcattcatcaagcttaattcgacattacctatgttcggcagcagttgaaagactttttagtatagcagggcaggttttatgtaacagacgatgtaaattatcggataatcatatcgtcatgatgatttttttaagagatctcccaacacatccactgtatacttctgttttagaccaatttggacaataaaatttcgttgataatgttacaatagtgtaaataaatatgctgataattaagtcttgttcagttgtcctgaaaattaatgttatcaaatgtcaactgaaaagttgtttaaaagtagttactaactacttatttaaaaattagtttaaaatagttactaactacattttcctataagtagttaactaccttttaattagttaaagcccatcgctgcatttttaatatttgaattccaaggaaagaaatgcatgggtaatttttataagttactagatgtttaccctgctctgcccggtaatagatttgcaattgaataaacttcatgtatagtataacatataatatgaattaacacaatacttttttataaacagcattcttagtctaacccctggagcaagaataaataaattgttgggggaacccacccttgagcatgcaacataatgttgtccatgggaaaaacatggacgtaatttcagcttgttccaagggcgggcaaatcctcgtaaaatatgaggggatgacctcttttttgcgtctagtctaaaatcagtctaaaggccaatttctcctttagccctgatttcagccagaattaaattggttaaaaatgtttttcctgtgccttcaaaagcatcaaggaaaatgatacagtcttgttgttttgctagaattgcaaccttaccggccggtttgtaggcccgcgaatttatatatgtaataaaatgtgggattgatgaatggatgggtggttggatggatggatggatgaatgtatggatggatatatataacattatataccgtgatatattcacttctgcttatttgaacaatgattgttgtttat*ag*agaattttagaaac**tgc**actgatcgatatttgatggaacca**tgc**ctc**tgt**ggg*gt*aaattataaaattacaactttaatttatcaactgttattatatatgttcgtggtttatgagtttattgaataaaacaaacaagccgttatatgcaacgaaaaaaacaaaacgcgaaagcactaaataaattcaaagaaacccaaagggccagcctgaaagtggctatatacaaaatcataagctatacacttcccctcaataaacaatgtttgcacgactttttgaatagtaaataaagtaatttgaataggcatgtgtttaactgagttagatccttgatagataaaggtatggaggagtaaagtctgtttaatataaataagtataatacaatatgatattaataatatcatattattaatattatcattgaacattc*ag*aatggctccaaggta**tgt**agccgatatgaagat**tgt**gacggaaacaaa**tgc**aaag*gt*aatatttatgtaataaaatttactattatatgtgcttatgttattaattttatttttgtcattaatttcacttttgtttaaaatttaattttgcatgaataatatttattattgttaataataattaatattgttatgcccaaggaaagaattataaataataaaatgtgcctcatcaatgatttgaaatggattaaaggatgtatgaagttatggctataacattacatatcgtgatattttcacatctgttcattataaacatgaatgttgattgtagagatttataaactttttatttgtggatggaaggatgggtttatgaatggatggacggatggatgtatggatggatggatgggttgatgggtgcatgagtgatttggtttgtggatgaatgcatttgtggattgatggatggatggttggttatagcattacataccgtgaaaaattcacttatgattattcttgaaatgttgttggttgt*ag*agattcac**tgc**tctgatcgaaatgcgaatgaaccattt**tgc**ctt**tgt**ggg*gt*aaatgataagattacaagttttagttatcaattgtaatcataaataaagaataattttttattgaacattc*ag*aatgacactgaa**tgt**aatcgatacgaagtt**tgt**gagcagaattctgaaggaaacaaa**tgc**aaaa*gt*aattttaatttcacaaatgttattaattacatttttgtgatttgaattccaaggaaagaaatgcatgggtaattttgataagttattaattttacatttttattaatttaaattttgtcattaattttttaatgttaataatttttgtttttattatcaatttcatttttttttattaaattttaaaaaatgttaatgcaatttattgttaactttattatgctcaacgaaagagtgatatgtaataaaatgtgggattgatgaatggatgggtggttggatggatggatgaatgtatggatggatgtatataacatataacattatataccgtgatatattcacttctgtttatttgaacaattattgttgtttat*ag*agaattttagaaac**tgc**attgttcgatattacatggaacca**tgc**ctc**tgt**ggg*gt*aaattataaaattacaactttaatttatcaactgttattatatatgttcgtggtttatgagtttattgaataaaacaaacaagccgttatatgcaacgaaaaaaacaaatcgcgaaagcactaaataaattcaaagaaacccaaagggccagcctgaaagtggctatatacaaaatcataagctatacacttcccctcaataaacaatgtttgaacgacttcttgaatagcaagtaaagtaatttgaatgggcatgtgtttaactgagttagatccttgatagataaaggtatggaggagtaaaatctgtttaatataaataagtataatatgatatgatattattactatcatattattaatattaccattgaacattc*ag*aaaggcgctaaggta**tgt**agtcagtatgaagat**tgt**gaagaaaacgaa**tgc**atag*gt*aatttttaattaataaaatttattattaattttccttctgtcatttattttatttttgttattaatttcattttttttaaattttatttttacataaatatcattgtaatttattgtttttttttaaatttactttcatttaaataatattgttatatttgttaatgctcaacgaaagactgatacttagcaaaaagtatcaaggatttggaatggatggatggatatttataacattacatactgtgatatattcacttctcttcattttcaaattgaatatttgttgt*ag*agattgatgtacac**tgc**tcttatcgaaatcctaggaaatca**tgc**ctc**tgt**ggg*gt*aaattataaaattataactttaatctatcaactgttattatatttttttgtggcttatgagtttattgaataaaacaaacaagccgttatatacacggaaaaaacaacacgcgaaagcactaaatagattcatataaacccaaaggactaaccctgaaagtggctatatacaaaatcataagctatattaacttccactcaataaatattgttcgcctgacattttgaattgtaaataaattaatttgaacggtcatgttgagttagatcagttagatcattgatagaggaaggtatggaggagtaaagtctgtttatcataaataacgtataatatgatattattgatatcatcattgaacattc*ag*aatggctctaaggca**tgt**agtcgatatgaagat**tgt**caaggaaacaaa**tgc**aaag*gt*aatatttatgtaataaaatttactattaattttgcttatgttattaattttatttttgctaataatttatttttgctatcaatttctttttatatttttattaatttcattttttattaatcttatttttataatattttaatttattattaatactgttaagctcaacgaaagattgatacttaacaaaaatgcctcatcaaagatttggaatggatggatggatgcagtgttatttccatttgccaaataatttccatttcaattttaattccattattatttgacaaataatttctatttgttttctaattccattcaaagttctaaatttccattcaattctaagtgaattaattctgtgaagtcctggacaataagtcctgggctatgtcatacaatttactgtagcgagtagtcttctctttccagaaatttattgcatcattatgttcttcttctattatatcagctagatatctcttgagattggaaaattcatccataatattatttgttaactaagcttgggttacaccgaattggcttcagaaataatttctatttctcattataattccattttacaaaataaattctacttctcagtctatttccatttttttgggcaatttcaattcctattcaaaatttcattcccaaatttcaattttcattttcgagaaaaaataatttttatttcaattaccattaatggatatatcactggatggatggatgtaacattacacaccgtgatattttaacttatgtttattataaaaatgaatggttgt*ag*cgcgttatagaagc**tgc**tctgatcataataagcggcca**tgc**ctc**tgt**aag*gt*aaattataagattacaacttttaattatcagatgttatcattaataacgtataatatcatcattgaacattc*ag*ggtaatcatgaa**tgt**gatccttatgaaact**tgt**gaactaggagagattaacaaa**tgc**aaaa*gt*aattttcatataataacatttgttaataatttttttttattcatgttattttttataaaaaataatattgtaatttattattaatacaatgctcttaatatttatattaaaaatgtaacttgattgt*ag*aaaaatac**tgt**tcggaaataaatacgtggccagaaataaataaggagcca**tgc**ttc**tgt**ggg*gt*aaattataagataataacttatatttaccaaatgttatcataaataacgtataatatcatcattgaacaatc*ag*aatgaaactaaa**tgc**aaatttgatcaaatt**tgt**gatgcaaagacaaacaaa**tgc**aaaa*gt*aattttcatataaaaaatttgttattaatgttttttattaattataattttacaaaaataatattgtaatttattattgaaaatatgctcttcatatttataataaaaataaaagttgattgt*ag*aaaattac**tgt**tcagaagattcagatgaatggcca**tgc**tac**tgt**acg*gt*aaattataagataataacttttatttatcaaatgttatcataaataacgtataatatcatatcattgaacattc*ag*gaggatcaaatc**tgt**tattcatatgaaaaa**tgt**gaactgaattctgaaggaaacaaa**tgc**atatttg*gt*aatgaaatttaataataatattacttttaatattaattttatttttattattaatttcaaattttattatttacaaaaataatattataatttactgttatataatttatattacattcttaatatttatattaaaaatgaatgttggttgt*ag*aaaaa**tgc**aaggatacaaatgcacatact**tgt**tac**tgt**gtg*gt*aaatttagaaataataactttcagttaccaaatgttatcataaataacgtataatatcatcattgaacattc*ag*aatgacactgaagta**tgt**ggtgaaaatcaagag**tgt**gaagtgaattttgaaggaaacaaa**tgc**aaag*gt*aattttatatttttattaattttaatttttattatgaataatattgcaatttattattaacactgttatcctctataaaaatagtaataaatgtaataaaaaagagtgcctctctattgatgaatggatggatggatggatggacggacggattgatggatggatggatgaatggatggatagatggatggatggatggatggatggatgaatgatggatggatggatgtaacattacacaccgtgatgataaattcacttctgtttatttcaaaaatgttattggttgt*ag*agttatatgaactc**tgt**tctgatggaagtgactggcca**tgt**cgc**tgt**ggg*gt*aaattataagattaaatattattattaaagattataataatttttagtcatcaaatgtttacataaataacgtataatatcatcattgaacattc*ag*aatgaaactcta**tgt**tataaacatcaatat**tgt**gacctgaattctgaaggagaaaaa**tgc**aaaa*gt*agttattgtgcaataaattttataaatgataataatttgatttttgttattaacttcattttattacttttacacaaataatactgtaatttattattaatactaatatgatcagtgatatgcaataaaacgtgcttcaaaactgatatggaattgatggatataacattacataccgtgatatattcaattctgtttattttaaatgtaaatattggttgt*ag*agattaattttagtctc**tgc**tctgaaagtaataagtatcca**tgc**cgc**tgt**gag*gt*aagttataagattgcaatttttatttatcaaatgttctcataaacaacgtataatatcgtcattgaacattc*ag*ggtgataatata**tgc**agtgaatatgaatta**tgt**gacctgggaattaacaaa**tgc**agaa*gt*aaattttcatacaataaaatttatcattaatttttattaatttcaatttttaaaaaataacattgcattttaatattaataccatgctcttaatatttataataaaaatgaatgtttacgatgaaatttgtaaactaggagagaataacacatgcaaaagtaattttcatatcataacatttgttattaatgattttttatttattttaatttatttattttaaattgtattgaaatttattattaaaactatgctcttaatttaaattacattcttaatatttatattaaaaatgaattttgtttgt*ag*aaaaatac**tgt**tcggaaataaatgaacagcca**tgc**tac**tgt**gag*gt*aaattataacataataacttttattcatcataaataacgtataatatcatcattgaacattc*ag*cgtgaacgtatc**tgt**ggcttgcttcaaaat**tgt**gaattgaatcctgaagaaggaaacaga**tgc**agac*gt*aatttttaatttttattaaatttcaattaaattgatttcaaatatgttattaattctatttattatacttttagcattaatatttttttgt*ag*ttagaaaa**tgc**tcggaagaaaataactatcct**tgc**ctt**tgt**gtg*gt*aaatttgaagataataacttttatatatcaaatgttatcataaataacgtataatatcatatcatattc*ag*gagaatcgtacc**tgt**cattattatgaaaaa**tgt**gaatttaattctgaaggaaacaaa**tgc**ataagac*gt*aatttttataagttatttggtatttatgaaatttaataataattttacttgtgttattaattttatttttattgttaatttcaagtttttgttattaatttttttttacacaatttattattatttaatttaaattacgttcttatttttatttattgaatatgaatgttggttgt*ag*aaaaa**tgc**acggatagaaatggaattcgatatccc**tgc**ctc**tgt**gtg*gt*aaattataagattgtaacttttatttatcaaatgttatcataaataacgtataatatcatcattgaacattc*ag*aatgactctgaaata**tgt**gctaaaaatgaattc**tgt**aaacttaattctgaaggaaacaaa**tgc**atag*gt*aatttttataagttattaattttgtatttttggatggatggatggatggatggatggatggatggatggatggatggatggatggatggatggatggatggatggatggatggatggatggatggatggatggatggatggatggatggatgtatggatataacattacatatcgtgacaaattcacttctgttcattttaaaaatgttgttggttgt*ag*agattcataaattc**tgc**tatgatcgaaagtggaatgaaaaa**tgt**ctc**tgt**ggg*gt*aaattataagttcacaacttctatttatcaattgttaacataaataacatataataccatcattgttcattt*ag*aataaaaccatg**tgt**tctccaaaggaattt**tgt**gacctgaattctgaaggaaacaaa**tgc**aaaa*gt*aatttttatgcaataaaatttactattaattttacaattgttggatggatggatggaaggatgaatggatggatggatggatataacattacacaccgtgataaattcacttctgtttattttaaacatgaatgttggttgt*ag*ggatttataaacac**tgc**tctgatctaaatgggaaagaacca**tgc**ctc**tgt**ggg*gt*caatgataagataacaacttttatttatcaaatgttatcataaataacatataatatctttgtcgaacattc*ag*ggtaaaagtata**tgc**aatgaaaatgaaatt**tgt**gacctgaaaattaaaaaa**tgc**aaaa*gt*aattttcacattataaaattttttattaattttaacattttttaaaaataatattgcaattcattattaatccttattctatatatatatataaaacaaggatcttgtatgtatatttcttaaacaaatctttaatccttaaccgatttgattgaaacttgaaacacttgagtactttgattaaggggtcagtttagtccacttgaaaaaaaaatcgttaaattatttttcgtattattgccaaaaaacctctcctgggcttattctcccgagaacgttcgcgcagttttcaaagctggtgagcttaggcgcttgctgattaatattcatgtgcgtaaacatttagtttatattgtaaattttccgttttatttttttaaatttacattttctattatttatctatgatttaatattagcgtttcataggtagccatctcatactgtaacgttctttctcctaaatactcgagatgtgcccaaacgagccgagtcgaatcgaaataacgccacaagattcagatattatgaaggcaatatattaaaaatcacaagtgtcaatcaaaatgtacaagaaatcgatttcaaataacaattaaatacaatattcgataaaaactgtcaaccataaaactcttcaaaactcgtctgccactctcgcctctctatctatctctctctctctctctccctctatctctctttttctctctctctcactaacatgctctccgacctttcccaaggctgaaaagctaatctgtcggttggttggtattttattaactaatctgtatttatattatttatttatttgattatagcccgccgcataactagatgattaggacggactaatccgtcgtctgcctggcgccttttatagccatgcgacctagatatgttaattgtctcactgcacagccaatcaacgcatcgcgcggttctaggcagacgacagacacgccgcatcacattcgttccacctacgcgatataaaataaaaagccaaaataaaacacaataaataatataaataaatacagttaggacactgacagattcgtaatttctggcgttgtagcagtcgttgcaaataaaagaattgaagtttattaatttgaatatttattacc*ag*gcaaaggaggaaacgaggaggcggtggaaacagccagaataa*gt*aattttttatattaaaatttgcaattaatttcagttcttttaatattataattttgtttttagtattgattttatttttgttattttttttgttttaaaatacaatgttattattaatttcatttttttttcataaatttatttttacaaaaatactaatgtaattcattattagtagtatgctcttaatctggtattaattaataaaggcaaacagtgtttaaagttgaaatttcttttttgactc*ag*aggga**taatag**

**atg**ttgaagctgtttgttgtcctcttggcttcttttggcatcggtctgtctgattgcgagtttgatctgtctcaag

agatttataaacac**tgt**tctgatcgaaatgggactgaacca**tgc**ctc**tgt**ggg

aatagctctaaggta**tgt**ggtcgatatgaagat**tgt**gaagggaacata**tgc**aaag

agattaatattcac**tgc**tctgatcgaaatgggaatgaacca**tgc**ctc**tgt**ggg

aatgactctaaggta**tgt**agtcgatatgaagat**tgt**caaagaaacaaa**tgc**aaag

agattaatattcac**tgc**tcagatcgaaattggaatgaacca**tgc**ctc**tgt**ggg

aatgagactata**tgt**tatcgatatgaagtt**tgt**gagcagaattctgaaggaaacaaa**tgc**aaag

agaattttagaaac**tgc**actgatcgatatttgatggaacca**tgc**ctc**tgt**ggg

aatggctccaaggta**tgt**agccgatatgaagat**tgt**gacggaaacaaa**tgc**aaag

agattcac**tgc**tctgatcgaaatgcgaatgaaccattt**tgc**ctt**tgt**ggg

aatgacactgaa**tgt**aatcgatacgaagtt**tgt**gagcagaattctgaaggaaacaaa**tgc**aaaa

agaattttagaaac**tgc**attgttcgatattacatggaacca**tgc**ctc**tgt**ggg

aaaggcgctaaggta**tgt**agtcagtatgaagat**tgt**gaagaaaacgaa**tgc**atag

agattgatgtacac**tgc**tcttatcgaaatcctaggaaatca**tgc**ctc**tgt**ggg

aatggctctaaggca**tgt**agtcgatatgaagat**tgt**caaggaaacaaa**tgc**aaag

cgcgttatagaagc**tgc**tctgatcataataagcggcca**tgc**ctc**tgt**aag

ggtaatcatgaa**tgt**gatccttatgaaact**tgt**gaactaggagagattaacaaa**tgc**aaaa

aaaaatac**tgt**tcggaaataaatacgtggccagaaataaataaggagcca**tgc**ttc**tgt**ggg

aatgaaactaaa**tgc**aaatttgatcaaatt**tgt**gatgcaaagacaaacaaa**tgc**aaaa

aaaattac**tgt**tcagaagattcagatgaatggcca**tgc**tac**tgt**acg

gaggatcaaatc**tgt**tattcatatgaaaaa**tgt**gaactgaattctgaaggaaacaaa**tgc**atatttg

aaaaa**tgc**aaggatacaaatgcacatact**tgt**tac**tgt**gtg

aatgacactgaagta**tgt**ggtgaaaatcaagag**tgt**gaagtgaattttgaaggaaacaaa**tgc**aaag

agttatatgaactc**tgt**tctgatggaagtgactggcca**tgt**cgc**tgt**ggg

aatgaaactcta**tgt**tataaacatcaatat**tgt**gacctgaattctgaaggagaaaaa**tgc**aaaa

agattaattttagtctc**tgc**tctgaaagtaataagtatcca**tgc**cgc**tgt**gag

ggtgataatata**tgc**agtgaatatgaatta**tgt**gacctgggaattaacaaa**tgc**agaa

aaaaatac**tgt**tcggaaataaatgaacagcca**tgc**tac**tgt**gag

cgtgaacgtatc**tgt**ggcttgcttcaaaat**tgt**gaattgaatcctgaagaaggaaacaga**tgc**agac

ttagaaaa**tgc**tcggaagaaaataactatcct**tgc**ctt**tgt**gtg

gagaatcgtacc**tgt**cattattatgaaaaa**tgt**gaatttaattctgaaggaaacaaa**tgc**ataagac

aaaaa**tgc**acggatagaaatggaattcgatatccc**tgc**ctc**tgt**gtg

aatgactctgaaata**tgt**gctaaaaatgaattc**tgt**aaacttaattctgaaggaaacaaa**tgc**atag

agattcataaattc**tgc**tatgatcgaaagtggaatgaaaaa**tgt**ctc**tgt**ggg

aataaaaccatg**tgt**tctccaaaggaattt**tgt**gacctgaattctgaaggaaacaaa**tgc**aaaa

ggatttataaacac**tgc**tctgatctaaatgggaaagaacca**tgc**ctc**tgt**ggg

ggtaaaagtata**tgc**aatgaaaatgaaatt**tgt**gacctgaaaattaaaaaa**tgc**aaaa

gcaaaggaggaaacgaggaggcggtggaaacagccagaataa

aggga**taa**

MLKLFVVLLASFGIGLSDC

EFDLSQEIYKH**C**SDRNGTEP**C**L**C**GNSSKV**C**GRYED**C**EGNI**C**K

EINIH**C**SDRNGNEP**C**L**C**GNDSKV**C**SRYED**C**QRNK**C**K

EINIH**C**SDRNWNEP**C**L**C**GNETI**C**YRYEV**C**EQNSEGNK**C**K

ENFRN**C**TDRYLMEP**C**L**C**GNGSKV**C**SRYED**C**DGNK**C**K

EIH**C**SDRNANEPF**C**L**C**GNDTE**C**NRYEV**C**EQNSEGNK**C**K

KNFRN**C**IVRYYMEP**C**L**C**GKGAKV**C**SQYED**C**EENE**C**I

EIDVH**C**SYRNPRKS**C**L**C**GNGSKA**C**SRYED**C**QGNK**C**K

ARYRS**C**SDHNKRP**C**L**C**KGNHE**C**DPYET**C**ELGEINK**C**K

KKY**C**SEINTWPEINKEP**C**F**C**GNETK**C**KFDQI**C**DAKTNK**C**K

KNY**C**SEDSDEWP**C**Y**C**TEDQI**C**YSYEK**C**ELNSEGNK**C**I

FEK**C**KDTNAHT**C**Y**C**VNDTEV**C**GENQE**C**EVNFEGNK**C**K

ELYEL**C**SDGSDWP**C**R**C**GNETL**C**YKHQY**C**DLNSEGEK**C**K

KINFSL**C**SESNKYP**C**R**C**EGDNI**C**SEYEL**C**DLGINK**C**R

KKY**C**SEINEQP**C**Y**C**ERERI**C**GLLQN**C**ELNPEEGNR**C**R

LRK**C**SEENNYP**C**L**C**VENRT**C**HYYEK**C**EFNSEGNK**C**I

RQK**C**TDRNGIRYP**C**L**C**VNDSEI**C**AKNEF**C**KLNSEGNK**C**I

EIHKF**C**YDRKWNEK**C**L**C**GNKTM**C**SPKEF**C**DLNSEGNK**C**K

RIYKH**C**SDLNGKEP**C**L**C**GGKSI**C**NENEI**C**DLKIKK **C**KSKGGNEEAVETARIKG-

Theoretical pI/Mw: **5.60** / 79896.76

**Htia_mHV12 (this study)**

**chromosom 2 position 1706734 - 1717866 rev + comp**

**atg**tcgaagctgtttgttgtcctcttggcttcttttggcatcggtctgtctgattgcgagtttgatctgtctcaag*gt*tagtgtgatttgagtttctgaacgaaagagtgatatgtaatatttgtactacaaattatttcaccaatgattttcgaagaatggatggatggatggatggatgaaaggatggatggatataacattacataccgtgacaaattcacttcattttattttaaaaatgaatgttggttgt*ag*agatttataaacac**tgt**tctgatcgaaatgggactgaacca**tgc**ctc**tgt**ggg*gt*aggttataagatttatttacaaaatgttatcagaaatataatatgatattaaaaatattaaaaatatcataactgaacattc*ag*aatggctctaaggta**tgt**ggtcgatatgaagat**tgt**gaagggaacata**tgc**aaag*gt*aatttttaattaatcaaatatactattaattttgcttatgttattaattttatttttgtcaacaatttcacttttgtttaaaaattatttttacaaaaataatattcattatcaatactgttatgcccaaggaaagaatgataattaataaaaattgtctcaccaatgatttggagtggatgaaaggatgtatgaagggatggatataacattacatgtcgtgatattttcacttctgtttattatagacatgattgttgagatttataaacattgtatttgtagattgatggatggatggatggatggatagatataaaattacataccgtgataaattcacttttgtttattctaaaaatgttgttggttgtagagattaatattcactgctg**atg**ttgaagctgtttgttgttctcttggcttcttttggcatcg*gt*ctgtctcaagatgagactttaaaaaaggcttcaccaatgatttgaaatgaatggatagatggatggatgggtggatggatggatggatgggtggatggatggatggatggatggatggatggatggatggatggatggatggatggatggatggatggatgggtggatggatggatggatggttaaactttacataatgtgatattttatttcctgtttattttaaaaatgaatgtttgt*ag*agatttatgaacac**tgc**tacatcccatacaagattcca**tgc**ctt**tgt**ggg*gt*aagttattacaactattatttatcaaatgatgaataatgtataataatcattgaacattc*ag*ggtaatcatgta**tgt**gatgatgatgaagtt**tgt**gatacatggacaaacgaa**tgc**aaaa*gt*aattttcatacaataaaatttgttaataatgttttttattgattttatttttagaaaaataatattgtaacttattattaatacagtgcacttaatatttatgttaaaagtaatgttggttgt*ag*aaatatac**tgt**tcggaaagaaatgtgtggcca**tgc**tac**tgt**gag*gt*aaattataagataataacttttatttatgagatattatcataaataacatttaatatcatcattgaacattc*ag*gaggatagaata**tgt**aagaaagatgaaatt**tgt**aaaataggaaagatc**tgt**tattcagaaaaaaga**tgt**gaactgaatcataaagccaacaaa**tgc**acatttg*gt*aagttttctgacaatgaaatttaatttttcttatgttactaatttttttattatttatttcaatatttaaaaataataatattgtaatttaatattttttctctgttcttattttttatattaaaaataattgttggttgt*ag*aaaaa**tgc**acggatagaaatcgatatcct**tgt**tac**tgt**gtg*gt*aaattataagataataactttcagttatcaaatgttatcataaataacatataatatcatcattgaacattc*ag*aatgactctgaagta**tgt**gggaaatatgaagat**tgt**gaacttaattctgaaggaaacaaa**tgc**atag*gt*aatttttataagttattaattttatatttttattaattttaattttatataaatatttgtgtaattttttattaatattattatcctcaatgaaaaagtaatatgtaatataaagtgccgcaccaatgatttgggatggatggatggataaatggatataaggatggaaggtcataacattacacatcgtgataaattcacttctgtttattctaaaaatgattttggttgt*ag*agattaaaattcac**tgc**tttgatcgaaaagggaatgaatca**tgc**atc**tgt**ggg*gt*aaaatataagattacaacttttatttacaaaatgttatcataaataataattaataccttcattgaacattc*ag*aatgaaaccata**tgt**tctcgaaatgaatat**tgt**gacttgaattctgaaggattcaaa**tgc**aaaa*gt*aatttttattcaataaaatatagtgtcaattttacaaacgttattaatttaatttttgtgacttgaattctgaaggaaagaaatgcataggtaatttgtataagttattaattttattttcattatcaatttaattttttttttaaatatacacaagtaatactgtaatttattattaatattattaaatatactattatgctgcgaaagagtgatatgtaataaaacgaacttcaaaacgtatgtgtgggaggatggatggatggatggatggatggatggatggatggatggatggatggatggatggatggatggatggatggatggatggatggatggatggatggatggatggatggatggatggatggatggatggatggatggatggatggatggatggatggatggatggatggatggatggatggatggatggatggatggatggatggatggatggatggatggatggatggatggatggatggatggatggatggatggatggatggatggatggatggatggatggatggatggatggatggatggatggatggatggatggatggatggatggatggatggatggatggatggatggatggatggatggatggatggatggatggatggatggatggatggatggatggatggatggatggatggatggatggatggatggatggatggatggatggatggatggatggatggatggatggatggatggatggatggatggatgtaatgatggatggatataacattacataccgtggtaaattcacatctgtttatttaaaaaatgaatgttgtttgt*ag*agaatttgagaccc**tgt**cctgatcgaaagtggtatgaatca**tgc**ctc**tgt**gcg*gt*aaatcataagattacaacttttaattatcaactgttatcataattttgttaacgtggtttatgagtttattgaataaaacagacaatcctcaaaaggccttaattccttaagtcggactgaccctgaaagtagctcaatacaaaatcataagctatatatttcactcaataaatattatttgcacattttgattagtaaataaagtaaattaaactagcatgttcagttagataaagtaatttgaaagggcatgtgtttaactcagttagatccttgatagatgaaggtatggaggagtaaagtctgtataacataatcaacgtataatatgattattgaacgttc*ag*aatggctctaaggta**tgc**agtcggtatgaaaat**tgt**gaagaaaacgaa**tgc**aaag*gt*aatttttatttaataaaatttactattgatttttgcgtatgttattaattttattttggttgtaaatttcattttttttttaaatttacttttacacaaataatattctaatttattatgaacactattacgttcaacaaaagagggatacttaacaaaaggacggaattgatggatggatggatggatggatggatggatggatggatggatggatggatggatggatggatggatggatggatggatggattgaaaatagatttttgaattttttggatgacagatttctgactcaacatgtcatggaaccaacaagatttagattcggacaaattgcacatatccttgatttggtgatatcaaatgatatagtagagaatgttgtttgcctcgctccattgggatgtagcgaccactctattgtttcgttcaatgttatatgtgatgttgaagatagcgccagagctagaagtaagccctgctatgatttaggagattacgccagtttaaatattgaacttgataacaagctggtggatgttttggaaccctctttgactcttgaatctaagtggacagtctttaaaaatactttaaacgagctgacaaaaaaacacataccccacagactgtgcaaaaagtttagattcaaaatgacaccagagcttcatgaattaataacgaggaaacactattgctggagacagatcttcaggtatggtgacacgaacagaatcaacgaattcaaagaccttagaaaaagagtaaaaaaattgtctgtgaagctaaatatagaaagccagaatcaattagcaatcaaattcaaatcaaatccaaaattattctggaatcatgtacaaaagaaaacaagaagtaaagatgcaatcagtacaatctcagtggacggtagtgatttgattggaggcaaggagaaggcacaggcttttgcggacttttttgcatcagtgtatattacggattcaggttctgaagtgacacctgttttcaatcccacagttccgggagtcgagacggaggaggttcggatcgacataaatgtactgtgtaggatgttgaaagatctaaacagcagtaaatcagcgggaccagatggaattcatccaagaattttaaaagagacacgggaatcgataaagtgttacttgaaacacatcttcgattgttccattcagtcaggggtggtggtggaagactggagatctagctatgtggttcctctgttcaaaaaaggaataaaaagtgtagtttcaaactatagacctgtgtcgttgacatcggtttgtagcaaaatattagaatcaatcattaaagaccatctgatagctcacttttcaagaaacgatcttttcgctcaaaatcagtttggatttttaccatctcgttctacgactctccaaatgctcagatttatggatacacttaccagtgcggtggaggaaggagatgaagttgatgtaatttacacggacttggaaaaagccttcgataagatctcgcatgaaaagctactcttcaagctagagagatatggggtgcaccacatggttgttgtgtggattggtaactatttgtcgggccggagtttccaggtgagggtcggtggggagctgtcagactcgttgcctgtgactagcggagtccctcaaggcagcgtacttggtccattgctgtttctcatatatattaacgacatatacactgtttgtgaaaacttattcttgtttgcggacgacacaaaaatatatagagttattagcgaagaggaggaccgcagcaagttacaggtggacgtggataacgtaattgattggttcaacagatagcaaatgaaagttaatatgaacaaatgttgtgcactgcatgtaggggggagacaccacttgaatagctaccagctaacagacctagatggaacaaaatatgaaattccagtttacaactcgttcacggaccttgaaattattgtagattcagatttaaaattcaaagaacacattatctcacgggttaagaaagcaaatatgatgttgggtatcatagcaagaaattttagatatctgtcggattttgcttttgtgagcttgtataagtgtttggtgaggtctcagttggagtatggagtacaggtgtggagcccttatagacaggaatgattgatgcgattgaaggagtacaaagaagagctacaagattaagacaatcctgtagggaccttccttaccaatcgagactagagaaactgaaattgccaagtttgtgttacagaagaaaaagagctgatttgattttgttgtttaagttgttgacaagtggaatgaaagaattagtttgtccttccctttctgtctgcacatattccaagaccagaggacatgaaagaaaactgctgacaagactggcccacaaagattgcagaaagttttatttttgcaacagagtggtccaggaatggaatagtttgccacaggaggttgtgatgtcaagggatatggaggaatttaagagaaacttggacggtttttttgttaatagaatttacactttggattgaggggttggtgcactggtttttgtttttgttttcggtttcggcgaggggccgttggcccgtgctgattttttttcaaataaatcaaataaatggatggatggatggatggatggatggatggatgaatggatggatggatggatggatggatggatggatggatggatggatggatggatggatggatggatggatggatggatggatggaaggatggatggatggatggatggatggatggatggatggatggatggatggatggatggatggatggatggatggatggatggatggatggatggatggatggatggatggatggatggatggatggatggatggatggatggatggatggatggatggatggatggatggatggatggatggatggatggatggatggatggatggacggacggacggacggacggagggacggacggacggatatgttcacttctgtttattttaaaaatgttggttgt*ag*agatttataaacgc**tgc**tatgttcaaaatacgtggcca**tgc**ctt**tgt**gag*gt*aagttaaaattacaactgttatttatcaaatgttatcataaataacgtgtaatatcatcaatgaacattc*ag*aatcaaactata**tgt**gatggtcatcaaatt**tgt**gacacaaagacaaacaaa**tgc**ataa*gt*aattttcatataataaaatttgttattaatgtttgttattaccgtattttcacccatattacacgctcgttatgttcgattttacgtaccacgcaccctcctctgcgtgctataatcgtctgcgtgttatgctagattaaatttacatgaatttgaattccgaccaaaaaaaataaaaaatgtaaatgttaacacagacttttgattgatttttttaaaagctttagactataaaataattttaattttaaatagtttatagttggttgggccttaatgcattgcataaagaactaccgatatgtgcttaaagacacagaagcatatggcaaaacagtttacaatagtaaaaaatcacatgcatgtacttagcaatgtcttcaatctcttaatatttctgctacaaaaactctttgaacacttctcaaaagcatggaataaagtggatacaattttgataatagtgtcagttatgagttttgtgcagtcttaattggaaaacaaaatagcccattctcttggaacccattatgaagattcaaattagtaactgtgatgaattaccaccactgctcttgcttttgctggtggtgagtcacttgattttctgcattgccataagagcatatcatgaaacccattggatgtcaaaaattatatgcagcttttggccatactttcatgttatagttgattcactgtttggacctcaacaattccataccctcacagtgaaggtgtcccagaacatgataacttcagtatctgcttcaagaattgatctcaatttattttgcgcgttataatcgtccgcgtgttaaatgcgtctgcgtgttaatttcgtgaaaatacggtaattttgtttttacgaaaatattggaatttattaattatactatgctcttaatgaaagttgattgt*ag*aaaaatac**tgt**tcggaagattcaaataagtggcca**tgc**tac**tg**tgag*gt*aaattataagttaataactcttagttatcaaatgttaccataaataacgtataatatcatcatttaacattc*ag*gagaatcgtatc**tgt**ctgacatatgaacaa**tgt**gaactgaattctgaaggaaacaaa**tgc**atatata*gt*aatttttttacaatgaaatttaataataattttacttgtgttattaattttatttttacaaaaatattattatcatttattattgtttatttgatattatgttcttatttcagtcaaatctcggtactatgccacctgtcggaaccgagcaagagtggcttagtagcgagcgtggcatactaaccagggcttgtaaaaacggctttattttcttaagttacccatcagtccaaagctttcaagaatcgatggctggcgcatcttctttccttgatcagatcgccgctgacaggcagattctgtcatcttgcctgattcagccacatgagcattccctcatcaagcctgcgtcaacttcatgcgcttcgatggaactccaactttgattgctgcctctattgcatccttcttgctgagaatgcccttcacggtagttcgcttaacttcgaagcccacttcttgctaatctccctggccaacttggactgattcggctatttcttcacggcatcgttgatctcctttttctccttcagcgtaaagtctgttcgcttgcgtttctgagctgacattatggggtgaatatgaaaaaaaaataacaaatgaacgactgatcgaaaagggaaaggttctcgaaccaaccaaagaataacaatcgtctagattttaaaataaaattcaattagacagacattttcacgactcgtgccttttaacagtcacacaatagtggcatagtagcgagagtagggggtggcatagtaaatttattttgtattgaatcctgtcgggaccgagaaaaattggtataatagcgcggtggcatagtaccgaaatttgacaaaatcaaaatgattgttgattgt*ag*aaaaa**tgc**acggaatatgatgaatggcca**tgt**tac**tgt**gtg*gt*aaatttgaagataataatttttagttatcaaatgttatcataaataacgtataatatcatcattgaacattc*ag*aataactcggaaata**tgt**agtccatatgaagag**tgt**gaagtgaattctgaaggaaacaaa**tgc**ataa*gt*aattttatatttttattagtttaatttctttattaattttatttttacataaatattgttataatttattattaatactattatcctcaacgaaaagtgatatgtattaaaaagtgcttcaccaatgatttgggagggagggatgtatggatatatcatttcataccgtgatagatccacttctgtttattttaaaaataaatgttggttgt*ag*tgaattataaactc**tgc**tctgagcgaaattggaacgaatcc**tgc**atg**tgt**gcg*gt*atattataagattataacttttagttatcaaatgttatcataaataacgtttaatattattatatttaacattc*ag*aataaattcaaa**tgt**cgtcgagatgaatat**tgt**gacctgaattatgaaggatacaaa**tgc**aaaa*gt*aatttttatgcaataaaatttactattaattttaaaaattttattaatgatatttttgttattaattttatttttattttcaatttcatttaaatttacacaaataatactgtaattttttattaaaactgttatgctcaactaaaagaatgatatgtaatgaaatgtgcttcaaaactgatgtggggttgatggatggatgggtggatggatgggtggatggatggattttttattgaaacaccacaccagttggaaccgaaaagaagggatggatggatatatggaaggatggatggatataacattacataccgtgataaattcacttctgtttattttaaaaatgaatgttggttgt*ag*ggatttataaacac**tgc**tctgatctaaatgggaaagaacca**tgc**ctc**tgt**ggg*gt*atatgataagatattaacttttatttacaaaatgttatcataaatatcatataatatcgtcgtcgaacattc*ag*ggtaaaagcata**tgc**aatgaaaatgaaatt**tgt**gacctggaaaataacaaa**tgc**aaaa*gt*aattttaaattttaaatttgtttagtaattttaactttttgaataatattgtaatttattattaatccttaatcaatgtatataaaacaatgatcttgtatgtatatttcttatacaaatctttaatcctgaaccgatttgattgaaacttgaaacactttagtacgttgattaaggggtcagtttaatcatatcaccatcccttttcgtactgtcgatgcaacttctgctcaacgcagtcgggtctaaaagcgcatttaggcatggatctctatatgctttcggtgaaagcgctcatggacgacaccactctagtaatgaataggcgaccggcaattcagaacattctgaataagtgcaatagtcttctaggatggtatcgaatggcttttaaacctgcaaagtcgaaaagtcttgctcttgtcaaagggaaagtttgcaacgacacctcgtttgttgcagtgggtgaacgggttcctacagttttcgaggaaccggttaagagcctagtctgagagtttaatgattcgctgaacgataggagccaagagagctgcacagcaggctgatgtgggtttgaaggcaatcggtaaggcacaattgccgggaaggtttaaggagtggttgttccagtttgtgcttcttccaaggctgctgtggccattgacgatctatgataaaggcttgcccacagtcgaggaaagttagcaggtacaccagaacgtggctcgggctagctccagggatgtcatatgttgccctctatagtaagaacgcgaagttaaggctacctcatcggtcccttttattattcaactatgagataatattagcgttatgatggtagccatcacataccgtttgtcattcttaatttctagcggtgtatacgtcgtagcaaataaaagaattgaagtttattaatttgtgtatctattacctgccaaagccggggaaacagtaattttttttatattaaaatttgcaattaatttcatttcttttattaattttgtttttttaactc*ag*aggtg**taa**

GTGATGATGATGAAGTTTGTGATACATGGACAAACGAATGCAAAAAAATATACTGTTCGGAAAGAAATGTGTGGCCA**TGC**TAC**TGT**GAGGAGGATAGAATA**TGT**AAGAAAGATGAAATT**TGT**GAAATAGGAAAGATC**TGT**TATTCAGAAA

**atg**tcgaagctgtttgttgtcctcttggcttcttttggcatcggtctgtctgattgcgagtttgatctgtctcaag

agatttataaacac**tgt**tctgatcgaaatgggactgaacca**tgc**ctc**tgt**ggg

aatggctctaaggta**tgt**ggtcgatatgaagat**tgt**gaagggaacata**tgc**aaag

agatttatgaacac**tgc**tacatcccatacaagattcca**tgc**ctt**tgt**ggg

ggtaatcatgta**tgt**gatgatgatgaagtt**tgt**gatacatggacaaacgaa**tgc**aaaa

aaatatac**tgt**tcggaaagaaatgtgtggcca**tgc**tac**tgt**gag

gaggatagaata**tgt**aagaaagatgaaatt**tgt**aaaataggaaagatc**tgt**tattcagaaaaaaga**tgt**gaactgaatcataaagccaacaaa**tgc**acatttg

aaaaa**tgc**acggatagaaatcgatatcct**tgt**tac**tgt**gtg

aatgactctgaagta**tgt**gggaaatatgaagat**tgt**gaacttaattctgaaggaaacaaa**tgc**atag

agattaaaattcac**tgc**tttgatcgaaaagggaatgaatca**tgc**atc**tgt**ggg

aatgaaaccata**tgt**tctcgaaatgaatat**tgt**gacttgaattctgaaggattcaaa**tgc**aaaa

agaatttgagaccc**tgt**cctgatcgaaagtggtatgaatca**tgc**ctc**tgt**gcg

aatggctctaaggta**tgc**agtcggtatgaaaat**tgt**gaagaaaacgaa**tgc**aaag

agatttataaacgc**tgc**tatgttcaaaatacgtggcca**tgc**ctt**tgt**gag

aatcaaactata**tgt**gatggtcatcaaatt**tgt**gacacaaagacaaacaaa**tgc**ataa

aaaaatac**tgt**tcggaagattcaaataagtggcca**tgc**tac**tg**tgag

gagaatcgtatc**tgt**ctgacatatgaacaa**tgt**gaactgaattctgaaggaaacaaa**tgc**atatata

aaaaa**tgc**acggaatatgatgaatggcca**tgt**tac**tgt**gtg

aataactcggaaata**tgt**agtccatatgaagag**tgt**gaagtgaattctgaaggaaacaaa**tgc**ataa

tgaattataaactc**tgc**tctgagcgaaattggaacgaatcc**tgc**atg**tgt**gcg

aataaattcaaa**tgt**cgtcgagatgaatat**tgt**gacctgaattatgaaggatacaaa**tgc**aaaa

ggatttataaacac**tgc**tctgatctaaatgggaaagaacca**tgc**ctc**tgt**ggg

ggtaaaagcata**tgc**aatgaaaatgaaatt**tgt**gacctggaaaataacaaa**tgc**aaaa

aggtg**taa**

MSKLFVVLLASFGIGLSDCEFDLSQ

EIYKH**C**SDRNGTEP**C**L**C**GNGSKV**C**GRYED**C**EGNI**C**K

EIYEH**C**YIPYKIP**C**L**C**GGNHV**C**DDDEV**C**DTWTNE**C**K

KIY**C**SERNVWP**C**Y**C**EEDRI**C**KKDEI**C**KIGKI**C**Y

SEKR**C**ELNHKANK**C**T

FEK**C**TDRNRYP**C**Y**C**VNDSEV**C**GKYED**C**ELNSEGNK**C**I

EIKIH**C**FDRKGNES**C**I**C**GNETI**C**SRNEY**C**DLNSEGFK**C**K

KNLRP**C**PDRKWYES**C**L**C**ANGSKV**C**SRYEN**C**EENE**C**K

EIYKR**C**YVQNTWP**C**L**C**ENQTI**C**DGHQI**C**DTKTNK**C**I

KKY**C**SEDSNKWP**C**Y**C**EENRI**C**LTYEQ**C**ELNSEGNK**C**I

YKK**C**TEYDEWP**C**Y**C**VNNSEI**C**SPYEE**C**EVNSEGNK**C**I

MNYKL**C**SERNWNES**C**M**C**ANKFK**C**RRDEY**C**DLNYEGYK**C**K

RIYKH**C**SDLNGKEP**C**L**C**GGKSI**C**NENEI**C**DLENNK**C**KKV-

Theoretical pI/Mw: **5.33** / 49886.35

**Htia_mHV13 (this study)**

**chromosom 2 position 1726000 - 1738104** **rev + comp**

**atg**ttctctttgaagctgtgtgttgtcctcttggcttcttttggcatctgtctctctcaac*gt*gagtttg*gt*ttgagtttctcaatgataaagatatatttaataacaatccatttgggatggatggatataacattatcgtaatatattacacttctgtttattttaaaaatgaattttggttta*ag*agaattatgaacac**tgc**tctgatcgaaaaaag**tga**cca**tgc**ctt**tgt**gag*gt*aaattataagcttacaacttttaaataaataatgtcgtcattgaacattc*ag*gatggctctgtc**tgt**gataatgatgaaact**tgt**aaattgatttctgaaggaaacaaa**tgc**aaaa*gt*aatttttatttaataaaattcactaataactttacttaatgtaagaaatatatatcaatatctcttgtgagtcgtgagttataacccacaggcatataaaataaaataataaaataataattataattattataataattattatattataattattataatatatatatatatatataattattattatattataatattatattattattataatattattatattatattatattatattatattatattatattatattatattatattatattatattatattatattatattatattatattatattataatattatattatattataatattatattatattattattattattattattattatatatttttataattattattattattatattattattattattattattattattattattattattattattattattattattattattattattgttattgaaattacccggcggtccgcaaaatagtaagtttaactaataagtttattggagcaagtattcagttaacggaactgaataatcgaagttaagtttttaaggatttgaggtacataatgacgcaaatggatattagggcttctaagttgcctggactactttgacataatactgatacgtctatgcgcaaaatgatactgaatataattctacaacgtttataacattattgttattgttgttgtggttgtcgtcgttgtcgttgttacctcatctgggcgatccaggacatccgtccgctattttccgttgatatagcttctaccctcgcatgctgccttgagtccaaatcatcttaattattcaacggactataatcaaataaataaataaataagcacgctgccttgttctgtctcgcctatactactgtacctatcttctctatggcccatccgctcttttaattatatccttgcatcgcgtccaaaacaagcttgccaaacttgttctcttcaacccaaccatcaataacacgtactgcctgaagagattgcactggttgccgattcatagtcgtatcaccttcaaaatcgtcctcatgacatatcacacactagcaacatccatcgcaccttatctccattatctaatagatcgccgccaagcaccagtcctcgttcatcgtcaaccactcaacttcaccaaccggtacataggtcatccctcccaaacagaggcttctcgcacacctctccagctgcctggaacagacttcttcccaccgtcagagcccagccatccctggagctcttcaaacgccacctcttcaggacttcatgggaaagcgaagacacaaggcgcctctgacgctatgcacatggcgctttataaattaaatattgttattattttattaattttatttttacaaaaattgtattgaaatttattattagtgctatgctagtaatatttatattaaaaatgaatgttgtttgt*ag*aaaaaaac**tgt**tcagaaataaatgagtggcca**tgc**tac**tgt**gag*gt*aaattataacataataacttttacactttttaactttcagtcatcaaatgttatcataaataacgtatactatcgtcattgaacattc*ag*ggtgaaactata**tgt**gttgagtttgaagtt**tgt**gatacaaagacaaacaaa**tgc**aaaa*gt*aattgtcatataataacatttgttattaatgtttttttatttattttaaattgtattgaaatttattattaaaactatgcacctaatatttatattaaaaatgtaagtggattgt*ag*aaaaaaaa**tgt**tcagaaaaacatgagtggcca**tgc**tac**tgt**gag*gt*aaattataacataataacttttagttagcaaatgttatcataaataacgtataatatcatcattgaacattc*ag*ggtgaaactata**tgt**gttgggtttgaagtt**tgt**gatacaaagacaaacaaa**tgc**aaaa*gt*aattctcatataataacgcttgttattaatgttttttattaattttatttttacaaacattgtattgaaatttattactaaaactatgctcctaatatttatatttaaaatgaatgttccttgt*ag*aaagatac**tgt**tcagaaaaaaatgagtttcca**tgc**tac**tgt**ggg*gt*aaattataacataataacttttagttagcaaatgttatcataaataacgtatactatcgtcattgaacattc*ag*aatcgttat**tgt**tacgaagatgaaatt**tgt**gaactaggagagattaacaaa**tgc**aaaa*gt*aattctcatataataacatttgttattcgtgttttttattaattctatttttacaaaaattgtattgaaatttattattaatactatgctcttaatatttattaaaattatattaaaaataaatgttgtatgt*ag*aaaaatac**tgt**tcggaaaaaaatgaatggcca**tgc**tac**tgt**gag*gt*aaattataacataataacttttagttatcaagtgttatcataaataacgtataatatcatcattaaacattc*ag*gaggatcacaaa**tgt**gataaacatgaaatt**tgt**gaactaggagagattaacaaa**tgc**aaaa*gt*aattttcatatcataacatttattattaatgttttttatttattttaaattatattgaaatttattattaaaactattctcttaatacttatattaaaaatgaaagttgattgt*ag*taaaatac**tgt**tcggaaataaataagtta**tgt**tac**tgc**gag*gt*aaattattagatattaaattttatctatcagatgttatcataaataacgtataatatcattattgaacattc*ag*ggggatcataaa**tgt**aaggaggatgaaatt**tgt**gaactaggagagattatcaaa**tgc**aaaa*gt*aattttcatatcataacattcgataataatgttttttgttaattttattcttacaaaaattgaattgaaatttattattaacactatgttcttaataatataataataatgtatgtcgtcattatgaaaattgaactttctttattggctattatgctcaacgaaagagtgatatgcaataaaacgggtttcaaaaaggatatgtatgaatggatggatggaaggacggatggatatatggatataccattatatattgtgatatattaacttctgttaattttaaaaatgaattttggttta*ag*ggaattataaacac**tgc**tccgatccaaataagtggcca**tgc**ctt**tgt**gag*gt*aaattacaaaattacaatttttaaataaataatgtcgtcattgaacattc*ag*gatggctctgcc**tgt**gataatgatgaaatt**tgt**aaattaatttctgaaggaaacaaa**tgc**aaaa*gt*aatttttatttaataaaattcactattaattatactcaatttaagaaatgtatatcaataattcttgtgagtcgtaagttatgacccatatgcacgcaaacttaagttaaaattattattattattattattattattattattattatttttattactattattattattattattattattattattattattattattattattattattattgttagaaacaatctgcacatccgataaataatcagcccgaaaaaacccttccaaaaaatttttaaagtcccccatcatgggtcgggatctccacgggactataagttgaatttaacttaaataaataactgaaactgtgcatgcacgctaaatcaaaaaacgaaaggaacacaaaggaaaatgatacaaagtaaataattagttctgttgatttcagtcaacaactcttcttctgtaagcaacagtgtggaaggcaccaggttctcctctgatcctcacaacccacttcttgcttcccccctttaagttgatctcttttaatttgtcttcaaacaatttttcctgtcttcctttgacaaatcatgacttagaataactttgttgaagacttcactgtctttcaatttgttgctgttgtccaaaaccatgttcttggtcaacaggttgtcaaatttaatcaacacaggccttagctttgaggcatcttcaactttttttccaattctatacatttctagaatttctttttcaatatttttccctgttacttctttcagaaacttggatacaagttctttttcttcggactttccagaatcttctgtcaggttgtagactatgacattattctgtcttttttgcctctcattgtttatttctactttatactttgtatctgtcacagagccttgaacctttatgatcaaggtttcaaccttgttttcaaataaacttgaccaaaatttggtttcttgcaaatggatattagggcttctaaattgtctggactactttgacataatgctgatacatctatgcgcaaaatgattctgaatataattctacaacgttaataacattattgttattgttgctgttgtcgtcgttgtcgttgtcacctcatctgggcaatccggcacatcggctccattttccgtttatataacttctactctcgtatgctgccttgttctatctcgcctagactattgtaattctctatggtacatatgcttctttaattcaatctttgcaaagcttgccaagcttgttctcttcaaccctaccctcaatagcacggactgcctgaagagattgcactggttcccgattcatagtcgtatcaccttcaaaatcgccctcatgacatattacacactagcaacatccatcgcacctcatctccatcacctaatacagtgatccccaacccccggttcgcaaaccggtaccggtccgtggatcaaatggcacagggccgcccatggaacaataaattatttccattttattaaaaaaatttaaaatagtttgtagggcttgataacaaccgcctccatctctttcttagcgtattcggtacaaattattgagcctgattttgtaacattctaagaataatgattaacttatcaagaaaattgatacactttctaattaagtaattacagtgaattaatttaattattttctttttttccatttcatctttttcgttttcgagcgtttcgccacttttgtaagcaaggatttcgtaacgtgtgtttcgatcttctccctgttcaccggtaattatcatatttaatagtgcttggtttacttgtatattgtatcaaatttaaccgaaagattagcaaaaatgagtacgaaacagacgtctttagaaagtttccttggaataaagaaaacgcccagtgaagggacagaagagccttcaacatcaaagaaacgtacaacttttagcagacaatatcatgagtcttacttgaaatatggatttgtcggagctggtgattcccacgcaccaattccgttctgcatagtttgcggcgaccatctctctaatgacgcaatggaaccttcaaaactgcctcgccacttgaactccaagcactctggattaaaagacaagcccctggagtattttgaaggaaaaaaacgggaacacgaaagacaaaaacatttattagggccaccacattattaaatgacaatgcactcagagcatcatatttggtggctaaccgcattgctaaggctaaaaagccattcacaatcggtgatcttgccctccactaaaaacatttgctgtgaaattctaggagaggctgctgcagaaaaaatagcacatgtgcctttgtcggctagcactgtgactaggcgccttgaggaaatatccgaagacacaattgctggagatgattaatgaataaccatgctacgcattccaagttgacgaaagtacagatagcgacaacaaggcaatactacttgtttatgtgctttatctatatcaggaggatgtgcatgaggatttgctatgtgcactatctttgccaaccaacaccacataagcagaactgttcaatttactgaatggttatatatcagaacaaataaaatggttcttttgtgcacagacggagctgctgccatgatcggacgactgtcaggtttaactgctcggattaaagatgttgcatctgaaagtgaatctactcattgtatcattcacaggaaagctggaaagccgaaaattggcatcgtattgattgatgtcgttaaagttattaacctcaccaaaacacacgcccttaactcgcgcctgtttgaggagatggacgcagagcacgttgtctgctcttacaaacagaaataagatggctatccagaggcaaatcgcgtaccagagtatttgaattacgagagccattgcaaagatttctctcagaaaagaagtcaccagcacatttcggtgacaaggtatgggtcgcaaaactagcttacttgtatgacatattcagcctgctcaataaacttaatctgacccttcagcggaaaatgacaactattgttggcagacaaattagctgcatttaaagccaaaatggagttgtggggaaaacacgtgaacataggcattttggacctgtttcaaacattagcggggattttatgcgagactgagcctgagcttccagttggtgacggatcacctgtctttgcttttaaaagaattcgagcgctacttccaaaccacaaaagacccacgaactggtaaggaatggatccgcgacccatttcatatgggccatttgtcaacaaaccaggcgaatctagcatgtctgagcaagaagaagatcaactgctgaaaattgctaatgacggcgtccttaaaactgcgttccagaaaacaactctgccggtgttctggattaaagtcttggcggaatacacagagattgcaaccacagcacttaaatccctgttgccatttccgacatcctatctttgtgaagcggggttttgacagaaaccaaaacaaagcaacggaataaactggacataagtaacgtacttcggatgtcattgtctactattacccccagatggaaccgtctcattgtaaagaaacaagctcggggttctcattgatttagcgttctaatgagttaaattttaaatcattttatattcaatttttgagtaactgtatgtatttttgaaggcatgtttaaatacaattaaatcaaaacgatccaaaatataaacaatcaactccccctccccccccccccgaacggtccgcggcgataaaaggttggggacaactgacctaacagatcgccgcactgcaccagtcctcgttcatcgtcaaccatcgatgcctcccaaacagaggcttctcgcacgcctctccagctgcctggaacagacttcttcccaccgtcagaacccagccatccctggagctcttcaaacgccacctgaagatttcttcagtacttcatgggacagcgaagacacaaggcgcctctgacgctatgcacatggcgctttataaattaaatattgttattattttattacttttatttttacaaaaattatattgaaatttattgttagtgctatgctagtaatatttatattaaaaatgaatgttgtttgt*ag*aaaaatac**tgt**tcggaaataaatacgtaccca**tgc**tac**tgt**gag*gt*aaattataacataataacttttacactttttaacttttagtcatcaaatgttatcataaataacgtatactatcgtcattgaacattc*ag*ggtgaaactata**tgt**gctgggtttgaagat**tgt**gatacaaagacaaacaaa**tgc**aaaa*gt*aactttcatataataacatttgttattaatgttttttatttattttattttatttattttaaattgtattgaaatttattattaaaactatgcacctaatatttatattaaaaatgtaagtggattgt*ag*aaagaaaa**tgt**tcagaaaaacatgagtggcca**tgc**tac**tgt**gag*gt*aaattataacataataacttttagttagcaaatgttatcataaataacgtataatatcatcattgaacattc*ag*ggtgaaactata**tgt**gttgggtttgaagtt**tgt**gatacaaagacaaacaaa**tgc**aaaa*gt*aattttcatataataacgcttgttattaatgttttttattaattttatttttacaaacattgtattgaaatttattactaaaactatgctcctaatatttatatttaaaatgaatgttccttgt*ag*aaagatac**tgt**tcagaaaaaaatgagtttcca**tgc**tac**tgt**ggg*gt*aaattataacataataacttttagttagcaaatgttatcataaataacgtatactatcgtcattgaacattc*ag*aatcgttat**tgt**tacgaagatgaaatt**tgt**gaactaggagagattaacaaa**tgc**aaaa*gt*aattctcatataataacatttgttattcatgttttttattaattctatttttacaaaaattgtattgaaatttattattaatactatgctcttaatatttattaaaattatattaaaaataaatgttgtatgt*ag*aaaaatac**tgt**tcggaaaaaaatgaatggcca**tgc**tac**tgt**gag*gt*aaattataacataataacttttagttatcaagtgttatcataaataacgtataatatcatcattaaacattcagg*ag*gatcacaaa**tgt**gataaacatgaaatt**tgt**gaactaggagagattaacaaa**tgc**aaaa*gt*aattttcatatcataacatttattattaatgttttttatttattttaaattatattgaaatttattattaaaactattctcttaatacttatattaaaaatgaaagttgattgt*ag*taaaatac**tgt**tcggaaataaataagtggcca**tgc**tac**tgt**gag*gt*aaattttaagataataaatgttatctatcagatgttatcataaataacgtataatatcataattgaacattc*ag*ggggatcataaa**tgt**aaggaggatgaaatt**tgt**gaactaggagagattatgaagaaaagaaatcaactggatatcactgaa**cggggagac**ttaagtctaaaattgtctaaactgctacctaatataagacctctg**tgt**agcaagcatcaagtacaaggatctcac**tag**ctgtgtacaatttagtaaaggataattaaaattatgacgggttattgaaatactttcattaaaactgttctatatttatgcagttcagttctgctttgctgacttaaactgagcttaatgattttattttgaaacggtaataaaactgtcataaatgaatgtcggatgtcgatttcattgtgttgtttatcacgaaaaactttgctaagctacggaagcagtaaatttttgaagatttgattcgatttgggatttgatcatttgggatttgatttgattcgaaattttgggatttgatttgagatttggctgttgagattagattattttacaaatgatttgagatttgaccatggagatttaattcgagatttgcctatcactgataaaagctcttgaaatttacattcttgaaagcacaaatttacagaggtgcgaggattgtctgcaaaaatattatgaatattgtaaattaaattcttatagtttttatataatttaaaattcgttctaaattataaaacaatataaattaaaagcttacttcacgaaccagcgtacacccataaaaagctttttggctataaaacgcaaaataatgtatcgattgttttgcaaaaaagactaaaccgaccccctaggtcaacgtgctcaagtgtttaaagtttcaatcaaagcaatccagaattaaagatgtttaagaaatatacatataaacaagatccttgttttatttatacagattcgttattacaaaaaatc*ag*aatttagtttaaacatttcacaacac**tgt**tcagaaataattcagtttcca**tgc**tac**tgc**aag*gt*aaattgtaagataataacttttagttatcaaatgttatcataaataacttataatatcatttcattaatatcattcagaattactctgaaatatgtgctgaaaatgaagaatgcaaaatgaattctgaaacaaatgcataggtaatatgtataagttattaattttaaatttttattaatgtcaatgcatgctctaaaataatgtaaaataaaatattgcgtcagctcgaggctatctgttttaattatgatatcataaaaataattggctaaagatattatctctgctaaatattaatggaatttgtttggtctccagggaaaaacaccattgattcgctggcagaattctgaataagtaagcaacaggtaaggaagtctgaacatttgtggaaaacttaaactttaatatttttggtcacaaattatatttgtaattaaaaaatgtaatttttatatattaattcgcagtttttaataataacctctgggtgtttggtgcgaataattctgaaattaaacgtcaaaaatgttattaattataagaaatacaatacattttcatcgataaaggttacaaaacattttttaaagttatttaagttgccttattataagaattaattttattatgtcgagcaacaaaatccgtaattaatcgtaaatgttatttatatttatgtattatataaatgtcattaattatgagaaatcccttaaattttcatatttagaaatgaaaaaacgaataactattcaaataatataaagttgtcattaaaccacctctcccatgttgccttgctttttaacaaatacgagtgttgaagctgcagccattaaaggcgtgttgcaattatcttttcacatacttttcattttttcagtggtttaaaacttaaaagcaattgtaaaatatattttatttttattaaaattattttgtttgtaaaattaactttattgcagagatttaattaattgttaaattgtggagtaacgacttaaatcatctatgatgatgatgatgatgacgataagtttgtaagaacgctgcttgcttttaaaaataattaaactaatagtatgcatgtaacaacctattgttattattagcgattattaaattaatttatagaaaaacgtttacatttcatacgtaataataattttgaaataatttaatatctattaaatgaatgtagcgtacgaagaaaagtacgaaggtaaataaacgtttgttgacctgttcagttagtggttgtttttgttgaagattgcataagaaaataattttgatgttcaatgaaattaagttatcgtttctaaggtattacaacaggctcatagtttgtataacatgcattgtatatgtttctattaataaatcaaagactgataatccacctgtcgacgattatataaaactgttatttacgtttcagaagttctttaataatgtcacaaaatctgattcacagaggctttccaattattaatggaaatcaaacgcttaattatcgacttaagtaccaagtcatcttaattaagcaaaactcactttaaataaattattaattatataaatttaaattacttattaattaaataaatttaaattacttataaattcttgtctaatattatatttaaaaatatacccaaaatccgtgacagattatagtgacatcccgtgatagtccaagtaccattaactttaaaaaaaataattaactaattatttaataatggtgttattgataattaataattacttaaattaaaaaaataaataaataaatcttgacaaataattgaaattgaaaaaaaattaaaaaaaaagtgatt

GTCAAAA**ATG**TTTTCTTTGAAGTTGTTTGTTGTCCTCTTGGCTTCTTTTGGCATCCGTCTGTCTCAAGGGAATTATAAACCC**TGC**TCTGGTCGAAATAAGTGGCCA**TGC**CTT**TGT**GAGGATGGCTCTGTC**TGT**GATAATGATGAAACT**TG**

MFSLKLFVVLLASFGIRLSQGNYKP**C**SGRNKWP**C**L**C**EDGSV**C**DNDET

**atg**ttctctttgaagctgtgtgttgtcctcttggcttcttttggcatctgtctctctcaac

agaattatgaacac**tgc**tctgatcgaaaaaag**tga**cca**tgc**ctt**tgt**gag

gatggctctgtc**tgt**gataatgatgaaact**tgt**aaattgatttctgaaggaaacaaa**tgc**aaaa

aaaaaaac**tgt**tcagaaataaatgagtggcca**tgc**tac**tgt**gag

ggtgaaactata**tgt**gttgagtttgaagtt**tgt**gatacaaagacaaacaaa**tgc**aaaa

aaaaaaaa**tgt**tcagaaaaacatgagtggcca**tgc**tac**tgt**gag

ggtgaaactata**tgt**gttgggtttgaagtt**tgt**gatacaaagacaaacaaa**tgc**aaaa

aaagatac**tgt**tcagaaaaaaatgagtttcca**tgc**tac**tgt**ggg

aatcgttat**tgt**tacgaagatgaaatt**tgt**gaactaggagagattaacaaa**tgc**aaaa

aaaaatac**tgt**tcggaaaaaaatgaatggcca**tgc**tac**tgt**gag

gaggatcacaaa**tgt**gataaacatgaaatt**tgt**gaactaggagagattaacaaa**tgc**aaaa

taaaatac**tgt**tcggaaataaataagtta**tgt**tac**tgc**gag

ggggatcataaa**tgt**aaggaggatgaaatt**tgt**gaactaggagagattatcaaa**tgc**aaaa

ggaattataaacac**tgc**tccgatccaaataagtggcca**tgc**ctt**tgt**gag

gatggctctgcc**tgt**gataatgatgaaatt**tgt**aaattaatttctgaaggaaacaaa**tgc**aaaa

aaaaatac**tgt**tcggaaataaatacgtaccca**tgc**tac**tgt**gag

ggtgaaactata**tgt**gctgggtttgaagat**tgt**gatacaaagacaaacaaa**tgc**aaaa

aaagaaaa**tgt**tcagaaaaacatgagtggcca**tgc**tac**tgt**gag

ggtgaaactata**tgt**gttgggtttgaagtt**tgt**gatacaaagacaaacaaa**tgc**aaaa

aaagatac**tgt**tcagaaaaaaatgagtttcca**tgc**tac**tgt**ggg

aatcgttat**tgt**tacgaagatgaaatt**tgt**gaactaggagagattaacaaa**tgc**aaaa

aaaaatac**tgt**tcggaaaaaaatgaatggcca**tgc**tac**tgt**gag

gatcacaaa**tgt**gataaacatgaaatt**tgt**gaactaggagagattaacaaa**tgc**aaaa

taaaatac**tgt**tcggaaataaataagtggcca**tgc**tac**tgt**gag

ggggatcataaa**tgt**aaggaggatgaaatt**tgt**gaactaggagagattatgaagaaaagaaatcaactggatatcactgaa**cggggagac**ttaagtctaaaattgtctaaactgctacctaatataagacctctg**tgt**agcaagcatcaagtacaaggatctcac**tag**

CTTTAGTGCTGTTAATGATTTGCTACTGAAGAAAAGAAATCGACTGGATATCACTGAA**CGGGGAGAC**TTAAGACTAAAATTGTCTAAACTGGTACCTAATATAAGATCTCTG**TGT**AGCAAGCATCAAGCACAAGGATCTCAC**TAG**CTGTG

FSAVNDLLLKKRNRLDITE**RGD**LRLKLSKLVPNIRSL**C**SKHQAQGSH-

TCCA**TGC**TAC**TGT**GAGGAGGATCAAAAA**TGT**AAGGAGGATGAAATT**TGT**GAACTAGGAGAGATTATCAAA**TGC**AAAATAAAATAC**TGT**TCGAAAATAAATGAGTTTCCA**TGC**TAC**TGT**GAGGAGGATCAAAAA**TGT**AAGGAGGATGAAAT

P**C**Y**C**EEDQK**C**KEDEI**C**ELGEIIK**C**KIKY**C**SKINEFP**C**Y**C**EEDQK**C**KEDE

MFSLKLCVVLLASFGICLS

QQNYEH**C**SDRKK-P**C**L**C**EDGSV**C**DNDET**C**KLISEGNK**C**K

KKN**C**SEINEWP**C**Y**C**EGETI**C**VEFEV**C**DTKTNK**C**K

KKK**C**SEKHEWP**C**Y**C**EGETI**C**VGFEV**C**DTKTNK**C**K

KRY**C**SEKNEFP**C**Y**C**GNRY**C**YEDEI**C**ELGEINK**C**K

KKY**C**SEKNEWP**C**Y**C**EEDHK**C**DKHEI**C**ELGEINK**C**K

IKY**C**SEINKL**C**Y**C**EGDHK**C**KEDEI**C**ELGEIIK**C**K

RNYKH**C**SDPNKWP**C**L**C**EDGSA**C**DNDEI**C**KLISEGNK**C**K

KKY**C**SEINTYP**C**Y**C**EGETI**C**AGFED**C**DTKTNK**C**K

KRK**C**SEKHEWP**C**Y**C**EGETI**C**VGFEV**C**DTKTNK**C**K

KRY**C**SEKNEFP**C**Y**C**GNRY**C**YEDEI**C**ELGEINK**C**K

KKY**C**SEKNEWP**C**Y**C**EDHK**C**DKHEI**C**ELGEINK**C**K

IKY**C**SEINKWP**C**Y**C**EGDHK**C**KEDEI**C**ELGEIMKKRNQLDITE**RGD**LSLKLSKLLPNIRPL**C**SKHQVQGSH-

Theoretical pI/Mw: **5.83** / 53033.53

**Htia_mHV14 (this study)**

**chromosom 2 position 1745847 - 1751224** **rev + comp**

**atg**ttgaagctgtttgttgtcctcttggcttcttttggcatcggtccgtctcaag*gt*gagtatgatctgagtttctgaacgaaagagtgatatgtaataaatagagcttcaccaatgatttggtatggaaggattgattgatttgatagatggatgcatggatgtaaggatggatagatataacattacgtaccgtgataaattcacttctgtttattttaaaaatgaatgttggttga*ag*tgatttataaacac**tgc**tctgatcgaaatgggaatgaacca**tgc**ctc**tgt**ggg*gt*aagttataagatttacaaaatataatcataaattacgtgtaatatgacatcattaatttcttcattgaacattc*ag*aatggctctaaggta**tgt**ggtcgatatgaagat**tgt**gatgaaaacaaa**tgc**aaaa*gt*aattttttttgctaaaaatttactattaattttacaattgttattaattttatttttgttattaatttatttttattaataatttcgatattttaaattttatttttacaaaataatattgtaatttattattactactattattatcgatgaaagagtaatacttaacaatgacttggaatggatggatggatggatagatggatggatggatggatggatggatggatagatggatggatggatggatggatggatataaccttacataccgtgatattttatttcctctttattttaaaaatgaatgttggttgt*ag*cgatttatgatccc**tgt**tacattcggcgtaggaaacca**tgc**ctt**tgt**ggg*gt*aagttattagattataactgttatttatcaattgttaaataacctataatatcgtcattgaacattc*ag*gataatcatgta**tgt**aagaacaacgaaact**tgt**gatacgtggacaaacaaa**tgc**aaaa*gt*gattttcatataataaaatttgttaataatgttttttaatgacttcatttaaaaaaaaataatattgtaatttattattgatactatgctcttaatatttatgataaaaattaatgttggttgt*ag*aaaaatac**tgt**tcgctaagaaatgtgtggcct**tgc**tat**tgt**gag*gt*aaattataagattataacttttatttatgagatattatcataaataacgtttaatatcatcattgaacattc*ag*gaggatagaata**tgt**aagaaagatgaaatt**tgt**gaaataggaaagatc**tgt**gattcagaaaataga**tgt**gaactgaattctgaagccaacaaa**tgc**ataa*gt*aacttttataagttattaattttatatttttatttacttcatttttttataaattttatttttatctaaacattattatactattatcctcaattaaaatgtaataaaagtgccgcaccactgatttgggatggatggatggatagatataacattacataacgtgagttgaacatcctgttaattctaaaaatgatgttggttat*ag*agattagtattcga**tgc**tctgatcgaaaaaggaatgaacct**tgc**ttc**tgt**ggg*gt*aaattataagattacaacttttatttacaagatattatcattaataacctataattcctacattgaacattc*ag*aatgaaactata**tgt**tatgaacatgaatta**tgt**gacctgaattctgaaggaaacaaa**tgc**aaaa*gt*aatttttatgcaataacatttagtgttaattttacaaatgttattaattacatttttgtgacttgaattccaaaggaaataaataaataataatattatttaataaatattgtgttcttagtatttacattaaaaattattgttggctgt*ag*aaaaa**tgc**aagagaaataatgaatatcct**tgc**tac**tgt**gtg*gt*aaattataagataataactttcagttatcaaatgttatcataaataacatataatatcatcattgaacattc*ag*aataactctaaagta**tgt**ggtgaatatgaagat**tgt**caactgaattctgaaggaaacaaa**tgc**atag*gt*aatttttataagttagtaattttatatttttattaattttaattttatttatgttattaattttaattttatttatgttattaattttatttttattataaatgtcattttgttttaaatttacacaaataattttataatttattatcaatattattacgctcaaaagatactattatgaaagagtggatgggtggatggatggttttatgggtgaatagatacatgtatgtaaggatggatggatgtaatattacattatattgccgtgatatattcacttctgtttattttcaaaataaatgttgtttgt*ag*agaatattagaccc**tgc**tctgatcaaaattggatggatcca**tgc**ctc**tgt**ggg*gt*aagttataagattacaacttttatttacaaaatgtaatcataaacaacgtatatcataaattaaaattaatttaatttaatttttttattaaatcaaacatacaaggcgttatatacacagaaaaacaaaaacgggaaagcactaaataaattcaaataaatgcaaagggccagacttagggtccgctggctatatacaaaatcataagctatacatttttactcaataaatattgtttacacgatattttgaattgtaaataaagtaatttgaaagggcatgtgccgaactgagttagattcttgatagatgaaggtatggaggagtaaagtctgtttaatataaataagtataatatgatatgatatcattaatatcttcattgaacattc*ag*aatgactctgaggta**tgt**gatgaatttcaatac**tgt**acaggaaacata**tgc**aaag*gt*aatttttaattaataaaatttactatttattttgattgcgttattaattttatattaattgttattaatttcatctttttttttattattagtttatttttattattagttttattttcttaaaatttacttttatatcaataatattgtactttattatgtaatgctaaacttaatagtgatacttaacataaagtgcctcaccaatgatttggaatggatggatgaatggagggatgggtggatggatatagcataaataccgtatacatatcgtgataaattcacttctgtttatttttaaaatgaatgctggttgt*ag*agatttacagaaac**tgt**tctaatcgaaaggggaatagaccaataaaa**tgc**cgc**tgt**ggg*gt*aagttataggatttatcaaatgttatcataaataacgtgtaatatcatcaatgaacattc*ag*aatcaaaccgta**tgt**ggtgatcatcaaatt**tgt**gactcaaaaacaaacaaa**tgc**aaaa*gt*aattttcatataataaaatttgttattaatgtttgttattaattttattttcacaaaaataatgttgtaatttattattattaatactatgcttttaatattcatattaaaaatgaatgttggttgt*ag*aaaaatac**tgt**tcggaagattcaaatgagcatcca**tgc**ttc**tgt**gag*gt*aaattattagataataactttctgttaccaataaatatattaataacgtatgatatcattctttgaacattc*ag*gagaatcgcatc**tgc**tctttaactgaaaaa**tgt**gaattgaattctgaaggaaacaaa**tgc**atatatg*gt*aattttttataatgaaattttataataattttacttatgttgttaaatttatttttattaatatttataattaatttttacaaaagtaattttataatttattattatataatttatattatgttcttttttatatttaatatgaatatttttttt*ag*aaaaa**tgc**acggaaagaaatcaatttcct**tgc**tac**tgt**gtg*gt*aaattataagataataactttcagttatcaaatgttatcataaataacgtataatatcatcattgaacattc*ag*aatgactctaaagta**tgt**ggtgaaaatgaagtt**tgt**gaactgaat**tgt**gaaggaaacaaa**tgc**atag*gt*aatttttataagttattaattatatatttttattaattttaattttacataactattattttaatttactgttaatactattatcctcaacgaaaaagttttatgtaatggattggtggatggatggatggatataacattacatatcgtcacttctgttcattttaaaaatgaatgttggttgt*ag*agata**tgt**aatagaaag**tgc**ctcaccaatgatttgggatggatggatgga**tgt**aacattacatacc*gt*gataaattcacttctgtttattttacaaatgaatgttgattgt*ag*agatttataaacac**tgc**tctgatcgaaagtggaatgaaaaa**tgt**ctc**tgt**gcg*gt*aaattttaagatcacaacttctatttatcaattgttttcataaataacatataatatcctcattgatgattt*ag*aatgaaaccatg**tgt**tctccaaaggaattt**tgt**gacctgaattctgaaggaaacgaa**tgc**aaaa*gt*aatttttatgcaataaaatttactattaattttacaattgttggatggatggatggaaggatgaatggatggatggatggatataacattacataccgtgataaattcacttctgtttattttaaacatgaatgttggttgt*ag*ggatttataaacac**tgc**tctgatctaaatggaaatgaacct**tgc**ctc**tgt**ggg*gt*aaatgataagattacaacttttatttatcaaatgttatcataaataacatataatatcaacattgaactttc*ag*ggtaaaagcata**tgc**aatgaacatgaaatt**tgt**gacctggaaattaacaaa**tgc**aaaa*gt*aatttttaaattataaaattatttattaattttaactttaaaaaaaataaaattgtaatttattaataatctatatataaaaaacaaggatcttgtatgtatatttcttatacaaatctttaatcctgaaccgatttgattgaaacttgaaacactttagtacgttgattatggggtcagtttagtctacttgaaaaaaaatcgataaattatttttcgtattattgccaataaacctctcctgggcttattatctcgagagcgttcgcgcagttttcaaaactggtgagcttaggcgcttactgattaatattcatgtgcgtaaacatttagtttatattgtaaattttccgttttattttttaaaatttacattttttattacttatctatgatttaatattagtgttatgatggtggccattttgtcattcataatttctagcgatgtattcgtcgttgcaaataaaagaattgaagtttattaatttgaatatctattacc*ag*gcaaaggaggaaacgaggagacggggaaa**tag**

**atg**ttgaagctgtttgttgtcctcttggcttcttttggcatcggtccgtctcaag

tgatttataaacac**tgc**tctgatcgaaatgggaatgaacca**tgc**ctc**tgt**ggg

aatggctctaaggta**tgt**ggtcgatatgaagat**tgt**gatgaaaacaaa**tgc**aaaa

cgatttatgatccc**tgt**tacattcggcgtaggaaacca**tgc**ctt**tgt**ggg

gataatcatgta**tgt**aagaacaacgaaact**tgt**gatacgtggacaaacaaa**tgc**aaaa

aaaaatac**tgt**tcgctaagaaatgtgtggcct**tgc**tat**tgt**gag

gaggatagaata**tgt**aagaaagatgaaatt**tgt**gaaataggaaagatc**tgt**gattcagaaaataga**tgt**gaactgaattctgaagccaacaaa**tgc**ataa

agattagtattcga**tgc**tctgatcgaaaaaggaatgaacct**tgc**ttc**tgt**ggg

aatgaaactata**tgt**tatgaacatgaatta**tgt**gacctgaattctgaaggaaacaaa**tgc**aaaa

aaaaa**tgc**aagagaaataatgaatatcct**tgc**tac**tgt**gtg

aataactctaaagta**tgt**ggtgaatatgaagat**tgt**caactgaattctgaaggaaacaaa**tgc**atag

agaatattagaccc**tgc**tctgatcaaaattggatggatcca**tgc**ctc**tgt**ggg

aatgactctgaggta**tgt**gatgaatttcaatac**tgt**acaggaaacata**tgc**aaag

agatttacagaaac**tgt**tctaatcgaaaggggaatagaccaataaaa**tgc**cgc**tgt**ggg

aatcaaaccgta**tgt**ggtgatcatcaaatt**tgt**gactcaaaaacaaacaaa**tgc**aaaa

aaaaatac**tgt**tcggaagattcaaatgagcatcca**tgc**ttc**tgt**gag

gagaatcgcatc**tgc**tctttaactgaaaaa**tgt**gaattgaattctgaaggaaacaaa**tgc**atatatg

aaaaa**tgc**acggaaagaaatcaatttcct**tgc**tac**tgt**gtg

aatgactctaaagta**tgt**ggtgaaaatgaagtt**tgt**gaactgaat**tgt**gaaggaaacaaa**tgc**atag

agata**tgt**aatagaaag**tgc**ctcaccaatgatttgggatggatggatgga**tgt**aacattacatacc

agatttataaacac**tgc**tctgatcgaaagtggaatgaaaaa**tgt**ctc**tgt**gcg

aatgaaaccatg**tgt**tctccaaaggaattt**tgt**gacctgaattctgaaggaaacgaa**tgc**aaaa

ggatttataaacac**tgc**tctgatctaaatggaaatgaacct**tgc**ctc**tgt**ggg

ggtaaaagcata**tgc**aatgaacatgaaatt**tgt**gacctggaaattaacaaa**tgc**aaaa

gcaaaggaggaaacgaggagacggggaaa**tag**

MLKLFVVLLASFGIGPS

QVIYKH**C**SDRNGNEP**C**L**C**GNGSKV**C**GRYED**C**DENK**C**K

TIYDP**C**YIRRRKP**C**L**C**GDNHV**C**KNNET**C**DTWTNK**C**K

KKY**C**SLRNVWP**C**Y**C**EEDRI**C**KKDEI**C**EIGKI**C**DSENR**C**ELNSEANK**C**IK

ISIR**C**SDRKRNEP**C**F**C**GNETI**C**YEHEL**C**DLNSEGNK**C**K

KK**C**KRNNEYP**C**Y**C**VNNSKV**C**GEYED**C**QLNSEGNK**C**I

ENIRP**C**SDQNWMDP**C**L**C**GNDSEV**C**DEFQY**C**TGNI**C**K

EIYRN**C**SNRKGNRPIK**C**R**C**GNQTV**C**GDHQI**C**DSKTNK**C**K

KKY**C**SEDSNEHP**C**F**C**EENRI**C**SLTEK**C**ELNSEGNK**C**I

YEK**C**TERNQFP**C**Y**C**VNDSKV**C**GENEV**C**ELN**C**EGNK**C**I

EI**C**NRK**C**LTNDLGWMDG**C**NITYQI

YKH**C**SDRKWNEK**C**L**C**ANETM**C**SPKEF**C**DLNSEGNE**C**K

RIYKH**C**SDLNGNEP**C**L**C**GGKSI**C**NEHEI**C**DLEINK**C**KSKGGNEETGK-

Theoretical pI/Mw: **6.05** / 52124.85

**Htia_mHV15 (this study)**

**chromosom 2 position 1922211 - 1926178**

**atg**ttctcattgaagctatttgttgtcctcttggcttcttttggcatcggtttgactcaag*gt*aagtgtgatctgagtttctgaacaaaagagtgatatttatattatttatattgcgtacttaagtacttcaaatccttaaacacttaacttctatcattcagttccgttaactgagtacttgctccgataaacttattagttaaacttactaatttgcggacccccgggttatttcaatatttttacaaaaaatatgtaataaaaagtgcttcaccaatgatttgggatgaatggaaggatggatggatggatataacattgcataccgttttcattttaaaaatgaatgttggttgc*ag*agattaataaacac**tgc**tctgatcgaaatgggagggaacca**tgc**ctc**tgt**ggg*gt*aaattataagattacaacttttatttacaaaataataaacaatatatgataggatattattaatatcttcattgaacattc*ag*gatggcaataatgta**tgt**ggtccatatgaatat**tgt**gaagtagacaaa**tgc**aaaa*gt*aatttttatgctataaaatttactgttaatttatgcaattgctattaatttattgttattatcaatttcattttatttaaattttatttttacataaataatattgtaatttattattactacaattattatcaacgaaagagtgatactttacaaacattgtattgaaatttattattaatactatattcttaatatttataataaaaatgaatgttggttgt*ag*aaatatac**tgt**tcggaaataaatgtctggtcat**gc**tac**tgt**atg*gt*aaattttaagatagtaacttttatttaccaactgttatcataaataacgtataatatcatcattaaacattc*ag*ggtgaaacaata**tgt**tatgaacatgaatat**tgt**gaacaaaatcctgaaggaaacaaa**tgc**atag*gt*aatttttataagttattaattttacacttttattaatttcattttttacataaataatatagtaatttattattaatactataatcctcaacgaaaaagttacacgtaatagattggcggatggatggatggatataacattacatacctcacttctttttatttaaaaaaatgaatgttttttgt*ag*cgaatcac**tgt**tctgatctaaatgtgcgacca**tgc**ctc**tgt**ggg*gt*aaattataagattgtaacttttatttatcaaatgttatcataaataacgtataatatcatcattgaacattc*ag*aacgaaagtcta**tgt**tatgaacaacattat**tgt**gacctgaattctgaaggaaacaaa**tgc**aaag*gt*aattattttgcaataaaagttagtattaattttacaattgttattaattttattttgttactaattttgttatgttattaaatttatttttattatcaatttcatttttttattaaattttcaaaaatagcaatggtatttattatactattgtgctcaacgctagagtgatatgtaataaaacgtgctacaaaactgatatgggattgatggatggatagatgggatggtttgtagatggatggatgcatttttgaattgatggatggatggatggatggatggacatagcattatataccgtgataaattcacttctgtttattttgaacatgaacgttggttgt*ag*agattaatattcac**tgc**tcggatcgaaatgggactgaacca**tgc**ctc**tgt**ggg*gt*aagttgtaatattacaacttttatttacaaaatattatcatacataaagtgtaatatgatatcattaatatcttcattgaacattc*ag*gatgactctaatgta**tgt**ggtcgacatgaagat**tgt**gaaggaaacata**tgc**atag*gt*aatttttatgtaataaaattttctattaacttcgtttttgttattaatttcgtttttgtttaaattttatttgtattcagataatattgtaatttattattaatactgttatgcccaacgaaatagtgataattaataaaaagttcctcaccaattatttagaatggatagatataacattacaaaccgtgataaattcacttctgcttataataaaaatgaatgttggttgt*ag*agatttataaactc**tgt**tctgaacataatgattatcca**tgc**ctc**tgt**ggg*gt*aagttataagattataactgttgttaatcaaatgttaaataatgtgtaatatcgtcattgaacattcatg*ag*gagcaagta**tgt**aaggaagatgaaatt**tgt**gaaacaaagataaacaaa**tgc**aaaa*gt*aattttcatataataacatttgtttttaatgcttttttattaattttatgtttgtagtactagtattttaatttattcttaatatttatgttaaaaatgaatgttggtttt*ag*aaatatac**tgt**tcggaaagaaatgtctggcca**tgc**tac**tgt**gtg*gt*aaattttaagatagtaacttttatttaccaactgttatcataaataacgtataatatcatcattgaacattc*ag*ggtgatactaag**tgt**gaaccacatgaaata**tgt**gaattgaattctgaaggaaacaaa**tgc**gtatttg*gt*aattttttacaatgaaatttaacattaattttacttattgttattaattttatttttattaacaatttcaattttatttacaatattaatattataatttattattaagtaattattaatattaatcttattatttatattaaacatgattgttggttgt*ag*aaaaa**tgc**cgggatagaaatgattatcct**tgc**ctc**tgt**gtg*gt*aaataaaaaataataaccttcagttttcaaatgttatcatgaataatgtataaaatcatcattgaactttc*ag*aatgactctgaacaa**tgt**ggtgaatatgaagag**tgt**gaaattcatgatgaaggaaacaaa**tgc**atag*gt*aatttttataagttattaattttatatatttattaatttcatttttttattaattttatttttacataaatattattgtaatttattattaatactattatcctcagcggaaaagtgataaataataaaaagtgcctcaccaatgatttgggaggggtggatggatggatggatggatgagtggatgtatgggtcgatggatagacggatataataacacttcatacctcacttctgtttattttaaaaatgaatgttttttgt*ag*cgtcttttgaacac**tgc**tctgatctaaatgtgcggcca**tgc**ctc**tgt**ggg*gt*aaattataagattgtaacttttatttatcaaatgttatcataaataacgtataatatcattgaacattc*ag*aacgaaaatcta**tgt**tatgaacaacattat**tgt**gacttgaattctgaaggaaacaaa**tgc**aaag*gt*aatttttttgcaataaaagttactattaattttgcaataggtgggatggtttttagatggatggatttgtggatgaatgcatttttgaattgatggatggatggatggacatagcattacataccgtgataaattcacgtgattaaattcacttctgtttatttttaacatgaacgttggttgt*ag*agattaacattcac**tgc**tcggatcgaaaggggaatgaacca**tgc**ctc**tgt**ggg*gt*aaattataagattacaacttttatttacaaaatgtaatcataaataacgtataatatcgtcattgaacattc*ag*gatgtgatc**tgt**gatgaagataaaact**tgt**gaaatgcattctctgggaaataaa**tgc**ataa*gt*aattttgttatattaaactttacaattaatatcacttcttttttttaatttttttttattttattgtattttttttaaataaaaatttattattaatattacgaccttaatctggtattaattaatagaggcaaacgatgtctaaagttgaaatttcttttatgactc*ag*aggtg**taa**

**atg**ttctcattgaagctatttgttgtcctcttggcttcttttggcatcggtttgactcaag

agattaataaacac**tgc**tctgatcgaaatgggagggaacca**tgc**ctc**tgt**ggg

gatggcaataatgta**tgt**ggtccatatgaatat**tgt**gaagtagacaaa**tgc**aaaa

aaatatac**tgt**tcggaaataaatgtctggtcat**gc**tac**tgt**atg

ggtgaaacaata**tgt**tatgaacatgaatat**tgt**gaacaaaatcctgaaggaaacaaa**tgc**atag

cgaatcac**tgt**tctgatctaaatgtgcgacca**tgc**ctc**tgt**ggg

aacgaaagtcta**tgt**tatgaacaacattat**tgt**gacctgaattctgaaggaaacaaa**tgc**aaag

agattaatattcac**tgc**tcggatcgaaatgggactgaacca**tgc**ctc**tgt**ggg

gatgactctaatgta**tgt**ggtcgacatgaagat**tgt**gaaggaaacata**tgc**atag

agatttataaactc**tgt**tctgaacataatgattatcca**tgc**ctc**tgt**ggg

gagcaagta**tgt**aaggaagatgaaatt**tgt**gaaacaaagataaacaaa**tgc**aaaa

aaatatac**tgt**tcggaaagaaatgtctggcca**tgc**tac**tgt**gtg

ggtgatactaag**tgt**gaaccacatgaaata**tgt**gaattgaattctgaaggaaacaaa**tgc**gtatttg

aaaaa**tgc**cgggatagaaatgattatcct**tgc**ctc**tgt**gtg

aatgactctgaacaa**tgt**ggtgaatatgaagag**tgt**gaaattcatgatgaaggaaacaaa**tgc**atag

cgtcttttgaacac**tgc**tctgatctaaatgtgcggcca**tgc**ctc**tgt**ggg

aacgaaaatcta**tgt**tatgaacaacattat**tgt**gacttgaattctgaaggaaacaaa**tgc**aaag

agattaacattcac**tgc**tcggatcgaaaggggaatgaacca**tgc**ctc**tgt**ggg

gatgtgatc**tgt**gatgaagataaaact**tgt**gaaatgcattctctgggaaataaa**tgc**ataa

aggtg**taa**

MFSLKLFVVLLASFGIGLT

QEINKH**C**SDRNGREP**C**L**C**GDGNNV**C**GPYEY**C**EVDK**C**K

KIY**C**SEINVWS**C**Y**C**MGETI**C**YEHEY**C**EQNPEGNK**C**I

ANH**C**SDLNVRP**C**L**C**GNESL**C**YEQHY**C**DLNSEGNK**C**K

EINIH**C**SDRNGTEP**C**L**C**GDDSNV**C**GRHED**C**EGNI**C**I

EIYKL**C**SEHNDYP**C**L**C**GEQV**C**KEDEI**C**ETKINK**C**K

KIY**C**SERNVWP**C**Y**C**VGDTK**C**EPHEI**C**ELNSEGNK**C**V

FEK**C**RDRNDYP**C**L**C**VNDSEQ**C**GEYEE**C**EIHDEGNK**C**I

ASFEH**C**SDLNVRP**C**L**C**GNENL**C**YEQHY**C**DLNSEGNK**C**K

EINIH**C**SDRKGNEP**C**L**C**GDVI**C**DEDKT**C**EMHSLGNK**C**IKV-

**Theoretical pI/Mw:** **4.70** / 37738.13

**Htia_mHV16 (this study)**

**chromosom 2 position 1971445 – 1972201 rev + comp**

**atg**tttcctctgatgttatttgttgttcttttggcttcttttggcatttgcttgtctgaagcaa*gt*gagtttgatatgagtttatcaatgatagaattatatgtaataaaaccagcttaaccagtaatgttggactgatagattgacggatggatggatggatagatggatgtaacattatatgcctcttctgtttacattgaaaatgaatattggttgt*ag*ctaaaaaa**tgt**tccaaaaataacatgactcct**tgc**cta**tgt**gag*gt*taactattaaactataacttttatttaccaagtattaatattaataacgtttgacatcatcgttgaacattc*ag*aaaaaggaaagcgaagta**tgt**cctgaaggtagcaaa**tgt**caactggattttgatggtggcaactta**tgc**tacgaaa*gt*aattatgcttataaagcaatttatatatagtaaaataatgcatttttattaacgctaatttatatattaaattatttcatttttactgttattactaactgctctagatctgttattaattaatttatgcatatcgtgtctgaatctgaaatttaatgaattgctatttattacttatttggaataaaactttttgaagaattattaatttttaaatttatttatctctgc*ag*gtatcagcggtagctca**tgc**aactctccgcatttaatcatcataata**tgctgt**ttgattcctcttgtatattttgatatattaaataaaatgttagtggtt**taa**

**atg**tttcctctgatgttatttgttgttcttttggcttcttttggcatttgcttgtctgaagcaa

ctaaaaaa**tgt**tccaaaaataacatgactcct**tgc**cta**tgt**gag

aaaaaggaaagcgaagta**tgt**cctgaaggtagcaaa**tgt**caactggattttgatggtggcaactta**tgc**tacgaaa

gtatcagcggtagctca**tgc**aactctccgcatttaatcatcataata**tgctgt**ttgattcctcttgtatattttgatatattaaataaaatgttagtggtt**taa**

MFPLMLFVVLLASFGICLSEATKK**C**SKNNMTP**C**L**C**EKKESEV**C**PEGSK**C**QLDFDGGNL**C**YESISGSS**C**NSPHLIIII**CC**LIPLVYFDILNKMLVV-

Theoretical pI/Mw: **5.19** / 8362.83

**Htia_HV6 (this study)**

**chromosom 2 position 1985268 – 1986029**

**Atg**tttcctctgatgttttttgttgttcttttggcatttgct**tga**ctgaatcaagtga*gt*ttgatatgagtttatcaatgatagaattatatgtaataaaaccagcttaaccattaatgttggactgatatattgacggatggatggatggatagatggatgtaacattatatgcctcttctgtttacattgaaaatgaatattggttgt*ag*ctaaaaaa**tgt**tccgaaaataacaagacccct**tgc**cta**tgt**gag*gt*taactattaaactataacttttatttaccaagtattaatattaataacgttttacatcatcattgaacattc*ag*aaaaaggaaagcgaagta**tgt**cctgaaggtagcaaa**tgt**caactggattttgatggtggcaactta**tgc**tacgaaa*gt*aattatgcttataaagcaatttatatatagtaaaataatacatttttattagcactattttatatattaaattatttaatttttactgttattactaactgttattaattaattgatgcatatcgtgtctgaatctgaaatttaatgaattgctacttattactactattattacttattaataaaaacttattaggaaaaaaactttgtgaagaattattaatttttaaatttatttatcactgc*ag*gtatcagcggtagctca**tgc**aactctccgcatttaatcatcataata**tgctgt**ttgattcctcttgtatactttgatatattaaataaaatgttagtggtt**taa**

**Atg**tttcctctg**atg**ttttttgttgttcttttggcatttgct**tga**ctgaatcaagtga

ctaaaaaa**tgt**tccgaaaataacaagacccct**tgc**cta**tgt**gag

aaaaaggaaagcgaagta**tgt**cctgaaggtagcaaa**tgt**caactggattttgatggtggcaactta**tgc**tacgaaa

gtatcagcggtagctca**tgc**aactctccgcatttaatcatcataata**tgctgt**ttgattcctcttgtatactttgatatattaaataaaatgttagtggtt**taa**

MFPLMFFVVLLAFA-LNQVTKK**C**SENNKTP**C**L**C**EKKESEV**C**PEGSK**C**QLDFDGGNL**C**YESISGSS**C**NS

PHLIIII**CC**LIPLVYFDILNKMLVV-

QVTKK**C**SENNKTP**C**L**C**EKKESEV**C**PEGSK**C**QLDFDGGNL**C**YESISGSS**C**NSPHLIIII**CC**LIPLVYFDILNKMLVV

Theoretical pI/Mw: **5.19** / 8387.83

**Htia_HV7 (this study)**
