# Supplementary material for: The great diversity: monomeric and oligomeric hirudins, hirudin-like factors and decorsins in the Asian medicinal leeches Hirudo nipponia and Hirudo tianjinensis
Source: Parasitol Res. 2026 Feb 7;125(1):18. doi: 10.1007/s00436-026-08634-0 (PMC12882960; doi:10.1007/s00436-026-08634-0)
Supplement: Supplementary file 1 — Supplementary Material 1 (ZIP 660 KB) [file 436_2026_8634_MOESM1_ESM.zip › S3_putative hirudin genes on chromosome 5 of Hirudo nipponia.docx]

Supplementary Information File S3: Localization of putative hirudin genes on chromosome 5 of *Hirudo nipponia*

**chromosome 5 position 15468609 - 15469291**

**atg**ttctctctgaaattgttcgtcgttctgttggcagtttgcatctgcacgtctcaagctc*gt*gagtttgactcgatcattactaaaatcggcaataaatgctagacagtagtgcatagacatttatttgctgtcggaattgatgtactggttttattgc*ag*agcatttcaaagat**tgc**tcagacagcaatccgactcca**tgc**ttg**tgc**gaa*gt*aagtatgagtggttattacacagatccatgtcttcttaaccactataaataattgttatgttatgttatgttatgttagttatttaaaattagggtttccgtcatttcaaataaggcaaatagttgcaacgttgaatttc*ag*aatagtaatctc**tgt**gcttttggtaacact**tgt**gatctgggcccaccaaagaaa**tgc**atcataaaag*gt*aacgatttctataatttataacatgaatattaaactataacaacattacatattacattaagctaaaaattactaacgcgatttattaatttctagaaaaaaaagcgctttccatacatatttgaataataactttttagtagcttacaaggagatttgtgaaacatttatatttaaattta*ag*tatcaccacctcccacctcggagaaagagaaaaataacaacaaaggaagtaaatctgattacgattattat**taa**

**atg**ttctctctgaaattgttcgtcgttctgttggcagtttgcatctgcacgtctcaagctc

agcatttcaaagat**tgc**tcagacagcaatccgactcca**tgc**ttg**tgc**gaa

aatagtaatctc**tgt**gcttttggtaacact**tgt**gatctgggcccaccaaagaaa**tgc**atcataaaag

tatcaccacctcccacctcggagaaagagaaaaataacaacaaaggaagtaaatctgattacgattattat**taa**

MFSLKLFVVLLAVCICTSQAQHFKD**C**SDSNPTP**C**L**C**ENSNL**C**AFGNT**C**DLGPPKK**C**IIKVSPPPTSEKEKNNNKGSKSDYDYY-

QHFKD**C**SDSNPTP**C**L**C**ENSNL**C**AFGNT**C**DLGPPKK**C**IIKVSPPPTSEKEKNNNKGSKSDYDYY

Theoretical pI/Mw: **6.72** / 6972.75

**hirudin_Hnip2 (Zhao et al. 2024)**

**Hnip_V3a** **(this study)**

**Hnip_HV2 (Müller et al. 2025)**

**Hnip hirudin (Lu et al. 2018)**

**chromosome 5 position 15402226 - 15402777**

**atg**tcctctctgaagctgcttgctgtctttttggttgtttgcatctccgtgtctcaagaacata*gt*ctgtacttggagaattatgagtattaattgattaattaattgaaatatatatttttttgcttatt*ag*tgtctggaaatttgattgct**tgt**aaaggagatcacgtaactgca**tgc**gtt**tgc**gag*gt*aaattcataatttattagtttattcataatttattaatttaaattttatttatgcatcaataatgctctgatgaatttt*ag*ggtaccgcaatt**tgc**ggtaagggcaaaaaa**tgc**atacttggctccactgtaaaggaaaataaa**tgt**gtcaaaa*gt*aattttattgacaatttatgatataaatattaaaatatcacacattgcattctattagacatttattatatgagtttctgaaatcacaattccgctgtgacctaattattattaattaataatagtaggcaactttaaaaataatgaagaattaattgctagctactaatacaaagattttaatctattatt*ag*atactggc**tga**

**atg**tcctctctgaagctgcttgctgtctttttggttgtttgcatctccgtgtctcaagaacata

tgtctggaaatttgattgct**tgt**aaaggagatcacgtaactgca**tgc**gtt**tgc**gag

ggtaccgcaatt**tgc**ggtaagggcaaaaaa**tgc**atacttggctccactgtaaaggaaaataaa**tgt**gtcaaaa

atactggc**tga**

MSSLKLLAVFLVVCISVSQEHMSGNLIA**C**KGDHVTA**C**V**C**EGTAI**C**GKGKK**C**ILGSTVKENK**C**VKNTG-

HMSGNLIA**C**KGDHVTA**C**V**C**EGTAI**C**GKGKK**C**ILGSTVKENK**C**VKNTG

Theoretical pI/Mw: **8.87** / 4808.68

**hirudin_Hnip1 (Zhao et al. 2024)**

**Hnip_V5 (Müller et al. 2022)**

**Hnip_HV1 (this study)**

**chromosome 5 position 15392527 - 15391957**

**atg**tccgtaaagatgtttgttgtctttttgactgtttgcatatccgtgtctcaggtag*gt*gattacaacctggtctttgtgataacaaacgttttaattttgatatattttacattttattttctatgtgaagtc*gg*gtccaatgttttttgaaaac**tgt**tcatcaactgttcaacgtaac**tgc**tta**tgc**aat*gt*gagtttataaattataatttcaacataccaaatgcttcaacaaacttgtaaacttcaacaaaattgttgaatttt*ag*ccaacaagtttt**tgc**agtagaggtacaaaa**tgc**atgttgtatgaagggattaagcaa**tgt**gttgacgaaa*gt*atttgattgtatttattgtttatgatgctaaaatttaaatatcatatttgcattacattgaattcaaatatattttcttattttccaaaatcaccatttgtcttaagacttaatcattattatttaataatagtgaacaacttaaaaagatttgtgagaaatattaatttattactagttattaatattaaattaattttactctcatgtt*ag*atcaaacctca**tag**

**atg**tccgtaaagatgtttgttgtctttttgactgtttgcatatccgtgtctcaggtag

gtccaatgttttttgaaaac**tgt**tcatcaactgttcaacgtaac**tgc**tta**tgc**aat

ccaacaagtttt**tgc**agtagaggtacaaaa**tgc**atgttgtatgaagggattaagcaa**tgt**gttgacgaaa

atcaaacctca**tag**

MSVKMFVVFLTVCISVSQVGPMFFEN**C**SSTVQRN**C**L**C**NPTSF**C**SRGTK**C**MLYEGIKQ**C**VDENQTS-

PMFFEN**C**SSTVQRN**C**L**C**NPTSF**C**SRGTK**C**MLYEGIKQ**C**VDENQTS

Theoretical pI/Mw: **6.48** / 5101.80

**hirudin_Hnip3 (Zhao et al. 2024)**

**Hnip_HV3 (this study)**
