# Supplementary material for: The great diversity: monomeric and oligomeric hirudins, hirudin-like factors and decorsins in the Asian medicinal leeches Hirudo nipponia and Hirudo tianjinensis
Source: Parasitol Res. 2026 Feb 7;125(1):18. doi: 10.1007/s00436-026-08634-0 (PMC12882960; doi:10.1007/s00436-026-08634-0)
Supplement: Supplementary file 1 — Supplementary Material 1 (ZIP 660 KB) [file 436_2026_8634_MOESM1_ESM.zip › S4_putative hirudin genes on chromosome 6 of Hirudo tianjinensis.docx]

Supplementary Information File S4: Localization of putative hirudin genes on chromosome 6 of *Hirudo tianjinensis*

**chromosom 6 position 15622291 - 15622887 rev+comp**

**atg**ttccccgtgaagatgcttgttgtctttttgactgtttgcatatccgtgtctcaggcaa*gt*gagtacaatctggtctttgtgatattaaatgtttaaattttaatatattttacaatcttttattttgtatgtga*ag*ttagttcacattatgtttttgaacac**tgt**tcttcaactcttcaacgtaat**tgt**ttt**tgc**aat*gt*gagtttatgaattacattttcaacataccaaatgcttcaacaaacattgttgaatttt*ag*ggaaaacatttt**tgc**ggtacaggtacaaaa**tgc**atgttgtataacgggattacacaa**tgt**gttgacgaaa*gt*gattttatttactatttatgatgctaaaatttaaatatcatacttgcattatattaaattcaaatttattttcttattttccaaaatcaccatttgtcttatgacttaatcattattatttaataatagtgaacaacttaaaaggattatatgagaaatattaattagttatattaattataatttatattaattaattatattaattactagttactaatattaaattaattttactttcatgtt*ag*ataagacctca**tag**

**atg**ttccccgtgaagatgcttgttgtctttttgactgtttgcatatccgtgtctcaggcaa

ttagttcacattatgtttttgaacac**tgt**tcttcaactcttcaacgtaat**tgt**ttt**tgc**aat

ggaaaacatttt**tgc**ggtacaggtacaaaa**tgc**atgttgtataacgggattacacaa**tgt**gttgacgaaa

ataagacctca**tag**

MFPVKMLVVFLTVCISVSQAISSHYVFEH**C**SSTLQRN**C**F**C**NGKHF**C**GTGTK**C**MLYNGITQ**C**VDENKTS-

ISSHYVFEH**C**SSTLQRN**C**F**C**NGKHF**C**GTGTK**C**MLYNGITQ**C**VDENKTS

Theoretical pI/Mw: **7.79** / 5394.08

**hirudin_** **Htia3 (Zhang et al. 2024)**

**Htia_HV3 (this study)**

**chromosom 6 position 15633162 - 15634521**

**atg**ttctctctgaagctgtttcttgtcctcttggctgtttgcatatgcgtgtctcaagcaa*gt*gggtgtagcctggtctttatgagaattaaaattattaactacaattccatgtatatttgttgttgttttgtgtgtgttttagttgatgaattcgttatcttttt*ag*atcgttactctgtc**tgt**actgaaactggtcaaaacctt**tgc**ctt**tgc**gag*gt*aagttaataaattatgctgttaataaattgtgttgttattaaattatgttgtcaataagttatgctgttaagttaagaagttatgttgtggctacaacaacaaccgttgaatttt*ag*ggaagtgatctt**tgc**tctctcgataaccat**tgc**gaaataggctctaatggaaagaataga**tgt**gtcaaaggag*gt*aattattaataaattaagaaattatatattataataaattataaattatatattataataaattataaattataataaattataaattatatattataataaattataaattatatatattaataaattatataaatctatatatatatatatacatatatatatataaaacaaggatcttgcattaatattttttacacaaatctttaatcttgaaccgatttcattgaaacttgaaacacttgactacgttgactatggggtcagtttggtctattttgcaaaaaaatcgataaattattttgcattttattgccaaaaacctcttctgggcttattctctcaatagcgtacgcgccgttttcgaagctggtgagcttaggcgcatgctgattaatattcatgtacgtaaatttttagtttatattgtaaactttccgttttataatttaaaaatttacatttttttattatttatctatgatttaatattagtgttattttggtagccatcttattccgtttgtcattcgtaagttatagtgttgtattcgtcggggcagcctagaaaatactgtttattgtgttaaatcatgttaataaaaaatttttataccacaataatgaatttaaaaaaaaaatgttttaatattttctgaaatactttgtcttacgataataaataatttaaaaattaaatggcagaacattacttgatattagttattaatatatataatataatatatatatatatattatatcattaaaatagataaaatataaaaatagatatataatatatattaataacatatatattagttattattattatattagttcaaatttttatctc*ag*aaggaaaaccaaagaaacctcaaagcaattctgacttgtctgaggagaagtatgaaccaatcccaattgaagactacgataaa**tga**

**atg**ttctctctgaagctgtttcttgtcctcttggctgtttgcatatgcgtgtctcaagcaa

atcgttactctgtc**tgt**actgaaactggtcaaaacctt**tgc**ctt**tgc**gag

ggaagtgatctt**tgc**tctctcgataaccat**tgc**gaaataggctctaatggaaagaataga**tgt**gtcaaaggag

aaggaaaaccaaagaaacctcaaagcaattctgacttgtctgaggagaagtatgaaccaatcccaattgaagactacgataaa**tga**

MFSLKLFLVLLAVCICVSQANRYSV**C**TETGQNL**C**L**C**EGSDL**C**SLDNH**C**EIGSNGKNR**C**VKGEGKPKKPQSNSDLSEEKYEPIPIEDYDK-

NRYSV**C**TETGQNL**C**L**C**EGSDL**C**SLDNH**C**EIGSNGKNR**C**VKGEGKPKKPQSNSDLSEEKYEPIPIEDYDK

Theoretical pI/Mw: **4.84** / 7668.44

**hirudin_Htia1 (Zhang et al. 2024)**

**Wpig_V5 (Müller et al. 2022)**

**Htia_HV1 (this study)**

**Hirudin-HN (Cheng et al. 2019)**

**chromosom 6 position 15636955 - 15637712**

**atg**ttctctttgaagctgtttcttgtctttttggttgtttgcatctccgtgtctcaagaaaaca*gt*ctgtacttggagagaattatgagtattaattaattaattaattaattgaaatatatattattctttttttgcttatt*ag*aagcagaactgttggctgct**tgt**tcaggaaaacgtgtaactctc**tgc**gct**tgc**aat*gt*aaattaataatttattcataatatattaatttatttataatttattaataatttattaacttattcaaagcttattaatttatttacaatttaatcatttatttatataccaataatgctttggtgaatttt*ag*ggtgtcgaattt**tgc**ggtagaggtaaaaaa**tgc**aaatttggctccactccagcagaaaataaa**tgt**gtcaaag*gt*aattttatcgacaatttatgatatatatatatatatatcatatatatgtatatatatatatattaaaatatcaaacattacattctattagacatttattacattagtttctgaaatcaccactccgctgtgacctaattataatattattaattaataaaagaacacaactttacaaattatgaagaattaattaatagttacaattagttacaattgattacaattagttacaattagttacaattgattacaattagttacaattagttacaattgattacaattagttacaattagttactagtacaaagattttaatctattatc*ag*atgattcccca**taa**

**atg**ttctctttgaagctgtttcttgtctttttggttgtttgcatctccgtgtctcaagaaaaca

AAGCAGaactgttggctgct**tgt**tcaggaaaacgtgtaactctc**tgc**gct**tgc**aat

ggtgtcgaattt**tgc**ggtagaggtaaaaaa**tgc**aaatttggctccactccagcagaaaataaa**tgt**gtcaaag

atgattcccca**taa**

MFSLKLFLVFLVVCISVSQENKAELLAA**C**SGKRVTL**C**A**C**NGVEF**C**GRGKK**C**KFGSTPAENK**C**VKDDSP-

QENKAELLAA**C**SGKRVTL**C**A**C**NGVEF**C**GRGKK**C**KFGSTPAENK**C**VKDDSP

Theoretical pI/Mw: **8.63** / 5293.08

**Htia_HV4 (this study)**

**chromosom 6 position 15703335 - 15703985**

**atg**ttttctttgaaagtgttcgtcgtcttgttggcagtttgcctctgcatgtctcaagctc*gt*gagtttgactcgatctttactaaaattggcaataaatgctagacagtagttttatttactgtcgtagttgatgtactggttttattgc*ag*agcgtttcaaagaa**tgc**tcaaggagtaatccgactcca**tgc**ttg**tgc**gaa*gt*aagtatgagtggttattacacagatccaggtcttcttaaccactataaataattgttatgttatgttaattatttaaaattacggtttccgtcatttcaaataaggcaaatagttgaaacgttgaatttc*ag*aatggtaatctc**tgt**acttctggtaacact**tgt**gatctgggcccgccaaagaaa**tgc**atcgtaaaac*gt*aatgatttctataatttataacatgaatattaaactataacaatattacatattacaataagctaaaaattactatttactattacgatttagtaatttctagaaaaaagcgctttccatgcatatttgaataataacattttagtagcttacaaagagatttgtgaaacatttatttttaaattta*ag*aatcttccatctcggagaataaagaaagcaagtctgattacgatgagtatgat**taa**

**atg**ttttctttgaaagtgttcgtcgtcttgttggcagtttgcctctgcatgtctcaagctc

agcgtttcaaagaa**tgc**tcaaggagtaatccgactcca**tgc**ttg**tgc**gaa

aatggtaatctc**tgt**acttctggtaacact**tgt**gatctgggcccgccaaagaaa**tgc**atcgtaaaac

aatcttccatctcggagaataaagaaagcaagtctgattacgatgagtatgat**taa**

MFSLKVFVVLLAVCLCMSQAQRFKE**C**SRSNPTP**C**L**C**ENGNL**C**TSGNT**C**DLGPPKK**C**IVKQSSISENKESKSDYDEYD-

QRFKE**C**SRSNPTP**C**L**C**ENGNL**C**TSGNT**C**DLGPPKK**C**IVKQSSISENKESKSDYDEYD

Theoretical pI/Mw: **5.22** / 6349.00

**hirudin_** **Htia2 (Zhang et al. 2024)**

**Wpig_V4a (Müller et al. 2022)**

**Htia_HV2 (this study)**

**HLF-HN (Fan et al. 2021)**
