# Supplementary material for: The great diversity: monomeric and oligomeric hirudins, hirudin-like factors and decorsins in the Asian medicinal leeches Hirudo nipponia and Hirudo tianjinensis
Source: Parasitol Res. 2026 Feb 7;125(1):18. doi: 10.1007/s00436-026-08634-0 (PMC12882960; doi:10.1007/s00436-026-08634-0)
Supplement: Supplementary file 1 — Supplementary Material 1 (ZIP 660 KB) [file 436_2026_8634_MOESM1_ESM.zip › S5_putative ornatin genes on chromosome 5 of Hirudo nipponia.docx]

Supplementary Information File S5: Localization of putative ornatin genes on chromosome 5 *Hirudo nipponia*

**chromosome 5 position 15256672 - 15257744**

**atg**agagctttgataatttgccttgttttgggtctggtcatcgcaagtggagtttctcctactggtttaaaaccattttgatttattgattcaaataatttctatatatccacattttttgagtttcttttacttttaaatttatacaatcatttcgaatctaattttgcaagtgaatttgaattacagattatactaaataaatttcctaagataagatgataagatgaccatttgatattctttgtagtttattttagggttttcactaaatagttgatatttaaaatatattaatagatcaatgataacaaacacagtaattacactgaacaaatataacgacttatatcggatatcttgatatccaataacatttcttaatcaatttgaacatgctaattttgaatatgcaaactatttgtctctgtcataatgtgtgtacaatatatgttttaagttgctaaaagtaatactttagcaaataaaatcaattgtttgacaatttcttaaatatttgacctgagagcaaaacgcttttcttatgaataactttttgacgtagaagatttcttcatcctgtttgacggtgtaatttatgataaaaaataatttttaaattgaaatatgcatgaagcatgaagcttattattatgttacaac*ag*gtgcgcaagtt**tgt**tcaggttataatgacaaaccggac**tgc**agg**tgc**aat*gt*aagattcctaattccattaattagcatttcctattgaataacatcttctaaaatggtattgctattaataaaattaatttttgtattaataatttcaaaatccaattaatgttcaatatatttagcttgtatattgatttacactaaaatgttaggcatagtatttgcatttttatcttaagaattacaaccagacaatattttattgtacaatttccatgaacgagtcaaattaattacaactgaaacttttaccgcatctacatttttgtaatttgttttatc*ag*ggtaaacca**tgc**gatcccggtcaaagt**tgc**caatttccg**agaggagat**gcagatccctac**tgc**gca**tga**

**atg**agagctttgataatttgccttgttttgggtctggtcatcgcaagtggagtttctcctactg

gtgcgcaagtt**tgt**tcaggttataatgacaaaccggac**tgc**agg**tgc**aat

ggtaaacca**tgc**gatcccggtcaaagt**tgc**caatttccg**agaggagat**gcagatccctac**tgc**gca**tga**

MRALIICLVLGLVIASGVSPTGAQV**C**SGYNDKPD**C**R**C**NGKP**C**DPGQS**C**QFP**RGD**ADPY**C**A-

AQV**C**SGYNDKPD**C**R**C**NGKP**C**DPGQS**C**QFP**RGD**ADPY**C**A

Theoretical pI/Mw: **4.69** / 4067.46

**decorsin_Hnip1 (Zhao et al. 2024)**

**Hnip_DV1 (this study)**

**chromosome 5 position 15259520 - 15260585**

**atg**agagctttgataatttgccttgttttgggtctggtcatcgcaagtggagtttctcctactg*gt*ttaaaaccatttcgatttattgattcaaataatttttatatatctaaattttttaagtttgtgttaattttaaatttatacaatcatttcaaatctaattttgcaagtgaatttgaatttcattcttgattattctaaataaatttcctaagataagttgaagggatatgtctacatgtccacatgatattctttgtagctcatgagattttaagcttttcactaaatagttcatatttaaattttattaatagatcaatggtaataaacactataattacactgaacaaaactaacgacttatctcggatatcttgatttccaatgccagttcttaatcaatttgaacatgctaattttgaatatgtaaactatttgtctctgtctcttgaatatgtgttcaatatatgttttaagttgctaaaagtaatattttagcaaataaaaacgtttgtttgacaatttcataaatatttgacctgagagcaaaacgcttttcttaaaaataactttttgacatagaaaatatcttcatcctgttttacgatgtgatttatgattaaaagtaatttttcaatgggaatatgcatgaaacatgaaacttattattatgttac*ag*caggtgcgaaaatt**tgt**agaggtagtggtgacgaaccggac**tgc**ata**tgt**aat*gt*aagactcttatttccattaattagcatttcttattgaataacatttgctaaaatggtattgctatcaaaatccaagtaatgttcaatatatttagcttgtgttttgatttacactgcaatgtttacgcataatatttgtatttttatcttaacaattacaatcagacatattttattgtacgatttccatgaacgagtcaaattaattacagttgaaactttttctgcatctaaacttatgttgttgtttttgtc*ag*aaacaacca**tgc**aatcccggtcaaagt**tgc**caatttccg**agaggagat**gcagatccctac**tgc**gca**tga**

**atg**agagctttgataatttgccttgttttgggtctggtcatcgcaagtggagtttctcctactg

caggtgcgaaaatt**tgt**agaggtagtggtgacgaaccggac**tgc**ata**tgt**aat

aaacaacca**tgc**aatcccggtcaaagt**tgc**caatttccg**agaggagat**gcagatccctac**tgc**gca**tga**

MRALIICLVLGLVIASGVSPTAGAKI**C**RGSGDEPD**C**I**C**NKQP**C**NPGQS**C**QFP**RGD**ADPY**C**A-

AKI**C**RGSGDEPD**C**I**C**NKQP**C**NPGQS**C**QFP**RGD**ADPY**C**A

Theoretical pI/Mw: **4.78** / 4045.50

**decorsin_Hnip2 (Zhao et al. 2024)**

**Hnip_DV2 (this study)**

**chromosome 5 position 15262002 -** **15263038**

**atg**agagctttgataatttgccttgttttgggtctggtcatcgcaagtggagtttctcctactg*gt*ttaaaaccatttcgatttattgagtcaaataatttctatatatctgcattgttgagtttctgttaattttaaatttatacaatcatttcaaatcaaattttgcaagtgaatttgaattacagattattctaaataaatttcctaagataagatgaagggatagccctacatgaccatttgatattctttgtagctcatgagattttagggtttttactaaatagttcatatttaaaatatattaatagatcaatgataacaaacactgtaattacactgaacaaatataacgacttatatcggatatccaataacatttcttaatcaatttgaacatgctaattttgaatatgcaaactatttgtctctgtcataatgtgtgtacaatatatgttttaagttgctaaaagtaatattttagcaaataaaaacatttgtttgacaatttcttaaatatttgacctgagagcaaaacgcttttcttatgaataactttttgacgtagaagatttcttcatcttgtttgacggtgtgatttatgataaaaaataatttttaaatggaaatatgcatgaagcatgaagcttattattatgttac*ag*caggtgcgaaagtt**tgt**tcaggttataatgacgaaccggac**tgc**agg**tgt**aat*gt*aagattcctaattccattatattgaataacatcttctaaaatggtattgctattaaaatccaagtaatgttcaatatatttagcttgtatattgatttacactaaaatgttaggcatagtaattgcatttttatcttaagaattacaaccagacaatattttattgtacgatttccatgaacgagtcaaattaattacaaatgaaacttttaccgtatctacatttttgtaatttgttttatc*ag*ggtaaacca**tgc**gatcccggtcaaagt**tgc**caatttccg**agaggagat**gcagatccctac**tgc**gca**tga**

**atg**agagctttgataatttgccttgttttgggtctggtcatcgcaagtggagtttctcctactg

caggtgcgaaagtt**tgt**tcaggttataatgacgaaccggac**tgc**agg**tgt**aat

ggtaaacca**tgc**gatcccggtcaaagt**tgc**caatttccg**agaggagat**gcagatccctac**tgc**gca**tga**

MRALIICLVLGLVIASGVSPTAGAKV**C**SGYNDEPD**C**R**C**NGKP**C**DPGQS**C**QFP**RGD**ADPY**C**A-

AKV**C**SGYNDEPD**C**R**C**NGKP**C**DPGQS**C**QFP**RGD**ADPY**C**A

Theoretical pI/Mw: **4.46** / 4068.44

**decorsin_Hnip3 (Zhao et al. 2024)**

**Hnip_DV3 (this study)**

**chromosome 5 position 15265547 - 15268223**

**atg**aaacaactgataatatgttttttcctgtcgttggcatttgcaaacg*gt*gagacatattattttaagtagtatgatcctttatttaaatggcttccttcaaatatttcaaattttctagagttctttgaaagtaatagtttttaatttgcaagtgattcgcatcaagtggcgtgcaagttagctcagagggtagcgtgcctgcgaacgacggtaaaagcaacagtcgttacgtgtcgtgttcgaaacccaacttgcactgccacaaagcgccccttggaattaaaattgaaattaaatatttaaatgc*AG*ACAAATTAGAAGAT**TGT**CGCTTGGTGGATAAAGATTTCAGAGGACAACCCAGAAGTGCC**TGC**AAG**TGT**GGG*GT*aataacgaattaaattaatattaatagtttaaagtttatcaacatatatgcacattaagcaatatttgttattatcgttttaaaaatttatttcttaattttaaagtttttgaaaggtgataaattaaaaactcatttgtgagtttaaggtgatcatgaaagcatcattttcttc*ag*gaaagacag**tgc**aaaaagttcgaagaa**tgc**gtgttttca**gatggaaat**gaacctgatctt**tgc**cggagaa*gt*aacaat**taa**ttacttcgttttgataatctttatttttaaatttagaacaatacaatcgaagtattcaaaatataaaaaattggattttattaaagagcaaatttttattagattgtgttagattgagtgttaagataatcatttccttatccaggtaaaaatacatagtttttttttatttatccc*ag*ctaaacccgat**tgc**atcggtaactcggatgatgaagagtgggga**tgt**gtt**tgt**gga*gt*aagcaaaattataattattttattttttaaaatattcattattcattattcattattagcaatatagctaaatagcgcagttactgctaacatgaatatctaactactgttatgaaacttaaaatttaaattctggaaaaattgaaaaaacccttttgaagttcttctgcatttc*ag*gaaaggcag**tgc**gaaccgaatgaaagc**tgt**aagaaagttgacaacaaattggaa**tgt**atcgaaa*gt*gagcctcattatgttgtttatctaatatgaacaggccgccaagaggggtggtgatgatgatgatgatgacgatgatgatgatgatgatgatgatgatgatgatgatgatgatgatgatgatgatgatgatgatgatgatgatgatgatgatgatgatgatgatgatgatgatgatgatgatgatgatgatgatgatgatgatgatgatgatgatgatgatgatgatgatgatgatgatgatgatgatgatgatgaagatgaagatgatgattatgatgacgatgatgacgattaaagatcaaacaaggggcctggctttagaataattcctttttttgcggacttaggtttgcagaaaatcgagatattattattcaatgtgaaataatataacgaaagctggtcactaaaaaatatatttcttcatatatatatatatgaatatatatgaataatatatattcttcactttattactgctaatatttctagatctaattttttagcagcaagcaagtaaggccaatcaataaactcacaaataaaatcttccatttactatggcgtttagtgataaaattattttgatttaaataatcaaattatttaaaataattatttcattatcaatgagaaactgaagaattattaataagatatattaatagatacaaataacattattaacttc*ag*aacctgtgcct**tgc**gagaaggcagaaagggacaaattcaaccgaccagttaatcct**tgc**act**tgt**agttat*gt*cagtcctcccttattttatttaattcttaattcaattgacacttgttaatttcgtttaatttttaagttactttgtactagttaattttattaccgaaaggcgctcgtagattaaatttgtccatttatttcattgttcattgcatttgaaatttgaaaatatttgtttaaaacattatgcctatgaattcatgttattatttac*ag*gggaagcca**tgc**aagaagcacgaatat**tgt**ctgattaaccgt**cgtggagac**aacatcgactat**tgt**atttcaa*gt*aagctagtgtcc**tag**taaattatttattaaccattttcgccaaaattcacgtgattaatctccgcattgattcatgttaaaactttgtcgat*ag*agtacccaaaa**tgc**aagtggccgattgaaaaggatacaaaatct**tgt**ata**tgc**ggc*gt*tagtatttacgacaacatgctgcattggaatcatgaatacaattttagttttgacaggcgattgattacactgtgcataatcacagtttaataaataactcttatttcattcaaacgaa*ag*ggcaaagta**tgt**cgcaaaggaaaggtt**tgc**aaatggataaaaggcaaagaaaaa**tgc**ttaaaag*gt*tattgaattgttttattacaaatcagcattcataaatcaaaaaataaattaaaattaatgaaatattaaaaattcttatttatta*ag*atgaagatgagaataaaaattcgataaaaaccaagaaaccaaagaacggtcgtactcttcgtctgggtcgagttgat**taa**

**atg**aaacaactgataatatgttttttcctgtcgttggcatttgcaaacg

ACAAATTAGAAGAT**TGT**CGCTTGGTGGATAAAGATTTCAGAGGACAACCCAGAAGTGCC**TGC**AAG**TGT**GGG

gaaagacag**tgc**aaaaagttcgaagaa**tgc**gtgttttca**gatggaaat**gaacctgatctt**tgc**cggagaa

ctaaacccgat**tgc**atcggtaactcggatgatgaagagtgggga**tgt**gtt**tgt**gga

gaaaggcag**tgc**gaaccgaatgaaagc**tgt**aagaaagttgacaacaaattggaa**tgt**atcgaaa

aacctgtgcct**tgc**gagaaggcagaaagggacaaattcaaccgaccagttaatcct**tgc**act**tgt**agttat

gggaagcca**tgc**aagaagcacgaatat**tgt**ctgattaaccgt**cgtggagac**aacatcgactat**tgt**atttcaa

agtacccaaaa**tgc**aagtggccgattgaaaaggatacaaaatct**tgt**ata**tgc**ggc

ggcaaagta**tgt**cgcaaaggaaaggtt**tgc**aaatggataaaaggcaaagaaaaa**tgc**ttaaaag

atgaagatgagaataaaaattcgataaaaaccaagaaaccaaagaacggtcgtactcttcgtctgggtcgagttgat**taa**

**long variant:**

**M**KQLIICFFLSLAFANDKLED**C**RLVDKDFRGQPRSA**C**K**C**GERQ**C**KKFEE**C**VFS**DGN**EPDL**C**RRTKPD**C**IGNSDDEEWG**C**V**C**GERQ**C**EPNES**C**KKVDNKLE**C**IEKPVP**C**EKAERDKFNRPVNP**C**T**C**SYGKP**C**KKHEY**C**LINR**RGD**NIDY**C**ISKYPK**C**KWPIEKDTKS**C**I**C**GGKV**C**RKGKV**C**KWIKGKEK**C**LKDEDENKNSIKTKKPKNGRTLRLGRVD-

NDKLED**C**RLVDKDFRGQPRSA**C**K**C**GERQ**C**KKFEE**C**VFS**DGN**EPDL**C**RR

TKPD**C**IGNSDDEEWG**C**V**C**GERQ**C**EPNES**C**KKVDNKLE**C**IE

KPVP**C**EKAERDKFNRPVNP**C**T**C**SYGKP**C**KKHEY**C**LINR**RGD**NIDY**C**IS

KYPK**C**KWPIEKDTKS**C**I**C**GGKV**C**RKGKV**C**KWIKGKEK**C**LK

DEDENKNSIKTKKPKNGRTLRLGRVD

Theoretical pI/Mw: **8.78** / 23290.77

**Hnip_DV4 (this study)**

**short variant:**

**atg**aaacaactgataatatgttttttcctgtcgttggcatttgcaaacg

aacctgtgcct**tgc**gagaaggcagaaagggacaaattcaaccgaccagttaatcct**tgc**act**tgt**agttat

gggaagcca**tgc**aagaagcacgaatat**tgt**ctgattaaccgt**cgtggagac**aacatcgactat**tgt**atttcaa*gt*aagctagtgtcc**tag**

**M**KQLIICFFLSLAFANEPVP**C**EKAERDKFNRPVNP**C**T**C**SYGKP**C**KKHEY**C**LINR**RGD**NIDY**C**ISSKLVS-

NEPVP**C**EKAERDKFNRPVNP**C**T**C**SYGKP**C**KKHEY**C**LINR**RGD**NIDY**C**ISSKLVS

Theoretical pI/Mw: **8.61** / 6220.11

**Hnip_DV4k (this study)**
