# Supplementary material for: The great diversity: monomeric and oligomeric hirudins, hirudin-like factors and decorsins in the Asian medicinal leeches Hirudo nipponia and Hirudo tianjinensis
Source: Parasitol Res. 2026 Feb 7;125(1):18. doi: 10.1007/s00436-026-08634-0 (PMC12882960; doi:10.1007/s00436-026-08634-0)
Supplement: Supplementary file 1 — Supplementary Material 1 (ZIP 660 KB) [file 436_2026_8634_MOESM1_ESM.zip › S6_putative ornatin genes on chromosome 6 of Hirudo tianjinensis.docx]

Supplementary Information File S6: Localization of putative ornatin genes on chromosome 6 of *Hirudo* *tianjinensis*

**chromosom 6 position 15046240 - 15047295 rev+comp**

**atg**agagctttgacaatttgccttgtcttgtctttggtcatcgcaagtattgtttctcctacag*gt*ttaaaatattttcgatttattgattaaaataagtttttatgtatctatattttttgggtttccgttaaatttaagtttatgcaaatttttcaaaatccaattttgcaagtgaatttgaattacagattattctaaataaattccctaagataagatgaagtgttatgtctacatgtccagatgatattctttgtagctcatcagattttaggcctttcactaaatagttcttatttaaattttattaatagatcaatgataataaacactataattacactgaacaaaaacaacgacttatctcgggtatcttgatatccaataatagttcttagtcagtttaaacatgttaattttgaatatgtaaactatttgactctatcataatatgtgtacaacatatattttaatttgctaaaaggcaattttatcaattgaaaacctttgtttgaaaatttctgaaatatttgacctgagagcaaaacgcttttcttaagaaaatcttaatgacgtataaaatatcttcatcctattttagggtgtgatttatgattaaaaataatttttaaatggatatatgcacgaaacttattattatgttac*ag*caggtgcgaaagct**tgt**acaggtagaaattttgatggtgctgacgaaccggac**tgt**ata**tgt**cat*gt*aagacttctaatttcatttattaccattttcaagtaatgttcaacatatttaaataaagtttgattacatttaaattaaatttagcttgtatatcgattgacactaaaatgtttggcataatatttgtattttcatcttaacaactacaatcagttatattttattgtacgatttccatcaactagccaaattaactacatttaaagctttttctaatttatgttattatgttttatc*ag*catggaccg**tgc**aaacccggtgagata**tgc**agtgtttct**agaggagat**aacaggaaatat**tgc**gagaaacagaat**tag**

**atg**agagctttgacaatttgccttgtcttgtctttggtcatcgcaagtattgtttctcctacag

CAGgtgcgaaagct**tgt**acaggtagaaattttgatggtgctgacgaaccggac**tgt**ata**tgt**cat

catggaccg**tgc**aaacccggtgagata**tgc**agtgtttct**agaggagat**aacaggaaatat**tgc**gagaaacagaat**tag**

MRALTICLVLSLVIASIVSPTAGAKA**C**TGRNFDGADEPD**C**I**C**HHGP**C**KPGEI**C**SVS**RGD**NRKY**C**EKQN-

AKA**C**TGRNFDGADEPD**C**I**C**HHGP**C**KPGEI**C**SVS**RGD**NRKY**C**EKQN

Theoretical pI/Mw: **6.91** / 4911.44

**decorsin_Htia5 (Zhao et al. 2024)**

**Htia_DV5 (this study)**

**chromosom 6 position 15354719 - 15355774**

**atg**agagctttgataatttgccttgtcttgtctttggtcatcgcaagtggaatttctcctacag*gt*ttaaaataattttgatttattcatttaaaaagtttttatgtatctatattttttgggtttctgttaaatttaaatttatgcaaatttttccaaatccaattttgcaagtgaatttgaattagagattattctaaataaattccctaagataagatgaagggatatgtctacatgtccacatgatattctttgtagttcatcagatttttggcctttcactcaatagttcttatttaaattttattaatagatcaatgataataaacactataattatactgaaccaaaaataacgacttatctcgggtatcttgatatccgataacaagtaaattagtaaatttgaacatgttaattttgaatatgtaaactatttctctatcataatatgtgtacaatatatattttaaattgctaaaaggcaattttatcaattgaaaacctttgtttaaaaatttctgaaatatttgacctgagagcaaaacgctattcttaagaaaaacttaatgacgtataaaatatcttcatcctattttagggtgtgatttatgattaaaaataatttttaaatggatatatgcacgaaacttattattatgttac*ag*caggtgcgaaagct**tgt**acaggtagaaattttgatggtgctgacgaacccgac**tgt**ata**tgt**cat*gt*aagacttctaatttcatttattaccattttcaagtaatgttcaacatatttaaattaagtttgattacatttaaattaaatttagcttgtatatcgattgacactaaaatgtttggcataatatttgtattttcatcttaacaactacaataagctatattttattgtacgatttccatcaactaaccaaattaattacatttaaagcattttctaatttatgttattttgttttatc*ag*catggaccg**tgc**aaacccggtgagata**tgc**agtgtttct**agaggagat**aacaggaactat**tgc**gagaaacagaat**tag**

**atg**agagctttgataatttgccttgtcttgtctttggtcatcgcaagtggaatttctcctacag

caggtgcgaaagct**tgt**acaggtagaaattttgatggtgctgacgaacccgac**tgt**ata**tgt**cat

catggaccg**tgc**aaacccggtgagata**tgc**agtgtttct**agaggagat**aacaggaactat**tgc**gagaaacagaat**tag**

MRALIICLVLSLVIASGISPTAGAKA**C**TGRNFDGADEPD**C**I**C**HHGP**C**KPGEI**C**SVS**RGD**NRNY**C**EKQN-

AKA**C**TGRNFDGADEPD**C**I**C**HHGP**C**KPGEI**C**SVS**RGD**NRNY**C**EKQN

Theoretical pI/Mw: **6.05** / 4897.37

**decorsin_Htia4 (Zhao et al. 2024)**

**Htia_DV4 (Müller et al. 2025)**

**chromosom 6 position 15358633 - 15359719**

**atg**agagctttgataatttgccttgtcttgtctttggtcatcgcaagtggaatttctcctacag*gt*ttaaaataattttgatttattcatttaaaaagtttttatgtatctatattttttgggtttctgttaaatttaaatttatgcaaatttttccaaatccaattttgcaagtgaatttgaattagagattattctaaataaattccctaagataagatgaagggatatgtctacatgtccacatgatattctttgtagttcatcagatttttggcctttcactcaatagttcttatttaaattttattaatagatcaatgataataaacactataattatactgaaccaaaaataacgacttatctcgggtatcttgatatccgataacaagtaaattagtaaatttgaacatgttaattttgaatatgtaaactatttctctatcataatatgtgtacaatatatattttaaattgctaaaaggcaattttatcaattgaaaacctttgtttaaaaatttctgaaatatttgacctgagagcaaaacgctattcttaagaaaaacttaatgacgtataaaatatcttcatcctattttagggtgtgatttatgattaacaatcatttttaaatggatatatgtatgaaacatgaaacgtattattatgttac*AG*CAGGTGCAACATAT**TGC**AAAGGTAGTGGTGACAAACAGGAC**TGC**ATA**TGT**AAT*GT*aagactcctaattccattaattagtatttcctattgaataacatattctaaaatagtattgcttttaataatattaatttttattaataatttcaaaatccaagtaatgttcaatatattttgcttgtatattgatttacactaaaatgttaggcataatgttggtatttttatcttattaattaacaattacaatcagacatatttaattgtaagattttcatgaacgagtcaaatttattacatttaaaacttttctgcatatacattgatgttttttaac*ag*aaaaaacca**tgc**gatcccggtcaaagg**tgc**caatttccg**agaggagat**gcagatccctac**tgc**gca**tga**

**atg**agagctttgataatttgccttgtcttgtctttggtcatcgcaagtggaatttctcctacag

CAGGTGCAACATAT**TGC**AAAGGTAGTGGTGACAAACAGGAC**TGC**ATA**TGT**AAT

aaaaaacca**tgc**gatcccggtcaaagg**tgc**caatttccg**agaggagat**gcagatccctac**tgc**gca**tga**

MRALIICLVLSLVIASGISPTAGATY**C**KGSGDKQD**C**I**C**NKKP**C**DPGQR**C**QFP**RGD**ADPY**C**A-

ATY**C**KGSGDKQD**C**I**C**NKKP**C**DPGQR**C**QFP**RGD**ADPY**C**A

Theoretical pI/Mw: **7.79** / 4140.64

**decorsin_Htia3 (Zhao et al. 2024)**

**Htia_DV3 (this study)**

**chromosom 6 position 15379193 - 15383000 rev+comp**

tgtttacgtttgacttacagaaatttaaagaattagagatatcttacctaacttgtacattgaccggtaacgaccaggttggtttcagattattttgtagacaactaggatatttctattcactttatgagacaaaacaataatatgttattgaggtaaattgacttattttggtaatactaactattacatctcaaaatggattaaccctctaacagtcgctatcaaggctaacattcaagattgcggaaatgcaaatgcaccccaatgcaacgcttatacattagtataagtaatgtgcttttttgcaactttggagtgttcagtgcttttatttaaaatttaaacaacaaataaacacgaataaccataatgaatttaattaattatatttattttgtttagctggagtcttttttgtaagtgcttgctctccctggtgttaaaacctggctacaccactggatatcatgctgccaatgttgatggtcaagtagaacaaggaaacgaaggagtcttttttggcggacctggttctccttaaactggtcacccccaaatgacgatacacagggttttataccccctgatccgaaggctatgatgacaaggcctacgaatctcaaccagctttatggggtgaaaaaaggatgtagattaattaattacgtttttgcctataactcgcttaatgtaacttcaacattactcaattttctgataaacaatttgtcgtatatagaacgctctgtaaaagagaaaaaatgaattttgaaggttatttaggatacttgactcgttcgttagcttggattttgcaacccgaaaaagtttggcgttagaagttgttgggtgaagttttttacaacaaaaaataaataattatgatttggcaagcaattattcactcaatgcatatatgagctttcatcgttggaagaatcgtagagtcgtaaccctaatgagcagatccttattcatatccagtaaaagttgaaattgcactttatgcaaatattgtcgatttatatcttattgattgttcattgatttgattaatgatttacttgattttaattgacgtaactggcttgatgatgtttgtaaataaagttgaattattgaaatgccaaagaattttaaattttaaacatgtatttggcaataaaattttaaaagtggctagttgttttgattaaaaaaagttctttatttgatttgtgccctgatttccttttagagtgttagctgtggtgatcaaatgagttgaatttgttaagaaacaaaaaggcactaaatatacatttttatttaaactaaaactaaaccatcaactaaaaagttttaattaaactattagttacaactgcttttaaaagttttcaactaaactggaaacaaaataattaaacttttcttaaccttttctgtcaaatttctggcatattcattatatcaaaatatcagtggacaaaatgactcactgttacaaattttatttcatatcaataaaaccgcaattagtaaccaaaatttatgacatgataacatttagactgaaatttctttctgatttcaatcttgatcaaagaaaaatcagcatgtcaatggcttcgtcagccagtttgcatctacgagcagtcaacatctgtacagctgcactgaacagtccttcaacagttgcagaacatggatatgtaaaccttgttggcttgaaaagttcagaaaggctcaatactaaattttgtatgaaagaaatttagagctggacataacagtgccgcagatccgtttattaaaaaaaatgtttagcttgtatattgatttacactaaaatgttaggcataatatttggatattttattatttgtattttatttaattacaatcagacatatttcattgtaagatttccatgaacgagtcaaatttattacatttaaaactttttctgcatatacatttatgtttgttttttttt*ag*aaaaaacca**tgc**gaacccggtcaaagg**tgc**caatttccg**agaggagat**gcagatccctac**tgc**gca**taa**aatccgaagataaaaaataattcactcattctttagttgaacatgagttgaagaatcacagtaaaataaatacattcaaaattggagattaaatttttaattaaaatattctagtatttcttttagaaaggacaattttcaataaatattttaagaatttagatgacatttcgtgaaaaaaggacttttaaaattttaatacttttagccaccttttaacctttttaacctttcatttcatatctaactaccgagcattctgttttgaaacttaaaattaaaatcctgactaaaatggaaaaaaaatccatttttgaagttcttctgcatttc*ag*gaaaggcat**tgc**gaagcggatgaaaaa**tgc**aagaaagtgaacgacaagttggag**tgt**atcgaaa*gt*gagcttctttatgttgtttatctaacaatgaacattccgacaagaggggtggtgatgattatgatgatgatgatgttgatgatgatgatgatgatgatgacgattaaagatcagaacaacaggcctggctttagaataattcctctttttttgtggacttatgtttgcagaaaatcgagatattattattcaatgtgaaataacgaaatgaacaaaagctagtcactaaaaaatatattcttcactttattactaatattgctaatatttctagagttgattttcttagcaccatgcaagtaaggccaaagccgcctacatatcaataaactaacaaataaaatcttccatttactattgcttttagtgatgaagttattttgattcaaataatcaaattatttaaaacaattatttcattatcaatgagaaactgaagaattattaataagttatgttattagatacaaacaacattattaacttc*ag*atgctgtgcct**tgc**gagaatgcaccattggacaaatactaccgaccagttaatcca**tgc**att**tgt**agttat*gt*cagtccttcttaattttatttaattcttaatttaattgacacttgttaatttcgtttaatttttaagttacatggtactagtttattttattaccgaaggacactcgtagattcaatttgtccattaatttcatagttcattacatttgaaatttgaaaatatttgtttaaaaccattatgcctactttaattcatgttattaattac*ag*ggga*ag*cca**tgc**aagaagcacgaatat**tgt**cggctctcc**tatggagac**aacatcaacgaa**tgt**gtttcaa*gt*aagctggcgtcccagtacattattaat**taa**ccattttcgccaagattcacgtgattaacttccacaatgatttatgttaaaactttttcg*at*agtacccccaa**tgc**aagacgtggtcgattgaaaagaagacaaaaact**tgt**ata**tgc**ggc*gt*tagtatttaccgacaacatactgcactggaatcataaatacaattttcgatttgacaggcgattgattacactgtgcataatcatagtttaatagttaacacttattttatacaaacgaaagggcaaagtatgttgcaaaggaa*ag*gtt**tgc**aaatggataaaa**tgc**aaagaaaga**tgt**ttaaaag*gt*tttttaattgttttatcacaaatcagcattcaaaaccaaaaaataaaataaaataaattaatgaagtattaaaaattcttatttaaca*ag*ataaaggtgagaataaacattcgagaaaaaccaagaaacccaagaacagtcgtacccttagtcagggtcaatctgat**taa**

gaaaggcat**tgc**gaagcggatgaaaaa**tgc**aagaaagtgaacgacaagttggag**tgt**atcgaaa

atgctgtgcct**tgc**gagaatgcaccattggacaaatactaccgaccagttaatcca**tgc**att**tgt**agttat

gggaagcca**tgc**aagaagcacgaatat**tgt**cggctctcc**tatggagac**aacatcaacgaa**tgt**gtttcaa

agtacccccaa**tgc**aagacgtggtcgattgaaaagaagacaaaaact**tgt**ata**tgc**ggc

gtt**tgc**aaatggataaaa**tgc**aaagaaaga**tgt**ttaaaag

ataaaggtgagaataaacattcgagaaaaaccaagaaacccaagaacagtcgtacccttagtcagggtcaatctgat**taa**

AVP**C**ENAPLDKYYRPVNP**C**I**C**SYGKP**C**KKHEY**C**RLS**YGD**NINE**C**VSKYPQ**C**KTWSIEKKTKT**C**I**C**GGKV**CC**KGKV**C**KWIK**C**KER**C**LKDKGENKHSRKTKKPKNSRTLSQGQSD-

ERH**C**EADEK**C**KKVNDKLE**C**IENAVP**C**ENAPLDKYYRPVNP**C**I**C**SYGKP**C**KKHEY**C**RLS**YGD**NINE**C**VSKYPQ**C**KTWSIEKKTKT**C**I**C**GV**C**KWIK**C**KER**C**LKDKGENKHSRKTKKPKNSRTLSQGQSD-

Theoretical pI/Mw: **9.42** / 12930.14

**Htia_DV6 (this study)**

assembled cDNA of Htia_DV6 based on the transcriptome data of Zhou et al. 2024:

**atg**aagcaactgataatatgttttttcctgtcgttggcatttgcaACCGACAACTTAAAAGAT**TGT**CGCTTGGTGTCTAAAGATTTCAGAGGACGTCCCAGAAGTGCC**TGC**AAG**TGT**GGGGAAAGAGAG**TGC**AAAAAGTTCGAAGAT**TGC**GTGTTTTCAAATGAAAATGAACCTGATTAT**TGC**CGGCAATTTGAACCTGAT**TGC**ATCGGTAACTCGGACAAAGAAGTGTGGGGA**TGT**GTT**TGC**GGAGAAAGGCAG**TGT**GAAGCGGATGAAAAA**TGC**AAGAAAGTGAACGACAAATTGGAG**TGT**ATCGAAAATCCTGTGCCT**TGC**GAGAATGCACCATTGGACAAATACTACCGACCAGTTAATCCA**TGC**ATT**TGT**AGTTACGGGAAGCCA**TGC**AAGAAGCACGAATAT**TGT**CGGCTCCCC**TATGGAGAC**AACATCAACAAA**TGT**GTTTCAAAGTACCCCCAA**TGC**AAGACGTGGTCGATTGAAAAGAAGACAAAAACT**TGT**ATA**TGC**GGCGGCAAAGTA**TGT**CACAAAGGAAGGGTT**TGC**AAATGGATAAAAGGCAAAGAAAAA**TGC**TTAAAAGATAAAGGTGAGAATAAACATTCGAGAAAAACCAAGAAACCCAAGAACAGTCGTACCCTTAGTCAGGGTCAATCTGAT**TAA**

MKQLIICFFLSLAFATDNLKD**C**RLVSKDFRGRPRSA**C**K**C**GERE**C**KKFED**C**VFSNENEPDY**C**RQFEPD**C**IGNSDKEVWG**C**V**C**GERQ**C**EADEK**C**KKVNDKLE**C**IENPVP**C**ENAPLDKYYRPVNP**C**I**C**SYGKP**C**KKHEY**C**RLP**YGD**NINK**C**VSKYPQ**C**KTWSIEKKTKT**C**I**C**GGKV**C**HKGRV**C**KWIKGKEK**C**LKDKGENKHSRKTKKPKNSRTLSQGQSD-

TDNLKD**C**RLVSKDFRGRPRSA**C**K**C**GERE**C**KKFED**C**VFS**NEN**EPDY**C**RQFEPD**C**IGNSDKEVWG**C**V**C**GERQ**C**EADEK**C**KKVNDKLE**C**IENPVP**C**ENAPLDKYYRPVNP**C**I**C**SYGKP**C**KKHEY**C**RLP**YGD**NINK**C**VSKYPQ**C**KTWSIEKKTKT**C**I**C**GGKV**C**HKGRV**C**KWIKGKEK**C**LKDKGENKHSRKTKKPKNSRTLSQGQSD

Theoretical pI/Mw: **8.92** / 23259.72

putative Htia_DV6k cDNA:

**atg**aagcaactgataatatgttttttcctgtcgttggcatttgcaAATCCTGTGCCT**TGC**GAGAATGCACCATTGGACAAATACTACCGACCAGTTAATCCA**TGC**ATT**TGT**AGTTACGGGAAGCCA**TGC**AAGAAGCACGAATAT**TGT**CGGCTCCCC**TATGGAGAC**AACATCAACAAA**TGT**GTTTCAA*gt*aagctggcgtcccagtacattattaat**taa**

MKQLIICFFLSLAFANPVP**C**ENAPLDKYYRPVNP**C**I**C**SYGKP**C**KKHEY**C**RLP**YGD**NINK**C**VSSKLASQYIIN-

**chromosom 6 position 15387745 - 15388840**

**atg**agagctttgataatttgccttgtcttgtctttggtcatcgcaagtggagtttctcctacag*gt*ttaaaatattttcgatttattgattaaaataagttttatgtatctacattttttgagtttctgttaattttaaatttatacaatcatttcaaatccaattttgcaagtgaatttgaattacagattattccaactaaattacctaagaaaatatgaagggatatttctaaatgtccacatgatattctatttagctcaatagattttaggcctttcactaaataattcttatttaaattttattaatagatcaataattatgaacactataattacactgaacaaaaaaaaccgacttatctcgggtatcttgatatccaataacagttcttagtaaatttaaacatgataattttgaatatgtaaactatttatctctatcataatatgtgtacaatatatattttaagttgctaaacgtcatattttagcaaataaattcttttgtttgacaatttctgaaatatttggcctgagagcaaaacgcttttcttaagaaaaacttaatgacgtataaaatatcttcatcttgttttacgctgtgattaatgattaacaatcatttttaaatggatatatgtatgaaacatgaaacttattattatgttac*ag*caggtgcaacatat**tgc**ggaggtagtggtgacaaacaggac**tgc**ata**tgt**aat*gt*aagactcctaattccattaattagtatttcctattgaataacatttgctaaaatggtattgcttttaataatattaattttttaataataatttcaaaatccaagtaatgttcaatatatttagcttgtatattgatttagactaaaatgttaggcataatatttggatattttattatttgtattttatttaattataatcagacatattttattgtaagatttccatgaacgagtcaaatttattacatttaaaactttgtctgcatataaatttatgtgtttttttatc*ag*aaaaaacca**tgc**gatcccggtcaaagg**tgc**caatttccg**agaggagat**gcagatccctac**tgc**gca**taa**

**atg**agagctttgataatttgccttgtcttgtctttggtcatcgcaagtggagtttctcctacag

caggtgcaacatat**tgc**ggaggtagtggtgacaaacaggac**tgc**ata**tgt**aat

aaaaaaccatgcgatcccggtcaaaggtgccaatttccgagaggagatgcagatccctactgcgca**taa**

MRALIICLVLSLVIASGVSPTAGATY**C**GGSGDKQD**C**I**C**NKKP**C**DPGQR**C**QFP**RGD**ADPY**C**A-

ATY**C**GGSGDKQD**C**I**C**NKKP**C**DPGQR**C**QFP**RGD**ADPY**C**A

Theoretical pI/Mw: **6.14** / 4069.52

**decorsin_Htia2 (Zhao et al. 2024)**

**Htia_DV2 (this study)**

**chromosom 6 position 15391995 - 15393089**

**atg**agagctttcatcatttgcctcgtcttgtctttggtcatcgcaagtggagtttctcctactg*gt*ttaaaataatttcgatttattgatttaaataatttttatgtatctatattttttgagtttcagttaaatttaaatttatgcaaacttttccaaatccaattttgcaagtgaatttgaattacagattattctaaaaaaattccctaagataagatgaaggaatatgtctacatgtccacatgatattctttgcagctcatgagatattagacatttcactaaataattcttatttaaattttattaatacctcaatgataataaacactataattacactgaacaaaaaaaaaccgacttatctcgggtatcttgatatccaataacagttcttagtaattttaaacatgctaattttgaatatgtaaactatttatctctatcataatatgtgtacaatatatattttaagttgctaaacgtcatattttagcaaataaattcttttgtttgacaatttctgaaatatttgacctgagagcaaaacgcttttcttaagaaaaacctaatgacgtagaaaatttcttcatcgttttacgctgtgattaatgattaacaatcatttttaaatggatatttgcatgaaacatgaaacttattattatgttac*ag*caggtgcaacatat**tgc**agaggtagtggtgacaaacaggac**tgc**ata**tgt**aat*gt*aagactcctaatttcattaattagtatttcctatagaataacatttgctaaaatggtattgcttttaataatattaattttttaataataatttcaaaatccaagtaatgttcaatatatttagcttgtatattgatttagactaaaatgttaggcataatatttggatattttattatttgtattttatttaattataatcagacatattttattgtaagatttccatgaacgagtcaaatttattacatttaaaactttttctgcatataaatttatgtttttttattc*ag*aaaaatcca**tgc**gatcccggtcaaagg**tgc**caatttccg**agaggagat**gcagatccctac**tgc**gca**taa**

**atg**agagctttcatcatttgcctcgtcttgtctttggtcatcgcaagtggagtttctcctactg

caggtgcaacatat**tgc**agaggtagtggtgacaaacaggac**tgc**ata**tgt**aat

aaaaatcca**tgc**gatcccggtcaaagg**tgc**caatttccg**agaggagat**gcagatccctac**tgc**gca**taa**

MRAFIICLVLSLVIASGVSPTAGATY**C**RGSGDKQD**C**I**C**NKNP**C**DPGQR**C**QFP**RGD**ADPY**C**A-

ATY**C**RGSGDKQD**C**I**C**NKNP**C**DPGQR**C**QFP**RGD**ADPY**C**A

Theoretical pI/Mw: **6.14** / 4154.58

**decorsin_Htia1 (Zhao et al. 2024)**

**Htia_DV1 (this study)**

**chromosom 6 position 15395084 - 15397517**

**atg**aagcaactgataatatgttttttcctgtcgttggcatttgcaagcg*gt*gagacatattattttgaatattatgaatgtttatttaaatggtttccatcaaatatttcaaattttctagagttctttcaaagtaatagttttacatttgcaagtgattcgtatgaagggagcttggaaaagatctaaacttacattaattaaatcaatcctagaataatttcagttcaatttcagttgggttttaaaattattttcaaacaattgagatgatcaagaacgtgctaattcaatgcgaagaaattaagtgaaagggaattgaagaataattttaaaagccaagttaaataacaatttcaattaacaacaaaaattagtgaatgattaattatgtaattaggtggtttgaaacctaaattaatcatccaatgaagattgcaattaaatatttaaatgc*ag*acaacttaaaagat**tgt**cgcttggtggataaagatttcagaggacaacccataagtgcc**tgc**aag**tgt**ggg*gt*aataacaatttataataacattaatactttaaagtttatcaacataagcacattgaacactatttgttattattgtttaaaaaaattatttatttattttaaagttattgaaaggtgattaaattaaaaactcatttgttacttgtgactttaagatgatcatgaaagcatcattttcttc*ag*gaaagagag**tgc**aaaacgttcgaagaa**tgc**gtgttttca**aatggaaat**gaacctgaccat**tgc**cagagaa*gt*atcgattcattacttctttttgataatctatgctttatttttaaatattaaacaatgcaatcgaagtattaaaaatatacaaaatcgaatttgattacagagcaaaaacgcgaaaattactgttgaattgtgttagattaaatgttaagataatcatttccgtatccaggtaaaaatacatagttttttttatatccc*ag*atatattaatacctagt**tgc**atcggtgaccccgagaaagaaggatgggga**tgt**gtt**tgt**gga*gt*aagcaaaattatagttattttaatttttaaatattcattattcattattcattattagaaatatagctaaatagcgcagttactactaacatgaatatctaacttctgagcattctgttttgaaacttaaaattaaaatcctgataaaattgaaaaaaaaaaccttttgaagttcttctgcatttc*ag*gaaagggtg**tgc**cacatgaatgaaagc**tgt**aagaaagttaacgacaaattggaa**tgt**ttcaaaa*gt*gagcttcattatcttttttaaataatatgaacaggacgccggggtgatgatgatgatgatgatgacgacgacgacgacgatgatgatgaagatgatgatgacgattaaagatcgagacaagaggcctgggtttagaataatttactatgagtaattattgattttgatttataacatttaaaataatcattttagtatcaataagaaactgatgaattattaataagttatgttattagatacaaataactttattaacttc*ag*aacttgtgcct**tgc**gagtatgtagaaatggacaaattcaaccgaccagttaatcca**tgc**att**tgt**agttat*gt*cagtcgttaattttatttaattcttaatttaattgacccttgttaatttcgattaatttttaagttagctggtaatagtttattttattaccgaaagacactcgtagattcaatttgtccatttatttcataattcattacatttgaaatttgaagttatttgtttaaaaacattatgcctatgaattcatgttattatttata*ag*ggcaagcca**tgc**aaggagcacgaatat**tgt**gagcttggc**aatggagaa**aacatcaacgaa**tgt**atttcaa*gt*aagatggcgtccaag**taa**aatattaattaaccattttcgccaagatttacgtgattaacttccacaatgatttatgttaaaactttgtcgat*ag*agtacccccaa**tgc**aagacgtggtcgattgaaaagaagacaaaaact**tgt**ata**tgt**ggc*gt*tagtatttaccgacaacatactgcactggaatcataaatacaattttcgatttgacaggcgattgattacactgtgcataatcatagtttaatagataacgcttattttattcaaacgaa*ag*ggcaaagta**tgt**tccaaaggaagggtt**tgc**aaatggataaaaggcaaagaaaaa**tgc**ttaaaag*gt*tatttaattgttttatcacaaatcagcattcataaaccaaaaaataaaataaaataaattaatgaagtattaaaaattcttatttaaca*ag*ataaaggtgagaataaacattcgagaaaaaccaagaaacccaagaacagtcgtacccttagtcagggtcaatctgat**taa**

**atg**aagcaactgataatatgttttttcctgtcgttggcatttgcaagcg

acaacttaaaagat**tgt**cgcttggtggataaagatttcagaggacaacccataagtgcc**tgc**aag**tgt**ggg

gaaagagag**tgc**aaaacgttcgaagaa**tgc**gtgttttca**aatggaaat**gaacctgaccat**tgc**cagagaa

atatattaatacctagt**tgc**atcggtgaccccgagaaagaaggatgggga**tgt**gtt**tgt**gga

gaaagggtg**tgc**cacatgaatgaaagc**tgt**aagaaagttaacgacaaattggaa**tgt**ttcaaaa

aacttgtgcct**tgc**gagtatgtagaaatggacaaattcaaccgaccagttaatcca**tgc**att**tgt**agttat

ggcaagcca**tgc**aaggagcacgaatat**tgt**gagcttggc**aatggagaa**aacatcaacgaa**tgt**atttcaa

agtacccccaa**tgc**aagacgtggtcgattgaaaagaagacaaaaact**tgt**ata**tgt**ggc

ggcaaagta**tgt**tccaaaggaagggtt**tgc**aaatggataaaaggcaaagaaaaa**tgc**ttaaaag

ataaaggtgagaataaacattcgagaaaaaccaagaaacccaagaacagtcgtacccttagtcagggtcaatctgat**taa**

MKQLIICFFLSLAFASDNLKD**C**RLVDKDFRGQPISA**C**K**C**GERE**C**KTFEE**C**VFS**NGN**EPDH**C**QRNILIPS**C**IGDPEKEGWG**C**V**C**GERV**C**HMNES**C**KKVNDKLE**C**FKKLVP**C**EYVEMDKFNRPVNP**C**I**C**SYGKP**C**KEHEY**C**ELG**NGE**NINE**C**ISKYPQ**C**KTWSIEKKTKT**C**I**C**GGKV**C**SKGRV**C**KWIKGKEK**C**LKDKGENKHSRKTKKPKNSRTLSQGQSD-

SDNLKD**C**RLVDKDFRGQPISA**C**K**C**GERE**C**KTFEE**C**VFS**NGN**EPDH**C**QR

NILIPS**C**IGDPEKEGWG**C**V**C**GERV**C**HMNES**C**KKVNDKLE**C**FK

KLVP**C**EYVEMDKFNRPVNP**C**I**C**SYGKP**C**KEHEY**C**ELG**NGE**NINE**C**IS

KYPQ**C**KTWSIEKKTKT**C**I**C**GGKV**C**SKGRV**C**KWIKGKEK**C**LKDKGENKHSRKTKKPKNSRTLSQGQSD

Theoretical pI/Mw: **8.66** / 23209.72

**Htia_DV7 (this study)**

**atg**aagcaactgataatatgttttttcctgtcgttggcatttgcaagcg

aacttgtgcct**tgc**gagtatgtagaaatggacaaattcaaccgaccagttaatcca**tgc**att**tgt**agttat

ggcaagcca**tgc**aaggagcacgaatat**tgt**gagcttggc**aatggagaa**aacatcaacgaa**tgt**atttcaa*gt*aagatggcgtccaag**taa**

MKQLIICFFLSLAFASELVP**C**EYVEMDKFNRPVNP**C**I**C**SYGKP**C**KEHEY**C**ELG**NGE**NINE**C**ISSKMASK-

SELVP**C**EYVEMDKFNRPVNP**C**I**C**SYGKP**C**KEHEY**C**ELG**NGE**NINE**C**ISSKMASK

Theoretical pI/Mw: **4.94** / 6117.96

**Htia_DV7k (this study)**
