# Supplementary material for: The great diversity: monomeric and oligomeric hirudins, hirudin-like factors and decorsins in the Asian medicinal leeches Hirudo nipponia and Hirudo tianjinensis
Source: Parasitol Res. 2026 Feb 7;125(1):18. doi: 10.1007/s00436-026-08634-0 (PMC12882960; doi:10.1007/s00436-026-08634-0)
Supplement: Supplementary file 1 — Supplementary Material 1 (ZIP 660 KB) [file 436_2026_8634_MOESM1_ESM.zip › S7_sources of coi sequences.docx]

Supplementary Information File S7: Sources of *coi* sequences

*Hirudo medicinalis*: isolate HMG2, GenBank: KR066920.1

*Hirudo verbana*: isolate HV2, GenBank: JN083793.1

*Hirudo orientalis*: isolate HO1, GenBank: JN104648.1

*Hirudo troctina*: isolate HT56, GenBank: JQ364946.1

*Hirudo sulukii*: isolate HS7, GenBank: KU216243.1

*Hirudo nipponia*: reference genome, GenBank: GCA_040113095.1

*Hirudo tianjinensis*: reference genome, GenBank: GCA_040113125.1

*Whitmania pigra*: reference genome, isolate K615, GenBank: GCA_041430665.1

*Whitmania laevis*: reference genome, GenBank: GCA_041381185.1

*Whitmania acranulata*: reference genome, GenBank: GCA_041381225.1

*Hirudinaria manillensis*: reference genome, GenBank: GCA_034509925.1

*Hirudinaria bpling*: TH02, GenBank: JQ846012.1

*Hirudinaria thailandica*: voucher A77-3, GenBank: OM415431.1

*Hirudinaria javanica*: isolate HDJV, GenBank: GQ368745.1
