# Supplementary material for: The great diversity: monomeric and oligomeric hirudins, hirudin-like factors and decorsins in the Asian medicinal leeches Hirudo nipponia and Hirudo tianjinensis
Source: Parasitol Res. 2026 Feb 7;125(1):18. doi: 10.1007/s00436-026-08634-0 (PMC12882960; doi:10.1007/s00436-026-08634-0)
Supplement: Supplementary file 1 — Supplementary Material 1 (ZIP 660 KB) [file 436_2026_8634_MOESM1_ESM.zip › S8_ sources of ITS sequences.docx]

Supplementary Information File S8: sources of ITS1-5.8srRNA-ITS2 region sequences

*Hirudo medicinalis*: isolate HM33, GenBank: JN119038.1

*Hirudo verbana*: isolate HV47, GenBank: JN119016.1

*Hirudo orientalis*: isolate HO10, GenBank: JN119041.1

*Hirudo troctina*: isolate HT2, GenBank: JN119045.1

*Hirudo sulukii*: isolate H3, GenBank: ON649999.1

*Hirudo nipponia*: reference genome, chromosome 10, GenBank: CM079037.1

*Hirudo tianjinensis*: reference genome, chromosome 10, GenBank: CM079104.1

*Whitmania pigra*: reference genome isolate K615, chromosome 4, GenBank: CM084493.1

*Whitmania laevis*: reference genome, chromosome 5, GenBank: CM084284.1

*Whitmania acranulata*: reference genome, chromosome 4, GenBank: CM084255.1

*Hirudinaria manillensis*: GenBank: JX885695.1

*Hirudinaria javanica*: GenBank: JX885696.1
