# Supplementary material for: The great diversity: monomeric and oligomeric hirudins, hirudin-like factors and decorsins in the Asian medicinal leeches Hirudo nipponia and Hirudo tianjinensis
Source: Parasitol Res. 2026 Feb 7;125(1):18. doi: 10.1007/s00436-026-08634-0 (PMC12882960; doi:10.1007/s00436-026-08634-0)
Supplement: Supplementary file 1 — Supplementary Material 1 (ZIP 660 KB) [file 436_2026_8634_MOESM1_ESM.zip › S9_MSA of coi sequences.rtf]

Supplementary Information File S9: MSA of coi sequences

                *        20         *        40         *        60         *        80         *      
Hmed : ATATTAGGTTCTTCTATAAGATCAATTATTCGAATTGAATTGGCACAACCTGGAAAGTTTTTGGGTGATGATCAACTATACAATTCTTTA :  90
Hver : ATATTAGGTTCTTCAATAAGATCTATTATTCGAATTGAATTAGCACAACCTGGAAAATTTTTAGGTGATGATCAATTATATAATTCTTTA :  90
Hori : ATATTAGGTTCTTCTATAAGATCAATTATTCGAATTGAATTAGCTCAACCTGGAAAATTTTTAGGTGATGATCAATTATATAACTCTTTA :  90
Htro : ATATTAGGTTCCTCTATAAGGTCAATTATTCGAATTGAGTTAGCACAACCTGGGAAATTCTTGGGGGATGATCAATTATACAATTCTTTA :  90
Hsul : ATATACAGCTCCTCAATGAGATCAATTATTCGAATTGAATTATCTCAACCTGGAAAATTTCTAGGTGATGATCAACTATATAATTCATTA :  90
Hnip : ATGCTAGGATCCTCAATAAGATCCATTATTCGAATTGAGCTATCACAGCCTGGAAGATTCCTAGGAGATGATCAATTATATAACTCACTA :  90
Htia : ATACTAGGTTCTTCTATAAGATCCATTATTCGTATTGAGTTAGCTCAACCAGGTAGATTTCTAGGGGATGATCAATTATATAATTCATTA :  90
Wpig : ATGTTAGGCTCCTCTATAAGATCAATTATTCGAATTGAATTAGCACAGCCAGGAAGATTCCTTGGAGACGACCAATTGTATAATTCACTA :  90
Wlae : ATATTGGGTTCCTCTATAAGGTCCATTATTCGAATTGAGTTAGCACAACCAGGCAGGTTTCTTGGGGACGACCAGCTATATAATTCATTA :  90
Wacr : ATATTGGGCTCATCCATAAGATCTATTATTCGAATTGAATTATCTCAACCAGGGAGGTTTTTAGGGGATGACCAACTATATAATTCATTA :  90
Hman : ATATTAGGCTCCTCTATAAGAACTATTATTCGAATTGAGTTATCTCAACCAGGTAGGTTTCTTGGGGATGATCAACTTTATAATTCTTTA :  90
Hbpl : ATATTAGGTTCTTCTATAAGAACTATTATTCGAATTGAGTTATCTCAACCAGGTAGATTTCTTGGTGATGATCAACTCTATAATTCTTTA :  90
                                                                                                       
              100         *       120         *       140         *       160         *       180      
Hmed : GTAACTGCTCATGGATTAGTAATAATTTTCTTTATAGTAATACCAATTTTAATTGGTGGCTTTGGAAATTGACTTTTGCCATTAATAGTT : 180
Hver : GTGACTGCTCATGGATTAGTAATAATCTTCTTTATGGTAATACCAATTTTAATTGGTGGTTTTGGAAATTGACTTTTACCTCTGATAGTT : 180
Hori : GTAACTGCTCATGGATTAGTAATAATTTTCTTTATAGTAATACCAATTTTAATCGGTGGTTTTGGAAATTGACTTTTACCATTAATAGTA : 180
Htro : GTAACTGCTCATGGACTAGTAATAATCTTCTTTATAGTAATGCCGATTTTAATTGGTGGGTTTGGAAATTGACTCCTACCATTAATAGTT : 180
Hsul : GTTACTGCTCATGGGTTAGTAATAATTTTCTTTATAGTTATGCCAATTTTAATTGGGGGATTCGGAAACTGACTCTTGCCCTTAATAGTT : 180
Hnip : GTAACTGCTCATGGGTTAGTTATAATCTTCTTTATGGTAATACCAATTCTGATTGGTGGCTTTGGTAATTGACTCCTTCCATTAATAGTT : 180
Htia : GTAACTGCTCATGGGTTAGTTATAATTTTTTTTATAGTAATACCTATTTTGATTGGTGGGTTTGGAAATTGATTATTGCCATTAATAGTT : 180
Wpig : GTAACGGCTCATGGGTTGGTTATAATCTTCTTTATAGTTATACCAATTCTAATTGGTGGGTTTGGTAATTGACTCCTGCCATTAATGGTA : 180
Wlae : GTAACTGCCCATGGGTTAGTTATAATCTTTTTTATAGTTATACCAATCTTAATTGGTGGGTTTGGTAATTGGTTATTACCATTAATAGTA : 180
Wacr : GTAACTGCACATGGGTTAGTTATAATTTTTTTTATAGTAATACCAATTCTTATTGGGGGCTTCGGTAATTGATTGTTACCCTTAATAGTA : 180
Hman : ATTACTGCACATGGACTTATTATAATTTTTTTTATAGTAATACCTATTTTAATCGGTGGGTTTGGTAATTGACTTTTACCGTTAATAATT : 180
Hbpl : ATTACTGCTCATGGACTTATTATAATTTTTTTTATGGTGATACCTATTTTAATTGGTGGTTTTGGTAACTGACTTTTACCATTAATAATT : 180
                                                                                                       
                *       200         *       220         *       240         *       260         *      
Hmed : GGTGCTATTGATATATCATTTCCCCGATTAAATAATTTTAGATTTTGGTTATTACCACCTTCAATAATTATATTATTAAGTTCATCAATA : 270
Hver : GGTGCTATTGATATATCATTTCCTCGGTTAAATAACTTTAGATTTTGATTATTGCCACCATCAATAATTATATTATTAAGTTCATCTATA : 270
Hori : GGCGCTATTGATATATCATTTCCTCGAATAAATAATTTTAGATTTTGATTATTACCTCCATCAATAATTATATTATTAAGTTCATCAATA : 270
Htro : GGAGCGATTGATATATCATTTCCTCGATTAAATAATTTTAGATTTTGATTACTACCACCATCAATAATTATATTATTAAGTTCATCAGTA : 270
Hsul : GGTGCTATTGATATATCATTTCCTCGATTAAATAATTTTAGATTTTGATTATTACCACCATCAATAATTATATTATTAAGTTCATCAATA : 270
Hnip : GGAGCTGTCGATATATCCTTTCCACGTCTAAATAACCTAAGGTTTTGGCTATTACCGCCCTCAATAATTATATTATTAAGTTCATCAATA : 270
Htia : GGAGCAGTTGATATATCATTTCCACGTCTTAATAATCTAAGCTTTTGATTATTACCACCATCTATAATTATACTACTAAGTTCCTCAATA : 270
Wpig : GGAGCCGTAGATATATCATTTCCTCGTCTGAATAATTTAAGATTTTGGTTACTACCCCCTTCAATAATCATATTGCTTAGGTCATCCTTA : 270
Wlae : GGGGCTGTGGATATATCGTTTCCTCGTCTAAATAATTTAAGATTTTGGTTGTTACCCCCATCAATGATTATACTATTAAGGTCCTCTCTA : 270
Wacr : GGAGCAGTAGATATGTCATTCCCACGGTTAAATAATCTTAGATTTTGATTGCTTCCACCTTCTATAATCATACTATTAAGATCTTCAATA : 270
Hman : GGTGCCCCAGATATGGCTTTTCCACGATTAAATAATTTTAGGTTTTGATTATTACCACCTTCATTAACTATATTAGTAAGATCATCAATA : 270
Hbpl : GGTGCACCAGACATAGCTTTTCCCCGATTAAATAATTTTAGATTTTGATTATTACCACCTTCATTAACTATATTAGTAAGATCTTCAATA : 270
                                                                                                       
              280         *       300         *       320         *       340         *       360      
Hmed : ATCGAAAATGGGGTAGGTACAGGATGAACCCTTTATCCTCCTCTAGCAGATAGTATTTCTCATTCAGGCCCATCTGTAGATATGGCTATT : 360
Hver : ATTGAAAATGGAGTAGGAACAGGATGAACCATTTATCCTCCATTGGCTGATAGTGTCTCTCATTCAGGCCCATCTGTAGATATAGCTATC : 360
Hori : ATTGAAAATGGGGTCGGGACAGGATGAACGCTTTATCCACCATTAGCTGATAATATTTCTCATTCAGGTCCATCTGTAGATATAGCCATT : 360
Htro : ATTGAAAATGGGGTAGGAACAGGATGAACCCTTTATCCTCCACTGGCTGATAATGTTTCTCACTCAGGTCCATCTGTAGATATAGCCATT : 360
Hsul : ATTGAAGATGGTGTAGGAACAGGATGAACACTTTATCCTCCATTAGCTGATAATGTTTCTCATTCTGGACCATCTGTAGATATAGCTATC : 360
Hnip : ATTGAAGGGGGGGTTGGAGCAGGCTGAACCCTATATCCTCCCCTATCCGACTCAGTATCCCACTCAGGCCCATCAGTAGATATAGCAATC : 360
Htia : ATTGAAGGTGGTGTAGGAGCTGGATGAACATTATATCCGCCATTATCAGATTCAATATCACATTCTGGTCCGTCAGTAGACATAGCAATT : 360
Wpig : ATTGAGGGTGGTGTAGGTGCAGGGTGAACCCTTTATCCCCCATTATCAGACTCCGTATCTCATTCAGGCCCATCCGTTGACATAGCCATC : 360
Wlae : ATTGAAGGTGGGGTAGGAGCAGGATGAACACTATACCCACCATTATCAGATTCAGTGTCTCACTCAGGTCCATCTGTTGATATAGCAATT : 360
Wacr : ATTGAGGGGGGTGTAGGAGCCGGTTGGACACTTTACCCTCCATTGTCTGATTCAATTTCTCACTCTGGTCCGTCAGTAGATATAGCTATT : 360
Hman : ATTGAATCCGGTGTTGGTACAGGATGGACTATTTATCCACCATTAGCTGATAGAGTTTCTCACTCAGGACCTTGTGTAGATATAGCTATC : 360
Hbpl : ATTGAAACAGGTGTTGGGACGGGTTGAACCATTTATCCTCCGTTAGCAGATAGAGTTTCTCATTCTGGACCTTGTGTAGATATGGCTATT : 360
                                                                                                       
                *       380         *       400         *       420         *       440         *      
Hmed : TTTTCATTACATATAGCTGGGGCGTCATCAATTCTTGGATCTTTAAATTTTATTTCAACTATTATTAATATACGTATTTCTGGAATAAGA : 450
Hver : TTCTCCCTACATATAGCTGGAGCTTCATCAATTCTTGGATCTTTAAACTTTATCTCAACTATTATTAATATACGTATTTCCGGTATAAGA : 450
Hori : TTTTCATTACATATAGCTGGAGCATCATCAATTCTTGGATCTTTAAATTTTATCTCAACTATTATTAATATACGTATTTCTGGGATAAGA : 450
Htro : TTTTCGTTACATATAGCTGGAGCATCATCAATTCTTGGGTCTTTAAATTTTATTTCAACTATTATTAATATACGAATTTCTGGTATAAGG : 450
Hsul : TTTTCATTACATATAGCAGGAGCATCATCAATTCTTGGTTCTTTAAATTTTATTTCAACTATCATTAATATACGTATTAACGGAATAAGA : 450
Hnip : TTCTCACTACATATAGCTGGTGCCTCCTCTATCTTAGGCTCATTAAATTTTATTTCGACTATTATTAATATACGAACTAAGGGTATAAGA : 450
Htia : TTTTCATTACATATAGCCGGGGCATCATCTATTTTGGGATCTTTAAACTTTATTTCTACAATTATTAATATACGAACTAATGGAATAAGA : 450
Wpig : TTCTCATTACATATAGCTGGTGCCTCATCTATTTTAGGGTCATTAAATTTTATTTCGACTATTATAAATATACGAACTAAAGGGATAACA : 450
Wlae : TTCTCATTGCATATAGCTGGTGCATCCTCTATTTTAGGTTCATTAAACTTTATTTCAACAATTATTAATATGCGAACTAAAGGGATAACA : 450
Wacr : TTTTCATTACATATGGCTGGGGCGTCATCGATTTTGGGTTCTTTAAATTTTATTTCTACAATTATAAATATACGTACTAATGGAATAAGG : 450
Hman : TTTTCATTGCATATAGCTGGTGCATCATCTATTTTAGGTTCTTTAAATTTTATTTCTACTATTATTAATATACGAACTAATGGTATAAGT : 450
Hbpl : TTTTCACTTCATATGGCCGGGGCATCTTCTATTCTAGGTTCTTTAAATTTTATTTCCACTATTATTAATATACGAACCAAGGGTATGGGA : 450
                                                                                                       
              460         *       480         *       500         *       520         *       540      
Hmed : TCTGAACGAGTTCCGCTATTTGTATGATCAGTAGTAATTACTACTATTTTATTGCTTCTTTCATTACCAGTATTAGCTGCAGCTATTACT : 540
Hver : TCTGAACGAGTACCTTTATTCGTGTGGTCGGTAGTAATTACTACCATTTTATTACTTCTCTCTTTACCAGTTTTAGCTGCAGCCATTACT : 540
Hori : TCTGAACGAGTTCCATTATTCGTATGATCAGTAGTAATTACCACTATTCTTTTACTTCTTTCACTACCGGTTTTAGCTGCAGCCATTACT : 540
Htro : TCTGAACGAGTACCACTCTTTGTATGATCAGTAGTTATTACTACTATTCTATTACTTCTTTCATTACCAGTTTTAGCTGCAGCCATTACT : 540
Hsul : TCTGAACGAGTTCCTCTATTTGTATGATCAGTAGTAATCACTACTATTTTATTGCTTCTTTCATTACCAGTTTTGGCTGCAGCTATCACT : 540
Hnip : GCTGAACGTGTCCCATTATTTGTTTGATCTGTAATTATTACTACTATTTTACTATTATTATCATTACCAGTTCTAGCAGCAGCTATTACA : 540
Htia : GCTGAACGTATTCCATTATTTGTATGGTCAGTAATTATTACTACTATTCTTCTACTTTTATCATTACCTGTTTTAGCTGCAGCAATTACT : 540
Wpig : ACTGAACGAGTACCATTATTTGTTTGGTCAGTTGTTATTACTACTATTTTATTATTATTATCATTACCAGTTTTAGCGGCAGCTATTACA : 540
Wlae : ATTGAGCGGGTTCCACTATTTGTTTGATCCGTTGTTATTACGACTATTTTACTATTATTATCTTTACCAGTTTTAGCAGCAGCTATTACA : 540
Wacr : GCAGAACGGGTGCCTTTATTTGTTTGATCTGTAATTATTACTACTGTCTTACTTTTGTTATCATTACCAGTATTAGCAGCAGCTATTACA : 540
Hman : AATGAACGAGTTCCATTATTTGTTTGATCTGTTGTAATTACTACTATCTTATTATTACTTTCATTACCTGTGTTAGCAGCAGCTATTACA : 540
Hbpl : AATGAGCGAGTACCCTTATTTGTTTGATCTGTTGTGATCACTACTATTTTATTATTACTCTCATTACCGGTTCTAGCTGCTGCTATTACA : 540
                *       560         *       580         *       600         *       620          
Hmed : ATATTATTAACTGATCGTAATTTAAATACTACTTTTTTTGATCCAATTGGAGGAGGGGATCCAGTCTTATTTCAACATCTATTT : 624
Hver : ATATTGTTAACTGATCGTAATTTAAATACTACTTTTTTTGATCCAATTGGAGGGGGGGATCCAATCTTATTTCAACATCTATTT : 624
Hori : ATATTATTAACCGATCGTAATTTAAATACTACTTTTTTTGATCCAATCGGGGGAGGGGATCCAATTTTATTTCAACATTTATTT : 624
Htro : ATACTATTAACTGATCGTAATTTAAATACTACTTTTTTTGATCCAATAGGAGGTGGGGACCCAGTTTTATTTCAACATCTATTT : 624
Hsul : ATACTACTTACTGATCGAAATTTAAATACTACTTTTTTTGATCCTATTGGAGGAGGAGATCCTATTTTATTCCAACACTTATTA : 624
Hnip : ATACTTTTAACTGATCGAAACCTAAATACTACATTCTTTGACCCTATAGGTGGTGGAGACCCCATTTTATTTCAACACTTATTT : 624
Htia : ATATTATTAACAGATCGAAATCTAAATACCTCTTTTTTTGATCCTATAGGAGGGGGAGACCCAATTTTGTTTCAACACTTATTT : 624
Wpig : ATATTACTTACAGATCGAAATTTAAATACTACTTTCTTTGACCCTATAGGAGGGGGGGATCCTATTTTGTTTCAACATTTATTT : 624
Wlae : ATGTTACTTACAGATCGAAATTTAAATACCACTTTCTTTGATCCGATAGGTGGTGGGGATCCAATTTTATTTCAACACTTATTC : 624
Wacr : ATACTCTTAACCGATCGAAATCTAAATACAACATTTTTTGACCCTATAGGTGGGGGGGACCCAATTTTATTTCAACATTTATTT : 624
Hman : ATGTTATTAACTGATCGTAATTTAAATACTTCATTTTTTGATCCAATGGGTGGTGGAGATCCAGTATTATTTCAACACTTATTT : 624
Hbpl : ATGTTATTAACTGATCGTAATTTAAATACTGCATTTTTTGATCCAATGGGTGGTGGTGACCCTGTATTATTTCAACATTTATTT : 624


degrees of pair wise sequences identities


         Hmed   Hver   Hori   Htro   Hsul   Hnip   Htia   Wpig   Wlae   Wacr   Hman   Hbpl 

  Hmed    624     90%    91%    91%    89%    79%    82%    80%    79%    80%    83%    82%
            0     90%    91%    91%    89%    79%    82%    80%    79%    80%    83%    82%
            0      0%     0%     0%     0%     0%     0%     0%     0%     0%     0%     0%

  Hver    565    624     90%    89%    87%    79%    80%    79%    78%    78%    80%    80%
          565      0     90%    89%    87%    79%    80%    79%    78%    78%    80%    80%
            0      0      0%     0%     0%     0%     0%     0%     0%     0%     0%     0%

  Hori    574    565    624     91%    88%    79%    83%    80%    80%    79%    82%    81%
          574    565      0     91%    88%    79%    83%    80%    80%    79%    82%    81%
            0      0      0      0%     0%     0%     0%     0%     0%     0%     0%     0%

  Htro    569    557    568    624     87%    81%    82%    81%    80%    79%    82%    80%
          569    557    568      0     87%    81%    82%    81%    80%    79%    82%    80%
            0      0      0      0      0%     0%     0%     0%     0%     0%     0%     0%

  Hsul    556    544    551    545    624     79%    82%    80%    79%    80%    81%    80%
          556    544    551    545      0     79%    82%    80%    79%    80%    81%    80%
            0      0      0      0      0      0%     0%     0%     0%     0%     0%     0%

  Hnip    498    499    495    507    498    624     83%    85%    82%    81%    79%    78%
          498    499    495    507    498      0     83%    85%    82%    81%    79%    78%
            0      0      0      0      0      0      0%     0%     0%     0%     0%     0%

  Htia    514    504    523    516    513    522    624     82%    83%    84%    80%    78%
          514    504    523    516    513    522      0     82%    83%    84%    80%    78%
            0      0      0      0      0      0      0      0%     0%     0%     0%     0%

  Wpig    504    498    503    509    504    532    514    624     85%    80%    79%    77%
          504    498    503    509    504    532    514      0     85%    80%    79%    77%
            0      0      0      0      0      0      0      0      0%     0%     0%     0%

  Wlae    498    491    502    504    499    516    523    533    624     81%    80%    77%
          498    491    502    504    499    516    523    533      0     81%    80%    77%
            0      0      0      0      0      0      0      0      0      0%     0%     0%

  Wacr    501    489    496    498    504    509    526    501    508    624     81%    78%
          501    489    496    498    504    509    526    501    508      0     81%    78%
            0      0      0      0      0      0      0      0      0      0      0%     0%

  Hman    519    505    516    515    511    497    502    495    505    506    624     90%
          519    505    516    515    511    497    502    495    505    506      0     90%
            0      0      0      0      0      0      0      0      0      0      0      0%

  Hbpl    515    505    506    504    503    487    492    482    485    487    562    624 
          515    505    506    504    503    487    492    482    485    487    562      0 
            0      0      0      0      0      0      0      0      0      0      0      0
