# Supplementary material for: The great diversity: monomeric and oligomeric hirudins, hirudin-like factors and decorsins in the Asian medicinal leeches Hirudo nipponia and Hirudo tianjinensis
Source: Parasitol Res. 2026 Feb 7;125(1):18. doi: 10.1007/s00436-026-08634-0 (PMC12882960; doi:10.1007/s00436-026-08634-0)
Supplement: Supplementary file 1 — Supplementary Material 1 (ZIP 660 KB) [file 436_2026_8634_MOESM1_ESM.zip › Table S1.docx]

**Table S1:** List of oligonucleotide primers used in the study

Hnip_HV1_fw1: 5`-GAAcatatgtctggaaatttgattgc-3`

Hnip_HV1_fw2: 5`-catatgtctggaaatttgattgc-3`

Hnip_HV1_rev: 5`-ATAAGCTTAGCCAGTATTTTTGAC-3`

Hnip_HV2a_fw: 5`-cagcatttcaaagattgctcag-3`

Hnip_HV2a_rev: 5`-ATAAGCTTAATAATAATCGTAATCAG-3`

Hnip_HV2b_fw: 5`-cagcatttcactgattgctca g-3`

Hnip_HV2b_rev: 5`-GTAAGCTTAATCATACTCATCGTAATCAG-3`

Htia_HV5_fw: 5`-gaaagaccaaagcgctgctcag-3`

Htia_HV5_rev: 5`-ATAAGCTTAATAGTTCGGTCTAAC-3`

Htia_DV3_fw: 5`-GCAACATATTGCAAAGGTAGTGG-3`

Htia_DV3_rev: 5`-TTAAGCTTTATGCGCAGTAGGGATCTGC-3`

Htia_DV4_fw: 5`-GCGAAGGCTTGTACAGGTAG-3`

Htia_DV4_rev: 5`-CCAAGCTTACGCCCTAATTCTGTTTCTC-3`

Htia_DV6k_fw: 5`-AATCCTGTGCCTTGCGAGAATGC-3`

Htia_DV6k_rev: 5`-TGAAGCTTCTATGAAACACATTTG-3`

Hnip_DV4_fw: 5`-AACGACAAATTAGAAGATTGTCG-3`

Hnip_DV4_rev: 5`-TTAAGCTTAATCAACTCGACCCAGAC-3`

Hnip_DV4k_fw: 5`-AAACCTGTGCCTTGCGAGAATGC-3`

Hnip_DV4k_rev: 5`-TTAAGCTTCTATGAAATACAATAGTTGATG-3`
