# Supplementary material for: The great diversity: monomeric and oligomeric hirudins, hirudin-like factors and decorsins in the Asian medicinal leeches Hirudo nipponia and Hirudo tianjinensis
Source: Parasitol Res. 2026 Feb 7;125(1):18. doi: 10.1007/s00436-026-08634-0 (PMC12882960; doi:10.1007/s00436-026-08634-0)
Supplement: Supplementary file 1 — Supplementary Material 1 (ZIP 660 KB) [file 436_2026_8634_MOESM1_ESM.zip › Table S2.docx]

**Table S2**: Abundance of mRNAs in salivary gland transcriptome data sets of *H. nipponia* and *H. tianjinensis*; + indicates presence, - indicates absence; (+) indicates remaining uncertainty

A) *H. nipponia*

| factor | salivary gland transcriptome SRX3466461 (Lu et al. 2018) |
| --- | --- |
| Hnip_HV1 | + |
| Hnip_HV2 | + |
| Hnip_HV3 | + |
| Hnip_HV4 | + |
| Hnip_HV5 | + |
| Hnip_HV6 | + |
| Hnip_HV7 | - |
| Hnip_mHV1 | + |
| Hnip_mHV2 | - |
| Hnip_mHV3 | + |
| Hnip_mHV4 | + |
| Hnip_mHV5 | + |
| Hnip_mHV6 | + |
| Hnip_mHV7 | + |
| Hnip_mHV8 | + |
|  |  |
| Hnip_DV1 | + |
| Hnip_DV2 | + |
| Hnip_DV3 | + |
| Hnip_DV4 | + |
| Hnip_DV4s | - |

B) *H. tianjinensis*

| factor | salivary gland transcriptome SRR26541739 (Zhao et al. 2024) |
| --- | --- |
| Htia_HV1 | + |
| Htia_HV2 | + |
| Htia_HV3 | + |
| Htia_HV4 | + |
| Htia_HV5 | + |
| Htia_HV6 | + |
| Htia_HV7 | + |
| Htia_mHV1 | + |
| Htia_mHV2 | + |
| Htia_mHV3 | + |
| Htia_mHV4 | + |
| Htia_mHV5 | + |
| Htia_mHV6 | (+) |
| Htia_mHV7 | + |
| Htia_mHV8 | + |
| Htia_mHV9 | + |
| Htia_mHV10 | + |
| Htia_mHV11 | + |
| Htia_mHV12 | + |
| Htia_mHV13 | + |
| Htia_mHV14 | (+) |
| Htia_mHV15 | + |
| Htia_mHV16 | + |
|  |  |
| Htia_DV1 | + |
| Htia_DV2 | + |
| Htia_DV3 | + |
| Htia_DV4 | + |
| Htia_DV5 | + |
| Htia_DV6 | + |
| Htia_DV7 | + |
